# Chamber-specific transcriptional responses in atrial fibrillation

**DOI:** 10.1172/jci.insight.135319

**Published:** 2020-09-17

**Authors:** Catherine E. Lipovsky, Jesus Jimenez, Qiusha Guo, Gang Li, Tiankai Yin, Stephanie C. Hicks, Somya Bhatnagar, Kentaro Takahashi, David M. Zhang, Brittany D. Brumback, Uri Goldsztejn, Rangarajan D. Nadadur, Carlos Perez-Cervantez, Ivan P. Moskowitz, Shaopeng Liu, Bo Zhang, Stacey L. Rentschler

**Affiliations:** 1 Department of Medicine, Cardiovascular Division,; 2 Department of Developmental Biology, and; 3 Department of Biomedical Engineering, Washington University in St. Louis, St. Louis, Missouri, USA.; 4 Departments of Pediatrics, Pathology, and Human Genetics, Biological Sciences Division, University of Chicago, Chicago, Illinois, USA.

**Keywords:** Cardiology, Development, Arrhythmias, Cardiovascular disease, Transcription

## Abstract

Atrial fibrillation (AF) is the most common cardiac arrhythmia, yet the molecular signature of the vulnerable atrial substrate is not well understood. Here, we delineated a distinct transcriptional signature in right versus left atrial cardiomyocytes (CMs) at baseline and identified chamber-specific gene expression changes in patients with a history of AF in the setting of end-stage heart failure (AF+HF) that are not present in heart failure alone (HF). We observed that human left atrial (LA) CMs exhibited Notch pathway activation and increased ploidy in AF+HF but not in HF alone. Transient activation of Notch signaling within adult CMs in a murine genetic model is sufficient to increase ploidy in both atrial chambers. Notch activation within LA CMs generated a transcriptomic fingerprint resembling AF, with dysregulation of transcription factor and ion channel genes, including *Pitx2*, *Tbx5*, *Kcnh2*, *Kcnq1*, and *Kcnip2*. Notch activation also produced distinct cellular electrophysiologic responses in LA versus right atrial CMs, prolonging the action potential duration (APD) without altering the upstroke velocity in the left atrium and reducing the maximal upstroke velocity without altering the APD in the right atrium. Our results support a shared human/murine model of increased Notch pathway activity predisposing to AF.

## Abstract 2

Distinct transcriptional changes occur in human left versus right atrial cardiomyocytes in atrial fibrillation, including Notch pathway activation, which alters electric properties and ploidy in murine models.

## Graphical abstract

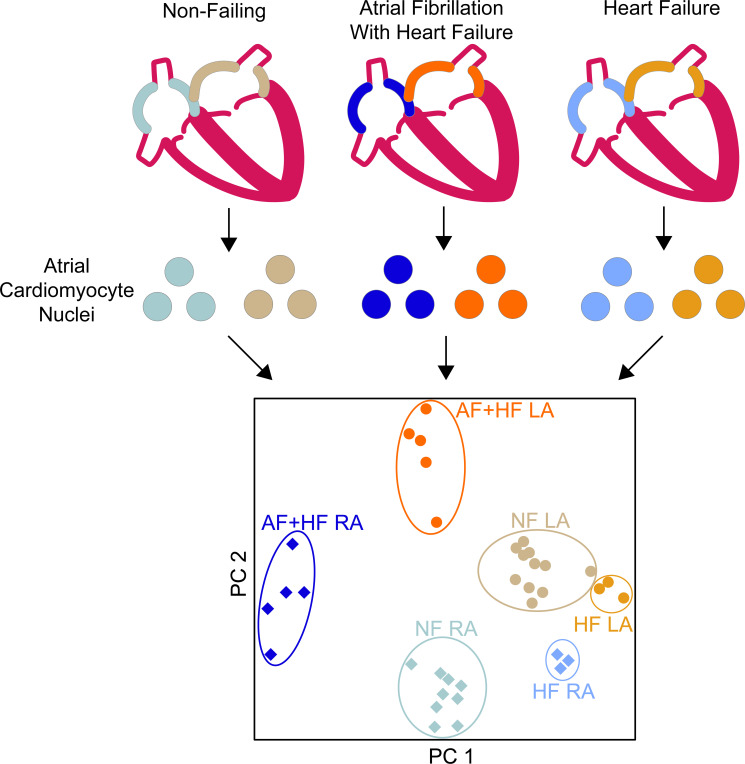

## Introduction

Atrial fibrillation (AF) is the most common cardiac arrhythmia, affecting approximately 2% of the population, and is characterized by rapid and irregular impulse initiation and propagation throughout the atrial myocardium, predisposing to increased risk of stroke, heart failure (HF), and death (1, 2). As many as 50% of patients with severe HF also have AF (3–5), which is not surprising given the number of shared risk factors, including hypertension, obesity, diabetes, and coronary artery disease, underlying the pathophysiology of both HF and AF (6). AF is classified as paroxysmal if sinus rhythm returns spontaneously or following intervention within 7 days, persistent if it lasts greater than 7 days, and long-standing persistent when AF lasts longer than 1 year and is refractory to treatment (7). While HF patients with concomitant AF have higher rates of hospitalization and death (8, 9), current pharmacologic and catheter-based therapies aimed at maintaining normal sinus rhythm remain limited in efficacy. Even for paroxysmal AF, which is the most responsive to treatment, recurrence rates range between 40% and 60% within 1 year after ablation therapy (10, 11). The longer the AF duration, the more likely it is to be resistant to treatment, prompting the dogma “AF begets AF” (12, 13). Despite the high and rising health care burden for AF, the molecular mechanism(s) underlying AF pathogenesis remain largely unknown (2, 14). AF most often results from combined effects of age, genetics, and acquired risk factors. Thus, a more in-depth molecular understanding of disease pathogenesis may enable recognition of patient-specific factors and development of precision medicine–based therapies targeting underlying mechanisms.

AF cellular mechanisms include an initiating trigger, related to Ca^2+^-mediated early afterdepolarizations or delayed afterdepolarizations, combined with a vulnerable substrate myocardium (13, 15). Trigger foci often originate from the pulmonary veins, and in the setting of a vulnerable substrate, reentrant circuits are maintained within the posterior left atrium (LA) (16). A vulnerable substrate can include a variety of cardiovascular pathologies, such as atrial chamber dilation, fibrosis, conduction velocity (CV) slowing, or changes in the effective refractory period (ERP) due to underlying ion channel remodeling (17–19). Though the most common acquired forms of AF are not Mendelian in inheritance, powerful genome-wide association studies (GWAS) have identified associations between genetic variation at more than 100 loci and AF (15, 20–24). Single nucleotide polymorphisms in the regulatory regions cause expression variation in local cardiac ion channel genes implicated in AF trigger and substrate, providing a molecular model for AF risk associations (25, 26). Interestingly, GWAS also identified variation in regulatory regions near cardiac transcription factors (TFs) and modulators of signaling pathways. The susceptibility locus with the strongest association is located in regulatory elements near paired like homeodomain 2 (*PITX2*), which encodes a TF involved in both left-right asymmetry and ion channel gene expression within the LA (24). *Pitx2*-haploinsufficient mice are predisposed to atrial arrhythmias, including atrial flutter and atrial tachycardia, validating *PITX2* as an AF risk locus (27). GWAS loci have also been identified in regions near many other TFs, including T-box transcription factor 5 (*TBX5*). Adult-specific *Tbx5* loss-of-function mice have spontaneous AF mediated through a *Tbx5/Pitx2* gene regulatory network involving membrane effector genes, such as sodium voltage-gated channel alpha subunit 5 (*Scn5a*, which encodes the main voltage-gated sodium channel in the heart, Na_v_1.5), gap junction protein alpha (which encodes connexin 43), and calcium handling genes (28). Therefore, elucidating the gene regulatory networks that maintain atrial rhythm and are altered by genetic perturbation or cardiac insult will be central to understanding AF risk.

Transcriptomic analysis of human atrial tissue will be an important tool for understanding the underlying AF mechanisms. Previous studies aimed at understanding the right versus left atrial transcriptome in nonfailing (NF) hearts, HF, or AF have often focused on the LA appendage (LAA) or right atrial appendage primarily because of tissue availability (29–31), while the LA posterior wall (LAPW) is a key anchor point for atrial reentrant activity (32–34). In addition, previous analyses of bulk tissue are complicated by changes in cellular composition of the LA in AF, including increased fibrosis. To overcome these limitations and better understand the transcriptional changes specifically in cardiomyocytes (CMs) underlying the vulnerable electrical substrate, we isolated and sequenced human right atrium (RA) and LA CM nuclei (CMN) from patients with a history of AF with end-stage heart failure (AF+HF) and with a history of end-stage HF alone (HF). The RA and LA had a largely distinct transcriptional signature in AF+HF when compared with either NF or HF alone, and among many dysregulated genes, we found the direct Notch target hairy and enhancer of split-1 (*HES1*) was significantly upregulated in human LA CMN. In comparison, *HES1* was not upregulated in AF+HF RA CMN or in HF CMN of either atrial chamber.

Here, we observed an association between Notch pathway activation, increased CM ploidy, and AF prevalence. Notch signaling is a strong mitogenic stimulus in developing CMs (35). Shortly after birth, mammalian CMs lose the ability to undergo cytokinesis in response to mitogenic stimuli during cardiac injury or stress but retain the ability to undergo DNA endoreplication (36, 37). As a result, in adult humans CMN transition from a primarily diploid (2n) population to a mixture containing tetraploid (4n) and even higher ploidy (>4n). Very little is understood about the polyploidization process and whether it is protective or maladaptive. While myocardial ischemia and congestive HF have been associated with increased CM ploidy in human ventricular CMs (36–38), it has not been previously demonstrated whether atrial CMs undergo similar changes in the setting of stressors such as AF.

Here, we demonstrate that only LA CMN, and not RA CMN, exhibited increased ploidy in the setting of AF+HF, correlating with the chamber-specific Notch activation. Transient Notch signaling activation in adult murine CMs using genetic models predisposed to reentrant atrial arrhythmias and increased ploidy in murine CMN. Interestingly, Notch activation resulted in distinct transcriptional and electrophysiologic responses in the murine RA when compared with the LA. Specifically, Notch activation in the RA resulted in sodium channel dysregulation and decreased cellular excitability (39). In contrast, genes encoding several potassium channels associated with AF in humans were dysregulated in the Notch-activated LA, giving rise to changes in the action potential duration (APD). Taken together, these data identify gene regulatory networks in human and murine atria that modulate the vulnerable substrate underlying AF and may guide therapeutic strategies.

## Results

### Chamber-specific transcriptomic differences in human cardiomyocyte nuclei in AF.

The murine LAPW has a different embryologic origin than the LAA (40), and LAPW action potentials (APs) are 40% longer when compared with the LAA APs (41), suggesting there is regional heterogeneity within the atria. Previous studies have assessed gene expression in the atrial appendages in the setting of AF (42, 43). To better understand the transcriptional changes accompanying AF that may give rise to the “vulnerable substrate,” we directly assessed changes in the LAPW, an anchor point for reentry, as well as within the RA. Given the diversity of cell types within atrial tissue and the increased fibrosis that is associated with disease states such as AF, we optimized previously described protocols to isolate CMN (44–46). We performed fluorescent activated nuclear sorting (FANS) for pericentriolar material 1 (PCM1), a protein expressed in the nuclear lamina of CMs (46), resulting in a highly pure population of CMN with minimal non-CMN contamination (Supplemental Figure 1; supplemental material available online with this article; https://doi.org/10.1172/jci.insight.135319DS1). We initially performed RNA-sequencing on a small set of NF human ventricular (*n* = 1 right ventricle [RV], *n* = 2 left ventricle [LV]) and atrial (*n* = 2 RA, *n* = 1 LA) samples to validate expected gene expression differences. Atrial markers natriuretic peptide A and sarcolipin were enriched in both the RA and LA, while the ventricular marker myosin light chain 2 was enriched in the RV and LV, as expected. The sequencing depth was sufficient to resolve TFs expressed at low levels in adult hearts, as evidenced by enrichment of *PITX2* in the LA (Supplemental Table 1).

**Figure 1:**
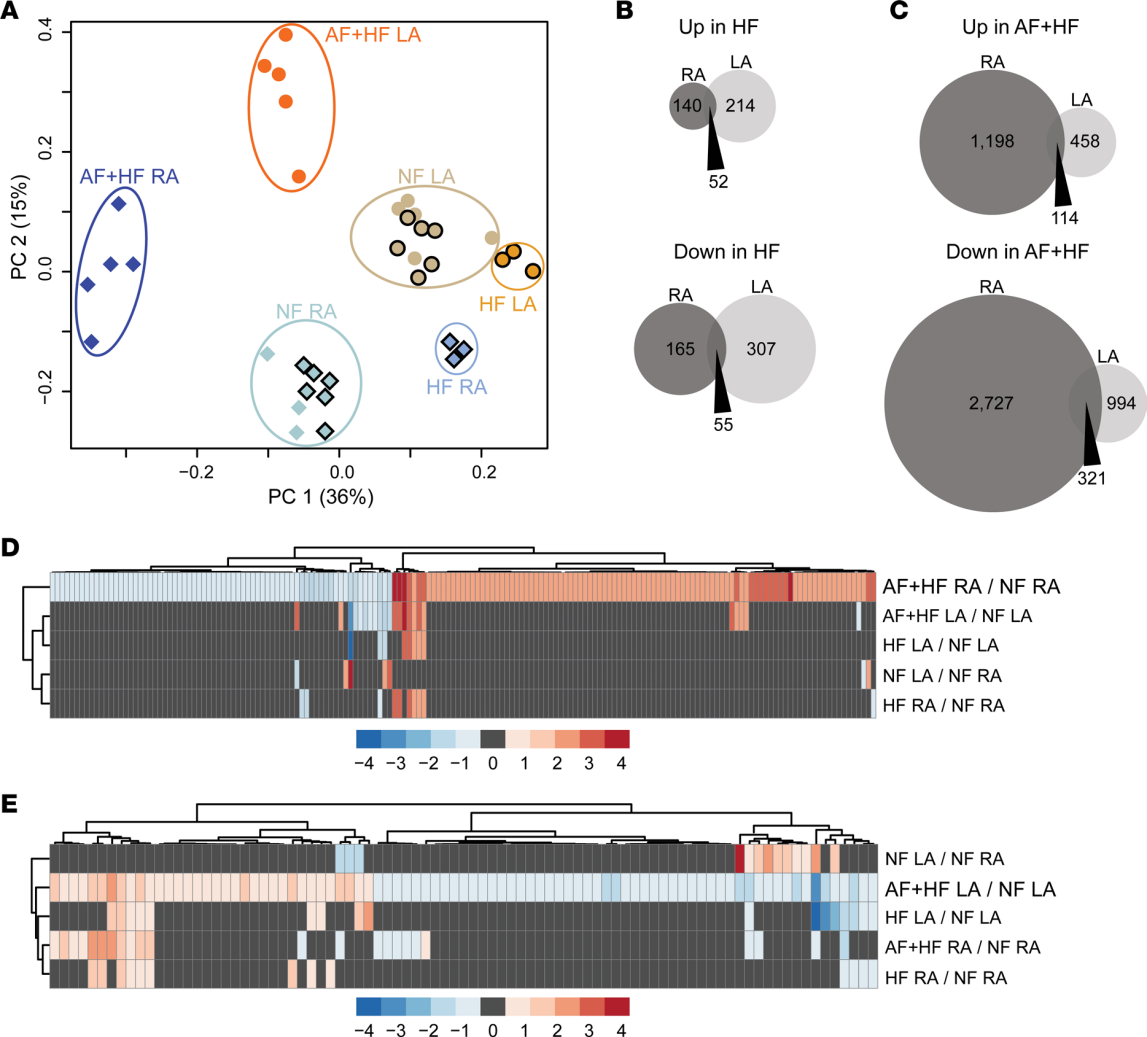
Chamber-specific transcriptomic differences in human atrial fibrillation cardiomyocyte nuclei. RNA-sequencing was performed on isolated cardiomyocyte nuclei (CMN) from human right atrium (RA) and left atrium (LA) from nonfailing (NF) donor hearts rejected for transplantation with no history of atrial fibrillation (AF), explanted hearts from individuals with end-stage heart failure (HF) who received a heart transplant without a history of AF, and end-stage heart failure with a documented history of AF (AF+HF). (**A**) Principal components analysis (PCA) plot showing the distribution of variance among all human samples (NF RA, light teal; AF+HF RA, dark blue; HF RA, light blue; NF LA, brown; AF+HF LA, bright orange; HF LA, orange). Venn diagrams representing the number of differentially upregulated (top) and downregulated (bottom) transcripts in the human RA or LA CMN with (**B**) HF or (**C**) AF+HF when compared with NF controls. (**D**) Heatmap with color scale showing the relative expression levels (*Z* score; red, up; blue, down) of transcription factors (TFs) differentially expressed in AF+HF RA when compared with NF RA. Relative expression levels of the same TFs are shown for each of the other group comparisons, including AH+HF LA compared with NF LA, HF LA compared with NF LA, HF RA compared with NF RA, as well as a comparison of NF LA to NF RA. (**E**) Similar heatmap analysis as shown in **D**, except the TFs shown are those differentially expressed in AF+HF LA when compared with NF LA. RNA-sequencing performed sequentially on 2 batches: batch 1: symbols with black outline on PCA, *n* = 9 individual RA (*n* = 6 NF, *n* = 3 HF) and *n* = 9 individual LA (*n* = 6 NF, *n* = 3 HF). Batch 2: symbols without black outline on PCA, *n* = 8 individual RA (*n* = 3 NF, *n* = 5 AF+HF) and *n* = 10 individual LA (*n* = 5 NF, *n* = 5 AF+HF). Transcripts with a fold change threshold of no more than 0.667 or at least 1.5 and false discovery rate (FDR) less than 0.05 were considered statistically significant.

We investigated global transcriptional differences in human CMN between patients with a history of AF+HF or HF and those without a clinical history of AF or HF (NF). To accomplish this, we performed RNA-sequencing on CMN isolated from right and left atrial tissue of hearts rejected for transplantation without a history of HF (NF controls, *n* = 3–7; Supplemental Table 2), CMN from explanted hearts from patients with end-stage HF undergoing heart transplantation (HF, *n* = 3, Supplemental Table 3), and CMN from patients with end-stage HF who underwent a heart transplant with a documented history of AF on at least 1 ECG (AF+HF, *n* = 5; Supplemental Table 3). Principal components analysis (PCA) to ascertain the variation within our RNA-sequencing data sets demonstrated that samples from each of the 6 groups (NF RA, NF LA, HF RA, HF LA, AF+HF RA, AF+HF LA) clustered together, with AF+HF versus HF versus NF variance mostly comprising the first component and right versus left atria mostly comprising the second component (Figure 1A). A total of 412 transcripts were dysregulated in RA CMN of HF versus NF samples (192 up and 220 down), while 628 transcripts were dysregulated (266 up and 362 down) in the LA CMN of HF versus NF samples using a linear fold change no more than 0.667 or at least 1.5 and a false discovery rate (FDR) less than 0.05, respectively (Figure 1B). Using identical analysis parameters, substantially more transcripts (4360 total, 1312 up and 3048 down) were dysregulated in RA CMN of AF+HF versus NF samples, while 1887 transcripts (572 up and 1315 down) were dysregulated in the LA CMN of AF+HF versus NF samples (Figure 1C). Similarly, there was a low frequency of overlap of transcripts dysregulated in HF alone with those dysregulated in AF+HF. Specifically, of the transcripts dysregulated in AF+HF, approximately 2% (92 of 4360 transcripts in the AF+HF RA) and approximately 11% (200 of 1887 transcripts in the AF+HF LA) were also changed in HF alone. These data suggest that unique transcriptional changes occur with AF in right versus left atria and that greater than 89% of the differentially expressed transcript differences in AF+HF atria are specific to AF and not secondary to HF alone (Figure 1, A–C, and Supplemental Tables 4–8).

Among the many differentially regulated genes in AF+HF LA were subunits encoding ion channels or their interacting proteins, such as potassium voltage-gated channel interacting protein 2 (*KCNIP2*), potassium calcium-activated channel subfamily N member 2, and calcium voltage-gated channel subunit alpha1 D (Supplemental Tables 4, 5, 8, and 9), which were expected based on previous studies associating these ion channels with AF (20, 47, 48). One of the downregulated transcripts in AF+HF LA samples was *PITX2*, which encodes a TF involved in left-right asymmetry. Therefore, we asked whether AF gene expression changes occur predominantly in transcripts differentially expressed between the LA and RA at baseline. There were 1180 differentially expressed transcripts between NF LA versus NF RA at baseline (614 up and 566 down). When comparing AF+HF against NF counterparts, approximately 2% differentially expressed transcripts changed within AF+HF RA (99 of 4360), and approximately 2% (39 of 1887) in AF+HF LA (Supplemental Table 8). Therefore, dysregulated transcripts in AF do not predominantly represent an overall change in left versus right chamber identity.

More than 100 loci have been associated with AF through GWAS (22). While GWAS offers powerful and unbiased genome-wide information, it is not able to provide cell type specificity or specific gene expression changes, which may be caused by the risk variants. To probe this relationship further, we generated a heatmap of genes located near AF GWAS loci and interrogated whether transcriptional changes occurred within CMs in our data set. A total of 18 GWAS-associated transcripts were dysregulated in RA CMN of AF+HF versus NF samples (10 up and 8 down) using a linear fold change no more than 0.667 or at least 1.5 and FDR less than 0.05, while none of these changes were observed in HF alone (Supplemental Figure 2 and Supplemental Table 9). Similarly, 19 transcripts were dysregulated (6 up and 13 down) in the LA CMN of AF+HF versus NF samples, and the majority of these changes (63%) also were not seen in HF alone (Supplemental Figure 2 and Supplemental Table 9). This data set provides a useful resource to guide future cell type–specific experimentation related to the mechanisms underlying AF.

Given the distinct global transcriptomic signatures of right versus left atrial CMN in AF, we asked whether differential regulation of TFs may underlie the atrial gene regulatory programs. Using a fold change threshold of no more than 0.667 or at least 1.5 and FDR less than 0.05, we observed that the majority of TFs were also dysregulated only within the AF+HF RA (Figure 1D; 140 unique TFs) or AF+HF LA (Figure 1E; 46 unique TFs), while only 9 TFs (~5% of total) met these criteria in both atria (Figure 1, D and E; and Supplemental Table 10). Several TFs dysregulated within LA CMN have previously been associated with predisposition to AF both in humans and in animal models, including *PITX2* (fold change = 0.37, FDR = 2.42E-02) (27, 49) and short stature homeobox protein 2 (*SHOX2*; fold change = 0.08, FDR = 2.37E-31) (50, 51). Additional TFs specifically dysregulated within human LA CMN have been linked with atrial gene regulation in murine models, including HOP homeobox (52) (fold change = 3.35, FDR = 8.31E-03) and paralogs of ETS variant 1 (*ETV1*; ref. 53), including *ETV5* (fold change = 2.00, FDR = 1.84E-03) and *ETV6* (fold change = 0.59, FDR = 1.28E-02). Interestingly, several TFs were dysregulated in both HF and AF+HF (*SHOX2* and *ETV6*), while the majority of TFs are unique to AF+HF. These data provide further rationale for investigating a chamber-specific role of TFs in regulating atrial gene networks in the context of AF.

### CM ploidy is increased in atrial CMs in human AF and in Notch-activated mice.

**Figure 2:**
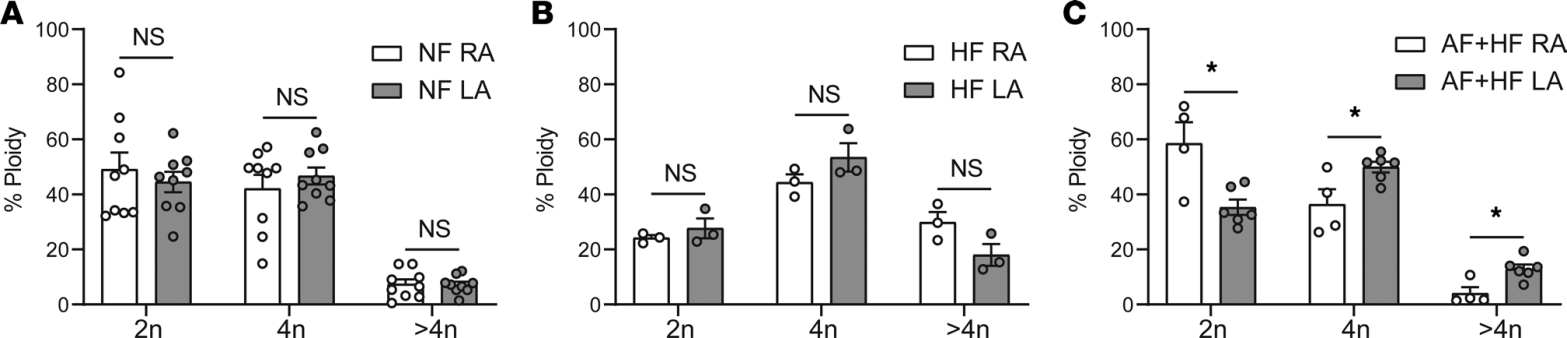
CM ploidy is increased in LA CMs of humans with AF. CMN were isolated from the RA and LA of NF, HF, and AF+HF. Nuclei were sorted based on DAPI and PCM1 staining, and distinct ploidy populations (2n, 4n, >4n) were identified based on differences in DAPI intensity (Supplemental Figure 3). (**A**) CMN ploidy in NF RA versus NF LA is similar. (**B**) CMN ploidy in HF RA versus HF LA is also similar. (**C**) Comparison of ploidy in AF+HF RA versus AF+HF LA shows loss of 2n nuclei, with concomitant increase in 4n and >4n or higher ploidy nuclei in the LA in the setting of AF. Ploidy experiments were performed on 2 sample batches: batch 1: (**A** and **C**) *n* = 9 NF RA, *n* = 9 NF LA, *n* = 4 AF+HF RA, *n* = 6 AF+HF LA; batch 2: (**B**) *n* = 3 HF RA, *n* = 3 HF LA. Data are presented as the mean from each group ± SEM. *P* < 0.05 was considered statistically significant. *P < 0.05; NS, not significant. Unpaired Student’s 2-tailed *t* test with a Welch’s correction was performed for all comparisons.

The FANS protocol described above to sort CMN using PCM1 can resolve distinct ploidy populations based on DAPI intensity (Supplemental Figure 3). At baseline, there were no differences in ploidy between the LA and RA in NF hearts, with a distribution of approximately 47% 2n cardiomyocytes, 45% 4n, and 7% >4n at baseline in both atrial chambers (Figure 2A). Similarly, in HF alone, the ploidy distribution was similar between the RA and LA (Figure 2B). In contrast, in the setting of AF+HF, there was a relative reduction of 2n nuclei in the LA, with concomitant increases in both the 4n and >4n LA CMN populations (Figure 2C). This increase in the >4n population remained statistically significant when directly comparing the AF+HF LA with NF LA (13% versus 7%, *P* < 0.001). To our knowledge, this is the first association of increased CM ploidy with human AF.

**Figure 3:**
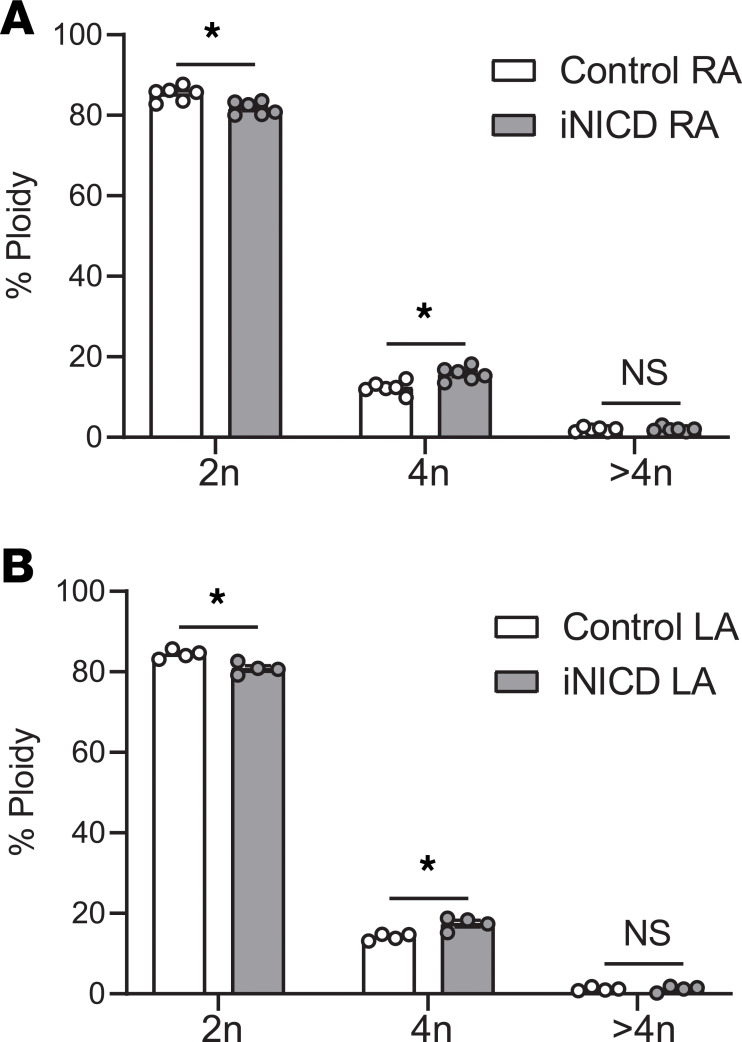
CM ploidy is increased in Notch-activated murine atrial CMs. CMN were isolated from the RA and LA in a murine genetic model of adult Notch activation (iNICD) and compared with littermate controls. FANS was performed for DAPI and PCM1 staining, and distinct ploidy populations (2n, 4n, >4n) were identified based on differences in DAPI intensity (Supplemental Figure 5). At baseline, mice have a higher 2n population (85% in the RA and 84% in the LA) when compared with human atria. Nuclear ploidy distribution of control versus iNICD PCM1-sorted murine (**A**) RA CMN and (**B**) LA CMN. *n* = 6 control RA, *n* = 6 iNICD RA. Two left atria were pooled for each biological replicate; *n* = 4 control LA, *n* = 4 iNICD LA. Data are presented as the mean from each group ± SEM. *P* < 0.05 was considered statistically significant. *P < 0.05; NS, not significant. Unpaired Student’s 2-tailed *t* test with a Welch’s correction was performed for all comparisons.

To better understand factors that could contribute to increased CM ploidy in AF+HF LA, we used a computational tool that integrates machine learning approaches to classify differentially expressed genes into biological categories (CompBio, https://www.percayai.com) and found that Notch signaling was dysregulated in the LA, but not the RA, in the setting of AF+HF. Multiple Notch pathway components were dysregulated in AF+HF, including upregulation of the direct Notch target *HES1* in the LA (fold change = 2.39, FDR = 2.66E-03) but not in the RA (Supplemental Figure 4 and Supplemental Tables 4, 5, and 10). Additionally, the Notch pathway was not upregulated in atrial CMN with HF alone, as only 1 of the 53 genes in the Kyoto Encyclopedia of Genes and Genomes database changed in either HF atria (Supplemental Figure 4 and Supplemental Tables 6 and 7). In the *Drosophila* gut, Notch signaling has been associated with endoreplication (54, 55). Specifically, Delta/Notch signaling induces *Hindsight*, which inhibits cyclin-dependent kinase activity (56). To directly test whether Notch signaling may regulate polyploidization within the atria, we used a doxycycline-inducible genetic model to probe the effects of Notch activation within adult myocardium (inducible Notch intracellular domain, iNICD) (39). In both the RA and LA, transient Notch activation for 3 weeks was sufficient to significantly increase 4n nuclei (13% up to 17%) at the expense of 2n nuclei (85% down to 81%) in mice (Figure 3, A and B; and Supplemental Figure 5), suggesting a potential role for Notch signaling in atrial polyploidization in AF.

### Notch activation differentially regulates the transcriptome of the murine left versus right atrium.

**Figure 4:**
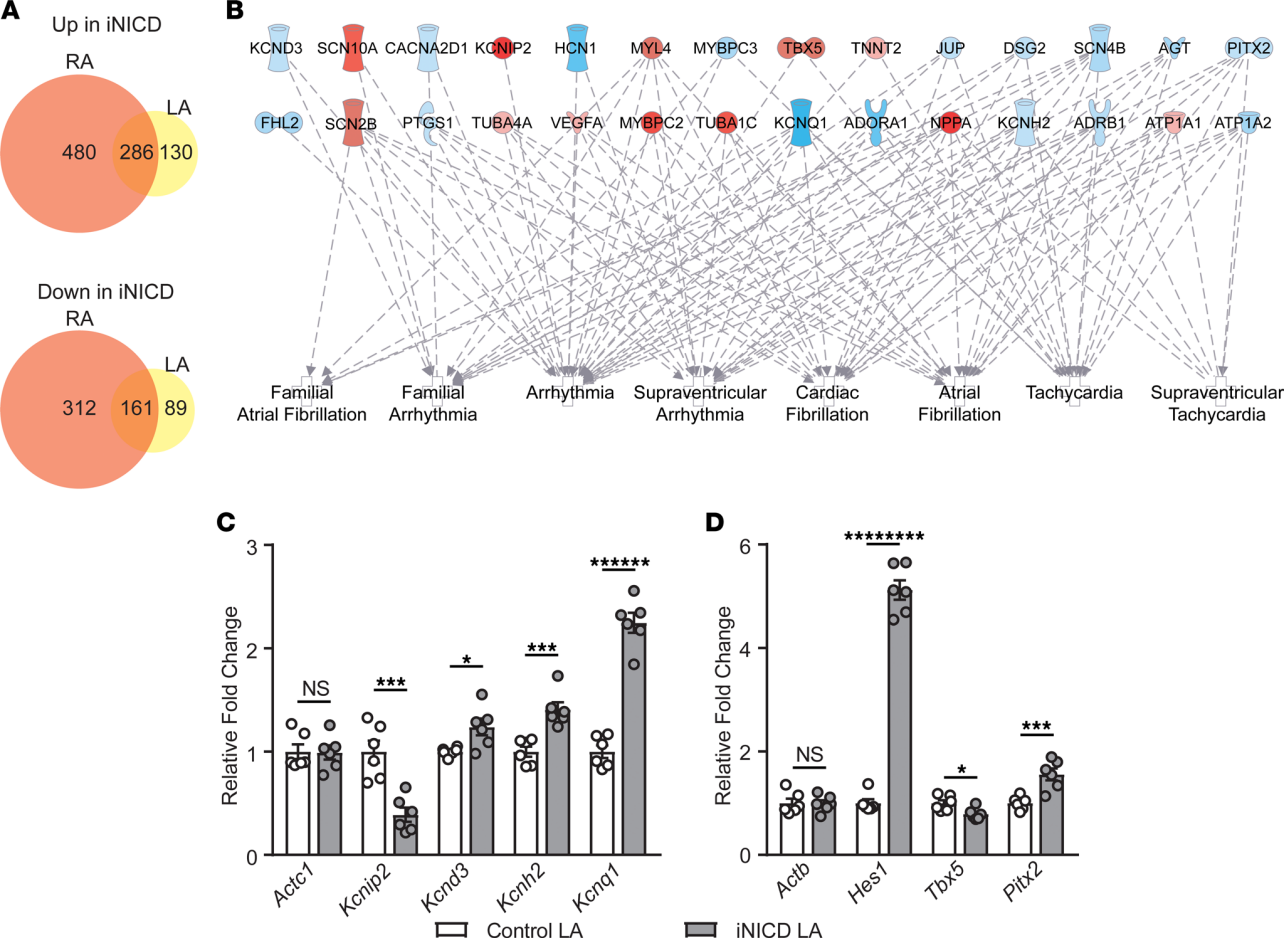
Notch activation differentially regulates the transcriptome of the murine left versus right atrium. Notch signaling was activated in adult CMs (iNICD) followed by RNA-sequencing of the RA and LA. (**A**) The number of significantly dysregulated transcripts in iNICD RA (orange) and LA (yellow) compared with littermate controls is shown, with Venn diagrams demonstrating primarily distinct transcriptional changes within RA and LA. (**B**) Gene ontology (GO) analysis on iNICD LA indicating the statistically significant Ingenuity Pathway Analysis–generated biological diseases that are either general or atrium-specific arrhythmia conditions from the top 25 disease categories and the key differentially expressed genes within the category. Red indicates downregulated, blue indicates upregulated, and color intensity is positively related to fold change. *n* = 6 control RA, *n* = 6 iNICD RA, *n* = 6 control LA, *n* = 6 iNICD LA. (**C** and **D**) Reverse transcription–quantitative polymerase chain reaction (RT-qPCR) validation of genes associated with cardiac electrophysiology and AF was performed on the LA of a second cohort of iNICD mice. K^+^ channel genes (**C**) and transcription factors (**D**) from the GO analysis that are associated with AF. *n* = 6 control LA, *n* = 6 iNICD LA. All fold changes are relative to *Tbp*. Data are presented as the mean from each group ± SEM. *P* < 0.05 was considered statistically significant. *P < 0.05; ***P < 0.001; ******P < 0.000001; ********P < 0.00000001; NS, not significant. Unpaired Student’s 2-tailed *t* test with a Welch’s correction was performed for all comparisons.

Previous work from our group demonstrated that transient Notch activation in a murine model predisposes to reentrant atrial arrhythmias (39). Given the chamber-specific responses in human atria in the setting of AF (Figure 1), we compared gene dysregulation in the Notch-activated LA with the RA from the same animals (Figure 4). A total of 896 genes were upregulated in response to Notch signaling in either the LA or RA, while only approximately 32% (286 genes) were upregulated in both atria (Figure 4A; Supplemental Table 11). Similarly, 562 genes were downregulated in response to Notch signaling, but only approximately 29% (161 genes) were downregulated in both the RA and LA (Figure 4A; Supplemental Table 11). Using Ingenuity Pathway Analysis software, GO analysis on differentially regulated transcripts in the Notch-activated LA revealed that, of the top 25 diseases and functions, 14 were related to arrhythmias, and 8 pathways were related to atrial arrhythmias (Figure 4B; Supplemental Table 12). This transcriptional signature occurred in the absence of structural changes within the LA (Supplemental Figure 6). Validation of the RNA-sequencing for specific AF-associated genes in a second murine cohort demonstrated dysregulation of genes encoding K^+^ channel subunits comprising I_to_ (*Kcnip2*, 0.37 ± 0.11, *P* < 0.001), I_Kr_ (potassium voltage-gated channel subfamily H member 2, *Kcnh2*, 1.4 ± 0.067, *P* < 0.001), and I_Ks_ (potassium voltage-gated channel subfamily Q member 1, *Kcnq1*, 2.3 ± 0.064, *P* < 0.000001) (Figure 4C, Supplemental Table 14). Several TFs associated with AF were also dysregulated in the LA after Notch activation, including the direct Notch target *Hes1* (5.17 ± 0.053, *P* < 0.00000001), *Tbx5* (0.79 ± 0.078, *P* < 0.05), and *Pitx2* (1.5 ± 0.11, *P* < 0.001) (Figure 4D, Supplemental Table 14). Although *Pitx2* has traditionally been thought to be downregulated in human and murine models of AF, these studies have mostly been associated with bulk tissue sequencing, which is not cell type specific. There is also evidence that *PITX2C* is upregulated specifically in CMs of humans with chronic AF, and models of human atrial cells suggest that *PITX2* upregulation can increase the risk of chronic AF (57, 58). These seemingly contradictory results suggest that *Pitx2* levels are sensitive to change in the setting of AF and that either up- or downregulation may be associated with an arrhythmic phenotype.

### The murine LA AP was prolonged after Notch activation and did not respond to I_Kr_ blockade.

**Figure 5:**
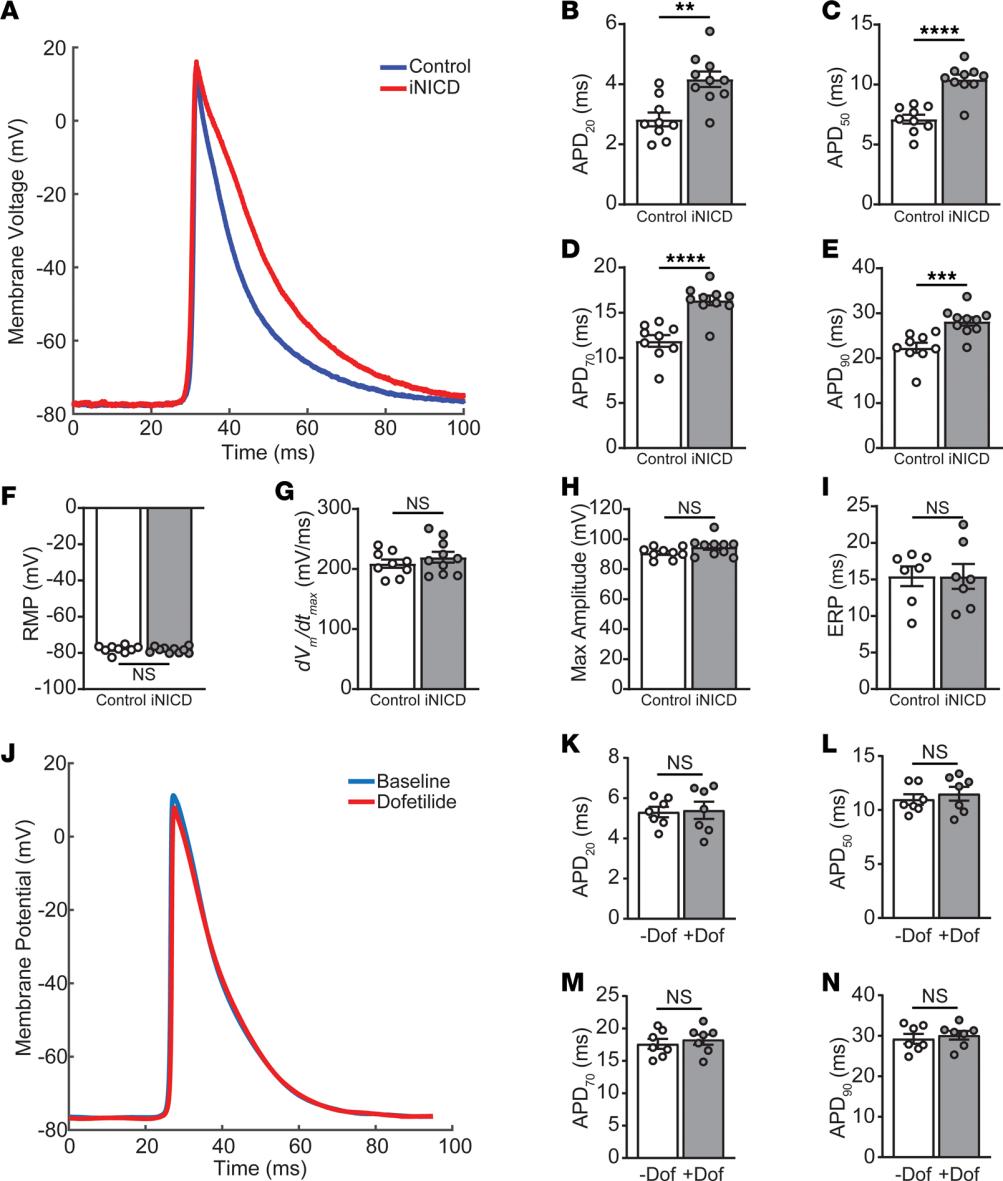
Notch signaling prolongs the murine LA AP and is not responsive to the I_Kr_ blocker dofetilide. Sharp microelectrode recordings were performed ex vivo on the LA of intact murine hearts while in sinus rhythm in control and iNICD mice after a 4-month washout period (**A**–**I**) versus during active Notch activation (**J**–**N**). In addition, during active Notch activation, recordings were taken at baseline (-Dof) versus after 10 nM dofetilide administration, a class III antiarrhythmic drug that blocks I_Kr_ (+Dof, **J**–**N**). (**A**) Representative averaged LA APs in control (blue) and iNICD (red). (**B**–**E**) APDs at 20% (**B**), 50% (**C**), 70% (**D**), and 90% (**E**) repolarization. AP characteristics, including RMP (**F**), *dV_m_/dt_max_* (**G**), max amplitude (**H**), and ERP (**I**). *n* = 9 control mice and *n* = 10 iNICD mice for **B**–**H**. *n* = 7 control mice and *n* = 7 iNICD mice for **I**. (**J**) Representative averaged LA APs in iNICD mice at baseline (blue) and after 10 nm dofetilide administration (red) demonstrate no shortening of the AP, suggesting absence of I_Kr_ current despite transcriptional upregulation of *Kcnh2* in iNICD mice. (**K**–**N**) APDs at 20% (**K**), 50% (**L**), 70% (**M**), and 90% (**N**) repolarization are also unchanged with dofetilide. *n* = 7 iNICD mice. Data are presented as the mean from each mouse ± SEM. *P* < 0.05 was considered statistically significant. **P < 0.01; ***P < 0.001; ****P < 0.0001; NS, not significant. Unpaired Student’s 2-tailed *t* test with a Welch’s correction was performed for comparisons in **B**–**I**. Paired Student’s 2-tailed *t* test with a Welch’s correction was performed for all comparisons in **K**–**N**.

Given distinct transcriptomic changes in right and left atria after Notch activation, we asked whether the cellular electrophysiologic response is also distinct. We previously reported that Notch activation within the RA leads to persistently reduced *Scn5a* expression, CV slowing, and reduced *dV*_m_*/dt*_max_ consistent with reduced sodium current, without affecting the resting membrane potential (RMP) or APD (39). While *Scn5a* expression was downregulated in the RA after Notch activation, in contrast, it remained unchanged within the LA (Supplemental Table 11). In contrast to findings in the RA, we observed LA-specific expression changes in genes encoding subunits of voltage-gated potassium channels (Figure 4C). To determine whether these transcriptional changes may result in functional changes in atrial electrophysiology, we performed sharp microelectrode recordings on Langendorff-perfused murine hearts. Notch signaling was activated for 4 weeks followed by a minimum 4-month washout period to determine whether electrophysiologic effects were persistent. To our surprise, we found that the LA APD was persistently prolonged during all phases of repolarization, including APD_20_, APD_50_, APD_70_, and APD_90_, during sinus rhythm (Figure 5, A–E; Supplemental Figure 7; Supplemental Table 13) while no other measured parameters, including RMP, *dV*_m_/*dt*_max_, AP amplitude, or ERP, were significantly changed in the LA (Figure 5, F–I; Supplemental Table 13). Given that APD can vary based on stimulus cycle length, we also performed sharp microelectrode recordings during 10 Hz pacing and observed consistent APD prolongation during all phases of repolarization in the Notch-activated LA (Supplemental Figure 8; Supplemental Table 13). Therefore, chamber-specific transcriptional responses predict cellular electrophysiology and demonstrate that transient Notch activation in atrial CMs can cause long-term changes in LA electrophysiology.

In rodent atria, high I_to_ current densities dominate during all phases of repolarization and account for the remarkably abbreviated APs lacking a clear plateau phase (59). Although mice express many of the genes encoding subunits of K^+^ channels found in humans, I_Kr_ and I_Ks_ currents contribute minimally to repolarization in adult murine hearts (60–62). The Notch-activated transcriptional signature and directionality of K^+^ channel subunit gene expression matched those observed in human AF, including upregulation of *Kcnh2* (subunit of I_Kr_) and *Kcnq1* (subunit of I_Ks_). However, in contrast to the shorter APD typically observed in the human atria during fibrillation (63), Notch activation prolonged the murine LA APD (Figure 5, A–E). Downregulation of *Kcnip2*, which encodes a subunit of I_to_, is reduced in both our murine model and in human AF (64–66). *Kcnip2* downregulation may contribute to prolongation throughout all phases of the AP observed in Notch-activated mice, while in humans, it may correlate with the prolonged AP duration at 20% repolarization observed in AF (67). To demonstrate whether Notch-mediated increases in *Kcnh2* expression would result in increased I_Kr_ and influence atrial APs, we acutely activated Notch signaling for 4 weeks and found similar APD changes at baseline (Figure 5J) to those that persisted after a prolonged washout period (Figure 5A). Next, we treated Notch-activated mice with dofetilide, the class III antiarrhythmic drug commonly used to treat AF through blocking the I_Kr_ current (Supplemental Figure 7, Supplemental Table 13) (68). Although the APD was prolonged at baseline in the iNICD LA (Figure 5J, Supplemental Table 13), 10 nM dofetilide did not further prolong the APD_20_, APD_50_, APD_70_, or APD_90_ or affect RMP, *dV*_m_/*dt*_max_, or AP amplitude (Figure 5, J–N; Supplemental Figure 7; Supplemental Figure 9; and Supplemental Table 13). Taken together, these data suggest that the murine model recapitulates transcriptional responses seen in humans, although differences in individual currents potentially germane to AF risk exist between species.

## Discussion

One of the most striking and consistent findings across species described herein is the observation of distinct transcriptional networks within the RA and LA. AF is a highly complex and heterogeneic disease (69), which manifests in the presence of various clinical backgrounds ranging from classic systolic HF to lone familial AF (3, 70). This detailed investigation of the transcriptome of human LAPW versus RA CMN determined that more genes are differentially regulated than similarly regulated between human atria in the setting of AF (Figure 1, Supplemental Tables 4 and 5). Here, we provide a resource detailing AF-associated GWAS loci and TF expression changes specifically within human right versus left atrial CMN, which may be used in guiding future cell type–specific experimentation related to AF mechanisms (Supplemental Figure 2, Supplemental Tables 9 and 10). Although there was very little overlap between changes observed in the RA and LA CMN, both chambers exhibited significant transcriptional changes, which may reflect the involvement of both the RA and LA in AF perpetuation. Although AF is frequently considered an LA disease, these data further support the emerging notion that the RA could contribute to AF by mechanisms different from those operative in the LA. The emerging need for extensive ablation of RA sites in addition to pulmonary vein isolation and LA modification in many patients, particularly those with persistent AF, supports this notion.

The AF GWAS patient population used as a reference (22) shares similar comorbidities to the AF samples in this manuscript, including age, hypertension, and diabetes. While the number of patients with HF was not defined for the GWAS, the meta-analysis reviewed approximately 65,000 patients with AF, including approximately 6500 with unspecified HF compared with the end-stage HF AF samples used in this study. It is possible that inherent differences between patient characteristics in the GWAS data set versus our cohort could limit our ability to detect relevant changes. At least 1 patient in the AF+HF group in our study was in sinus rhythm at the time of tissue procurement (paroxysmal AF). Since all AF+HF samples clustered together on the PCA and were separated from HF-alone samples, this suggests a distinct transcriptional profile may exist in AF-susceptible atria independent of the fibrillation process itself. Our finding is consistent with a previous study in dogs demonstrating that HF plus atrial tachycardia (as would occur in a patient with HF who develops AF) produced ionic changes distinct from HF or atrial tachycardia alone and distinct from predicted additive effects of HF and atrial tachycardia (71). Therefore, future studies focused on elucidating the transcriptome throughout various stages of AF, as well as in association with distinct AF-associated clinical entities, may provide additional valuable information.

Chamber-specific findings were also supported in our murine model of Notch activation, where transcriptional changes paralleled distinct electrophysiologic reprogramming between the RA and the LA (Figure 4 and Figure 5). Previous work from our laboratory demonstrated that Notch signaling regulates ion channel expression in part through chromatin modification (39, 72). The transcriptional and electrophysiologic changes persist long after transient Notch activation, consistent with a reprogramming process. It is known clinically that “AF begets AF.” While atrial fibrosis and atrial dilation are known contributing factors to AF perpetuation, it will be interesting to further probe the role of the epigenome in this process.

A recent study demonstrated that HEY1/HEY2 (Hes related family BHLH transcription factor with YRPW motif 1/2) TF binding motifs are abundant in open chromatin located near AF-associated GWAS loci in human LA tissue (26). HEY1 and HEY2 are direct targets of Notch signaling that play important roles in heart development (73). Interestingly, although we did not detect *HEY1* or *HEY2* expression in human AF+HF atrial CMN, the Notch downstream target *HES1* is upregulated specifically in human AF+HF LA but not RA (Supplemental Figure 4, Supplemental Tables 5 and 10). The HES1 consensus binding motif (C A/G CGTG) (74) is very similar to the HEY1/HEY2 motif (CACGTG) (75). Given that Notch activation upregulates *Hes1* in the murine LA, creating a transcriptional signature resembling AF, including dysregulation of *Pitx2* and *Tbx5* (Figure 4, B–D), ongoing and future work will further probe these gene regulatory networks driving AF susceptibility and perpetuation.

Human CMN are primarily diploid but become tetraploid and even octoploid in the setting of heart dysfunction, suggesting there may be activation of regenerative signaling pathways associated with new DNA synthesis without subsequent nuclear division (37, 38). Furthermore, there is evidence that failing hearts that undergo mechanical unloading through left ventricular assist device placement revert from primarily polyploid to diploid (76). We provide the first evidence to our knowledge that ploidy is increased in human atrial CMN in the setting of AF+HF, specifically in the LA (Figure 2). In addition, we show that Notch activation in adult atrial CMs is sufficient to induce polyploidization (Figure 3). The potential benefits of polyploidization in the heart are not fully understood (77). One potential evolutionary benefit could be to enable an increase in the metabolic capacity of a nonregenerative tissue such as the heart through funneling energy into gene duplication and additional protein synthesis without requiring dissolution of sarcomeres, which might have devastating effects on an already failing heart. Given that there is presumably an increased nuclear size resulting in a reduction of the nuclear surface-to-volume ratio of higher ploidy nuclei, there have been examples of a lower efficiency of nuclear import of specific factors resulting in small perturbations in gene expression (78, 79). A recent murine study demonstrated that the gene expression profiles in mono- versus multinucleated ventricular CMs are similar (80). Interestingly, a rabbit study demonstrated that LA mononuclear CMs have a more positive RMP and longer APD_90_ compared with binuclear CMs that may be associated with increased arrhythmogenic activity (81). Limitations in atrial tissue availability prohibited a thorough analysis of the effect of ploidy on human atrial gene expression in AF; however, this could be further explored in future studies.

Targeting individual AF arrhythmia mechanisms may provide a basis for the development of more effective therapies. Indeed, computational models taking into account individual fibrosis patterns have been used for personalizing ablation in a small number of patients with persistent AF with improved clinical efficacy (82). Also in support of a targeted approach, flecainide, an Na_v_1.5 blocker used to treat supraventricular tachycardias, is more effective in suppressing atrial arrhythmias in the setting of reduced *Pitx2c* mRNA levels in mice (83). Despite the promise of precision medicine approaches targeting molecular pathways, there are several barriers complicating this approach, including multifactorial AF etiologies. While it is typically thought that perpetuation of AF is associated with APD shortening and decreased atrial refractoriness (63, 67, 84, 85), there is also evidence to suggest that the electrophysiologic substrate predisposing to the onset of AF may be varied. An intriguing study involving 1308 people without structural heart disease who underwent an electrophysiologic study found that the atrial ERP (AERP) increases with age (86). Follow-up of these subjects over 12 years demonstrated that a prolonged baseline AERP, at least 280 ms, was predictive of patients at significantly increased risk of developing AF. In another study, individuals with AF associated with sick sinus syndrome also demonstrate AP prolongation (87). Therefore, AF is likely to be associated with diverse molecular and physiologic signatures. A limitation to the current study is that it only focused on human AF in the setting of HF, and it is possible that the findings may not be relevant to AF in other contexts.

While the Notch-activated murine model recapitulates transcriptomic changes associated with human AF, there are electrophysiological differences between murine and human atria. For example, the repolarizing current I_to_ is present in both mice and humans and predominates repolarization in mice, whereas I_Kr_ and I_Ks_ do not contribute significantly to murine repolarization. Downregulation of *Kcnip2*, which encodes a subunit of I_to_, is reduced in both our murine model and in human AF (64–66) and may contribute to prolongation throughout all phases of the AP observed in Notch-activated mice. To demonstrate whether Notch-mediated increases in *Kcnh2* expression would result in increased I_Kr_ and influence atrial APs, we treated Notch-activated mice with the I_Kr_ blocker dofetilide and saw no change in the APD (Figure 5, J–N; and Supplemental Table 13), suggesting that I_Kr_ is not present.

Given these differences in ionic currents in human atria when compared with animal models, there is a need for improved human model systems to better understand atrial arrhythmia mechanisms and test AF therapeutics. While the stem cell field has come a long way toward providing a valuable in vitro platform for understanding human biology and enabling high-throughput screening, it remains difficult to approximate the native atrial tissue architecture and adult atrial electrophysiologic properties (88). For example, typical conduction velocities in stem cell models are often an order of magnitude lower than velocities in human atria. Additionally, as demonstrated above, important differences exist between the human right and left atrium, and it has not been established whether atrial CMs derived from induced pluripotent stem cells behave more similarly to right or left atrial CMs. Future work will aim to establish robust platforms for elucidating underlying AF mechanisms, which may include human ex vivo models of atrial conduction to aid in the drug development process. A deeper understanding of the mechanisms underlying AF may enable development of new precision medicine strategies to reduce the high societal burden of AF.

## Methods

### Animals.

*αMHC-rtTA* (89), *tetO_NICD* (90) (iNICD) mice have been described previously and were maintained on a mixed genetic background. Mice between the ages of 2 and 9 months were used, including age-matched littermate control animals for comparison. For experiments involving conditional gene expression, induction of NICD expression was accomplished with doxycycline chow (BioServ 200 mg/kg) during the stated time points. Mice of both sexes were used in all studies, and though experiments were not powered to specifically detect sex differences, no obvious differences in any parameters were noted between sexes.

### Human tissue acquisition.

NF human hearts were obtained from Mid-America Transplant Services (MTS) in St. Louis, Missouri, USA. Experimental protocols were approved by the Washington University in St. Louis Institutional Review Board (IRB). Informed consent was obtained for all tissue used in this study. Tissue samples from the RV, LV, RA free wall, and LAPW of each heart were collected and immediately flash-frozen in liquid nitrogen and stored at −80°C. AF+HF and HF human heart tissues were obtained from the Translational Cardiovascular Biobank & Repository (TCBR) at Washington University in St Louis. Due to previously acquired patient demographics during initial tissue procurement, there is a potential for underrepresentation of clinically relevant data. The available patient characteristics of these donors are summarized in Supplemental Tables 2 and 3.

### Isolation and sorting of human and mouse CMN.

CMN isolation was modified based on previously published studies (44–46). Previously snap-frozen human samples or a combination of 2 mouse left atria were first mechanically homogenized (Bio-Gen PRO200, PRO Scientific) on ice in supplemented homogenization buffer (SHB). The crude homogenate was transferred to a 40 mL Dounce tissue grinder with glass pestle (Kimble) and underwent a minimum of 75 strokes. Progression of nuclei extraction was crudely assessed using a ×10 objective light microscope (Nikon Eclipse E200) after staining a nuclei aliquot with trypan blue. Samples were strained using a 40 μm nylon cell strainer (Corning) and centrifuged at 1000*g* (Avanti J-E Centrifuge, Beckman Coulter) for 5 minutes at 4°C. Supernatant was discarded, pellet was resuspended in SHB, and nuclei were stained with anti-PCM1 (1:1000, HPA023370, MilliporeSigma) for 30 minutes on a Nutator (Thermo Fisher Scientific) at 4°C. To wash nuclei, samples were centrifuged at 1000*g* (Centrifuge 5430R, Eppendorf) for 5 minutes at 4°C, supernatant was discarded, and pellet was resuspended in SHB. Samples were then stained with corresponding secondary antibody Alexa Fluor 647 (goat anti-rabbit, 1:1000, A21244, Invitrogen, Thermo Fisher Scientific) and DAPI (1:45,000, MilliporeSigma) for 20 minutes on a Nutator at 4°C. Nuclei were washed in SHB, then filtered using 30 μm CellTrics strainer (04-004-2326, Sysmex).

Human and mouse CMN were analyzed and sorted by FANS using a MoFlo sorter (Beckman Coulter) with a 100 μm nozzle for batch 1 or Sony SY3200 cell sorter (Sony Biotechnology) with a 100 μm nozzle for batch 2 as defined in Figure 1 and Figure 2 in collaboration with the Siteman Flow Cytometry Core at Washington University in St. Louis. For all experiments, the initial gate identified nuclei of interest and removed debris (SSC versus FSC), followed by doublet exclusion (SSC-w versus FSC), followed by autofluorescence debris exclusion (phycoerythrin versus GFP), followed by identification of CMN (DAPI versus PCM1) and final gate to identify the 2n, 4n, and >4n CMN populations. See Supplemental Figure 3 and Supplemental Figure 5 for the representative gating strategy of human and mouse CMN, respectively.

### Histology and immunohistochemistry.

Immunohistochemistry was performed on paraffin-embedded sections. Gross heart morphology and collagen content were examined using Masson’s trichrome stain (American MasterTech Scientific). Wheat germ agglutinin staining was used to visualize cell membranes and enable quantification of CM cell size.

### Microelectrode recordings.

Investigators were blinded to the sample group allocation during the experiment and analysis of experimental outcome. Mouse hearts were Langendorff perfused and were recorded while in sinus rhythm and when stimulated at 10 Hz (approximately 600 beats per minute). Using glass sharp microelectrodes, single LA CMs were sampled near the epicardial surface. To decrease noise from motion artifacts, blebbistatin (0.2 mg/mL) was used to arrest motion and allow for stable microelectrode recording without requiring the use of floating electrodes.

### Additional information.

All methods related to mouse RT-qPCR (Supplemental Table 14) and RNA-sequencing and analysis are detailed in the Supplemental Methods. Expanded methods for human tissue acquisition, CMN isolation, histology and immunohistochemistry, and microelectrode recordings are also supplied in the Supplemental Methods.

### RA RNA-sequencing accession number.

RA RNA-sequencing data discussed in this manuscript have been deposited in the National Center for Biotechnology Information’s (NCBI) Gene Expression Omnibus (GEO) database and are accessible through GSE100244.

### LA RNA-sequencing accession number.

LA RNA-sequencing data discussed in this manuscript have been deposited in NCBI’s GEO and are accessible through GSE138253.

### Human RNA-sequencing accession number.

Data have been deposited in NCBI’s GEO and are accessible through GSE138252.

### Statistics.

All data are expressed as mean ± SEM. Statistical analyses were performed after assessing for normal distribution using either paired or unpaired Student’s 2-tailed *t* tests for comparison of 2 groups with a Welch’s correction. Values of *P* < 0.05 were considered statistically significant.

### Study approval.

Animal protocols were approved by the Animal Studies Committee at Washington University in St. Louis, and animals were handled in accordance with the NIH’s *Guide for the Care and Use of Laboratory Animals* (National Academies Press, 2011). Protocols involving human tissue acquisition were approved by the Washington University in St. Louis IRB. Informed consent was obtained for all tissue before inclusion in this study. Methods described in this manuscript were performed in accordance with all human research guidelines.

## Author contributions

SLR was responsible for conceptualization of the study. SLR, CEL, JJ, and QG contributed to experimental design. CEL, JJ, QG, TY, and SB conducted experiments, acquired data, and performed data analysis. SCH conducted histology staining. GL contributed to data analysis. CEL, JJ, QG, GL, TY, SCH, DMZ, UG, KT, and BDB contributed to human tissue acquisition. RDN and CPC performed mouse RNA-sequencing and statistical analysis. SL and BZ performed mouse and human RNA-sequencing statistical analyses. CEL and JJ wrote the original draft of the manuscript. SLR and IPM edited the manuscript. First authorship order was determined by the number of figures contributed to the final manuscript.

## Supplementary Material

Supplemental data

## Version 1 08/25/2020

In-Press Preview

## Version 2 09/17/2020

Electronic publication
