# Supplementary material for: Chamber-specific transcriptional responses in atrial fibrillation
Source: JCI Insight. 2020 Sep 17;5(18):e135319. doi: 10.1172/jci.insight.135319 (PMC7526559; doi:10.1172/jci.insight.135319)
Supplement: Supplemental data [file jciinsight-5-135319-s070.pdf]

Supplemental Figure 1

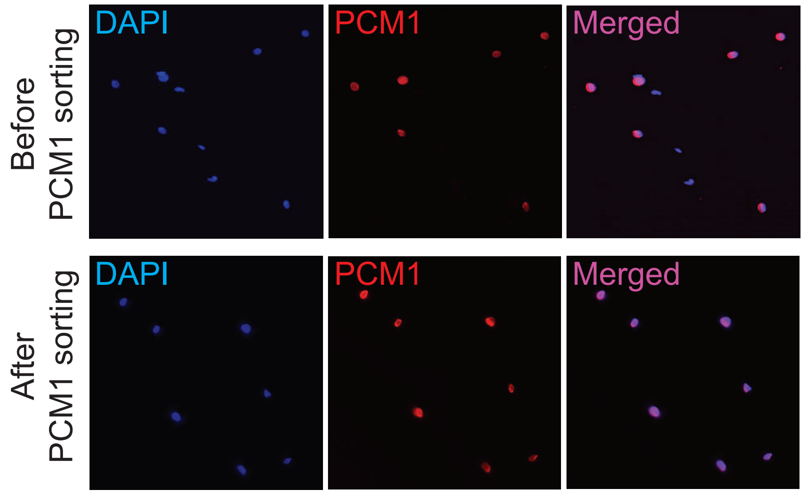

Supplemental Figure 1. Human cardiomyocyte nuclei are enriched after PCM1 sorting. Representative images of crude nuclei pellets before (top) and after (bottom) PCM1-based sorting show that sorting for PCM1 isolates a population highly enriched for cardiomyocyte nuclei.

Supplemental Figure 2

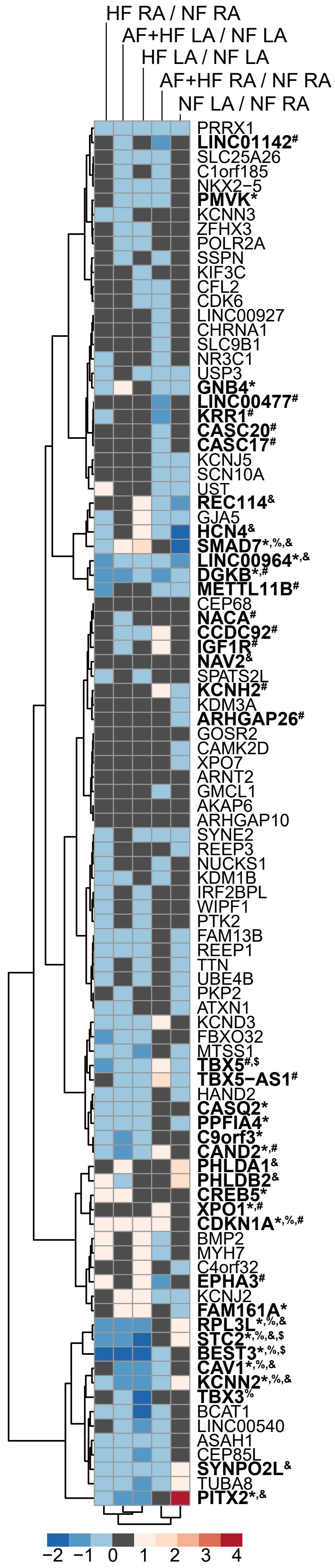

Supplemental Figure 2. Gene expression heatmap of atrial fibrillation-associated GWAS genes in human atrial cardiomyocyte nuclei. Heatmap showing the genes indicated in atrial fibrillation (AF) genome-wide association studies (GWAS) and their relative expression (red = up, blue = down) in cardiomyocyte nuclei (CMN). Color scale indicates the relative expression level (z-score). RNA sequencing performed from two separate sample batches: Batch 1) n = 9 individual RA (n = 6 NF, n = 3 HF) and n = 10 individual LA (n = 7 NF, n = 3 HF). Batch 2) n = 8 individual RA (n = 3 NF, n = 5 AF+HF) and n = 10 individual LA (n = 5 NF, n = 5 AF+HF). Genes with a fold change threshold of ≤ 0.667 or ≥ 1.5 and FDR < 0.05 were considered statistically significant. Comparisons for significantly changed versus NF counterparts are as follows: * = AF+HF LA; % = HF LA; & = NF LA; # = AF+HF RA; $ = HF RA. See corresponding **Supplemental Table S9** for fold change values.

Supplemental Figure 3

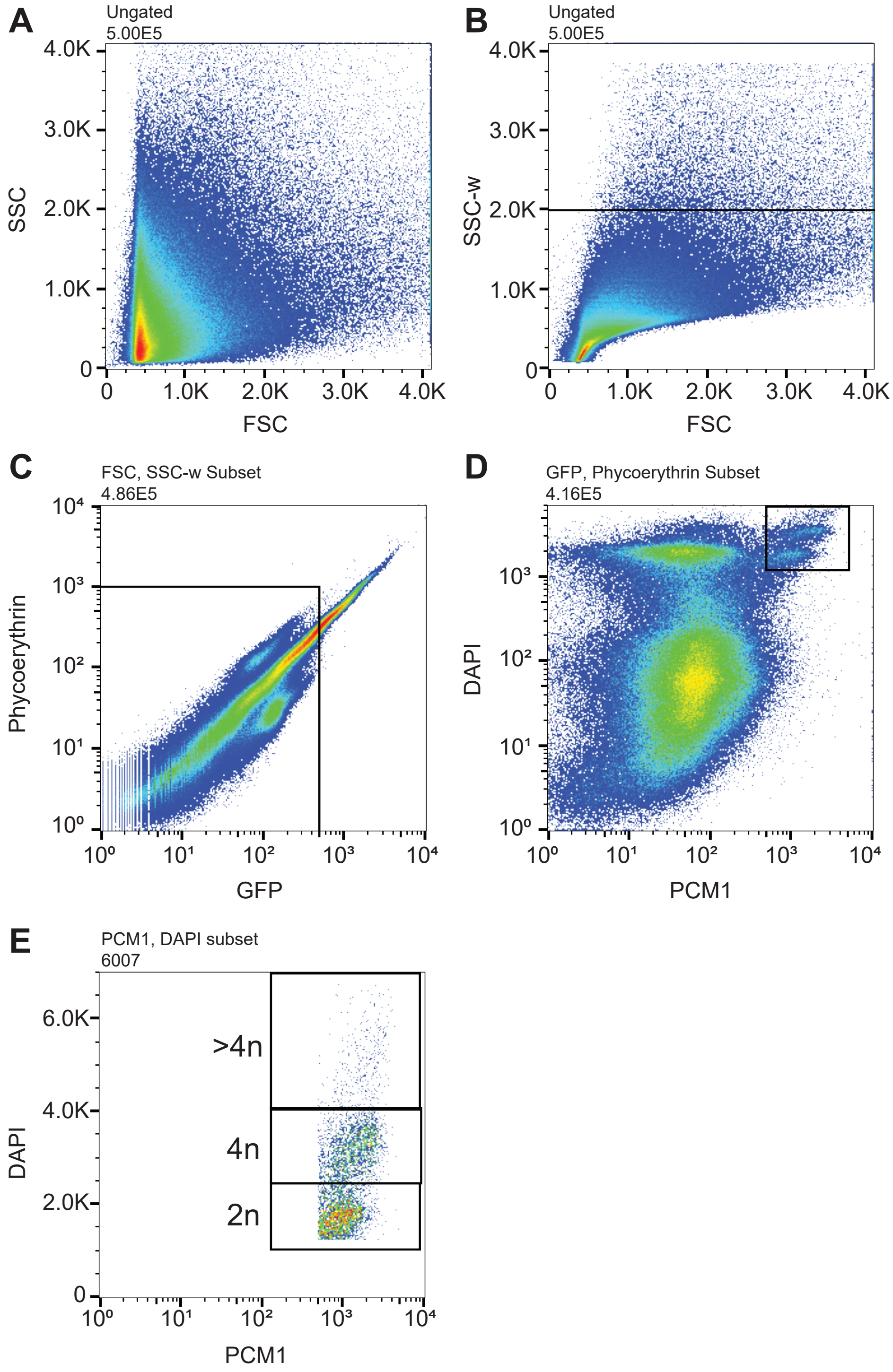

Supplemental Figure 3. Flow cytometry gating strategy to isolate human cardiomyocyte ploidy sub-populations. (**A**) Identification of nuclei of interest and debris removal (SSC versus FSC). (**B**) Doublet exclusion (SSC-w versus FSC). (**C**) Autofluorescence debris exclusion (Phycoerythrin versus GFP). (**D**) Identification of double-positive cardiomyocyte nuclei (CMN, DAPI versus PCM1). (**E**) Final gate to identify the different ploidy populations of CMN, separated into 3 sub-populations based on DAPI intensity: 2n, 4n, >4n.

Supplemental Figure 4

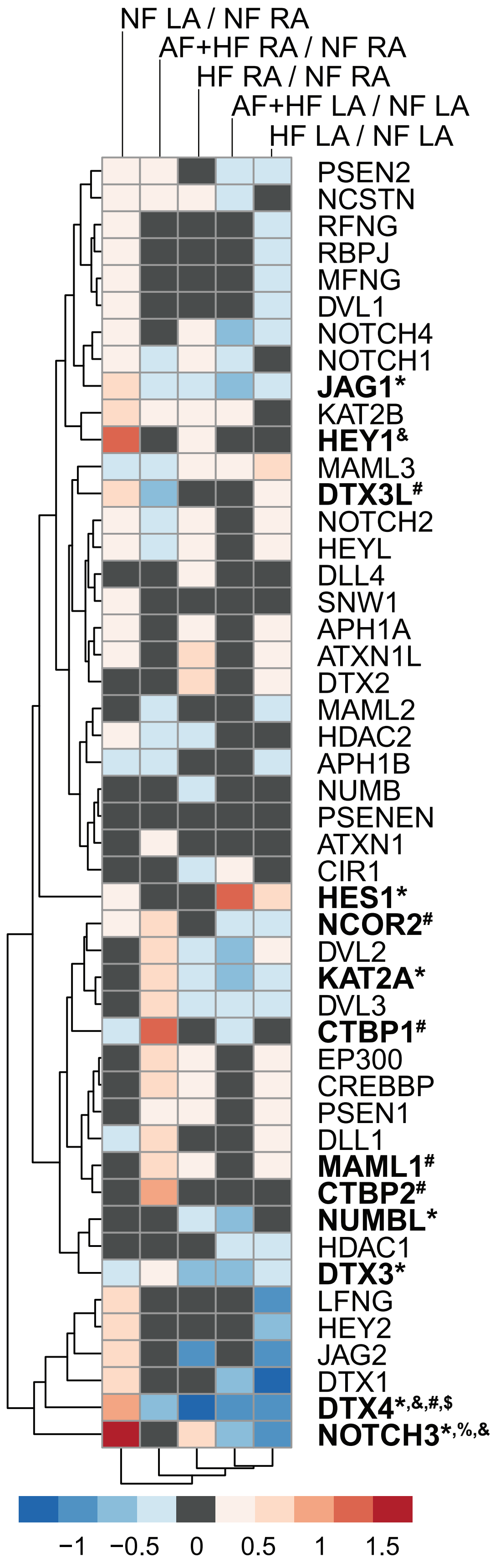

Supplemental Figure 4. Notch pathway expression heatmap in human atrial cardiomyocyte nuclei. The Kyoto Encyclopedia of Genes and Genomes (KEGG) database was used to create a list of Notch signaling pathway related genes. These genes were interrogated for their relative expression levels in human atrial cardiomyocyte nuclei (CMN). Heatmap of the relative expression levels for right (NF RA) and left atria (NF LA) CMN from non-failing hearts and hearts with AF in the setting of heart failure (AF+HF RA, AF+HF LA) or heart failure alone (HF RA, HF LA). Color scale indicates the relative expression level (z-score). RNA sequencing performed from two separate sample batches: Batch 1) *n* = 9 individual RA (*n* = 6 NF, *n* = 3 HF) and *n* = 10 individual LA (*n* = 7 NF, *n* = 3 HF). Batch 2) *n* = 8 individual RA (*n* = 3 NF, *n* = 5 AF+HF) and *n* = 10 individual LA (*n* = 5 NF, *n* = 5 AF+HF). Genes with a fold change threshold of ≤ 0.667 or ≥ 1.5 and FDR < 0.05 were considered statistically significant. Comparisons for significantly changed versus NF counterparts are as follows: * = AF+HF LA; % = HF LA; & = NF LA; # = AF+HF RA; $ = HF RA.

Supplemental Figure 5

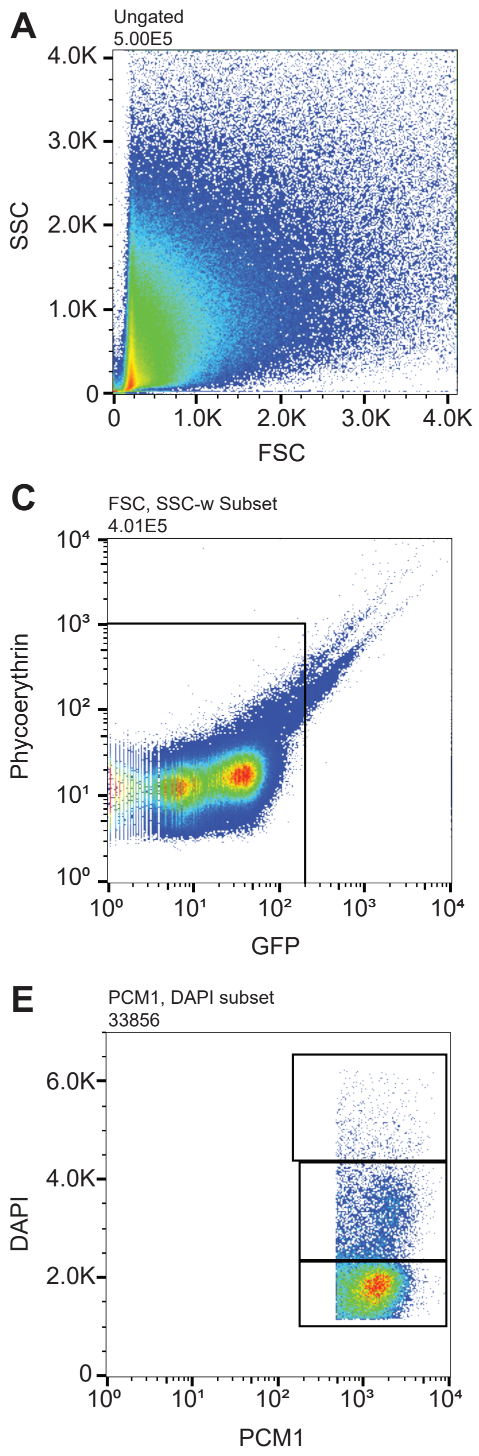

Supplemental Figure 5. Flow cytometry gating strategy to isolate murine cardiomyocyte ploidy sub-populations. (**A**) Identification of nuclei of interest and debris removal (SSC versus FSC). (**B**) Doublet exclusion (SSC-w versus FSC). (**C**) Autofluorescence debris exclusion (Phycoerythrin versus GFP). (**D**) Identification of double-positive cardiomyocyte nuclei (CMN, DAPI versus PCM1). (**E**) Final gate to identify the different ploidy populations of CMN, separated into 3 sub-populations based on DAPI intensity: 2n, 4n, >4n.

Supplemental Figure 6

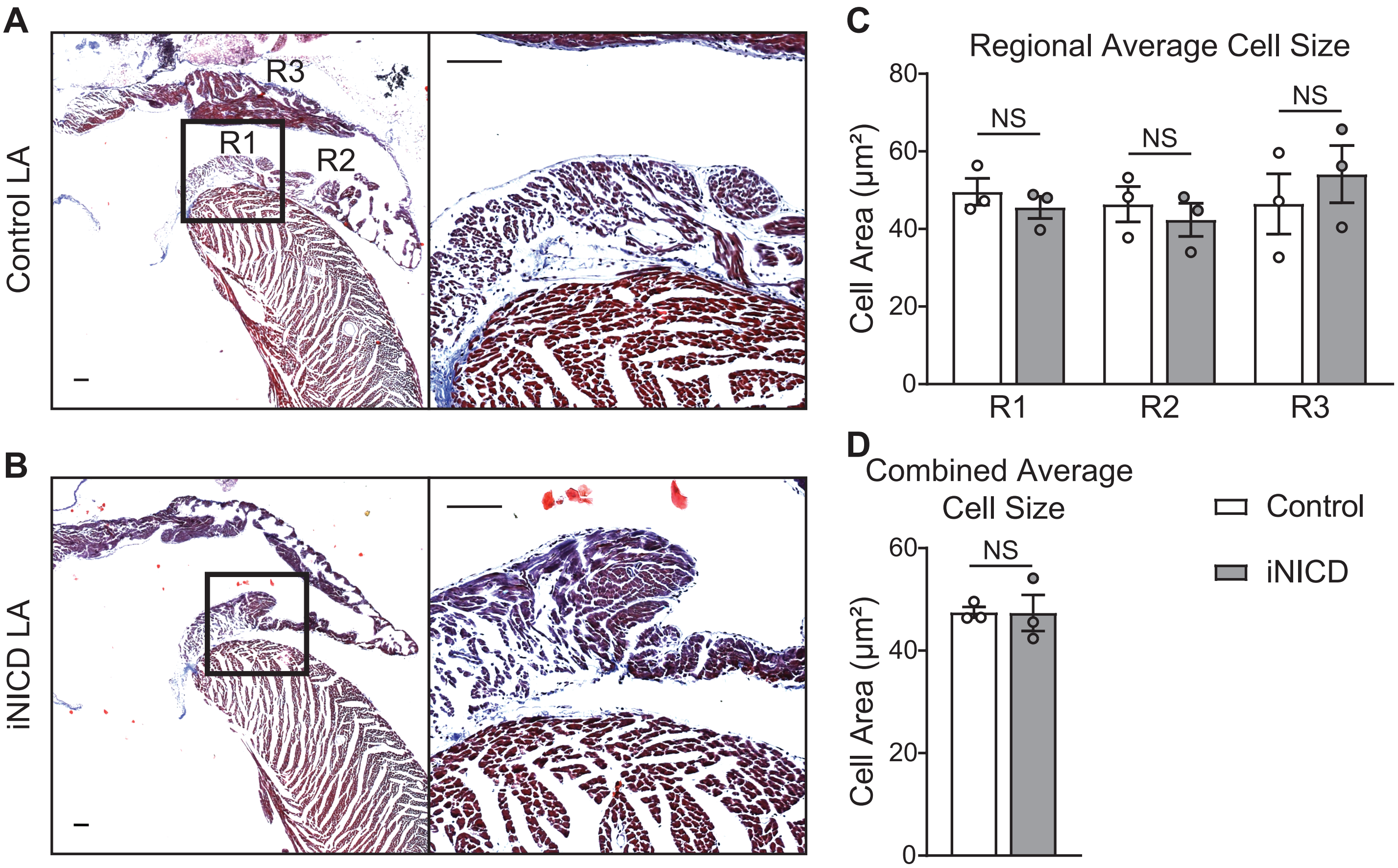

Supplemental Figure 6. Notch signaling does not alter left atrial structure or left atrial cardiomyocyte size. Histological sections from *αMHC-rtTA* littermate controls (n = 3) and iNICD *αMHC-rtTA*; *tetO_NICD* (n = 3) mice were labeled with Masson's trichrome stain to reveal the overall structure of the iNICD left atrium (LA). When mice were 2-4 months old, doxycycline chow was administered for 3-4 weeks. Representative Trichrome images of control (**A**) and iNICD (**B**) LA. The black box in the left panels is the region shown at higher magnification in the corresponding right panels. Scale bars represent 100 μm. WGA stain was performed on serial sections to visualize cell borders. Quantification of the average cardiomyocyte cell area was performed within three distinct LA regions (R1, R2, R3) as indicated by the areas shown on the representative Trichrome stained image in (**A**). The average cardiomyocyte cell area within each region (**C**) as well as the combined average cardiomyocyte cell area for all three regions (**D**) shows a comparable cardiomyocyte cell area in iNICD mice and littermate controls. Statistics were performed using unpaired t tests with Welch's correction. Values of $P < 0.05$ were considered statistically significant.

Supplemental Figure 7

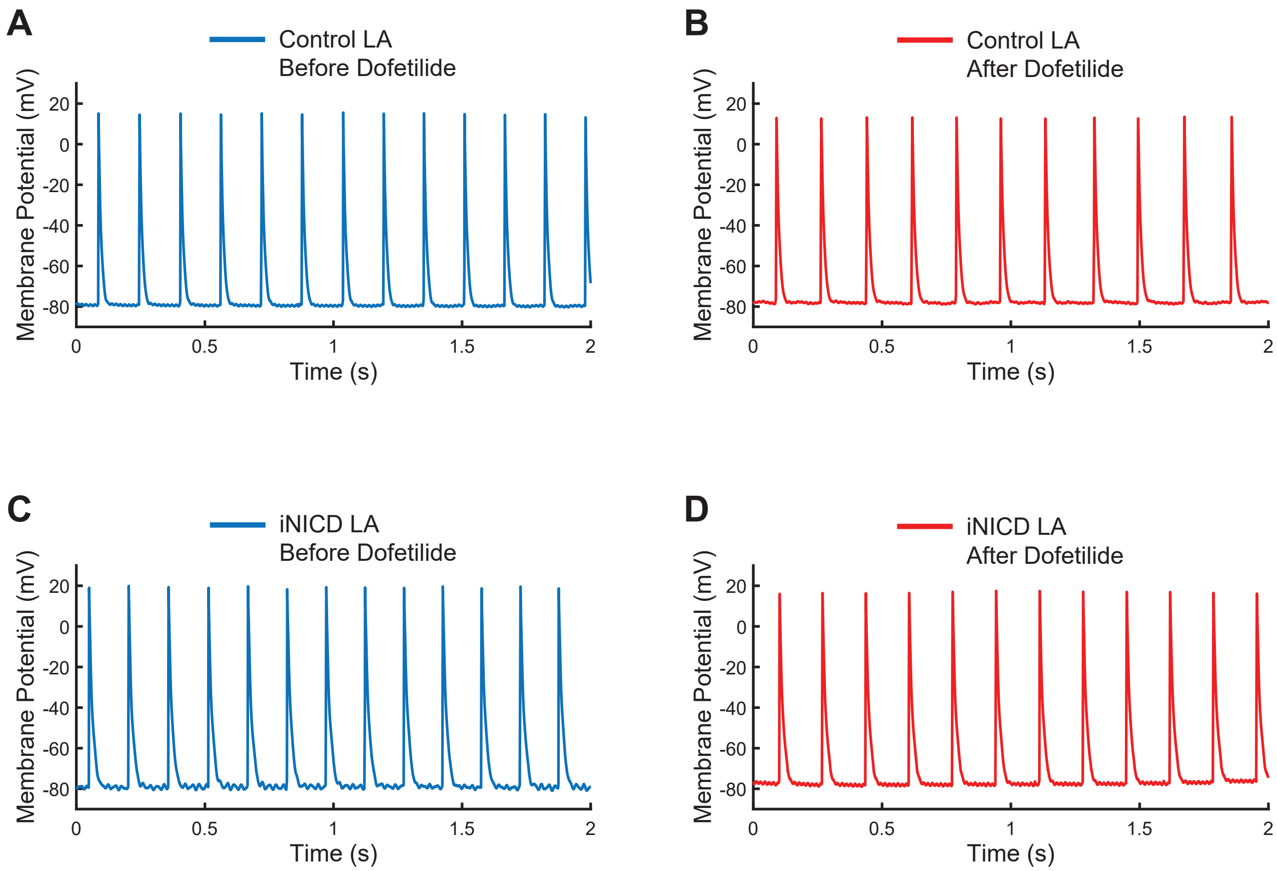

Supplemental Figure 7. Intracellular left atrial action potential traces. Representative action potential traces from sharp microelectrode recordings were obtained from the left atrium (LA) cardiomyocytes of intact, Langendorff-perfused murine hearts that were administered doxycycline chow for 4 weeks without a washout. Representative action potential traces are shown for Control LA before (**A**) and after (**B**) 10 nM dofetilide administration and iNICD LA before (**C**) and after (**D**) 10 nM dofetilide administration. Serial action potentials ($n \geq 10$) were recorded from a minimum of 4 cardiomyocytes per biological replicate in each group.

Supplemental Figure 8

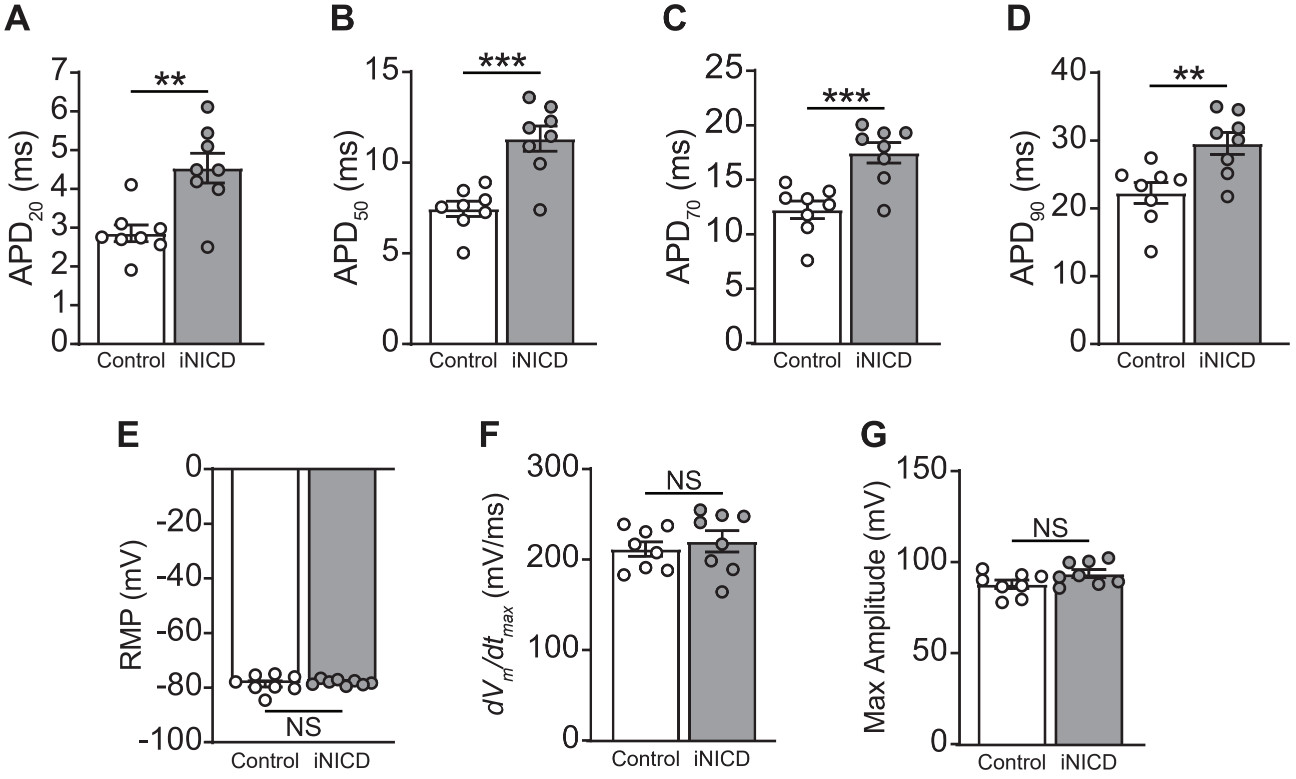

Supplemental Figure 8. Action potential duration is prolonged in iNICD LA during 10 Hz pacing. Sharp microelectrode recordings with 10 Hz pacing were performed ex vivo on the left atrium (LA) of intact hearts in control (*αMHC-rtTA*, *n* = 8) and iNICD (*αMHC-rtTA*; *tetO_NICD*, *n* = 8) mice that were administered doxycycline chow for 4 weeks followed by a minimum 4-month washout. Action potential duration (APD) at 20% (**A**), 50% (**B**), 70% (**C**), and 90% (**D**) repolarization are all significantly prolonged. Resting membrane potential (RMP, **E**), dV_m/dt_{max} (**F**), and max amplitude (**G**) are not significantly changed. Data are presented as the mean from each mouse ± SEM. $P < 0.05$ was considered statistically significant. **$P < 0.01$, ***$P < 0.001$, ****$P < 0.0001$, NS = not significant. Unpaired Student's *t* test with a Welch's correction was performed for all comparisons.

Supplemental Figure 9

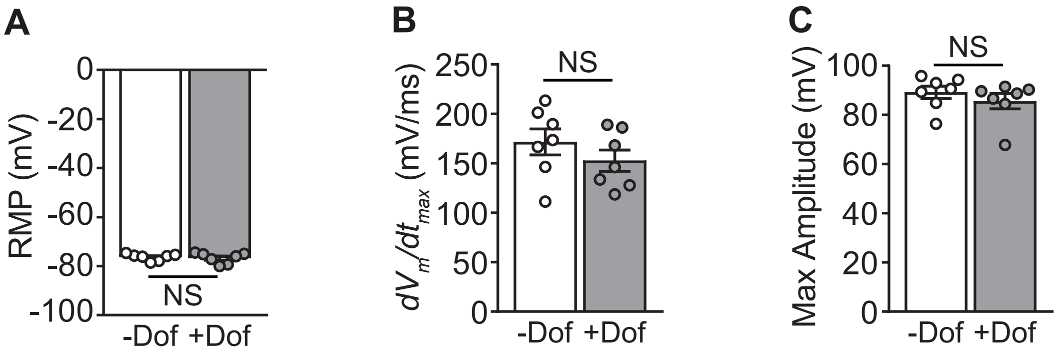

Supplemental Figure 9. Murine action potential characteristics after exposure to the I_{Kr} blocker dofetilide. Sharp microelectrode recordings were performed ex vivo on the left atrium (LA) of intact murine iNICD hearts that were administered doxycycline chow for 4 weeks without washout. Recordings were measured on hearts in sinus rhythm at baseline (-Dof), and after exposure to 10 nm dofetilide (+Dof), a Class III antiarrhythmic drug that blocks I_{Kr}. (**A-C**) Action potential characteristics including resting membrane potential (RMP, **A**), dV_m/dt_{max} (**B**), and max amplitude (**C**) are not changed as a result of dofetilide administration. *n* = 7 iNICD mice. Data are presented as the mean from each mouse ± SEM. $P < 0.05$ was considered statistically significant. NS = not significant. Paired Student's *t* test with a Welch's correction was performed for all comparisons.

Supplemental Methods

Mouse Reverse Transcription-Quantitative Polymerase Chain Reaction

Total RNA was isolated from mouse left atria (LA) using TRIzol (Invitrogen) and DNase treated using TURBO DNA-*free* DNase Treatment Kit (Ambion). First-strand cDNA was synthesized using a high Capacity cDNA Reverse Transcription kit (Applied Biosystems). Gene expression was assayed using the Power SYBR Green PCR Master Mix (Applied Biosystems) with primers listed below and quantified using the QuantStudio 6 Flex Real-Time PCR System (Applied Biosystems). Relative fold changes were calculated using the comparative threshold cycle methods (2-ΔΔCt). Primer sequences are listed in **Supplemental Table S14** of the online-only Data Supplement.

Mouse RNA Sequencing

For RNA sequencing, littermate control (*αMHC-rtTA*, n = 4 female, n = 2 male) and iNICD (*αMHC-rtTA; tetO_NICD*, n = 3 female, n = 3 male) mice at 2-3 months of age were administered doxycycline (dox) chow for 3 weeks with no washout prior to tissue harvest. Each LA was sequenced independently (n = 6 samples per condition). Right atria (RA) and LA were obtained from the same animals. Illumina libraries were constructed either by the Genome Technology Access Center (GTAC) at Washington University School of Medicine (RA) or the Moskowitz lab at University of Chicago (LA).

For the RA, after ribosomal RNA depletion, reverse-transcription with random-priming, library construction and multiplexing, samples were sequenced on an Illumina HiSeq 3000 using indexed libraries multiplexed at 6 samples per lane. Single-end 50-base sequencing reads with a total target depth of >25 million reads were performed. RNA-seq reads were aligned to the Ensembl release 76 assembly with STAR version 2.0.4b. Gene counts were derived from the number of uniquely aligned unambiguous reads by Subread:featureCount version 1.4.5. Transcript counts were produced by Sailfish version 0.6.3. Sequencing performance was assessed for total number of aligned reads, total number of uniquely aligned reads, genes and transcripts detected, ribosomal fraction known junction saturation and read distribution over known gene models with RSeQC version 2.3.

All gene-level and transcript counts were then imported into the R/Bioconductor package EdgeR and TMM normalization size factors were calculated to adjust for samples for differences in library size. Genes or transcripts not expressed in any sample were excluded from further analysis. The TMM size factors and the matrix of counts were then imported into R/Bioconductor package Limma and weighted likelihoods based on the observed mean-variance relationship of every gene/transcript were then calculated for all samples with the Voom function. Performance of the samples was assessed with a spearman correlation matrix and multi-dimensional scaling plots. Gene/transcript performance was assessed with plots of residual standard deviation of every gene to their average log-count with a robustly fitted trend line of the residuals. Generalized linear models with robust dispersion estimates were then created to test for gene/transcript level differential expression. Differentially expressed genes and transcripts were then filtered for FDR adjusted P-values less than 0.05.

RA RNA-Sequencing Accession number: RA RNA-sequencing data discussed in this manuscript have been deposited in NCBI's Gene Expression Omnibus and are accessible through GEO Series accession # GSE100244 (https://www.ncbi.nlm.nih.gov/geo/query/acc.cgi?acc=GSE100244).

For the LA, libraries were prepared from LA RNA starting with 1 µg per sample and using the mRNA-seq Sample Prep Kit (Illumina) as per recommended instructions. After Ribozero purification and removing only ribosomal RNA, barcoded libraries were prepared according to Illumina's instructions (2013) accompanying the TruSeq RNA Sample prep kit v2 (Part# RS-122-2001). Libraries were quantitated using the Agilent Bio-analyzer (model 2100) and pooled in equimolar amounts. The pooled libraries were sequenced with stranded 50-bp single-end reads on the HiSeq2500 in Rapid Run Mode following the manufacturer's protocols (2013). 22M to 30M reads were mapped to mouse genome with TopHat2 (v 2.1.1). Reads mapped to the mitochondrial genome, and with phred score < 30 were excluded. Counts aligned to exons were retrieved with HTseq (v.0.6.0) in union mode. Lastly, counts were analyzed for differential expression with R (3.4) package DEseq2.

To create a Venn diagram of the dysregulated transcripts from murine RA and LA when they were sequenced at two different facilities, RNA-seq reads were aligned to the mouse genome mm10 with STAR version 2.5.4b [23104886]. Gene counts were derived from the number of uniquely aligned unambiguous reads by Subread:feature, version 1.4.6 [23558742], with gene-body annotation of mouse GENCODE M15 [22955987]. The gene-level expression of transcripts with CPM >1.0 were further imported into R package EdgeR, and exactTest function were applied to detect gene expression differences. False discovery rate (FDR) for all statistical tests were determined by EdgeR using the Benjamini and Hochberg correction, and an FDR < 0.05 between normal samples and disease samples were used for analyses.

To enhance the biological interpretation of the large set of transcripts, grouping of genes/transcripts based on functional analysis was performed on differentially expressed genes using Ingenuity Pathway Analysis (IPA) (http://www.ingenuity.com). A total of 40 disease categories were statistically significant using an FDR < 0.05, and IPA was used to generate pathway maps. To identify important signaling pathways within our dataset, differentially expressed genes were classified into biological categories by COMPBIO (https://percayai.com), which performs a literature analysis to identify relevant biological processes and pathways represented by the differentially expressed entities (genes, proteins, miRNA's, or metabolites).

LA RNA-Sequencing Accession number: **LA RNA-sequencing data discussed in this manuscript have been deposited in NCBI's Gene Expression Omnibus and are accessible through GEO** Series accession # GSE138253 (https://www.ncbi.nlm.nih.gov/geo/query/acc.cgi?acc=GSE138253).

Lists of genes differentially regulated in the RA and LA of mouse and human tissue as previously published (1).

Human Tissue Acquisition

Non-failing human hearts were obtained from Mid-America Transplant Services (MTS) in St. Louis, MO. Experimental protocols were approved by the Washington University in St. Louis Institutional Review Board (IRB). Informed consent was obtained for all tissue used in this study. Methods described in this section were performed in accordance with all human research guidelines. Prior to organ harvest, intact hearts were perfused and arrested with cold cardioplegic solution (Belzer UW® Cold Storage Solution, Bridge to Life Ltd., Columbia, SC). After procurement, hearts were immediately submerged in cold cardioplegia and transported on ice. Tissue samples from the right ventricle, left ventricle, RA free wall, and LA posterior wall of each heart were collected and immediately flash-frozen in liquid nitrogen and stored at −80°C. AF+HF human heart tissues were obtained from Tissue Translational Cardiovascular Biobank & Repository (TCBR) at Washington University in St Louis. The patient characteristics of these donors are summarized in **Supplemental Tables S2**, **S3**.

Isolation of Human and Mouse Cardiomyocyte Nuclei

Cardiomyocyte nuclei isolation was modified based on previously published (2-4). Previously snap-frozen human samples weighing ~150-250 mg or a combination of two mouse left atria weighing ~9-16 mg (total) were first mechanically homogenized (Bio-Gen PRO200, PRO Scientific) at ¾ strength for 15-20 s on ice in supplemented homogenization buffer (SHB) containing: 250 mM sucrose (Sigma-Aldrich), 25 mM KCl (Sigma-Aldrich), 5 mM $MgCl_2$ (Sigma-Aldrich), 20 mM Tricine-KOH (Sigma-Aldrich), 15 nM spermine (Sigma-Aldrich), 50 nM spermidine (Sigma-Aldrich), 1 mM Dithiothreitol (Sigma-Aldrich), 0.2% IGEPAL CA-630 (Sigma-Aldrich), 80 U/ml RNasin (Promega #N2611). The crude homogenate was transferred to a 40 mL (for human) or 15 mL (for mouse) Dounce tissue grinder with glass pestle (Kimble) and underwent a minimum of 75 strokes. Progression of nuclei extraction was crudely assessed using a 10x objective light microscope (Nikon Eclipse E200) after staining a nuclei aliquot with Trypan blue. Samples were strained using a 40 µm nylon cell strainer (Corning) and centrifuged at 1,000 g (Avanti J-E Centrifuge, Beckman Coulter) for 5 min at 4°C. Supernatant was discarded, pellet was resuspended in SHB, and nuclei were stained with anti-pericentriolar material 1 (PCM-1, 1:1000, HPA023370, Sigma-Aldrich) for 30 min on a Nutator (Fisher Scientific) at 4°C. To wash nuclei, samples were centrifuged at 1,000 g (Centrifuge 5430R, Eppendorf) for 5 min at 4°C, supernatant was discarded, and pellet was resuspended in SHB. Samples were then stained with corresponding secondary antibody Alexa Fluor 647 (goat anti-rabbit, 1:1000, A21244, Invitrogen) and 4′,6-Diamidino-2-phenylindole (DAPI, 1:45,000, Sigma) for 20 min on a Nutator at 4°C. Nuclei were washed in SHB then filtered using 30 µm CellTrics strainer (04-004-2326, Sysmex).

Human RNA Sequencing

RNA was extracted from $PCM1^+$ cardiomyocyte nuclei using Zymo Direct-zol RNA MicroPrep kit (Cat# 11-330M) and submitted to GTAC at Washington University School

of Medicine (St. Louis, MO) for RNA sequencing. Indexed RNA libraries were prepared from 10 ng of RNA from each sample with the Sigma Seqplex RNA amplification Kit (Sigma, Cat# SEQR). cDNA libraries were PCR-amplified in the presence of indexing adapters and pooled for RNA-sequencing. To minimize batch effects, library preparations for samples from each individual heart were performed simultaneously. Single-end 1 × 50 sequencing was performed on an Illumina HiSeq 2500 sequencer.

Accession number: Data have been deposited in the NCBI GEO database for release after publication and are accessible through GEO Series accession #138252. The following link and security token have been created to allow review: https://www.ncbi.nlm.nih.gov/geo/query/acc.cgi?acc=GSE138252 (security token: gbireuaahrurtil)

Human RNA Sequencing Analysis

Nuclear-RNA-seq reads were aligned to the human genome hg38 with STAR version 2.5.4b [23104886]. Gene counts were derived from the number of uniquely aligned unambiguous reads by Subread:feature, version 1.4.6 [23558742], with gene-body annotation of human GENCODE V27 [22955987]. The genes located on sex chromosomes (chrX/Y) were removed to avoid sex-bias. All gene-level transcript counts were then imported into the R/Bioconductor package RUVseq [25150836] to remove unwanted batch effect variation after upper-quantile normalization. The gene-level expression of transcripts with CPM >1.0 were further imported into R package EdgeR [19910308], and exactTest function was applied to detect gene expression differences. False discovery rate (FDR) for all statistical tests were determined by EdgeR using the Benjamini and Hochberg correction, and genes with a 1.5-fold expression change between normal samples and disease samples with FDR < 0.05 were considered differentially expressed genes (DEGs). Principal Component Analysis was performed using plotPCA also from the DESeq2 [25516281] package and plotted using ggplot2 package. The human TFs list was obtained from AnimalTFDB [22080564]. TFs with a 1.5-fold expression change between normal samples and disease samples with FDR < 0.05 were considered differentially expressed TFs. Expression of TFs (RPKM) were z-score scaled and hierarchical clustered using R package pheatmap.

Histology and Immunohistochemistry

Immunohistochemistry was performed on paraffin-embedded sections. Gross heart morphology and collagen content were examined using Masson's Trichrome stain (American MasterTech Scientific, REF: KTMTRPT). Histology was analyzed using Adobe Photoshop. Control and mutant images were treated identically in all cases where brightness and contrast were altered. To compare the left atrial cardiomyocyte size between genotypes, wheat germ agglutinin (WGA) staining was performed on 3 *αMHC-rtTA* controls (*n* = 1 female, *n* = 2 male) and 3 littermate *αMHC-rtTA; tetO_NICD* iNICD (*n* = 2 female, *n* = 1 male) paraffin-embedded sections. Mice were fed dox chow for 2-4 weeks beginning when the mice were between 2-4 months old. No washout period was given. The area of approximately 100 cells perpendicular to the plane of sectioning was outlined and quantified using Axiovision. The area of only circular cardiomyocytes was included in the analysis, while elongated cells were excluded, to minimize bias in the analysis from cells not perpendicular to the plane of sectioning. Quantification was

performed in 3 distinct regions of the LA from *n* = 3 hearts of each genotype by an observer blinded to genotype, and data from all 3 regions were also pooled for comparison. Statistical comparison was performed using an unpaired Student's *t* test with a Welch's correction.

Microelectrode Recordings

Investigators were blinded to the sample group allocation during the experiment and analysis of experimental outcome. Mouse hearts were Langendorff perfused and were recorded while in sinus rhythm and when stimulated at 10 Hz (approximately 600 beats per minute). Using glass sharp microelectrodes, single LA cardiomyocytes were sampled near the epicardial surface. Thin glass pipettes with high resistance (10-20 MΩ) filled with 3 M KCl were used to record action potentials from a minimum of 4 single cells from each region of the heart (technical replicates) in the intact perfused heart. To decrease noise from motion artifacts, blebbistatin (0.2 mg/mL) was used to arrest motion and allow for stable microelectrode recording without requiring the use of floating electrodes. Electrical signals were digitized by a Power 1401 A/D converter. Experiments were performed and recordings were analyzed by an observer blinded to genotype. Sampled cells with a resting membrane potential (RMP) less than –70mV were averaged. The RMP, action potential amplitude (APA), maximum upstroke velocity (dV_m/dt_{max}), and action potential duration (APD) at 20, 50, 70, and 90% repolarization are reported. Analysis of microelectrode recordings was performed using a custom MATLAB script. Cells with at least 4 consecutive action potentials and stable RMP were included in the data analysis. For each action potential in each cell, the peak potential value and time were calculated. The RMP was then calculated for each action potential using a histogram, where the maximum frequency value was defined as the RMP. The magnitude (amplitude) of each action potential was then defined as the difference between peak potential value and the RMP. The maximum change in voltage with respect to time (dV_m/dt_{max}) was calculated using a first derivative. The point in time that corresponds to this value was then used as the start time for APD calculation. The end time was when the potential decreased to 90% of the amplitude value.

For LA microelectrode recordings examining the effect of cardiomyocyte-specific Notch activation on LA electrophysiology, mice were 3 months old at the onset of dox chow administration. Dox was administered for 4 weeks, followed by a washout of between 4-5 months. Controls were *αMHC-rtTA* on dox (*n* = 4 females, *n* = 5 males) for sinus rhythm recordings, a subset of which we were also able to obtain recordings at 10Hz pacing (*n* = 4 females, *n* = 4 males). Experimental iNICD mice were *αMHC-rtTA; tetO_NICD* (*n* = 5 females, *n* = 5 males) for sinus rhythm recordings, a subset of which we were also able to obtain recordings at 10Hz pacing (*n* = 3 females, *n* = 5 males). To examine the effect of dofetilide, mice were 2 months old at the onset of dox chow administration. Dox was administered for 4-6 weeks and recordings were performed without a washout period. Controls were *αMHC-rtTA* on dox (*n* = 5 females, *n* = 2 males) for sinus rhythm recordings. Experimental iNICD mice were *αMHC-rtTA; tetO_NICD* (*n* = 2 females, *n* = 5 males) for sinus rhythm recordings. After baseline recordings were obtained, dofetilide was administered directly into the circulating Tyrodes solution at a final concentration of

10 nM. The dofetilide was allowed to circulate for 30 minutes before action potential recordings were measured.

References

1. Heberle H, Meirelles GV, da Silva FR, Telles GP, and Minghim R. InteractiVenn: a web-based tool for the analysis of sets through Venn diagrams. *BMC Bioinformatics.* 2015;16:169.
2. Bergmann O, and Jovinge S. Isolation of cardiomyocyte nuclei from post-mortem tissue. *J Vis Exp.* 2012(65):pii: 4205.
3. Krishnaswami SR, Grindberg RV, Novotny M, Venepally P, Lacar B, Bhutani K, et al. Using single nuclei for RNA-seq to capture the transcriptome of postmortem neurons. *Nature Protocols.* 2016;11:499-524.
4. Preissl S, Schwaderer M, Raulf A, Hesse M, Gruning BA, Kobele C, et al. Deciphering the Epigenetic Code of Cardiac Myocyte Transcription. *Circulation research.* 2015;117:413-23.

Table S1. Validation of cardiomyocyte nuclei using RNA-sequencing from different cardiac chambers.

Transcripts per Kilobase Million				LV-1	LV-2	RV	LA	RA	RA*
PCM1+ RNA-Sequencing	Atrial Specific		*NPPA*	355	235	40	40364	48793	39623
			NPPB	5	30	2	194	159	885
			SLN	6	6	8	536	2253	2041
		Transcription Factors	*PITX2*	1	0.3	0.1	26	0.0	0.2
			TBX5	59	87	46	273	374	369
			NR2F1	0.1	0.2	0.2	12	22	28
			NR2F2	15	20	23	99	116	123
		Ion Channels	*GJA5*	10	11	22	51	74	133
			KCNA5	1	2	2	33	45	61
			KCNJ3	1	1	1	134	151	105
	Ventricular Specific		*KCNJ2*	1954	1679	1284	131	167	42
		Structural Genes	*MYL2*	16891	10120	19103	416	34	18
			MYL3	3233	2749	3471	44	49	71
			HEY2	41	58	33	3	3	1
	Non-Cardiomyocytes		*PDGFRA*	15	16	52	37	33	30
			COL1A1	7	12	14	45	10	73
			TCF21	0.3	2	4	3	3	3
			CD90	Undetected					
			CD31	Undetected					

Transcripts from a pilot cohort of human non-failing cardiomyocyte nuclei (CMN), expressed as transcripts per kilobase million, to determine whether RNA-sequencing on RNA extracted from our CMN protocol has the resolution to detect changes in chamber-specific transcripts. CMN were extracted from the left ventricle (LV), right ventricle (RV), left atrium (LA), and right atrium (RA) of the same biological sample (74-year-old female that died from a stroke with a history of hypertension and smoking). LV-1 and LV-2 were

two different pieces of tissue from the LV of the same biological sample to determine whether there is similar transcript expression between technical replicates. RA* CMN extracted from a separate biological sample (46-year-old female that died from anoxia with a history of hypertension, diabetes, and nonsmoker) to determine whether there is similar CMN transcript expression between biological replicates. $n = 1$ for LV-1, LV-2, RV, LA, RA, RA*.

Table S2. Summary of the clinical information of non-failing heart donors included in the present study

						Comorbidities		
		Sample ID	Sex	Age (yr)	Cause of Death	HTN	DM	Smoker
Control (HF)	Right Atrium	1*	F	74	Stroke	Y	N	Y
		2*	M	56	Anoxia	N	N	Y
		3*	M	63	Anoxia	Y	N	Y
		4*	M	57	Stroke	Y	N	N
		5*	M	34	Anoxia	N	N	Y
		6*	F	34	Anoxia	N	N	N
	Left Atrium	7*	F	74	Stroke	Y	N	Y
		8*	M	64	Stroke	N	N	Y
		9*	M	56	Anoxia	N	N	Y
		10*	M	63	Anoxia	Y	N	Y
		11*	M	57	Stroke	Y	N	N
		12*	M	34	Anoxia	N	N	Y
		13*	F	34	Anoxia	N	N	N
Control (AF+HF)	Right Atrium	14	M	61	Stroke	N	N	N
		15	F	57	Stroke	Y	Y	Y
		16	F	47	Anoxia	Y	Y	N
		17*	F	74	Stroke	Y	N	Y
		18*	F	49	Gunshot wound	N	N	Y
		19*	F	46	Stroke	N	N	Y
		20	M	46	Anoxia	Y	Y	Y
		21	M	60	Anoxia	Y	Y	Y
		22	M	30	Anoxia	N	N	Y
	Left Atrium	23	F	66	Anoxia	N	N	Y
		24	M	74	Stroke	N	N	Y
		25	F	35	Anoxia	N	N	Y
		26*	F	74	Stroke	Y	N	Y
		27*	M	64	Stroke	N	N	Y
		28	F	67	Anoxia	Y	Y	N
		29*	F	49	Gunshot wound	N	N	Y
		30*	F	46	Stroke	N	N	Y

*Matched samples: #1, 7, 17, 26; #2, 9; #3, 10 #4, 11; #5, 12; #6, 13; #8, 27; #18, 29; #19, 30.

AF = atrial fibrillation; DM = diabetes mellitus; HF = heart failure; HTN = hypertension

Table S3. Summary of the clinical information of HF and AF+HF patients included in the present study.

						Comorbidities				Devices					
		Sample ID	Sex	Age (yr)	Diagnosis	CAD	Valve Disease	DM	Smoker	ICD	BiV	LVAD (mo)	Cardiac Surgery	Pre-Transplant EF (%)	Arrhythmias
HF	Right Atrium	31	F	65	NICM	CAD	sev MR, mild TR	Y	N	N	Y	13.7	CABG	20	
		32	M	52	NICM	N	sev MR, mod TR	N	N	Y	N	10.0		25	
		33*	F	54	NICM	CAD	sev MR, mod TR	Y	Y	N	Y	25.0		15	
	Left Atrium	34	M	42	ICM	CAD	mild TR	N	Y	Y	N	15.6		10	
		35*	F	54	NICM	CAD	sev MR, mod TR	Y	Y	N	Y	25.0		15	
		36	M	62	ICM	CAD	mild MR	N	Y	Y	N	7.1		15	VT
AF+HF	Right Atrium	37*	M	25	NICM	N	mod MR, sev TR	Y	N	Y	N	0		5	AF
		38	M	59	NICM	N	sev MR	N	N	Y	N	24.0	MVR	34	AF
		39*	F	45	NICM	N	mod MR, sev TR	N	N	Y	N	0	AVR	40	AF & VT
		40	M	64	NICM	N	mod TR	Y	Y	Y	N	22.8		20	AF
		41	M	60	NICM	N	NA	Y	Y	NA	NA	Yes		NA	AF
		42	M	63	ICM	Y	NA	N	Y	NA	NA	Yes		28	AF
		43	M	56	NICM	N	mod MR, mild TR	N	Y	N	Y	10.0		10	AF & VT
	Left Atrium	44*	M	25	NICM	N	mod MR, sev TR	Y	N	Y	N	0		5	AF
		45	F	78	NA	NA	NA	NA	NA	NA	NA	NA		NA	AF
		46*	F	45	NICM	N	mod MR, sev TR	N	N	Y	N	0	AVR	40	AF & VT
		47	F	52	ICM	CAD	NA	N	Y	Y	N	12.0	CABG	10	AF
		48	M	67	ICM	CAD	NA	N	N	Y	N	16.0	CABG	10	AF & VT
		49	M	47	NICM	N	mild MR, sev TR	Y	N	Y	N	20.2		19	AF & VT

*Matched samples: #33, 35; #37, 44; #39, 46.

AF = atrial fibrillation; AVR = aortic valve replacement; BiV = biventricular implantable cardioverter-defibrillator; CABG = Coronary artery bypass graft; CAD: coronary artery disease; DCM = dilated cardiomyopathy; DM = diabetes mellitus; EF = ejection fraction; ICD = implantable cardioverter-defibrillator; ICM = ischemic cardiomyopathy; LVAD = left ventricular assist device; MI = myocardial infarction; mod/sev MR = moderate/severe mitral regurgitation; mod/sev TR = moderate/severe tricuspid regurgitation; MVR = mitral valve repair; NA = not available from records; NICM = non-ischemic cardiomyopathy; NL = No LVAD; VT = ventricular tachycardia

Table S4. Differentially expressed RNA-sequencing transcripts from human atrial fibrillation with heart failure RA cardiomyocyte nuclei compared to non-failing RA cardiomyocyte nuclei

Gene ID	Fold Change: AF+HF RA versus NF RA	FDR
A2MP1	0.49	1.35E-04
A4GALT	2.51	5.99E-05
AACSP1	0.58	9.49E-03
AADACL3	0.61	2.38E-02
AADACL4	0.56	2.73E-02
AAK1	1.67	3.08E-03
AB015752.1	0.64	9.55E-03
ABBA01000935.2	0.66	1.44E-02
ABCA10	0.47	3.56E-06
ABCA12	0.65	2.19E-02
ABCA13	0.65	1.04E-02
ABCA4	0.63	1.90E-02
ABCA6	0.37	1.26E-10
ABCA9	0.29	4.44E-11
ABCB4	0.66	6.99E-03
ABCC12	0.65	3.24E-02
ABCC13	0.58	2.17E-03
ABCC2	0.64	2.55E-03
ABCC4	0.64	8.09E-04
ABCC5	1.64	2.37E-02
ABCD4	1.92	3.61E-03
ABCF3	1.67	2.70E-03
ABHD10	0.67	1.68E-02
ABHD2	2.99	1.66E-10
ABI3BP	0.60	1.68E-02
ABLIM1	1.58	1.63E-02
ABTB1	2.10	1.24E-02
ABTB2	2.42	7.22E-04
AC000372.1	0.56	4.32E-02
AC002383.1	0.62	1.90E-02
AC002384.2	0.51	1.55E-03
AC002428.1	0.56	4.56E-03
AC002454.1	0.63	4.72E-03
AC003092.1	0.62	4.02E-02
AC003973.2	0.46	3.95E-03
AC003984.1	0.52	4.25E-02
AC003988.1	0.63	3.52E-02
AC004009.1	0.65	4.88E-03
AC004052.1	0.64	1.64E-02
AC004053.1	0.59	2.55E-02
AC004076.1	1.64	1.21E-02
AC004160.1	0.54	1.12E-03
AC004231.1	0.61	9.00E-03
AC004485.1	0.61	1.46E-03
AC004540.1	0.66	4.87E-02
AC004690.2	0.53	6.81E-05
AC004704.1	0.60	6.78E-03
AC004784.1	0.58	8.26E-04
AC004830.1	0.67	8.32E-03
AC004852.2	0.59	4.14E-02
AC004870.4	0.44	5.76E-03
AC004917.1	0.66	2.62E-03
AC004936.1	0.57	4.37E-02
AC004980.1	1.66	1.52E-03
AC004990.1	0.55	3.13E-02
AC005008.2	0.56	1.03E-02
AC005037.1	1.58	2.14E-02
AC005062.1	0.66	9.11E-03
AC005064.1	0.66	1.71E-02
AC005144.1	0.61	2.72E-03
AC005162.3	0.49	1.59E-02
AC005165.1	0.52	5.56E-03
AC005280.1	0.58	3.14E-02
AC005307.1	0.62	3.90E-02
AC005394.2	0.40	1.14E-03
AC005532.1	0.64	3.92E-02
AC005699.1	0.57	2.80E-03
AC005772.1	0.66	8.04E-03

Gene ID	Fold Change: AF+HF RA versus NF RA	FDR
AC005833.3	1.53	2.34E-02
AC005885.1	0.61	2.02E-03
AC005899.8	2.02	3.99E-05
AC005909.1	0.41	4.30E-06
AC005972.4	0.63	5.36E-03
AC005999.1	0.47	6.81E-05
AC006013.1	0.61	9.30E-03
AC006059.1	1.71	8.90E-03
AC006148.1	0.55	7.86E-03
AC006206.2	0.60	1.13E-03
AC006296.2	0.66	4.42E-02
AC006299.1	0.57	5.84E-03
AC006305.1	0.60	2.60E-03
AC006547.1	3.77	1.69E-07
AC007091.1	0.58	1.27E-03
AC007100.1	0.36	3.64E-04
AC007106.1	0.61	1.89E-02
AC007207.1	0.54	8.36E-03
AC007221.2	0.62	3.96E-03
AC007277.1	0.66	2.02E-02
AC007285.1	0.64	1.50E-02
AC007319.1	0.56	1.46E-03
AC007333.1	0.49	6.46E-04
AC007333.2	0.55	9.87E-04
AC007342.4	0.60	2.19E-02
AC007364.1	0.63	7.29E-03
AC007376.2	2.99	3.26E-04
AC007402.1	0.61	7.06E-03
AC007424.1	0.65	1.81E-02
AC007463.1	0.60	2.17E-02
AC007491.1	0.65	5.56E-03
AC007529.2	0.58	5.80E-03
AC007563.2	0.66	3.55E-02
AC007569.1	1.85	4.04E-02
AC007603.3	0.57	4.52E-02
AC007611.1	0.61	1.13E-03
AC007637.1	1.56	3.54E-02
AC007848.1	0.55	5.23E-04
AC007966.1	0.45	2.80E-03
AC008080.3	0.41	6.69E-05
AC008109.1	0.65	1.69E-02
AC008127.1	0.55	4.93E-04
AC008163.1	0.63	4.99E-02
AC008164.1	0.60	1.07E-02
AC008267.3	2.05	5.35E-04
AC008415.1	0.62	1.64E-03
AC008443.1	1.65	1.38E-02
AC008496.2	2.07	2.62E-02
AC008554.1	0.59	5.91E-03
AC008568.1	0.64	3.02E-02
AC008662.1	0.61	1.61E-02
AC008725.1	0.59	1.18E-02
AC008770.1	1.75	1.12E-03
AC008794.1	0.56	3.62E-03
AC008825.1	0.53	6.26E-03
AC008840.1	0.66	4.19E-02
AC008897.2	0.63	2.23E-02
AC008937.1	0.57	2.24E-02
AC008938.1	0.61	4.51E-03
AC008969.1	1.77	1.03E-02
AC009055.1	0.62	4.21E-03
AC009055.2	0.56	3.30E-03
AC009119.2	2.13	1.19E-03
AC009120.2	1.71	3.18E-02
AC009139.2	0.64	1.03E-02
AC009158.1	0.56	4.18E-03
AC009264.1	0.61	3.64E-03
AC009313.1	0.62	1.61E-02
AC009435.1	0.63	4.85E-03
AC009478.1	0.56	9.34E-03
AC009495.1	0.62	4.07E-02
AC009502.1	0.62	1.65E-02
AC009554.1	0.59	9.42E-04
AC009654.1	0.51	7.33E-05
AC009950.1	0.61	3.04E-03
AC009975.1	0.61	9.08E-03
AC010099.4	0.62	2.00E-02
AC010132.1	0.60	3.91E-03

Gene ID	Fold Change: AF+HF RA versus NF RA	FDR
AC010145.1	0.64	2.80E-02
AC010149.1	0.62	9.49E-03
AC010198.1	0.65	7.10E-03
AC010230.1	0.61	1.03E-02
AC010329.5	0.62	9.94E-03
AC010343.3	0.62	5.75E-03
AC010395.1	0.62	3.03E-03
AC010511.1	0.64	1.50E-02
AC010522.1	2.03	1.20E-02
AC010528.1	0.58	3.85E-03
AC010531.1	1.82	1.25E-02
AC010542.3	1.57	3.12E-02
AC010601.1	0.65	9.87E-03
AC010618.3	1.74	1.18E-02
AC010620.2	0.64	3.67E-02
AC010754.1	0.56	4.67E-03
AC010884.1	0.64	5.51E-03
AC010983.1	0.57	2.15E-02
AC011095.1	0.57	8.68E-03
AC011124.1	0.65	3.04E-02
AC011131.1	0.55	1.45E-02
AC011290.1	0.63	6.27E-03
AC011306.1	0.62	3.39E-03
AC011450.1	2.04	6.40E-03
AC011483.2	0.59	2.57E-02
AC011586.1	0.55	1.53E-03
AC011754.1	0.53	8.38E-04
AC012349.1	0.61	1.08E-02
AC012409.2	0.50	2.99E-06
AC012414.5	0.58	1.90E-02
AC012560.1	0.64	2.15E-02
AC012593.1	0.57	1.44E-03
AC012625.1	0.59	9.09E-03
AC012668.3	0.66	2.53E-02
AC013391.2	0.65	2.19E-02
AC013460.1	0.65	4.89E-02
AC013470.2	0.64	6.99E-03
AC013546.1	0.63	2.67E-02
AC013652.1	0.62	2.53E-03

Gene ID	Fold Change: AF+HF RA versus NF RA	FDR
AC013727.1	0.63	4.14E-02
AC015522.1	0.65	3.90E-02
AC015574.1	0.66	3.51E-02
AC015813.2	1.75	1.63E-04
AC015845.2	2.31	5.79E-03
AC015908.2	0.61	1.91E-03
AC016152.1	0.60	6.07E-04
AC016582.1	0.60	1.90E-02
AC016723.1	0.66	8.17E-03
AC016766.1	0.60	4.14E-03
AC016831.5	6.04	1.16E-11
AC016903.2	0.62	4.51E-03
AC017007.3	2.88	2.17E-04
AC017007.4	1.83	3.89E-03
AC017048.2	0.57	1.66E-02
AC017091.1	0.64	3.39E-02
AC018437.2	0.63	2.67E-02
AC018541.1	0.58	1.59E-03
AC018554.1	0.61	2.00E-03
AC018618.1	0.63	5.40E-03
AC018638.4	3.15	4.16E-04
AC018653.1	0.63	3.12E-02
AC018680.1	0.64	1.96E-02
AC018697.1	0.64	7.51E-03
AC018709.1	0.63	4.30E-03
AC018730.2	0.59	3.79E-02
AC018731.1	0.65	1.84E-02
AC019117.3	0.62	2.30E-02
AC019330.1	0.56	2.20E-03
AC020637.1	0.55	1.47E-02
AC020687.1	0.63	2.61E-03
AC020718.1	0.62	1.65E-02
AC020743.2	0.55	2.04E-03
AC020905.1	0.59	2.19E-02
AC020916.1	3.13	1.62E-03
AC021079.1	0.64	3.37E-02
AC021086.1	0.62	1.74E-03
AC021192.1	0.62	3.44E-02
AC021242.2	0.55	1.53E-03

Gene ID	Fold Change: AF+HF RA versus NF RA	FDR	Gene ID	Fold Change: AF+HF RA versus NF RA	FDR
AC021678.2	0.60	7.30E-03	*AC025822.2*	0.65	4.64E-02
AC021733.1	0.63	1.51E-02	*AC025946.1*	0.63	2.95E-03
AC022126.1	0.58	3.82E-04	*AC026116.1*	0.63	3.18E-03
AC022140.1	0.54	2.74E-02	*AC026167.1*	0.63	2.10E-02
AC022167.2	2.49	8.38E-04	*AC026336.2*	0.59	2.46E-02
AC022398.1	0.60	2.18E-03	*AC026347.1*	0.60	7.10E-03
AC022523.1	0.66	7.99E-03	*AC026371.1*	0.64	7.97E-03
AC022568.1	0.61	1.82E-02	*AC026412.1*	1.53	4.37E-02
AC022639.1	0.60	8.41E-03	*AC026434.1*	0.60	1.75E-02
AC022695.2	0.59	2.38E-02	*AC026780.1*	0.59	3.54E-02
AC022733.2	0.57	1.65E-02	*AC027458.1*	0.59	2.22E-02
AC022762.1	0.63	6.65E-03	*AC027559.1*	0.64	4.62E-02
AC022784.6	0.65	4.54E-03	*AC027613.1*	0.58	1.24E-02
AC022796.1	0.57	1.50E-03	*AC027682.4*	1.56	2.19E-02
AC022858.1	0.66	4.21E-02	*AC027701.1*	0.63	7.06E-03
AC022905.1	0.58	1.63E-02	*AC034195.1*	0.54	4.39E-03
AC023202.1	0.40	3.63E-04	*AC035140.1*	0.64	1.80E-02
AC023355.1	0.66	9.90E-03	*AC036214.1*	0.62	1.23E-03
AC023442.3	0.54	3.34E-02	*AC037450.1*	0.65	2.78E-02
AC023469.1	0.63	3.19E-03	*AC037486.1*	0.55	1.74E-04
AC023511.1	0.36	5.88E-06	*AC058791.1*	4.80	2.15E-08
AC023669.1	0.63	4.37E-03	*AC060765.1*	0.63	2.73E-02
AC024022.1	0.66	6.82E-03	*AC060809.1*	0.55	7.30E-05
AC024132.1	0.61	2.18E-02	*AC062031.1*	0.57	1.60E-02
AC024230.1	0.63	1.19E-02	*AC063949.2*	0.58	3.31E-03
AC024257.1	0.62	2.45E-03	*AC063977.6*	0.62	2.75E-02
AC024382.1	0.56	1.58E-03	*AC063979.2*	0.63	3.74E-03
AC024405.2	0.65	1.05E-02	*AC064807.2*	0.61	8.36E-04
AC024475.4	0.64	1.15E-02	*AC068286.1*	0.62	4.09E-02
AC024600.1	0.62	2.56E-02	*AC068295.1*	0.57	1.39E-02
AC024901.1	0.64	3.03E-03	*AC068305.3*	0.64	3.69E-03
AC024909.1	0.63	5.42E-03	*AC068413.1*	0.61	6.31E-03
AC025034.1	0.56	3.33E-03	*AC068473.1*	0.65	1.33E-02
AC025244.1	0.62	3.68E-03	*AC068481.1*	0.62	1.99E-02
AC025428.2	0.61	2.90E-02	*AC068587.6*	0.64	1.62E-02
AC025437.4	0.59	2.37E-03	*AC068599.1*	0.66	1.14E-02
AC025569.1	0.62	1.60E-03	*AC068631.1*	0.65	7.77E-03
AC025575.2	0.63	2.88E-02	*AC068633.1*	0.64	7.99E-03
AC025809.1	0.62	4.85E-03	*AC068643.1*	0.56	2.40E-03

Gene ID	Fold Change: AF+HF RA versus NF RA	FDR
AC068672.2	0.60	1.37E-03
AC068722.1	0.65	6.40E-03
AC068759.1	0.57	2.55E-03
AC068875.1	0.61	2.24E-03
AC069208.1	0.60	1.59E-02
AC073050.1	0.65	5.02E-03
AC073062.1	0.62	2.24E-02
AC073172.2	0.63	8.27E-03
AC073530.1	0.65	2.95E-02
AC073534.1	1.76	3.11E-03
AC073834.1	0.50	1.21E-04
AC073869.1	2.30	5.10E-06
AC073941.1	0.57	2.02E-02
AC073987.1	0.61	2.50E-03
AC074032.1	1.65	1.80E-03
AC074237.1	0.57	4.38E-03
AC074286.1	0.62	9.70E-04
AC078788.1	0.59	2.07E-02
AC078881.1	0.65	2.38E-03
AC078929.1	0.58	1.14E-02
AC079098.1	0.62	2.13E-03
AC079298.3	0.51	4.20E-05
AC079340.1	0.63	1.85E-02
AC079340.2	0.61	2.36E-02
AC079362.1	0.64	1.58E-02
AC079380.1	0.51	5.42E-03
AC079414.1	0.49	1.28E-02
AC079584.1	0.65	2.96E-02
AC079760.1	0.66	1.61E-02
AC079760.2	0.59	1.48E-03
AC079763.1	0.52	6.46E-04
AC079801.1	0.62	6.34E-03
AC079896.1	0.52	1.59E-03
AC079907.1	0.55	1.07E-03
AC079917.1	0.60	9.44E-03
AC079942.1	0.56	2.17E-03
AC079950.1	0.65	2.72E-02
AC083805.1	0.63	3.61E-02
AC083864.2	0.58	2.34E-03

Gene ID	Fold Change: AF+HF RA versus NF RA	FDR
AC083906.3	0.53	1.63E-02
AC083923.2	0.60	3.32E-03
AC083949.1	0.56	1.57E-02
AC084026.1	0.60	1.60E-02
AC084048.1	0.54	2.39E-03
AC084116.2	0.65	2.02E-02
AC084200.1	0.57	1.86E-03
AC084337.2	1.56	1.84E-03
AC084357.2	0.53	4.08E-04
AC084759.2	0.58	1.51E-02
AC084768.1	0.46	7.68E-05
AC084816.1	0.62	2.82E-03
AC087235.1	0.63	7.43E-03
AC087269.1	0.62	1.69E-03
AC087280.2	0.62	1.59E-03
AC087379.1	0.58	1.96E-03
AC087627.1	0.53	3.93E-03
AC087633.2	0.62	1.41E-03
AC087672.3	0.63	1.67E-02
AC087798.1	0.48	2.08E-03
AC087857.1	0.54	1.13E-03
AC087863.1	0.65	5.84E-03
AC087894.2	0.60	2.01E-03
AC087897.2	0.60	1.91E-03
AC090001.1	0.61	2.02E-03
AC090023.2	0.51	1.62E-04
AC090114.3	1.85	2.02E-04
AC090124.1	0.58	8.14E-04
AC090125.1	0.66	7.91E-03
AC090150.1	0.51	4.64E-03
AC090155.2	0.63	3.45E-02
AC090159.1	0.60	1.60E-02
AC090282.1	0.67	3.36E-02
AC090358.1	0.56	1.20E-03
AC090376.1	0.60	1.23E-03
AC090403.1	0.55	1.62E-03
AC090458.1	0.59	2.79E-03
AC090503.2	0.58	2.71E-02
AC090527.2	0.63	4.64E-02

Gene ID	Fold Change: AF+HF RA versus NF RA	FDR
AC090568.2	0.65	1.63E-02
AC090572.2	0.55	7.29E-03
AC090630.1	0.40	2.70E-03
AC090791.1	0.62	1.99E-02
AC090796.1	0.65	4.30E-03
AC090809.1	0.64	1.68E-02
AC090833.1	0.65	3.37E-02
AC090897.3	0.46	2.26E-05
AC090912.1	0.60	3.43E-02
AC090987.1	0.58	5.78E-03
AC091078.1	0.62	1.13E-03
AC091096.2	0.61	7.86E-03
AC091114.1	0.66	1.89E-02
AC091534.1	0.47	2.46E-04
AC091691.2	0.62	1.96E-02
AC091860.2	0.53	1.03E-02
AC091901.1	0.54	6.31E-03
AC091939.1	0.64	7.42E-03
AC091946.1	0.60	3.90E-02
AC091959.3	0.60	7.63E-03
AC092078.2	0.61	1.19E-02
AC092138.2	0.64	1.97E-02
AC092422.1	0.62	1.15E-02
AC092573.2	0.61	2.60E-02
AC092598.1	0.50	5.85E-03
AC092637.1	0.52	1.08E-03
AC092650.1	0.66	3.93E-03
AC092673.1	0.62	1.73E-02
AC092687.1	2.06	4.60E-03
AC092691.1	0.63	2.86E-02
AC092783.1	0.58	2.37E-03
AC092813.2	0.63	6.93E-03
AC092819.1	0.58	1.41E-02
AC092821.3	0.65	1.77E-02
AC092920.1	0.64	7.38E-03
AC092957.1	0.66	2.51E-02
AC092979.1	0.63	1.75E-02
AC093277.1	0.63	3.33E-02
AC093297.2	0.61	1.64E-03

Gene ID	Fold Change: AF+HF RA versus NF RA	FDR
AC093331.2	0.56	2.70E-03
AC093459.1	0.60	6.81E-03
AC093523.1	0.65	8.01E-03
AC093535.2	0.61	1.41E-02
AC093599.1	0.62	1.09E-02
AC093655.1	0.66	4.55E-02
AC093722.1	0.57	8.28E-03
AC093725.2	0.64	4.89E-02
AC093730.1	0.41	1.69E-02
AC093766.1	0.60	2.55E-03
AC093768.1	0.52	1.59E-03
AC093772.1	0.63	1.06E-02
AC093817.1	0.59	4.29E-02
AC093821.1	0.63	2.67E-02
AC093829.1	0.48	4.44E-03
AC093857.1	0.53	2.75E-04
AC093865.1	0.56	1.50E-03
AC093909.2	0.63	1.43E-02
AC093911.1	0.48	1.54E-04
AC095032.1	0.60	4.34E-03
AC095050.1	0.54	1.01E-02
AC096543.1	0.59	2.93E-03
AC096554.1	0.55	2.60E-03
AC096588.1	0.65	2.85E-02
AC096637.2	0.51	2.96E-03
AC096666.1	0.47	8.73E-04
AC096669.1	0.56	3.33E-02
AC096677.1	1.59	3.73E-02
AC096719.1	0.59	7.55E-03
AC096751.1	0.60	2.86E-02
AC096773.1	0.65	7.22E-03
AC097059.1	0.62	1.50E-03
AC097066.1	0.60	2.81E-02
AC097372.1	0.55	2.93E-03
AC097374.1	0.63	1.32E-03
AC097375.1	0.56	7.86E-03
AC097467.3	0.61	2.24E-02
AC097478.1	0.61	7.61E-03
AC097480.1	0.60	1.63E-03

Gene ID	Fold Change: AF+HF RA versus NF RA	FDR
AC097501.2	0.44	3.80E-05
AC097515.1	0.63	1.37E-02
AC097713.1	0.57	9.67E-03
AC097717.1	0.65	7.24E-03
AC098613.1	0.59	3.30E-03
AC098679.1	0.53	4.01E-03
AC099060.1	0.53	3.82E-03
AC099062.1	0.63	6.54E-03
AC099063.4	0.56	1.75E-02
AC099511.1	0.59	1.60E-02
AC099518.1	0.60	3.97E-02
AC099520.1	0.64	3.34E-02
AC099522.1	0.62	5.68E-03
AC099542.1	0.50	1.56E-03
AC099560.1	0.59	6.76E-04
AC099566.1	0.58	2.97E-03
AC099567.1	0.65	2.69E-02
AC099673.1	0.58	1.99E-02
AC099687.1	0.50	4.62E-02
AC099753.1	0.63	2.45E-02
AC099792.1	0.59	6.37E-03
AC099805.1	0.63	4.97E-03
AC100768.2	0.59	8.15E-03
AC100781.1	0.56	1.22E-03
AC100801.1	0.61	2.66E-03
AC100807.1	0.66	3.27E-02
AC100818.1	0.63	1.84E-02
AC100830.1	2.64	6.05E-08
AC100844.1	0.51	6.49E-03
AC100849.1	0.61	2.46E-03
AC100872.1	0.53	9.51E-03
AC103409.1	0.66	1.15E-02
AC103719.1	0.51	4.23E-02
AC103760.1	0.55	4.30E-03
AC103796.1	0.56	9.42E-04
AC103923.1	0.66	3.91E-02
AC103957.1	0.63	3.86E-02
AC104036.1	0.55	3.55E-03
AC104051.1	0.63	1.57E-02

Gene ID	Fold Change: AF+HF RA versus NF RA	FDR
AC104078.1	0.56	7.31E-04
AC104078.2	0.66	1.58E-02
AC104119.1	0.60	2.58E-02
AC104151.1	0.61	3.14E-02
AC104162.1	0.63	1.44E-02
AC104232.1	0.45	1.58E-04
AC104233.1	0.58	1.94E-02
AC104248.1	0.56	3.05E-02
AC104257.1	0.50	1.15E-02
AC104435.2	0.62	1.43E-02
AC104574.2	0.53	9.39E-06
AC104596.1	0.62	8.23E-03
AC104663.1	0.53	1.17E-02
AC104777.1	0.65	1.51E-02
AC104777.2	0.49	1.59E-03
AC104803.1	0.50	2.38E-04
AC104825.2	2.36	1.22E-04
AC105021.1	0.64	1.03E-02
AC105180.1	0.66	7.60E-03
AC105389.3	0.63	2.50E-03
AC105411.1	0.66	9.05E-03
AC105919.1	0.60	1.08E-02
AC106053.1	0.65	4.64E-02
AC106729.1	0.50	9.67E-03
AC106744.1	0.65	3.50E-02
AC106798.1	0.54	5.56E-03
AC106799.2	0.62	5.45E-03
AC106865.1	0.66	1.18E-02
AC106874.1	0.60	5.90E-04
AC106892.1	0.61	1.80E-02
AC106895.2	0.61	2.17E-02
AC107029.1	0.63	1.89E-02
AC107029.2	0.60	3.95E-03
AC107032.2	0.59	2.09E-03
AC107068.2	0.35	2.68E-02
AC107373.1	0.59	1.39E-02
AC107396.1	0.66	3.57E-02
AC108062.1	0.63	1.58E-02
AC108515.1	0.59	3.81E-02

Gene ID	Fold Change: AF+HF RA versus NF RA	FDR
AC108517.1	0.59	3.39E-03
AC108748.1	0.39	1.06E-04
AC108752.1	0.61	4.41E-02
AC108866.1	0.67	2.72E-02
AC108868.1	0.62	1.01E-02
AC108941.2	0.52	2.29E-04
AC109349.1	0.61	2.33E-02
AC109439.2	0.54	2.30E-03
AC109462.2	0.62	4.54E-02
AC109466.1	0.63	2.89E-02
AC109492.1	0.30	3.10E-08
AC109588.1	0.56	1.45E-02
AC109830.1	0.61	9.56E-03
AC110009.1	0.58	1.22E-02
AC110023.1	0.61	4.02E-03
AC110079.1	2.34	7.53E-05
AC110079.2	0.62	1.44E-02
AC110491.3	0.59	5.67E-03
AC111000.4	0.58	7.63E-03
AC111194.2	0.61	9.45E-03
AC111198.1	0.65	3.53E-02
AC112198.2	0.65	2.06E-02
AC112719.2	0.61	2.18E-02
AC113137.1	0.62	4.91E-02
AC113347.4	0.61	2.60E-02
AC113383.1	0.53	1.60E-03
AC113386.1	0.64	3.84E-02
AC113418.1	0.54	1.36E-02
AC114316.2	0.56	1.81E-03
AC114321.1	0.57	2.28E-03
AC114400.1	0.60	1.69E-03
AC114550.1	0.56	4.33E-03
AC114689.3	0.60	3.89E-03
AC114947.1	0.61	3.09E-02
AC115220.1	0.61	2.10E-02
AC115622.1	0.58	2.61E-03
AC116049.2	0.62	9.27E-03
AC116337.3	0.61	2.40E-03
AC116345.1	0.65	3.17E-03

Gene ID	Fold Change: AF+HF RA versus NF RA	FDR
AC116345.3	0.60	1.23E-02
AC116616.1	0.65	2.08E-02
AC116634.1	0.58	1.22E-02
AC117386.2	0.66	1.80E-02
AC117460.1	0.60	3.28E-02
AC117465.1	0.61	2.04E-02
AC117513.1	0.60	2.15E-03
AC117569.1	0.62	4.42E-02
AC118942.1	0.53	4.29E-03
AC119674.1	0.59	3.04E-02
AC120193.1	0.63	1.02E-02
AC121154.1	0.60	6.05E-03
AC123767.1	0.66	4.25E-02
AC123905.1	0.58	1.14E-02
AC124290.1	0.64	6.99E-03
AC124854.1	0.60	7.32E-03
AC125232.1	1.56	4.67E-02
AC125618.1	0.61	6.29E-03
AC126121.2	0.66	6.79E-03
AC126177.4	0.59	3.03E-03
AC128685.1	0.62	3.40E-03
AC128689.1	0.63	1.31E-02
AC128707.1	0.60	1.18E-02
AC129807.1	0.65	2.47E-02
AC131254.1	0.65	1.73E-02
AC131956.2	0.50	9.32E-04
AC132008.2	1.59	5.50E-03
AC133634.1	0.63	2.68E-02
AC134043.1	0.57	6.92E-03
AC135178.3	1.64	2.19E-02
AC136759.1	0.63	8.67E-03
AC137579.1	0.63	1.54E-02
AC137735.1	0.66	3.54E-02
AC137761.1	0.39	2.20E-03
AC137800.1	0.50	5.51E-04
AC138150.1	1.70	4.63E-03
AC138915.3	0.63	1.80E-02
AC139713.2	0.62	6.65E-03
AC139769.2	0.66	5.79E-03

Gene ID	Fold Change: AF+HF RA versus NF RA	FDR
AC139887.2	2.02	6.46E-04
AC142384.1	0.46	4.43E-04
AC215219.1	2.47	1.62E-05
AC239584.1	0.59	1.60E-02
AC241377.2	0.57	6.59E-03
AC241952.1	1.75	2.10E-03
AC243772.3	0.52	1.03E-02
AC244021.1	0.40	4.01E-02
AC244205.1	0.53	7.52E-03
AC244258.1	0.62	1.35E-03
AC244517.2	0.59	4.43E-03
AC244669.1	1.88	3.06E-02
AC245054.1	0.56	4.57E-03
AC245123.1	0.62	2.21E-03
AC245128.1	0.63	4.37E-02
AC245297.3	1.90	2.79E-03
AC245519.1	0.53	4.33E-03
AC246817.2	0.63	2.80E-02
ACACB	1.79	6.34E-04
ACAD10	1.55	9.41E-03
ACBD4	1.57	3.82E-02
ACER2	0.54	2.37E-04
ACIN1	1.55	6.20E-03
ACLY	0.63	3.76E-02
ACO2	1.72	5.51E-04
ACOT11	2.22	4.97E-04
ACOT12	0.62	1.66E-03
ACOX3	2.41	5.71E-07
ACSF3	1.90	7.99E-05
ACSL5	0.64	8.13E-03
ACSM2A	0.61	2.84E-02
ACSM2B	0.66	4.99E-02
ACSM3	0.57	5.05E-03
ACSS1	2.17	1.16E-03
ACTA2	0.55	3.32E-02
ACTG1P22	0.66	3.67E-02
ACTL8	0.63	4.85E-03
ACTN1	1.54	3.97E-02
ACTN2	2.15	3.07E-04

Gene ID	Fold Change: AF+HF RA versus NF RA	FDR
ACTN4	1.70	3.94E-03
ACVR1C	0.63	1.01E-02
ADAM18	0.65	2.44E-02
ADAM22	0.66	7.29E-03
ADAMTS12	0.66	2.69E-02
ADAMTS16	0.66	3.05E-03
ADAMTS18	0.62	1.03E-02
ADAMTS20	0.62	7.99E-03
ADAMTS3	0.52	1.81E-04
ADAMTS5	0.51	5.03E-03
ADAMTS6	0.64	2.05E-02
ADAMTS7P4	0.63	1.77E-03
ADAMTSL2	0.56	3.42E-02
ADAMTSL4-AS1	2.40	4.27E-04
ADAMTSL5	1.65	4.31E-02
ADCY2	0.61	1.52E-03
ADCY6	3.24	1.06E-04
ADCY8	0.63	5.20E-03
ADGB	0.64	2.74E-02
ADGRB3	0.52	1.58E-04
ADGRD1	2.28	9.33E-04
ADGRF2	0.57	2.07E-02
ADGRF5	0.51	3.40E-06
ADGRG6	0.51	1.37E-03
ADGRL3	0.56	2.49E-03
ADGRL4	0.48	4.27E-04
ADH1B	0.18	1.40E-10
ADHFE1	1.68	1.02E-03
ADI1	1.60	5.28E-03
ADORA1	1.73	3.36E-02
ADPRHL1	1.97	4.55E-04
ADTRP	0.61	9.37E-04
AES	1.50	4.66E-02
AF121898.1	0.60	8.79E-03
AF123462.1	0.57	1.61E-03
AF127577.4	0.66	1.56E-02
AF130351.1	0.65	1.22E-02
AF130359.1	0.60	9.19E-03
AF241725.1	0.60	3.85E-02

Gene ID	Fold Change: AF+HF RA versus NF RA	FDR
AF279873.3	0.63	4.52E-03
AFF4	1.67	3.32E-03
AFG3L1P	1.91	3.20E-05
AFG3L2	1.67	9.36E-04
AGA	0.51	1.50E-04
AGAP6	1.61	8.37E-03
AGBL1	0.65	7.06E-03
AGBL2	0.67	3.27E-02
AGO2	1.98	6.17E-07
AGPAT3	1.85	7.99E-05
AGPAT5	0.63	2.06E-03
AGR2	0.65	1.36E-02
AHDC1	1.79	9.61E-03
AICDA	0.47	6.46E-04
AIM2	0.64	3.29E-03
AJ006995.1	0.56	6.70E-03
AK5	0.62	7.33E-04
AK9	0.66	8.83E-03
AKAIN1	0.66	3.63E-02
AKAP12	0.62	2.71E-02
AKAP8	1.78	1.02E-04
AKAP8L	1.91	9.64E-05
AKIRIN1	1.79	1.13E-04
AKR1B15	0.64	2.53E-02
AKR1C1	0.41	4.61E-04
AKR1C2	0.47	1.07E-04
AKR1C3	0.65	9.96E-03
AKR1C6P	0.63	9.11E-03
AKR1C8P	0.54	1.88E-02
AL008638.1	0.58	2.33E-02
AL020995.1	1.65	4.33E-03
AL021026.1	0.53	7.79E-04
AL021408.1	0.45	2.99E-04
AL021707.2	1.99	2.68E-04
AL021877.2	0.54	4.67E-03
AL022068.1	0.55	5.51E-03
AL022100.1	0.62	2.04E-02
AL023495.1	0.63	2.32E-03
AL023755.1	0.65	3.71E-02

Gene ID	Fold Change: AF+HF RA versus NF RA	FDR
AL031686.1	0.43	7.87E-07
AL031985.4	0.59	1.02E-02
AL033381.1	0.63	2.51E-02
AL034347.1	0.59	9.30E-03
AL035458.2	1.91	3.94E-03
AL049646.1	0.66	5.99E-03
AL049649.1	0.63	9.04E-03
AL049812.2	0.61	5.84E-03
AL049844.1	0.52	6.76E-04
AL050403.2	0.57	4.39E-05
AL079307.1	0.60	2.92E-03
AL109659.1	0.59	4.30E-02
AL109807.1	0.64	4.46E-02
AL109936.8	1.68	4.57E-03
AL110505.1	0.64	3.26E-02
AL117329.1	0.65	1.30E-02
AL117337.1	0.56	7.62E-03
AL118523.1	0.56	2.43E-04
AL121781.1	0.63	6.05E-03
AL121852.1	0.65	3.74E-03
AL121936.1	0.39	1.99E-04
AL121956.1	0.62	2.69E-02
AL122019.1	0.62	3.22E-02
AL132719.1	0.55	1.26E-03
AL132857.2	0.61	1.38E-03
AL132996.1	0.61	3.92E-03
AL133346.1	0.44	5.52E-07
AL133372.2	0.55	1.18E-02
AL133464.1	0.57	1.59E-03
AL135908.1	0.64	1.45E-02
AL136181.1	0.55	1.73E-02
AL136295.1	1.95	4.46E-04
AL136460.1	0.61	3.87E-02
AL136537.2	0.58	2.95E-02
AL136985.3	0.63	1.32E-02
AL137002.2	1.82	3.51E-02
AL137129.1	0.60	1.19E-02
AL137220.1	0.61	1.42E-02
AL137224.1	0.66	4.81E-02

Gene ID	Fold Change: AF+HF RA versus NF RA	FDR
AL137230.2	0.65	4.21E-03
AL137247.1	1.83	3.81E-03
AL138731.1	0.48	5.53E-04
AL138737.1	0.54	1.13E-03
AL138767.2	0.55	5.44E-03
AL138962.1	0.65	2.35E-02
AL139010.1	0.64	1.01E-02
AL139020.1	0.60	4.68E-02
AL139023.1	0.66	4.04E-02
AL139042.1	0.65	1.59E-02
AL139260.1	1.84	9.74E-03
AL139806.1	0.50	3.55E-03
AL157378.1	0.62	3.44E-02
AL157400.3	0.63	2.33E-02
AL158090.1	0.65	3.87E-02
AL158152.2	1.52	8.00E-03
AL158154.2	0.51	6.35E-04
AL158175.1	0.66	2.12E-02
AL160162.1	0.61	4.42E-02
AL160272.1	0.51	2.07E-03
AL160287.1	0.56	3.92E-03
AL160408.6	0.58	3.66E-02
AL161616.2	0.54	2.13E-03
AL161630.1	0.51	1.41E-03
AL161716.1	0.59	1.57E-02
AL161740.1	0.56	7.25E-04
AL161751.1	0.65	4.11E-02
AL161912.1	0.61	4.74E-02
AL161935.3	0.57	2.94E-02
AL162231.2	1.82	4.26E-03
AL162254.1	0.53	6.34E-04
AL162384.1	0.65	4.95E-02
AL162414.1	0.63	9.84E-03
AL162511.1	0.63	4.15E-03
AL162726.3	0.65	1.25E-02
AL163932.1	0.63	5.96E-03
AL353052.1	0.66	2.18E-02
AL353148.1	0.56	6.08E-03
AL353743.4	0.56	4.86E-03
AL353753.1	0.61	1.37E-02
AL353780.1	0.58	1.77E-02
AL354754.1	0.48	1.10E-02
AL354809.1	0.59	7.99E-05
AL354821.1	0.54	5.31E-03
AL354863.1	0.63	1.01E-02
AL354896.1	0.57	2.80E-03
AL354994.1	0.62	2.38E-02
AL355076.2	0.64	7.21E-03
AL355303.1	0.63	5.13E-03
AL355306.2	0.63	3.68E-02
AL355339.1	0.41	2.67E-02
AL355390.2	0.60	2.05E-03
AL355838.1	0.65	4.83E-03
AL355860.1	0.64	1.56E-02
AL355922.5	0.58	1.46E-02
AL356022.1	0.64	1.09E-02
AL356108.1	0.66	5.01E-03
AL356258.1	0.64	8.24E-03
AL356272.1	0.66	4.65E-02
AL356277.3	0.57	1.57E-02
AL356421.2	0.56	2.32E-03
AL356441.1	0.62	6.45E-03
AL356479.1	0.65	3.53E-02
AL357052.1	0.54	1.55E-03
AL357054.2	0.66	4.01E-03
AL357060.1	0.62	9.65E-03
AL357060.2	0.65	1.17E-02
AL357143.1	0.63	1.32E-02
AL357153.3	0.55	8.91E-03
AL357314.1	0.47	6.28E-04
AL357507.1	0.64	1.08E-02
AL358292.1	0.58	2.14E-03
AL358335.2	0.64	4.56E-02
AL358975.1	0.55	2.00E-03
AL359075.2	0.57	1.09E-03
AL359232.1	0.65	1.92E-02
AL359313.1	0.60	1.34E-03
AL359551.1	0.64	8.13E-03

Gene ID	Fold Change: AF+HF RA versus NF RA	FDR
AL359636.1	0.61	3.33E-03
AL359715.2	0.61	6.37E-03
AL359915.1	0.66	6.05E-03
AL359924.1	0.62	3.35E-03
AL360007.1	0.41	1.23E-05
AL360014.1	0.59	1.24E-02
AL365204.1	0.51	1.63E-03
AL365259.1	0.65	6.85E-03
AL365275.1	0.55	2.84E-02
AL365277.1	2.61	7.73E-04
AL365295.1	0.61	7.87E-04
AL390838.1	0.60	5.19E-03
AL390866.1	0.59	6.79E-03
AL390961.1	0.64	2.01E-02
AL391117.1	0.63	1.05E-02
AL391361.2	0.66	4.33E-02
AL392023.2	0.58	5.71E-04
AL392086.1	0.63	1.04E-02
AL441943.1	0.61	3.47E-02
AL442163.1	0.62	2.47E-02
AL445218.1	0.48	4.90E-03
AL445250.1	0.65	3.79E-02
AL445307.1	1.95	3.56E-02
AL445430.1	0.60	3.46E-03
AL445430.2	0.62	5.23E-03
AL445433.2	0.63	1.73E-02
AL445483.1	0.52	1.94E-04
AL445584.2	0.63	1.06E-02
AL445623.2	0.56	1.40E-03
AL449266.1	0.66	2.68E-02
AL450322.2	0.48	6.64E-04
AL450426.1	0.58	1.31E-02
AL451164.1	0.66	4.98E-02
AL512638.2	0.61	1.83E-02
AL513323.1	0.65	7.57E-03
AL583808.1	0.62	1.46E-02
AL583859.2	0.66	3.47E-02
AL589740.1	0.66	4.23E-02
AL590483.1	0.64	4.18E-02

Gene ID	Fold Change: AF+HF RA versus NF RA	FDR
AL591178.1	1.73	1.51E-02
AL591543.1	0.61	2.58E-02
AL591885.1	0.64	5.31E-03
AL592182.1	0.56	5.76E-03
AL592494.1	0.38	1.61E-06
AL596220.1	0.62	9.30E-03
AL596275.2	0.58	1.32E-03
AL603840.1	0.58	1.23E-04
AL606970.3	0.60	4.50E-02
AL627443.1	0.58	1.98E-02
AL671511.2	0.65	8.83E-03
AL672167.1	0.63	4.51E-03
AL691515.2	0.51	2.55E-03
AL713851.1	0.63	6.17E-03
AL731577.2	1.76	2.67E-04
AL807761.4	0.60	1.31E-03
AL954650.1	0.65	4.41E-02
ALCAM	0.27	2.24E-04
ALDH1A1	0.54	1.23E-03
ALDH1B1	0.54	5.06E-03
ALDH1L1	1.52	2.46E-02
ALDOA	0.37	5.47E-08
ALG12	1.99	4.00E-03
ALPK1	0.65	1.74E-03
ALPK2	1.70	1.36E-02
ALPK3	2.34	2.17E-05
ALS2CL	2.03	1.89E-03
AMBN	0.54	1.61E-03
AMDHD1	0.65	3.51E-02
AMZ2P1	1.64	5.50E-03
ANAPC13	0.57	3.10E-02
ANAPC2	1.72	2.35E-02
ANGPT1	0.31	5.06E-04
ANKEF1	0.60	3.66E-03
ANKFN1	0.61	2.18E-02
ANKH	1.53	3.83E-03
ANKRD11	1.73	6.23E-04
ANKRD18CP	0.62	3.91E-03
ANKRD18DP	0.63	3.91E-02

Gene ID	Fold Change: AF+HF RA versus NF RA	FDR
ANKRD20A7P	0.52	1.28E-02
ANKRD20A9P	0.61	2.46E-03
ANKRD23	2.01	1.71E-02
ANKRD30BL	0.63	8.37E-03
ANKRD31	0.64	2.26E-03
ANKRD36	0.60	1.19E-03
ANKRD40	1.71	2.39E-03
ANKRD42	0.67	1.18E-02
ANKRD49	0.60	2.09E-02
ANKRD50	0.53	2.11E-03
ANKRD52	1.60	2.06E-03
ANKRD54	1.63	7.59E-03
ANKRD55	0.64	2.05E-03
ANKRD62	0.57	1.01E-02
ANKRD7	0.63	1.52E-02
ANKRD9	1.65	1.29E-02
ANKS1B	0.66	1.41E-02
ANKUB1	0.60	6.92E-04
ANKZF1	1.96	1.65E-03
ANLN	0.58	3.72E-02
ANO2	0.66	2.02E-02
ANO3	0.60	4.43E-03
ANO4	0.63	4.49E-03
ANXA10	0.63	1.67E-02
ANXA11	1.52	1.35E-02
ANXA3	0.54	1.03E-03
AOC3	0.46	4.03E-02
AOX1	0.61	4.99E-02
AP000282.1	0.63	2.95E-03
AP000445.2	0.57	9.05E-03
AP000721.1	1.92	2.33E-03
AP000769.1	1.75	7.90E-03
AP000797.3	0.58	5.31E-03
AP000924.1	0.63	7.99E-03
AP001025.3	0.63	2.04E-03
AP001033.1	0.59	3.13E-03
AP001043.1	0.65	1.53E-02
AP001107.9	1.70	1.30E-02
AP001109.1	0.62	7.87E-03

Gene ID	Fold Change: AF+HF RA versus NF RA	FDR
AP001122.1	0.53	1.06E-02
AP001266.2	1.57	1.84E-02
AP001372.1	0.66	6.10E-03
AP001574.1	0.55	1.47E-02
AP001599.1	0.57	4.22E-02
AP001605.1	0.58	4.14E-04
AP001803.2	0.58	8.01E-03
AP001830.2	0.54	1.99E-03
AP001993.1	0.59	1.33E-03
AP001993.2	0.54	1.21E-03
AP001999.1	0.61	2.64E-02
AP002001.1	0.46	1.62E-04
AP002444.1	0.59	1.26E-02
AP002518.2	1.86	9.46E-04
AP002784.1	0.55	8.64E-04
AP003031.2	0.64	2.32E-02
AP003049.2	0.65	6.56E-03
AP003066.1	0.58	5.00E-04
AP003108.2	2.03	3.62E-06
AP003121.1	0.61	4.15E-03
AP003123.1	0.64	2.67E-02
AP003171.2	0.65	4.52E-02
AP003174.1	0.61	4.59E-03
AP003399.1	0.58	4.88E-04
AP003481.1	0.58	2.60E-03
AP003730.2	0.57	3.37E-02
AP003900.1	0.54	1.31E-03
AP005121.1	0.65	3.65E-02
AP005203.1	0.65	5.94E-03
AP005209.1	0.57	1.71E-03
AP005328.1	0.64	1.35E-02
AP005436.1	0.64	3.11E-02
AP006222.1	0.56	9.19E-03
AP3D1	1.91	2.97E-05
APBB1IP	0.57	3.07E-04
APCDD1	0.50	3.03E-04
APELA	0.38	2.97E-05
APOL6	0.60	6.95E-03
APOOP5	0.57	2.42E-02

Gene ID	Fold Change: AF+HF RA versus NF RA	FDR
APP	0.61	7.50E-04
APPL2	1.86	1.52E-04
AQP4-AS1	0.59	2.42E-03
ARAP2	0.59	9.56E-03
AREG	4.98	2.39E-02
ARF4	1.84	3.87E-04
ARFGAP1	1.84	3.87E-03
ARHGAP17	2.10	4.62E-05
ARHGAP20	0.62	2.70E-02
ARHGAP23	1.52	1.92E-02
ARHGAP24	1.68	2.37E-03
ARHGAP25	0.52	3.68E-03
ARHGAP26	1.52	1.44E-02
ARHGAP28	0.59	2.18E-03
ARHGDIB	0.45	3.60E-05
ARHGEF1	1.69	9.11E-03
ARHGEF10L	1.90	2.35E-04
ARHGEF17	1.54	4.77E-03
ARHGEF26-AS1	0.61	2.95E-03
ARHGEF38	0.66	2.75E-02
ARID5A	2.52	3.55E-03
ARIH2	1.61	2.36E-03
ARL10	1.59	3.27E-03
ARL15	0.65	3.76E-03
ARL6	0.61	3.66E-03
ARMC3	0.65	1.31E-02
ARMC4	0.62	2.05E-03
ARPP21	0.55	9.90E-04
ARRDC4	2.11	1.76E-02
ARSJ	0.45	7.28E-03
ART3	0.56	1.93E-02
ASAH2	0.62	1.44E-03
ASB16-AS1	2.07	7.71E-04
ASB3	0.66	6.09E-03
ASB4	0.60	1.29E-02
ASCC2	1.98	2.63E-05
ASIC2	0.65	1.63E-02
ASIP	0.63	6.75E-03
ASPG	1.68	1.03E-02
ASPM	0.61	2.31E-02
ASPSCR1	1.76	1.97E-02
ASTN1	0.63	1.43E-02
ASZ1	0.54	1.65E-02
ATAD3B	1.69	9.72E-04
ATAT1	1.73	1.39E-03
ATG16L2	1.58	1.71E-02
ATG4B	2.40	4.60E-08
ATG4D	2.02	3.64E-04
ATP10B	0.63	1.06E-03
ATP11A	1.66	1.34E-03
ATP13A1	1.54	1.07E-02
ATP13A3	1.69	5.34E-03
ATP13A4	0.65	5.18E-03
ATP13A5	0.66	7.25E-03
ATP1A2	1.65	9.67E-03
ATP1B1	1.84	1.61E-05
ATP2A2	1.80	9.44E-04
ATP2B1	0.61	7.92E-03
ATP5B	0.59	1.20E-03
ATP5D	0.52	7.68E-03
ATP5E	0.41	1.96E-05
ATP5G3	0.48	7.17E-06
ATP5I	0.49	5.20E-03
ATP6V0E2-AS1	1.84	8.75E-03
ATP8A2P3	0.55	2.36E-04
ATP8B4	0.61	4.17E-03
ATPIF1	0.59	5.58E-03
ATXN2L	2.15	5.39E-04
ATXN7L3B	0.60	1.97E-02
ATXN8OS	0.51	8.04E-03
AURKAIP1	0.32	9.01E-04
AXIN2	1.91	3.36E-03
AZIN1	1.71	2.28E-03
B2M	0.44	1.04E-03
B3GALNT2	1.53	3.61E-03
B4GALT1	2.34	1.03E-03
B4GALT4-AS1	0.59	1.52E-03
B4GALT7	1.77	7.52E-03

Gene ID	Fold Change: AF+HF RA versus NF RA	FDR
B9D1	1.85	3.91E-04
BAALC	0.66	2.64E-02
BAG3	1.57	5.67E-03
BAHD1	1.54	1.53E-02
BAIAP2	2.54	3.45E-07
BANK1	0.60	5.67E-03
BARD1	0.65	1.19E-02
BARX2	0.65	4.62E-03
BASP1	0.50	2.24E-04
BATF	0.57	2.68E-04
BAZ2A	1.63	4.33E-03
BBOX1-AS1	0.65	3.76E-02
BBS7	0.55	8.23E-04
BBS9	0.66	2.79E-03
BCAR1	2.31	1.01E-04
BCAS1	0.62	1.22E-03
BCHE	0.37	1.37E-04
BCL2	2.03	5.00E-03
BCL2L1	1.54	8.93E-03
BCL2L13	1.87	7.99E-06
BCL2L2-PABPN1	1.52	2.64E-02
BCL7A	1.71	1.31E-03
BEND4	0.66	9.80E-03
BHLHE40-AS1	0.62	2.68E-03
BLCAP	1.76	5.57E-04
BLK	0.65	1.86E-02
BLNK	0.62	1.02E-02
BMP6	0.66	2.26E-02
BMP8A	2.09	3.36E-02
BMPER	0.59	1.12E-03
BOC	0.63	3.32E-03
BORA	0.56	1.42E-04
BPIFA4P	0.62	2.91E-02
BPIFB4	0.58	2.70E-02
BPIFB9P	1.69	3.76E-02
BPIFC	0.65	8.53E-03
BRAT1	1.64	2.28E-02
BRCA2	0.61	1.32E-03
BRD1	1.67	3.81E-04

Gene ID	Fold Change: AF+HF RA versus NF RA	FDR
BRD2	1.55	1.02E-02
BRD4	1.77	9.17E-05
BRF1	1.81	9.87E-04
BRINP1	0.67	4.55E-02
BRINP2	0.66	6.85E-03
BRINP3	0.29	1.09E-07
BSG	0.47	5.56E-03
BTBD2	1.58	2.90E-02
BTC	0.63	1.40E-02
BTF3	0.43	1.62E-04
BTG3	0.63	3.58E-03
BTG3-AS1	0.67	4.38E-02
BTLA	0.63	6.38E-03
BTN2A3P	0.57	4.64E-02
BUB1	0.63	4.33E-03
BUB1B	0.62	2.65E-03
BX322639.1	0.59	1.63E-02
BX640514.1	2.65	2.37E-02
C10orf107	0.63	3.76E-02
C10orf71	1.56	4.82E-02
C11orf24	1.81	2.70E-03
C11orf40	0.41	2.60E-04
C11orf44	0.64	1.48E-02
C11orf53	0.61	4.02E-02
C11orf70	0.61	1.48E-03
C11orf74	0.66	3.49E-02
C11orf84	1.65	1.40E-02
C12orf29	0.58	6.78E-03
C12orf42	0.57	5.71E-04
C12orf50	0.57	6.62E-04
C12orf57	0.50	3.98E-03
C13orf42	0.61	2.08E-03
C14orf2	0.63	1.41E-02
C15orf32	0.58	9.74E-03
C16orf78	0.62	3.08E-03
C16orf97	0.57	1.93E-04
C19orf12	1.88	4.20E-04
C19orf53	0.44	2.76E-06
C1orf112	0.64	1.50E-03

Gene ID	Fold Change: AF+HF RA versus NF RA	FDR
C1orf132	1.96	1.03E-03
C1orf141	0.55	4.00E-04
C1orf159	1.89	2.79E-03
C1orf198	1.63	5.39E-04
C1orf87	0.61	1.57E-03
C1QB	0.30	1.97E-05
C1QC	0.19	4.62E-07
C1QL3	0.55	7.51E-03
C1QTNF1	2.32	9.72E-03
C1R	0.25	6.17E-07
C1S	0.53	1.81E-04
C2CD6	0.65	2.60E-03
C2orf27A	0.51	7.49E-04
C2orf73	0.62	5.31E-03
C2orf80	0.61	1.03E-02
C3	0.26	6.46E-04
C3AR1	0.47	2.54E-04
C3orf70	0.67	5.17E-03
C3orf85	0.64	1.58E-02
C4BPB	0.59	3.76E-02
C4orf47	0.56	3.23E-04
C4orf51	0.64	5.90E-03
C5	0.60	7.06E-04
C5AR1	1.89	2.92E-02
C5orf67	0.62	5.23E-03
C6orf136	1.54	2.23E-02
C7	0.36	1.54E-06
C7orf26	1.58	1.13E-02
C7orf31	0.66	1.90E-03
C7orf57	0.62	2.07E-02
C7orf72	0.65	2.71E-02
C7orf77	0.54	1.04E-02
C8A	0.59	1.59E-02
C8B	0.58	4.03E-03
C8orf34	0.51	8.20E-06
C8orf34-AS1	0.50	6.41E-05
C8orf89	0.61	2.49E-02
C9orf170	0.47	1.62E-03
CA1	0.53	7.45E-05

Gene ID	Fold Change: AF+HF RA versus NF RA	FDR
CA10	0.62	1.15E-02
CA12	0.65	1.89E-02
CA3	0.61	1.35E-03
CABLES2	1.94	2.29E-02
CACHD1	0.62	3.21E-03
CACNA1C	3.89	8.77E-11
CACNB2	2.03	3.82E-04
CACNG3	0.60	2.80E-03
CADM2	0.53	1.74E-05
CAGE1	0.66	2.76E-02
CALCB	0.61	1.12E-03
CALCR	0.55	6.57E-03
CALR	0.56	7.25E-03
CALR4P	0.53	9.56E-03
CAMK2B	2.11	2.08E-04
CAMK4	0.60	1.53E-03
CAND2	1.64	1.15E-02
CANX	0.66	1.07E-02
CAPN15	2.65	2.68E-05
CAPNS1	0.67	2.81E-02
CAPSL	0.62	3.35E-03
CARD6	0.51	2.25E-03
CARHSP1	2.07	3.68E-02
CARM1	1.89	4.61E-04
CARM1P1	0.59	1.30E-03
CASC1	0.65	5.30E-03
CASC11	0.62	1.99E-03
CASC15	0.60	5.16E-04
CASC17	0.60	4.56E-03
CASC18	0.60	6.34E-03
CASC19	0.61	4.71E-03
CASC20	0.66	9.56E-03
CASC22	0.51	1.59E-03
CASP14	0.51	9.84E-03
CASP4	0.57	2.05E-03
CASP9	1.51	3.32E-02
CASR	0.58	3.32E-04
CASTOR2	1.88	5.39E-03
CASZ1	2.28	2.17E-07

Gene ID	Fold Change: AF+HF RA versus NF RA	FDR	Gene ID	Fold Change: AF+HF RA versus NF RA	FDR
CATSPERB	0.44	7.58E-09	*CD1A*	0.44	1.08E-03
CATSPERE	0.64	2.07E-03	*CD2*	0.52	2.35E-03
CBLB	1.58	5.22E-03	*CD200*	0.53	2.35E-03
CBX7	1.69	6.82E-03	*CD200R1*	0.52	5.31E-03
CBY1	2.13	1.77E-04	*CD200R1L*	0.64	2.84E-02
CC2D1A	1.57	4.33E-02	*CD209*	0.59	3.63E-02
CC2D1B	1.66	6.76E-04	*CD226*	0.64	2.89E-02
CCAR1	1.54	4.13E-03	*CD244*	0.66	1.61E-02
CCDC102B	0.61	1.72E-03	*CD276*	2.18	1.39E-02
CCDC110	0.61	2.79E-02	*CD28*	0.59	2.42E-03
CCDC12	1.67	6.35E-04	*CD33*	0.60	5.46E-03
CCDC130	1.63	1.21E-03	*CD34*	0.43	1.16E-04
CCDC144CP	0.59	4.33E-03	*CD38*	0.50	1.62E-04
CCDC148	0.52	1.71E-04	*CD44*	0.52	1.89E-03
CCDC158	0.61	4.83E-03	*CD53*	0.49	7.82E-04
CCDC170	0.63	1.69E-03	*CD63*	0.52	1.88E-02
CCDC173	0.60	5.60E-03	*CD86*	0.55	1.85E-04
CCDC190	0.52	1.89E-03	*CD93*	0.59	4.81E-02
CCDC191	0.60	1.72E-04	*CDAN1*	1.77	1.05E-03
CCDC192	0.64	1.99E-02	*CDC14B*	1.52	5.25E-03
CCDC195	0.65	4.24E-02	*CDC20B*	0.56	1.38E-04
CCDC26	0.61	2.90E-03	*CDC42BPB*	1.61	1.24E-03
CCDC34	0.66	1.44E-02	*CDC42EP4*	2.00	7.12E-06
CCDC39	0.64	1.03E-02	*CDCA7*	0.59	1.22E-02
CCDC57	1.59	8.93E-03	*CDCP1*	0.66	8.18E-03
CCDC59	0.60	2.30E-03	*CDH10*	0.54	4.68E-02
CCDC68	0.60	3.05E-03	*CDH18*	0.64	2.81E-02
CCDC73	0.66	6.10E-03	*CDH19*	0.43	8.56E-03
CCDC83	0.57	2.73E-03	*CDH5*	0.61	1.04E-02
CCDC84	1.83	3.76E-03	*CDH6*	0.60	1.32E-02
CCDC9	1.72	5.90E-03	*CDH7*	0.63	2.85E-02
CCDC92	1.62	5.72E-04	*CDH9*	0.64	4.64E-02
CCER1	0.62	3.85E-02	*CDHR3*	0.64	1.51E-03
CCND3	1.57	3.67E-02	*CDIP1*	1.71	2.36E-03
CCNYL2	0.66	5.68E-03	*CDK5RAP1*	1.51	1.16E-02
CCR2	0.59	2.88E-02	*CDKN1A*	2.18	9.05E-03
CD101	0.64	3.29E-02	*CDO1*	0.56	1.25E-02
CD163	0.33	1.01E-05	*CDON*	0.64	1.58E-02
CD180	0.63	2.72E-02	*CDS1*	0.57	9.42E-04

Gene ID	Fold Change: AF+HF RA versus NF RA	FDR	Gene ID	Fold Change: AF+HF RA versus NF RA	FDR
CECR2	1.76	3.03E-03	*CHST3*	2.60	1.16E-09
CENPE	0.61	1.22E-02	*CIZ1*	1.58	8.33E-03
CENPF	0.66	1.28E-02	*CKB*	0.26	1.14E-06
CENPQ	0.62	2.05E-03	*CKM*	0.31	4.44E-08
CEP126	0.62	6.88E-03	*CLASRP*	1.70	4.33E-03
CEP128	0.60	1.37E-03	*CLCA2*	0.66	3.55E-02
CEP135	0.66	5.91E-03	*CLCN3P1*	0.63	2.10E-03
CEP152	0.60	3.96E-03	*CLCN6*	1.74	6.23E-05
CEP162	0.64	3.92E-03	*CLDN11*	0.64	1.22E-02
CEP170P1	0.60	1.10E-03	*CLDN16*	0.63	2.44E-02
CEP290	0.59	1.15E-03	*CLEC12A*	0.58	8.84E-03
CEP78	0.61	2.19E-03	*CLEC16A*	1.85	5.45E-04
CERK	2.21	2.91E-05	*CLEC19A*	0.64	2.87E-02
CERKL	0.60	2.59E-02	*CLEC1A*	0.62	2.92E-03
CERS3-AS1	0.63	2.90E-02	*CLEC1B*	0.62	3.08E-02
CERS4	1.73	2.36E-02	*CLEC2A*	0.64	1.78E-02
CES1P2	0.55	1.40E-03	*CLEC6A*	0.62	1.39E-02
CES2	1.73	2.03E-02	*CLEC7A*	0.36	4.06E-08
CES5AP1	0.66	4.26E-02	*CLEC9A*	0.61	7.13E-03
CFAP43	0.56	5.67E-04	*CLECL1*	0.60	4.09E-02
CFD	0.10	1.89E-10	*CLIC1*	0.63	7.02E-03
CFH	0.39	8.58E-05	*CLIC5*	1.52	4.19E-02
CFHR5	0.60	9.13E-03	*CLK1*	1.78	2.72E-03
CFI	0.55	6.63E-05	*CLK3*	1.63	3.35E-03
CFLAR	1.55	5.84E-03	*CLMAT3*	0.56	5.33E-04
CFTR	0.64	1.51E-02	*CLMP*	0.55	8.26E-04
CGNL1	0.50	1.38E-03	*CLPTM1L*	1.61	8.74E-03
CHCHD4	1.56	3.73E-02	*CLSPN*	0.65	2.55E-02
CHIAP3	0.56	5.45E-03	*CLTB*	2.39	4.93E-07
CHID1	1.52	2.36E-02	*CLTCL1*	2.05	1.19E-03
CHL1	0.53	1.59E-02	*CLU*	0.50	2.96E-02
CHMP2A	0.61	2.21E-02	*CMAHP*	0.49	9.47E-05
CHMP3	0.62	8.16E-03	*CMPK2*	0.63	9.54E-03
CHODL	0.63	5.36E-03	*CMYA5*	1.99	7.63E-03
CHODL-AS1	0.61	1.68E-03	*CNBD1*	0.59	1.87E-03
CHPF2	1.76	1.37E-02	*CNOT3*	1.68	6.38E-03
CHPT1	1.70	2.24E-03	*CNOT9*	1.51	6.12E-03
CHRM3	0.58	6.01E-04	*CNRIP1*	0.55	1.17E-02
CHST11	1.55	4.57E-02	*CNTN1*	0.58	3.52E-03

Gene ID	Fold Change: AF+HF RA versus NF RA	FDR
CNTN3	0.54	1.08E-04
CNTN4	0.64	9.56E-03
CNTN6	0.57	1.47E-02
CNTNAP2	0.63	5.97E-03
CNTNAP3	0.57	1.73E-02
CNTNAP3B	0.52	2.45E-03
CNTNAP3P2	0.53	5.77E-03
CNTNAP4	0.64	1.57E-02
CNTNAP5	0.61	1.75E-03
COA3	0.46	3.12E-04
COBLL1	0.49	5.08E-04
COG8	1.60	3.44E-02
COL11A1	0.63	3.05E-02
COL14A1	0.66	1.71E-02
COL15A1	0.58	4.69E-03
COL18A1	1.76	1.02E-02
COL19A1	0.43	2.51E-03
COL1A2	0.51	1.26E-02
COL24A1	0.57	3.68E-04
COL28A1	0.54	1.37E-03
COL3A1	0.41	1.28E-02
COL4A1	1.61	1.33E-02
COL4A2	1.51	3.80E-02
COL4A4	0.66	3.16E-02
COL5A2	0.56	9.42E-03
COL6A3	0.59	7.61E-03
COL6A4P2	0.52	8.37E-05
COL6A5	0.60	4.73E-03
COL8A1	0.40	3.40E-02
COL9A1	0.48	4.80E-02
COLGALT2	0.62	3.56E-04
COLQ	2.07	5.29E-03
COMMD5	2.11	6.56E-04
COMMD7	1.55	8.56E-03
COMT	2.37	1.96E-05
COPS9	0.58	1.03E-02
COPZ2	1.66	2.50E-02
COQ4	2.03	1.47E-05
CORIN	0.58	3.95E-02

Gene ID	Fold Change: AF+HF RA versus NF RA	FDR
COX10-AS1	0.60	7.25E-03
COX19	1.66	7.57E-03
COX5B	0.59	2.18E-03
COX6A2	0.34	6.93E-05
COX6B1	0.63	2.04E-03
COX7A1	0.21	5.82E-11
COX7A2	0.59	9.18E-03
COX7B2	0.63	2.04E-02
COX7C	0.33	1.85E-07
CP	0.55	5.33E-04
CPA2	0.58	5.62E-03
CPA6	0.65	2.75E-03
CPB2-AS1	0.60	1.34E-02
CPE	0.52	5.51E-03
CPEB2-AS1	0.60	2.09E-03
CPEB4	2.61	4.09E-06
CPHL1P	0.60	2.42E-03
CPLX4	0.65	3.26E-02
CPNE1	1.83	2.44E-04
CPNE5	2.64	4.59E-04
CPS1	0.58	1.60E-03
CPSF7	1.52	4.23E-03
CR1	0.61	1.47E-02
CR1L	0.58	2.85E-04
CR2	0.61	2.98E-03
CR392039.3	0.65	1.38E-02
CR589904.1	0.53	4.72E-05
CRAMP1	1.80	2.49E-03
CRAT37	0.59	2.06E-02
CRB1	0.52	3.32E-04
CREB3	2.02	5.42E-04
CRIP2	0.35	2.54E-07
CRISPLD1	0.54	1.58E-03
CRK	1.81	3.92E-05
CRLF3	1.51	1.71E-02
CROCCP2	2.36	3.23E-04
CROT	0.64	2.10E-03
CRTC1	1.74	1.61E-03
CRTC2	1.70	4.90E-03

Gene ID	Fold Change: AF+HF RA versus NF RA	FDR	Gene ID	Fold Change: AF+HF RA versus NF RA	FDR
CRTC3	1.57	3.54E-03	*CXCL13*	0.66	4.25E-03
CRY2	1.61	1.35E-03	*CXXC4*	0.55	7.33E-04
CRYAB	0.61	4.94E-02	*CXXC4-AS1*	0.58	1.58E-04
CRYBB1	0.52	1.33E-02	*CYB5R1*	1.66	6.54E-03
CSAD	1.64	1.05E-03	*CYB5R2*	1.57	2.30E-02
CSDC2	2.55	6.81E-05	*CYBRD1*	0.50	1.17E-06
CSF1R	0.65	9.90E-03	*CYC1*	0.49	8.63E-04
CSMD1	0.61	7.92E-03	*CYHR1*	2.13	2.63E-03
CSMD3	0.60	2.17E-02	*CYLC2*	0.56	4.67E-02
CSNK1D	2.15	6.58E-05	*CYP19A1*	0.65	1.44E-02
CSNK1G2	1.72	1.96E-03	*CYP1B1-AS1*	0.56	3.40E-05
CSPG4	3.88	7.94E-09	*CYP24A1*	0.62	3.10E-03
CSRNP2	1.55	2.46E-03	*CYP2C8*	0.61	1.29E-02
CSRNP3	0.62	3.43E-03	*CYP4F9P*	0.55	1.48E-02
CST3	0.50	1.20E-02	*CYP7B1*	0.58	2.94E-04
CSTB	0.45	5.40E-04	*CYSLTR2*	0.47	2.45E-07
CTBP1	2.53	3.62E-06	*CYTIP*	0.65	2.16E-02
CTBP2	1.76	5.22E-04	*D21S2088E*	0.59	3.11E-02
CTDP1	1.55	4.31E-03	*D2HGDH*	1.54	4.54E-02
CTDSP2	1.60	3.08E-03	*DAB1*	0.63	2.49E-02
CTHRC1	2.09	9.90E-04	*DAB2*	0.64	3.26E-02
CTIF	1.61	1.81E-02	*DAB2IP*	1.62	9.45E-03
CTNNA2	0.64	8.65E-03	*DACH1*	0.52	5.82E-03
CTNNBIP1	1.67	4.06E-03	*DAP3*	1.52	3.56E-03
CTNS	1.77	4.90E-03	*DAP3P1*	1.69	1.17E-02
CTSC	0.57	1.33E-02	*DAPK2*	2.17	4.25E-03
CTSE	0.52	4.05E-03	*DAPK3*	1.84	1.12E-03
CTSL3P	0.54	6.33E-03	*DAPL1*	0.64	3.75E-03
CTSO	0.47	3.38E-05	*DAZAP1*	2.21	6.31E-06
CTSV	0.62	3.41E-02	*DAZAP2*	0.66	8.09E-03
CTSZ	1.73	5.90E-03	*DAZL*	0.66	6.73E-03
CTTN	1.71	2.40E-03	*DBI*	0.45	1.83E-05
CUBN	0.61	1.60E-03	*DBNL*	1.81	3.12E-04
CUBNP2	0.63	1.89E-02	*DBX2*	0.59	6.03E-03
CUEDC1	1.66	1.21E-03	*DCC*	0.63	1.66E-02
CUTA	0.54	4.41E-03	*DCDC1*	0.62	5.27E-03
CUX1	1.81	2.17E-04	*DCDC2*	0.63	2.80E-03
CWH43	0.64	4.92E-03	*DCLK1*	0.53	5.66E-05
CXCL12	0.47	3.16E-03	*DCLK3*	0.64	4.43E-02

Gene ID	Fold Change: AF+HF RA versus NF RA	FDR
DCN	0.26	6.17E-07
DCP1B	0.65	3.62E-02
DCT	0.66	2.60E-02
DCUN1D2	1.81	9.90E-04
DDC	0.59	1.35E-02
DDTL	2.68	9.82E-04
DDX17	1.80	2.20E-04
DDX43	0.63	3.67E-02
DDX5	1.61	3.05E-03
DDX56	1.98	8.16E-05
DDX60	0.63	2.89E-03
DDX60L	0.64	2.80E-03
DEAF1	1.72	1.25E-02
DEC1	0.65	7.82E-03
DEFB125	0.58	1.55E-02
DENND1B	0.62	4.80E-04
DEPDC1	0.62	3.50E-02
DEPDC1-AS1	0.66	1.36E-02
DGCR2	1.54	6.41E-03
DGCR8	1.68	1.42E-03
DGKB	0.56	6.94E-03
DGKD	1.55	9.60E-03
DGKG	2.50	4.50E-04
DGKZ	1.85	7.84E-03
DHCR24	0.62	2.20E-02
DHFR2	0.52	1.24E-04
DHODH	1.56	9.56E-03
DHRS4-AS1	0.59	2.07E-03
DHX30	1.67	9.72E-04
DIDO1	1.79	5.00E-05
DIO2-AS1	0.54	8.26E-05
DIP2C	1.51	1.34E-02
DIRAS2	0.58	1.91E-03
DISP1	0.66	1.63E-02
DKK2	0.64	9.53E-03
DLEU1	0.66	3.98E-03
DLEU7	0.60	1.74E-03
DLG2	0.66	1.15E-02
DLG5	1.60	3.13E-03

Gene ID	Fold Change: AF+HF RA versus NF RA	FDR
DLGAP4	1.53	6.32E-03
DLGAP5	0.61	3.13E-03
DMAC1	0.54	6.23E-05
DMBT1P1	0.63	2.95E-03
DMRT1	0.62	2.57E-03
DMWD	1.80	2.26E-03
DNAH1	1.72	3.00E-03
DNAH5	0.62	1.25E-03
DNAH6	0.66	2.59E-02
DNAH7	0.64	6.16E-03
DNAH8	0.63	6.18E-03
DNAH9	0.65	5.52E-03
DNAJA4	2.20	1.07E-04
DNAJB12	1.80	1.06E-04
DNAJB6	1.80	2.43E-04
DNAJC17	1.66	1.26E-03
DNAJC5	1.84	8.76E-05
DNASE1L3	0.58	1.50E-02
DNASE2B	0.55	3.04E-04
DNM2	2.31	6.34E-06
DNMT3A	1.56	4.06E-03
DNTT	0.60	3.51E-03
DOCK10	0.59	6.46E-04
DOCK2	0.63	2.10E-03
DOCK5	1.86	3.32E-03
DOCK7	0.64	1.32E-03
DOCK8	0.52	5.45E-05
DOK6	0.57	6.46E-04
DOK7	2.29	4.24E-04
DOT1L	1.89	1.68E-04
DPF2	1.51	8.73E-03
DPF3	1.92	9.90E-04
DPH1	1.92	3.59E-03
DPH7	1.85	5.42E-04
DPP10	0.64	1.86E-02
DPP3P1	0.40	3.26E-02
DPP9	1.58	1.32E-02
DPPA2	0.64	1.70E-02
DPPA4	0.62	2.31E-02

Gene ID	Fold Change: AF+HF RA versus NF RA	FDR
DPT	0.57	6.85E-04
DPY19L2P3	0.54	7.39E-05
DPYD	0.61	4.02E-03
DPYS	0.66	6.62E-03
DPYSL5	0.65	9.74E-03
DRAM1	0.65	3.27E-03
DRD3	0.61	1.39E-03
DSCAM	0.62	4.50E-03
DSCR4	0.61	2.86E-03
DSE	0.65	5.41E-03
DSG1	0.47	9.77E-04
DSG3	0.55	1.96E-03
DSP	0.65	2.21E-02
DTHD1	0.51	4.17E-03
DTL	0.58	2.91E-03
DTX3L	0.59	3.09E-03
DTX4	0.61	2.15E-03
DTYMK	1.89	5.66E-03
DUBR	0.65	3.76E-02
DUSP6	2.32	3.39E-02
DUSP8	2.04	2.49E-03
DUX4	0.66	4.42E-02
DUXA	0.62	1.32E-02
DYNC2H1	0.50	5.78E-06
DYRK4	0.64	1.74E-02
DYSF	1.65	1.70E-03
DZIP1	0.58	3.05E-03
E2F4	1.51	3.35E-02
E2F7	0.66	7.02E-03
EAF2	0.60	4.25E-04
EBF1	0.45	1.79E-05
EBF2	0.54	8.67E-05
EBF3	0.62	2.11E-03
ECE1	2.11	8.37E-05
ECH1	0.43	3.35E-05
ECHDC2	1.60	2.57E-02
ECHDC3	1.96	6.41E-05
ECHS1	0.64	3.67E-02
ECI1	1.69	7.48E-03

Gene ID	Fold Change: AF+HF RA versus NF RA	FDR
ECSIT	1.51	1.66E-02
ECT2	0.61	7.82E-03
EDIL3	0.53	1.12E-03
EDNRA	2.05	6.85E-04
EDNRB	0.46	3.32E-04
EEF1A2	0.57	8.68E-03
EEF1D	2.23	1.47E-04
EEF2	0.51	2.87E-03
EFEMP1	0.39	2.73E-06
EFNB2	0.57	2.79E-03
EGFEM1P	0.54	6.06E-03
EGFR	1.83	1.67E-02
EGR1	4.12	1.63E-02
EHBP1	0.66	4.30E-03
EHD2	0.60	3.14E-02
EHHADH-AS1	0.58	1.38E-02
EHMT1	1.50	5.22E-03
EIF1	0.38	1.58E-05
EIF1B	1.69	7.23E-03
EIF4EBP2	1.64	3.59E-03
EIF5A	1.74	2.04E-02
EIF5A2	0.63	4.39E-03
ELAVL1	1.63	1.43E-03
ELF5	0.67	3.03E-02
ELL	1.92	5.74E-04
ELL2	3.42	5.34E-05
ELOB	0.59	3.11E-02
ELOVL2	0.63	3.31E-03
ELOVL2-AS1	0.66	1.17E-02
ELOVL7	0.66	8.18E-03
ELSPBP1	0.66	4.64E-02
EMB	0.59	8.20E-03
EMBP1	0.56	7.33E-04
EMC1	1.64	9.23E-03
EMC10	6.02	9.16E-04
EMC4	0.66	1.77E-02
EMCN	0.50	5.11E-04
ENAM	0.63	1.12E-02
ENC1	0.48	7.27E-05

Gene ID	Fold Change: AF+HF RA versus NF RA	FDR
ENDOD1	0.62	2.19E-03
ENG	0.53	9.42E-04
ENKUR	0.56	3.38E-04
ENO4	0.63	3.32E-03
ENPP2	0.58	5.88E-04
ENPP3	0.64	1.55E-02
ENPP7P5	0.62	8.84E-03
ENTPD1	0.51	5.76E-05
ENTPD6	1.54	1.55E-03
ENY2	0.61	4.14E-03
EP400	1.53	2.76E-03
EP400NL	1.76	2.71E-03
EPB41L2	0.49	3.21E-07
EPHA1-AS1	0.63	8.53E-04
EPHA3	0.56	3.92E-02
EPHA6	0.64	3.07E-02
EPHX4	0.63	2.35E-02
EPN1	1.57	1.11E-02
EPN2	2.18	3.73E-06
EPS15L1	2.65	5.02E-07
EPSTI1	0.58	1.04E-03
EPYC	0.63	4.54E-02
ERG	0.62	3.59E-04
ERGIC1	1.53	3.83E-03
ERICH3	0.57	1.15E-02
ERICH6	0.66	8.47E-03
ERP27	0.66	3.91E-02
ERVK13-1	1.87	5.28E-04
ESR1	0.58	1.86E-04
ESRP1	0.62	8.83E-03
ESRRA	2.38	2.20E-04
ETS1	0.58	5.08E-04
EVA1A	0.64	3.15E-03
EVA1C	2.16	5.78E-06
EXOC3	1.63	1.08E-03
EXOC6B	1.52	1.48E-02
EXOSC1	1.74	3.06E-03
EXOSC9	0.65	2.33E-02
EXPH5	0.60	8.59E-03

Gene ID	Fold Change: AF+HF RA versus NF RA	FDR
EXTL2	0.63	1.24E-03
EYA1	0.60	2.54E-02
EYS	0.66	4.34E-02
EZH1	1.50	5.43E-03
EZR	1.76	1.80E-03
F10	1.61	7.79E-03
F11	0.43	1.21E-04
F11-AS1	0.66	5.06E-03
F13A1	0.42	2.10E-07
F2R	0.60	4.21E-02
FABP3	0.66	3.48E-02
FADS2P1	0.60	5.53E-03
FADS3	1.52	2.52E-02
FAIM2	0.53	2.88E-02
FAM102A	1.59	2.20E-02
FAM102B	0.64	9.10E-03
FAM105A	0.61	4.63E-03
FAM107A	0.66	4.82E-02
FAM107B	0.65	9.95E-03
FAM111A	0.56	1.21E-03
FAM118A	1.72	1.28E-02
FAM120B	1.65	9.17E-04
FAM124B	0.54	4.59E-03
FAM131A	1.84	3.51E-03
FAM135B	0.64	6.96E-03
FAM13C	0.64	1.18E-02
FAM155A	0.65	1.51E-02
FAM160B2	1.58	1.32E-02
FAM168B	1.65	2.01E-04
FAM171B	0.65	1.88E-02
FAM177B	0.59	3.86E-03
FAM184A	0.64	2.83E-03
FAM193B	1.52	1.33E-02
FAM198B	0.46	5.01E-09
FAM19A1	0.53	5.06E-04
FAM19A2	0.59	6.35E-04
FAM19A4	0.65	4.57E-03
FAM207A	1.60	3.49E-02
FAM20C	1.61	4.50E-03

Gene ID	Fold Change: AF+HF RA versus NF RA	FDR
FAM212B	2.35	1.31E-04
FAM216B	0.46	1.58E-02
FAM219A	1.57	4.02E-03
FAM219B	1.81	1.21E-02
FAM229B	0.56	1.18E-04
FAM234A	1.90	1.91E-03
FAM26D	0.51	2.10E-03
FAM71F1	0.63	5.96E-03
FAM71F2	1.71	1.27E-02
FAM78B	1.90	1.99E-02
FAM81B	0.54	3.11E-02
FAM83A	0.60	1.05E-02
FAM83B	0.57	2.50E-02
FAM86DP	2.19	5.57E-03
FAR2	0.63	1.45E-03
FARP2	1.55	4.94E-03
FAS	0.62	3.49E-02
FASN	0.11	6.27E-04
FAT4	0.62	5.35E-03
FAU	0.39	2.62E-03
FBLIM1	1.54	2.83E-02
FBLN1	0.48	1.88E-03
FBLN2	0.59	7.84E-03
FBLN5	0.48	3.56E-06
FBN1	0.50	4.78E-04
FBP2P1	0.60	3.30E-02
FBRSL1	2.63	8.70E-10
FBXL12	1.55	2.79E-02
FBXO18	1.77	5.99E-04
FBXO21	1.58	4.50E-03
FBXO31	1.65	7.21E-04
FBXO34	1.72	1.32E-04
FBXO47	0.59	1.20E-02
FBXW4	1.72	6.39E-04
FCGBP	0.58	1.10E-02
FCMR	0.61	2.72E-02
FCRL1	0.56	1.36E-03
FCRL2	0.54	5.92E-04
FCRL4	0.59	5.28E-03

Gene ID	Fold Change: AF+HF RA versus NF RA	FDR
FER1L6	0.64	5.76E-03
FER1L6-AS2	0.60	8.96E-04
FGD4	1.87	6.41E-03
FGF10	0.52	1.21E-03
FGF2	0.54	1.45E-02
FGF5	0.65	2.52E-02
FGL1	0.58	3.35E-03
FH	0.61	7.30E-03
FHOD1	1.83	3.03E-02
FITM1	0.51	3.30E-02
FKBP5	3.36	6.58E-07
FKRP	1.51	1.24E-02
FLI1	0.55	2.97E-05
FLJ37453	1.55	2.36E-02
FLJ46284	0.53	1.91E-02
FLRT2	0.48	1.69E-03
FLT1	0.55	6.46E-04
FLT3	0.65	3.04E-03
FLYWCH1	1.54	2.14E-02
FMN1	0.60	7.17E-03
FMN2	0.66	8.24E-03
FMO11P	0.62	8.88E-03
FMO2	0.42	5.01E-09
FMO3	0.66	3.57E-02
FMO6P	0.62	4.65E-02
FMO8P	0.52	1.10E-03
FMOD	0.56	4.37E-02
FN3K	1.71	1.03E-02
FNDC3B	1.63	2.24E-03
FNDC7	0.65	3.39E-02
FNIP2	2.22	1.55E-04
FO393415.1	0.61	2.41E-02
FO393415.3	0.65	1.62E-02
FOLH1	0.62	7.61E-03
FOSL2	2.60	1.25E-03
FOXB1	0.64	6.26E-03
FOXG1-AS1	0.61	1.67E-02
FOXK1	1.79	1.12E-03
FOXK2	2.06	1.27E-06

Gene ID	Fold Change: AF+HF RA versus NF RA	FDR	Gene ID	Fold Change: AF+HF RA versus NF RA	FDR
FOXO3	1.88	3.32E-04	*GALNT15*	0.60	1.15E-02
FOXP4	1.86	3.59E-04	*GALNT3*	0.59	5.86E-03
FOXRED1	1.51	3.65E-02	*GALNT8*	0.64	1.73E-02
FP671120.4	0.42	1.39E-03	*GALNTL6*	0.62	1.34E-02
FPGS	1.80	8.98E-03	*GALR1*	0.54	2.17E-04
FREM1	0.47	5.28E-06	*GAPDH*	0.29	1.04E-08
FREM2	0.66	3.22E-02	*GAPDHP63*	0.26	2.11E-05
FRK	0.50	5.08E-04	*GAR1*	0.59	6.16E-04
FRMD6	0.65	7.51E-03	*GAS2*	0.65	6.65E-03
FRMD6-AS2	0.59	6.46E-04	*GAS2L3*	0.66	3.92E-02
FRMD8	2.03	3.03E-04	*GAS6*	3.04	1.23E-05
FRRS1	0.61	7.78E-04	*GATA4*	1.59	1.69E-03
FRZB	0.58	1.61E-03	*GATA6*	2.11	2.45E-04
FSIP1	0.65	2.43E-03	*GATAD2A*	1.70	3.13E-03
FSIP2	0.47	2.61E-06	*GATAD2B*	1.70	1.23E-03
FST	0.61	3.92E-02	*GBA2*	2.07	3.32E-06
FSTL1	2.98	2.09E-05	*GBF1*	1.77	6.92E-05
FSTL5	0.62	3.29E-02	*GBP1P1*	0.56	1.33E-02
FTL	0.48	2.04E-03	*GBP2*	0.46	1.77E-04
FUBP3	1.96	1.23E-05	*GBP3*	0.53	7.33E-04
FUK	1.86	2.77E-03	*GBP4*	0.50	1.93E-04
FUS	1.52	1.38E-02	*GBP6*	0.61	6.86E-03
FUT9	0.62	2.17E-02	*GC*	0.61	2.89E-02
FXR2	1.56	6.14E-03	*GCA*	0.51	2.07E-05
FYB1	0.51	9.36E-05	*GCM1*	0.57	4.71E-03
FYTTD1	1.68	1.18E-03	*GDAP1L1*	3.01	5.23E-07
FZR1	2.04	1.63E-04	*GFOD1*	1.79	2.04E-03
G6PC	0.59	2.80E-02	*GFRA1*	0.59	2.14E-04
GABARAPL1	1.73	2.65E-02	*GFRAL*	0.60	1.14E-02
GABPB2	2.01	3.11E-05	*GGA1*	1.64	6.81E-03
GABRA2	0.58	3.92E-03	*GGT7*	1.61	1.21E-02
GABRG3	0.66	1.48E-02	*GIGYF1*	1.86	2.02E-02
GABRR3	0.63	1.99E-02	*GIMAP2*	0.48	1.15E-04
GACAT1	0.67	9.21E-03	*GIMAP4*	0.44	4.07E-06
GAD2	0.56	1.59E-03	*GIMAP6*	0.60	1.60E-02
GADL1	0.59	1.04E-02	*GIMAP8*	0.65	2.53E-02
GAK	1.86	5.90E-04	*GIPC1*	1.71	2.14E-02
GALNS	1.73	2.26E-03	*GLCE*	0.61	3.06E-03
GALNT13	0.62	1.16E-02	*GLIPR1L2*	0.49	8.20E-06

Gene ID	Fold Change: AF+HF RA versus NF RA	FDR
GLIS3	0.56	3.12E-04
GLRA3	0.60	5.25E-03
GLRX5	0.65	2.67E-02
GLS2	1.91	2.97E-02
GLUD1P3	1.77	1.63E-02
GLUL	0.58	3.24E-02
GMFB	0.62	1.48E-02
GMNC	0.56	6.40E-03
GMPR	1.77	2.36E-04
GNA14	0.58	4.82E-05
GNAI1	0.60	3.36E-03
GNAQ	0.52	4.19E-05
GNAS	1.82	1.07E-02
GNB3	2.37	6.47E-03
GNG2	0.61	2.13E-03
GNG7	1.76	7.82E-03
GOLGA4	1.53	4.95E-03
GOLGA8A	1.56	2.41E-02
GORASP1	1.60	2.36E-02
GPAM	0.44	6.78E-03
GPAT3	2.71	9.44E-04
GPATCH4	1.58	3.50E-03
GPATCH8	1.69	5.86E-03
GPC6	0.65	3.39E-02
GPD1L	1.68	4.85E-03
GPD2	0.60	1.31E-04
GPI	1.52	2.01E-02
GPM6A	0.58	1.76E-02
GPNMB	0.44	7.36E-04
GPR139	0.58	1.52E-03
GPR149	0.61	2.97E-03
GPR157	1.51	3.53E-02
GPR158	0.66	1.23E-02
GPR37	0.63	3.82E-02
GPR39	0.66	1.08E-02
GPR63	0.62	8.83E-03
GPR83	0.59	1.50E-02
GPX1	0.19	9.09E-10
GPX3	0.28	5.01E-09

Gene ID	Fold Change: AF+HF RA versus NF RA	FDR
GPX6	0.56	9.45E-04
GPX7	1.61	2.41E-02
GRAMD1A	1.51	3.13E-02
GRAMD1C	0.44	2.63E-07
GRAMD4	2.12	3.03E-04
GRB14	0.57	1.98E-02
GREB1L	2.20	1.04E-05
GRHL2	0.65	3.19E-03
GRIA2	0.60	1.34E-02
GRIA4	0.58	2.52E-03
GRID2	0.55	2.24E-03
GRIK2	0.58	1.47E-02
GRIN2B	0.61	7.68E-03
GRIN3A	0.63	6.38E-03
GRIP2	1.76	4.67E-03
GRK4	1.61	5.43E-03
GRM1	0.59	2.05E-03
GRM3	0.53	8.24E-05
GRM5	0.59	3.89E-03
GRM7	0.61	7.00E-03
GRN	0.56	8.83E-03
GRXCR1	0.59	4.28E-03
GSDMB	1.87	6.33E-03
GSDMC	0.61	4.83E-02
GSE1	2.09	1.85E-03
GSTA1	0.53	1.52E-03
GSTA10P	0.53	7.92E-03
GSTA2	0.50	1.12E-03
GSTA3	0.54	2.25E-03
GSTK1	0.62	1.18E-02
GTF2H2	0.62	2.06E-02
GTF2I	1.74	1.16E-03
GTF2IP2	0.62	3.04E-02
GTF2IP20	1.52	3.63E-02
GTF2IRD1	4.36	8.30E-11
GTF2IRD2	2.56	5.39E-04
GTF2IRD2B	2.00	2.86E-03
GTF3C1	1.59	6.22E-04
GTPBP1	1.75	3.05E-04

Gene ID	Fold Change: AF+HF RA versus NF RA	FDR
GTPBP2	1.62	2.23E-03
GUCY1A2	0.59	7.69E-03
GUCY1A3	0.57	3.17E-02
GUCY1B2	0.59	1.84E-02
GUCY1B3	0.57	8.53E-03
GULP1	0.52	2.05E-04
GUSBP1	0.66	7.68E-03
GVINP1	0.60	1.79E-03
H19	0.25	4.52E-06
H1F0	0.37	3.40E-05
H2AFJ	0.63	1.25E-02
H2AFZ	0.37	5.26E-05
H6PD	1.83	2.82E-03
HACD1	1.84	1.40E-04
HACD4	0.66	9.06E-03
HAGH	1.85	1.28E-03
HAL	0.56	3.84E-03
HAO1	0.56	1.63E-03
HAPLN1	0.57	9.99E-03
HAS2	0.48	2.09E-03
HAVCR1	0.56	1.01E-03
HAX1	0.61	3.21E-03
HCRTR2	0.64	1.73E-02
HDAC11	1.61	1.75E-02
HDAC2-AS2	0.63	9.62E-03
HDAC4	1.94	1.07E-04
HDAC5	1.95	8.26E-05
HDAC7	2.05	6.79E-05
HDGFL2	1.78	9.20E-03
HDHD3	1.97	6.08E-03
HDHD5	1.65	2.91E-02
HEATR4	1.63	2.86E-03
HECW1	0.63	2.89E-03
HEG1	2.12	3.91E-05
HELLPAR	0.63	1.58E-02
HELLS	0.66	1.50E-02
HEMGN	0.65	1.24E-02
HEMK1	1.58	3.39E-02
HENMT1	0.59	2.76E-02
HEPACAM2	0.57	2.20E-02
HEPHL1	0.62	1.18E-03
HERC2P10	0.59	5.35E-03
HERC2P4	0.53	4.42E-02
HERC5	0.61	1.76E-02
HGD	0.63	1.22E-02
HGF	0.62	4.92E-02
HHIPL2	0.54	6.73E-03
HHLA1	0.60	1.99E-02
HHLA2	0.64	2.18E-03
HIF3A	3.39	4.09E-06
HINT1	0.58	3.55E-03
HIPK2	2.23	2.74E-06
HIST1H1C	0.19	8.22E-09
HIST1H1E	0.27	8.30E-04
HIST1H2BC	0.55	2.53E-02
HIST1H2BD	0.51	1.70E-02
HIST1H4C	0.38	6.08E-05
HIST1H4D	0.31	1.82E-05
HIST1H4H	0.61	4.67E-03
HIST2H2AC	0.34	1.16E-04
HKR1	1.59	2.19E-03
HLA-C	0.33	7.79E-05
HLA-DPA1	0.55	2.24E-02
HLA-DPA3	0.50	2.91E-03
HLA-DQB2	0.56	1.27E-02
HLA-DRA	0.58	1.75E-02
HLA-DRB1	0.44	2.26E-03
HLA-E	0.42	6.79E-04
HLX-AS1	0.64	3.04E-02
HM13	1.72	8.56E-04
HMCN1	0.51	7.84E-04
HMGCLL1	0.48	1.43E-03
HMGCS2	0.33	4.26E-05
HMMR	0.62	2.25E-02
HMSD	0.56	2.21E-03
HNF4G	0.63	9.11E-03
HNMT	0.57	1.19E-02
HNRNPA2B1	1.54	9.16E-03

Gene ID	Fold Change: AF+HF RA versus NF RA	FDR
HNRNPC	1.52	1.72E-03
HNRNPF	1.52	9.57E-03
HNRNPH1	1.66	2.26E-03
HNRNPKP3	0.64	9.20E-03
HNRNPL	2.00	2.17E-04
HOOK1	0.56	1.88E-02
HOOK2	1.96	1.45E-02
HORMAD2	0.59	1.83E-03
HPS3	0.66	1.67E-02
HPSE	0.61	1.48E-02
HRASLS5	0.58	5.30E-04
HRC	0.53	2.46E-03
HS3ST4	0.62	4.05E-03
HS6ST1	1.63	1.71E-03
HSD17B7	0.63	1.01E-02
HSD17B7P2	0.64	3.19E-02
HSD3BP4	0.52	3.91E-03
HSF1	1.61	1.40E-03
HSP90AA1	0.51	8.37E-05
HSP90AB1	0.62	1.59E-02
HSP90AB2P	1.61	6.87E-03
HSPA13	0.61	4.78E-02
HSPA2	0.44	1.01E-02
HSPA4L	0.66	3.45E-02
HSPA5	0.49	7.82E-03
HSPA8	0.52	1.12E-02
HSPB1	0.55	2.36E-02
HSPB3	0.62	1.20E-02
IBSP	0.54	4.60E-03
ICOS	0.60	4.08E-03
ID1	0.20	8.20E-06
IFI16	0.49	9.56E-04
IFI27L2	0.30	2.38E-04
IFI44	0.48	7.05E-03
IFI44L	0.51	3.81E-02
IFI6	0.22	1.29E-02
IFIH1	0.47	5.21E-04
IFITM2	0.29	2.51E-05
IFT122	1.83	6.72E-04

Gene ID	Fold Change: AF+HF RA versus NF RA	FDR
IFT57	0.63	7.45E-03
IGF1	0.45	2.02E-03
IGF1R	2.15	1.10E-05
IGFBP3	0.48	5.68E-03
IGFBP4	0.45	6.23E-04
IGFBP5	0.48	3.74E-03
IGFBP7-AS1	0.65	9.94E-03
IGSF10	0.32	1.96E-05
IGSF9B	2.12	8.27E-04
IKZF1	0.64	3.00E-03
IKZF2	0.61	3.67E-03
IL15	0.57	9.02E-04
IL17RC	1.60	2.72E-02
IL18R1	0.58	2.43E-02
IL18RAP	0.57	1.09E-02
IL19	0.63	3.84E-02
IL1RL2	0.56	5.03E-03
IL21-AS1	0.60	6.23E-04
IL22RA2	0.59	4.02E-03
IL31RA	0.61	3.16E-03
IL33	0.42	3.01E-09
IL36G	0.59	1.71E-02
IL5RA	0.51	6.71E-03
IL7	0.59	2.54E-03
IL7R	0.62	1.31E-02
ILDR1	0.59	8.07E-04
ILF3	1.52	3.71E-03
IMPG1	0.63	4.55E-03
IMPG2	0.62	4.24E-04
ING5	1.78	1.84E-04
INHBA	0.53	1.96E-03
INHBA-AS1	0.62	7.34E-03
INMT-MINDY4	0.51	3.94E-04
INPP5A	1.70	9.42E-04
INSC	0.66	2.49E-02
INSR	2.04	1.74E-05
IP6K2	1.59	8.85E-03
IP6K3	2.96	4.86E-07
IQCE	1.56	8.62E-03

Gene ID	Fold Change: AF+HF RA versus NF RA	FDR
IQCM	0.63	2.47E-02
IRAK3	0.57	9.11E-03
ISCU	0.58	1.77E-03
ISM1	0.60	6.10E-03
ISOC1	0.53	3.15E-04
IST1	1.79	2.68E-05
ISX	0.58	1.20E-02
ITGA1	0.53	1.80E-04
ITGA4	0.58	1.03E-02
ITGA7	1.70	9.86E-03
ITGB8	0.55	1.79E-03
ITGBL1	0.49	1.13E-05
ITPK1	2.35	5.79E-05
ITPKC	1.69	5.55E-03
ITPRIP	3.01	6.69E-05
IVNS1ABP	1.83	4.33E-03
IYD	0.58	5.22E-04
JADE2	1.55	1.26E-02
JAKMIP2	0.59	6.53E-03
JAKMIP2-AS1	0.58	6.79E-03
JAML	0.59	6.38E-04
JARID2	1.58	4.05E-03
JAZF1-AS1	0.62	6.92E-04
JDP2	1.54	1.45E-02
JMJD6	1.88	1.61E-05
JPH2	2.24	8.30E-05
JPT2	1.53	1.42E-02
JRK	1.75	1.07E-02
JRKL	0.61	2.65E-03
KANK1	1.79	3.59E-04
KANK2	1.51	1.64E-02
KANK4	0.65	1.01E-02
KAT8	1.60	3.15E-03
KAZN	0.47	4.34E-05
KC6	0.59	3.76E-02
KCNA5	0.43	5.66E-03
KCNAB2	2.56	4.19E-05
KCNB2	0.60	9.09E-03
KCND2	0.59	3.76E-02

Gene ID	Fold Change: AF+HF RA versus NF RA	FDR
KCNE4	0.62	2.93E-03
KCNH2	1.58	7.99E-03
KCNH5	0.63	1.16E-02
KCNH7	0.43	3.70E-05
KCNH8	0.64	3.26E-02
KCNIP2	2.29	4.56E-03
KCNIP2-AS1	2.58	4.61E-04
KCNIP4	0.61	2.98E-03
KCNJ1	0.62	2.31E-03
KCNJ10	0.64	1.80E-03
KCNJ12	2.07	2.76E-03
KCNJ15	0.65	3.59E-03
KCNJ4	1.84	1.77E-02
KCNK1	1.72	4.30E-03
KCNK2	0.63	2.16E-02
KCNMB4	0.63	1.65E-02
KCNQ1	1.68	1.01E-02
KCNQ5	0.65	3.67E-02
KCNS3	0.64	3.10E-03
KCNT2	0.42	2.95E-03
KCNV1	0.59	2.27E-02
KCTD1	1.68	8.48E-03
KCTD12	0.34	1.28E-04
KCTD16	0.57	5.51E-04
KDM4B	1.80	9.17E-04
KDM4D	0.62	5.80E-03
KDM6B	1.76	3.85E-03
KDR	0.49	4.23E-05
KHDRBS2	0.64	3.92E-02
KIAA0141	1.52	1.04E-02
KIAA0232	1.60	1.27E-03
KIAA0895L	2.05	2.26E-03
KIAA1324L	0.54	6.13E-03
KIAA1524	0.61	1.17E-02
KIAA1549L	0.62	3.10E-02
KIAA1614	1.97	5.06E-04
KIAA1755	0.58	1.35E-02
KIF11	0.65	9.34E-03
KIF18A	0.61	1.66E-02

Gene ID	Fold Change: AF+HF RA versus NF RA	FDR
KIF20B	0.62	9.13E-04
KIF23	0.63	2.54E-02
KIF6	0.61	4.67E-04
KIF9-AS1	1.71	1.34E-03
KIR3DL2	0.49	3.21E-03
KIT	0.63	3.72E-02
KITLG	0.61	1.44E-02
KL	0.56	5.22E-04
KLC1	2.35	1.10E-06
KLF15	4.62	4.94E-09
KLF17	0.62	2.93E-02
KLF9	1.91	2.66E-04
KLHDC4	1.57	9.42E-03
KLHL1	0.64	4.17E-02
KLHL14	0.65	1.70E-02
KLHL25	1.82	4.71E-04
KLHL2P1	0.48	6.79E-03
KLHL3	1.82	1.55E-03
KLHL30	1.68	1.68E-02
KLHL36	2.01	1.59E-03
KLHL5	0.63	2.25E-03
KLKP1	0.49	5.81E-03
KLRF1	0.47	8.04E-03
KMT2B	1.56	1.04E-02
KMT2D	1.68	2.98E-03
KMT5A	1.67	2.49E-03
KPNA7	0.65	2.18E-02
KRBA1	3.00	9.39E-06
KRR1	0.59	5.88E-03
KRT20	0.53	3.10E-03
KRTAP5-AS1	1.77	2.27E-02
KYNU	0.57	2.37E-03
L3MBTL3	0.65	1.11E-02
LAMA2	1.56	4.59E-02
LAMTOR3	0.52	3.61E-04
LAPTM4B	1.84	1.32E-03
LARGE1	2.04	9.36E-05
LARP4	1.75	1.08E-04
LATS2	1.97	5.69E-05
LAYN	0.48	7.30E-06
LCA5	0.60	2.51E-03
LCAT	2.41	1.85E-03
LCMT1-AS1	1.66	5.36E-03
LCP1	0.55	3.40E-03
LCP2	0.62	7.25E-03
LDB2	0.41	2.17E-07
LDB3	2.82	2.23E-05
LDHA	0.58	2.26E-02
LDLRAD4	2.01	4.39E-04
LEF1-AS1	0.60	1.08E-02
LEKR1	0.56	1.29E-05
LEMD2	1.98	1.11E-04
LENG8	2.21	1.28E-03
LENG8-AS1	2.28	1.02E-03
LEUTX	0.65	4.67E-02
LGALS1	0.27	4.57E-07
LGALS3BP	0.50	4.56E-03
LGI1	0.55	2.07E-03
LGR5	0.60	1.10E-03
LGSN	0.64	1.89E-02
LHFPL3	0.62	1.36E-03
LHPP	1.57	8.33E-03
LHX9	0.56	3.44E-03
LILRB4	0.59	8.41E-03
LIN28B	0.63	2.18E-03
LINC00032	0.65	8.56E-03
LINC00113	0.51	5.21E-03
LINC00158	0.57	2.48E-03
LINC00210	0.57	4.14E-03
LINC00221	0.61	6.38E-03
LINC00237	0.57	8.01E-03
LINC00271	0.59	3.71E-04
LINC00276	0.64	3.67E-02
LINC00282	0.64	4.62E-02
LINC00290	0.60	7.35E-03
LINC00293	0.50	5.06E-04
LINC00297	0.62	3.55E-02
LINC00298	0.61	5.90E-03

Gene ID	Fold Change: AF+HF RA versus NF RA	FDR
LINC00299	0.53	6.67E-04
LINC00305	0.61	4.16E-02
LINC00320	0.61	2.62E-02
LINC00326	0.56	1.99E-02
LINC00351	0.60	3.63E-02
LINC00358	0.51	5.50E-03
LINC00374	0.45	8.17E-04
LINC00376	0.60	8.85E-03
LINC00383	0.54	1.17E-02
LINC00393	0.66	2.18E-02
LINC00402	0.61	9.38E-03
LINC00448	0.54	2.51E-03
LINC00458	0.48	3.84E-03
LINC00466	0.67	5.18E-03
LINC00467	0.65	5.33E-03
LINC00477	0.55	1.58E-03
LINC00484	1.65	2.40E-02
LINC00485	0.59	1.34E-02
LINC00487	0.65	6.40E-03
LINC00492	0.62	3.50E-02
LINC00499	0.55	1.74E-03
LINC00506	0.54	2.43E-04
LINC00513	3.66	1.09E-08
LINC00534	0.65	1.61E-02
LINC00535	0.66	3.78E-03
LINC00536	0.61	2.24E-03
LINC00547	0.52	4.81E-04
LINC00550	0.62	2.30E-02
LINC00551	0.58	2.81E-02
LINC00558	0.51	8.09E-03
LINC00559	0.60	3.37E-02
LINC00571	0.50	1.22E-02
LINC00575	0.53	1.01E-02
LINC00581	0.61	8.37E-03
LINC00601	0.57	1.63E-02
LINC00603	0.61	2.38E-02
LINC00604	0.50	5.96E-04
LINC00607	0.64	4.84E-03
LINC00613	0.65	4.56E-02
LINC00635	0.60	4.23E-03
LINC00636	0.51	1.60E-03
LINC00639	0.63	3.22E-02
LINC00640	0.61	2.05E-02
LINC00687	0.64	1.09E-02
LINC00692	0.61	4.49E-02
LINC00708	0.49	6.21E-04
LINC00824	0.60	2.13E-03
LINC00836	0.59	2.98E-03
LINC00838	0.65	4.02E-02
LINC00843	2.44	9.33E-05
LINC00862	0.61	2.32E-03
LINC00884	1.80	1.65E-02
LINC00885	0.66	4.49E-02
LINC00907	0.62	3.27E-03
LINC00922	0.60	1.48E-03
LINC00924	0.52	7.55E-03
LINC00939	0.60	1.00E-02
LINC00944	0.60	4.43E-03
LINC00958	0.66	1.19E-02
LINC00967	0.44	4.57E-03
LINC00969	1.72	4.64E-02
LINC00970	0.56	9.21E-04
LINC00972	0.61	2.58E-02
LINC00976	0.60	4.54E-03
LINC01012	0.60	2.19E-02
LINC01013	0.60	2.33E-02
LINC01019	0.65	1.71E-02
LINC01020	0.59	9.77E-03
LINC01021	0.47	1.45E-04
LINC01024	0.64	6.85E-03
LINC01029	0.51	7.39E-04
LINC01035	0.56	6.19E-03
LINC01036	0.59	6.65E-04
LINC01037	0.51	1.10E-04
LINC01040	0.57	1.46E-02
LINC01049	0.60	1.38E-02
LINC01055	0.67	4.15E-02
LINC01075	0.47	4.34E-05

Gene ID	Fold Change: AF+HF RA versus NF RA	FDR
LINC01088	0.62	1.53E-02
LINC01090	0.63	5.21E-03
LINC01095	0.61	1.06E-02
LINC01098	0.56	1.32E-02
LINC01102	0.60	4.41E-03
LINC01111	0.60	3.96E-03
LINC01122	0.64	2.58E-02
LINC01128	1.60	1.29E-02
LINC01142	0.54	3.57E-02
LINC01146	0.59	4.38E-02
LINC01151	0.63	3.83E-02
LINC01162	0.62	4.12E-03
LINC01173	0.65	3.97E-02
LINC01179	0.60	1.12E-02
LINC01181	0.67	3.97E-02
LINC01194	0.62	4.61E-02
LINC01197	0.60	1.05E-03
LINC01205	0.65	1.01E-02
LINC01206	0.62	1.02E-03
LINC01208	0.58	5.92E-03
LINC01214	0.62	1.31E-02
LINC01215	0.58	6.01E-03
LINC01228	0.64	3.43E-02
LINC01239	0.60	2.58E-02
LINC01241	0.64	2.65E-02
LINC01248	0.57	1.58E-03
LINC01250	0.63	2.70E-03
LINC01252	0.58	1.59E-02
LINC01255	0.48	1.43E-03
LINC01266	0.59	7.15E-03
LINC01287	0.58	3.10E-02
LINC01288	0.62	7.70E-03
LINC01299	0.61	1.10E-03
LINC01307	0.65	9.11E-03
LINC01317	0.65	1.08E-02
LINC01322	0.59	4.73E-03
LINC01324	0.51	1.57E-02
LINC01331	0.63	1.91E-03
LINC01339	0.64	2.15E-02

Gene ID	Fold Change: AF+HF RA versus NF RA	FDR
LINC01344	0.65	3.55E-03
LINC01349	0.63	4.90E-02
LINC01358	0.64	1.86E-03
LINC01359	1.86	3.93E-03
LINC01362	0.57	4.78E-03
LINC01375	0.62	5.95E-03
LINC01378	0.65	3.30E-02
LINC01387	0.66	1.08E-02
LINC01392	0.61	3.08E-03
LINC01425	0.61	1.69E-03
LINC01432	0.57	3.44E-03
LINC01435	0.60	2.02E-03
LINC01440	0.61	4.50E-03
LINC01442	0.56	2.18E-02
LINC01448	0.56	4.80E-04
LINC01449	0.64	1.31E-02
LINC01450	0.55	1.09E-02
LINC01455	0.61	2.91E-02
LINC01467	0.66	2.35E-02
LINC01468	0.64	2.94E-02
LINC01473	0.61	8.03E-04
LINC01481	0.64	3.97E-02
LINC01483	0.65	1.41E-02
LINC01485	0.58	2.41E-03
LINC01497	0.60	3.49E-02
LINC01498	0.62	6.34E-03
LINC01500	0.65	5.64E-03
LINC01501	0.57	3.35E-03
LINC01509	0.59	2.09E-02
LINC01515	0.48	1.45E-06
LINC01516	0.64	2.83E-03
LINC01524	0.60	7.36E-04
LINC01550	0.63	9.27E-03
LINC01551	0.54	1.35E-03
LINC01559	0.61	4.56E-02
LINC01563	0.60	2.23E-02
LINC01566	0.65	3.36E-02
LINC01571	0.57	1.89E-02
LINC01572	0.60	1.95E-03

Gene ID	Fold Change: AF+HF RA versus NF RA	FDR	Gene ID	Fold Change: AF+HF RA versus NF RA	FDR
LINC01579	0.59	6.53E-04	*LINC01780*	0.64	8.10E-03
LINC01581	0.64	3.59E-02	*LINC01781*	0.59	7.84E-04
LINC01594	0.62	4.30E-03	*LINC01787*	0.61	4.49E-03
LINC01598	0.66	2.03E-02	*LINC01788*	0.58	4.01E-04
LINC01606	0.61	3.73E-02	*LINC01789*	0.58	3.31E-03
LINC01609	0.64	2.90E-02	*LINC01791*	0.58	1.17E-03
LINC01611	0.62	1.21E-02	*LINC01794*	0.58	3.29E-02
LINC01629	4.09	2.46E-03	*LINC01795*	0.54	9.96E-04
LINC01630	0.65	4.15E-03	*LINC01799*	0.66	3.05E-02
LINC01641	0.66	1.56E-02	*LINC01807*	0.51	2.93E-03
LINC01643	0.62	3.19E-03	*LINC01811*	0.63	2.48E-03
LINC01644	0.58	1.55E-02	*LINC01812*	0.60	2.00E-02
LINC01645	0.62	1.53E-02	*LINC01837*	0.60	5.35E-03
LINC01655	0.62	8.00E-03	*LINC01846*	0.52	4.16E-04
LINC01676	0.56	1.24E-02	*LINC01847*	0.66	9.90E-03
LINC01677	0.64	1.06E-02	*LINC01850*	0.62	3.53E-02
LINC01681	0.56	5.90E-04	*LINC01853*	0.61	3.66E-02
LINC01682	0.48	6.46E-04	*LINC01876*	0.58	1.76E-02
LINC01684	0.61	2.40E-02	*LINC01877*	0.66	6.91E-03
LINC01685	0.49	3.33E-03	*LINC01892*	0.57	2.89E-02
LINC01687	0.66	4.42E-02	*LINC01899*	0.54	2.05E-03
LINC01692	0.64	1.99E-02	*LINC01901*	0.62	9.41E-03
LINC01697	0.58	4.64E-02	*LINC01916*	0.61	2.74E-02
LINC01701	0.56	3.64E-03	*LINC01919*	0.58	5.22E-03
LINC01704	0.64	1.80E-02	*LINC01920*	0.57	9.09E-03
LINC01706	0.60	1.11E-02	*LINC01924*	0.65	2.25E-02
LINC01707	0.66	1.13E-02	*LINC01929*	0.59	1.19E-02
LINC01709	0.64	1.87E-02	*LINC01934*	0.61	3.67E-02
LINC01716	0.60	1.72E-02	*LINC01938*	0.63	7.41E-03
LINC01722	0.64	9.18E-03	*LINC01947*	0.63	2.52E-02
LINC01725	0.60	1.18E-02	*LINC01949*	0.54	9.32E-05
LINC01726	0.59	5.12E-04	*LINC01951*	0.64	5.63E-03
LINC01727	0.60	8.09E-03	*LINC01954*	1.83	2.45E-02
LINC01739	0.66	2.35E-02	*LINC01965*	0.59	1.42E-02
LINC01741	0.60	1.22E-02	*LINC01968*	0.55	3.46E-03
LINC01744	0.53	1.39E-03	*LINC01982*	0.61	8.73E-03
LINC01748	0.54	7.29E-04	*LINC01990*	0.60	9.55E-03
LINC01760	0.53	9.93E-03	*LINC01991*	0.60	7.39E-03
LINC01776	0.59	4.98E-03	*LINC02008*	0.64	7.51E-03

Gene ID	Fold Change: AF+HF RA versus NF RA	FDR
LINC02027	0.58	5.44E-03
LINC02031	0.56	4.31E-03
LINC02042	0.62	2.57E-02
LINC02045	0.59	2.53E-03
LINC02052	0.64	1.28E-02
LINC02057	0.63	1.62E-03
LINC02058	0.57	4.64E-03
LINC02064	0.43	4.19E-04
LINC02066	0.60	3.34E-02
LINC02067	0.57	3.42E-03
LINC02070	0.46	7.98E-04
LINC02086	0.61	7.79E-03
LINC02089	0.57	4.30E-03
LINC02096	0.58	2.97E-04
LINC02105	0.62	9.72E-04
LINC02107	0.64	2.98E-03
LINC02108	0.57	1.18E-02
LINC02109	0.56	1.04E-03
LINC02112	0.63	4.88E-02
LINC02113	0.54	3.06E-03
LINC02126	0.62	1.22E-02
LINC02136	0.60	2.32E-02
LINC02137	3.17	2.55E-03
LINC02141	0.62	1.14E-02
LINC02144	0.60	4.30E-02
LINC02148	2.83	1.02E-02
LINC02161	0.64	2.36E-02
LINC02165	0.63	1.37E-02
LINC02172	0.59	1.62E-02
LINC02173	0.54	2.03E-03
LINC02174	0.58	3.78E-03
LINC02177	0.66	1.60E-02
LINC02180	0.54	2.95E-03
LINC02183	0.53	1.72E-03
LINC02196	0.62	2.53E-03
LINC02198	0.65	1.09E-02
LINC02202	0.60	5.25E-03
LINC02223	0.61	3.82E-03
LINC02226	0.66	3.53E-02

Gene ID	Fold Change: AF+HF RA versus NF RA	FDR
LINC02227	0.60	3.89E-04
LINC02233	0.64	9.21E-03
LINC02234	0.48	1.66E-02
LINC02236	0.60	7.29E-03
LINC02240	0.58	3.62E-02
LINC02241	0.64	1.26E-02
LINC02261	0.65	5.79E-03
LINC02267	0.64	2.83E-02
LINC02275	0.61	3.32E-03
LINC02276	0.53	1.15E-02
LINC02284	0.59	4.99E-02
LINC02290	0.53	2.94E-04
LINC02291	0.56	1.22E-03
LINC02293	0.59	4.58E-02
LINC02299	0.61	4.33E-02
LINC02302	0.52	1.89E-04
LINC02306	0.35	2.46E-02
LINC02309	0.62	2.80E-02
LINC02312	0.58	8.42E-03
LINC02319	0.57	1.06E-02
LINC02325	0.62	3.90E-02
LINC02328	0.61	3.32E-03
LINC02330	0.65	2.33E-02
LINC02331	0.63	9.67E-03
LINC02334	0.51	1.79E-02
LINC02342	0.47	3.62E-04
LINC02346	0.64	3.02E-03
LINC02355	0.64	4.04E-02
LINC02357	0.58	5.74E-03
LINC02358	0.62	4.23E-02
LINC02364	0.42	1.94E-04
LINC02366	0.54	1.28E-02
LINC02367	0.62	1.82E-02
LINC02369	0.59	1.71E-02
LINC02375	0.65	1.91E-02
LINC02380	0.57	4.97E-04
LINC02383	0.65	2.09E-02
LINC02386	0.67	1.32E-02
LINC02389	0.63	6.60E-03

Gene ID	Fold Change: AF+HF RA versus NF RA	FDR
LINC02391	0.65	2.40E-02
LINC02398	0.58	1.10E-03
LINC02401	0.61	4.00E-02
LINC02402	0.50	5.39E-04
LINC02405	0.59	4.62E-04
LINC02408	0.64	3.78E-03
LINC02416	0.51	6.58E-05
LINC02422	0.63	2.95E-02
LINC02428	0.59	1.71E-03
LINC02431	0.29	8.94E-06
LINC02436	0.57	1.41E-02
LINC02438	0.62	2.44E-02
LINC02442	0.64	1.63E-02
LINC02450	0.65	1.85E-02
LINC02451	0.62	8.82E-03
LINC02452	0.56	1.34E-02
LINC02462	0.55	1.22E-02
LINC02465	0.63	2.46E-02
LINC02466	0.54	3.68E-03
LINC02471	0.64	1.71E-02
LINC02477	0.63	1.94E-02
LINC02490	0.45	1.37E-03
LINC02493	0.57	3.25E-03
LINC02498	0.53	2.98E-03
LINC02501	0.63	2.19E-02
LINC02503	0.61	3.91E-03
LINC02505	0.59	6.40E-03
LINC02509	0.59	2.66E-02
LINC02512	0.60	3.32E-03
LINC02513	0.58	2.59E-03
LINC02516	0.48	2.12E-04
LINC02525	0.62	2.32E-02
LINC02528	0.58	1.17E-03
LINC02529	0.62	2.00E-02
LINC02532	0.62	4.09E-03
LINC02540	0.59	1.08E-02
LINC02543	0.49	5.84E-03
LINC02546	0.58	4.57E-03
LINC02549	0.63	5.09E-03

Gene ID	Fold Change: AF+HF RA versus NF RA	FDR
LINC02550	0.57	1.14E-02
LINC-PINT	4.67	5.67E-09
LINGO2	0.62	1.48E-02
LIPF	0.60	3.62E-02
LIPG	0.66	1.63E-02
LIPI	0.62	2.46E-02
LIPJ	0.58	1.47E-02
LIPK	0.64	2.53E-02
LIPM	0.56	4.24E-04
LIPN	0.49	1.15E-02
LMBR1L	1.50	7.16E-03
LMF1	1.57	1.41E-02
LMNA	1.80	1.01E-02
LONP1	2.02	4.50E-05
LPA	0.66	1.14E-02
LPAR1	0.51	2.16E-04
LPCAT1	1.52	2.45E-02
LPCAT2	0.38	2.87E-09
LPIN3	1.66	9.05E-03
LPL	3.18	9.41E-03
LPP	1.61	2.17E-03
LRAT	0.65	8.11E-03
LRFN5	0.59	5.20E-03
LRGUK	0.64	4.06E-03
LRIG1	1.94	8.23E-06
LRMP	0.62	1.25E-03
LRP1	0.58	2.71E-03
LRP2BP	1.92	5.12E-03
LRP4	1.67	3.62E-03
LRP5L	2.09	1.92E-03
LRRC2	1.85	2.75E-03
LRRC29	1.61	2.48E-02
LRRC37A16P	1.68	3.33E-03
LRRC3B	0.60	2.38E-03
LRRC4C	0.62	1.13E-02
LRRC69	0.63	2.69E-03
LRRC72	0.66	6.10E-03
LRRC8A	1.73	1.03E-03
LRRCC1	0.63	4.06E-03

Gene ID	Fold Change: AF+HF RA versus NF RA	FDR
LRRFIP1	1.54	6.10E-03
LRRFIP2	1.66	3.96E-04
LRRIQ1	0.60	7.09E-03
LRRIQ3	0.61	1.44E-02
LRRIQ4	0.50	3.97E-03
LRRK1	0.66	4.84E-03
LRRK2	0.53	3.77E-06
LRSAM1	1.79	1.32E-03
LSAMP	0.63	1.51E-02
LSM12	1.62	1.18E-02
LSM5	0.65	3.36E-02
LTF	0.66	3.30E-02
LUC7L	1.87	2.03E-04
LUM	0.34	6.09E-05
LURAP1L-AS1	0.61	1.35E-02
LUZP2	0.59	4.80E-03
LY9	0.64	9.53E-03
LYN	0.56	1.52E-04
LYNX1	0.57	8.04E-03
LYPD6	0.58	1.70E-03
LYPD6B	0.60	4.15E-03
LYPD8	0.59	3.91E-03
LYPLAL1-AS1	0.53	2.95E-04
LYRM2	0.64	4.34E-02
LYSMD4	1.54	1.87E-02
LYZL1	0.61	2.81E-03
LZTR1	1.82	1.61E-03
M1AP	0.64	2.11E-03
MAATS1	0.56	3.67E-04
MAB21L3	0.66	2.61E-02
MACC1	0.60	8.73E-03
MACROD1	1.99	1.74E-03
MAD2L1	0.50	2.57E-03
MAEA	1.92	1.23E-04
MAF	0.35	2.93E-08
MAFK	1.84	1.35E-02
MAL2	0.61	6.97E-03
MALAT1	2.54	1.04E-03
MALRD1	0.57	6.38E-04
MAML1	1.61	1.86E-03
MAN1A1	0.48	7.48E-04
MAN1B1	1.64	1.83E-02
MAN2A1	0.65	8.01E-03
MAN2B1	1.50	2.19E-02
MAN2C1	1.68	1.40E-02
MANEA	0.51	1.06E-04
MAP1LC3A	1.59	1.36E-02
MAP2	0.66	2.58E-02
MAP2K2	1.56	2.17E-02
MAP2K3	2.23	2.50E-03
MAP2K6	0.60	3.81E-03
MAP3K10	1.64	2.73E-02
MAP3K14	1.55	1.94E-02
MAP3K20-AS1	0.63	1.21E-02
MAP3K3	1.56	2.52E-03
MAP3K6	1.63	4.06E-02
MAP4	1.79	5.23E-03
MAP7D1	1.69	4.15E-03
MAPK1	1.63	1.28E-03
MAPK4	2.40	1.98E-02
MAPK8IP3	2.00	1.33E-04
MAPKAPK2	1.85	1.05E-04
MAPRE2	1.57	1.23E-02
MARCH1	0.62	7.79E-03
MARCH10	0.61	8.56E-03
MARCH4	0.64	9.87E-03
MARCKS	0.39	1.16E-04
MARCO	0.49	2.15E-02
MARK2	1.80	3.67E-04
MARK3	2.21	8.51E-08
MARK4	1.72	4.78E-03
MAST4	1.60	6.56E-03
MAU2	2.21	8.99E-06
MAVS	1.83	2.61E-04
MB	0.34	7.81E-09
MBD4	2.37	1.65E-03
MBTPS1	1.89	3.16E-04
MCM3AP	1.69	2.04E-03

Gene ID	Fold Change: AF+HF RA versus NF RA	FDR
MCRIP2	2.11	3.26E-03
MCTP2	0.64	1.08E-02
MDFIC	0.66	2.34E-02
MDH1B	0.54	1.39E-03
MDH2	0.61	1.65E-03
MDM4	1.57	5.01E-03
ME1	0.63	3.33E-03
MECOM	0.44	1.88E-08
MED20	1.60	1.13E-02
MED24	1.53	6.32E-03
MED28	1.54	4.44E-03
MED9	1.84	4.22E-04
MEF2C-AS1	0.53	4.26E-04
MEF2C-AS2	0.63	2.27E-02
MEF2D	1.90	2.95E-05
MEGF10	0.56	1.05E-03
MEI4	0.65	3.66E-02
MELK	0.67	9.23E-03
MEOX2	0.47	3.94E-04
MEPE	0.54	3.24E-04
METTL11B	0.64	1.15E-02
METTL13	0.63	3.92E-03
METTL21EP	0.60	1.17E-02
METTL23	1.60	6.64E-03
MFAP4	0.40	6.16E-03
MFGE8	1.83	3.08E-03
MFSD2B	1.74	1.46E-02
MFSD9	0.66	5.90E-03
MGAT4C	0.49	1.09E-03
MGAT4D	0.59	2.28E-02
MGEA5	1.81	1.58E-03
MGRN1	1.95	3.07E-04
MGST1	0.44	1.28E-03
MICAL2	1.71	1.21E-03
MICAL3	1.93	1.26E-03
MIDN	1.60	2.83E-02
MIER2	1.52	1.62E-02
MIGA2	1.86	8.14E-04
MIIP	1.67	2.56E-02

Gene ID	Fold Change: AF+HF RA versus NF RA	FDR
MINK1	1.67	2.29E-03
MIR100HG	0.51	3.40E-02
MIR1-1HG	2.15	4.06E-03
MIR155HG	0.67	1.85E-02
MIR181A1HG	0.50	4.01E-04
MIR193BHG	3.11	7.36E-12
MIR2052HG	0.63	2.72E-02
MIR22HG	1.77	9.36E-03
MIR3681HG	0.62	4.48E-03
MIR3936HG	1.70	6.03E-04
MIR3945HG	0.58	1.54E-02
MIR3976HG	0.60	4.72E-03
MIR4290HG	0.65	4.63E-02
MIR4300HG	0.65	2.45E-02
MIR4422HG	0.60	1.47E-02
MIR4527HG	0.66	1.15E-02
MIR4697HG	1.74	1.30E-02
MIR548A1HG	0.65	1.11E-02
MIR663AHG	0.64	3.82E-02
MIR99AHG	0.61	1.80E-04
MIRLET7BHG	1.77	1.80E-03
MKI67	0.53	6.26E-03
MKNK1	1.65	1.32E-03
MKNK2	4.13	5.92E-05
MKRN2	1.95	4.26E-05
MLF1	1.76	7.12E-03
MLIP	1.57	1.10E-02
MLPH	2.03	6.95E-05
MLXIP	1.71	8.53E-04
MLYCD	2.06	3.49E-06
MMP13	0.52	3.35E-03
MMP16	0.58	5.86E-03
MMP20	0.57	1.37E-03
MMP26	0.62	1.63E-02
MMP8	0.52	1.15E-02
MMRN1	0.56	1.78E-02
MMS22L	0.62	1.06E-03
MOB2	2.04	1.01E-04
MOBP	0.62	1.69E-03

Gene ID	Fold Change: AF+HF RA versus NF RA	FDR	Gene ID	Fold Change: AF+HF RA versus NF RA	FDR
MOCS1	1.52	6.72E-03	*MSR1*	0.56	2.79E-03
MORC1	0.62	5.77E-03	*MT-ATP6*	0.13	1.23E-17
MORN2	0.65	7.34E-03	*MT-ATP8*	0.13	1.07E-13
MOXD2P	0.63	3.17E-02	*MTBP*	0.59	3.40E-03
MPC1	1.54	7.27E-03	*MTCH1*	1.55	1.34E-02
MPP2	2.29	2.20E-02	*MT-CO1*	0.18	5.93E-13
MPP3	3.49	7.95E-07	*MT-CO2*	0.13	6.39E-15
MPPED2	0.57	1.49E-03	*MT-CO3*	0.19	2.16E-13
MPRIP	1.85	4.27E-05	*MT-CYB*	0.12	4.22E-21
MPST	1.74	4.52E-03	*MTERF4*	1.61	1.20E-02
MPV17	1.50	7.54E-03	*MTG2*	1.72	3.62E-03
MPZL3	0.59	2.82E-03	*MTHFD2P1*	0.55	3.22E-03
MRAS	1.66	1.93E-02	*MTHFR*	2.61	2.43E-06
MRC1	0.44	5.86E-05	*MTMR14*	2.20	5.05E-07
MRFAP1	0.47	5.80E-04	*MT-ND1*	0.15	2.60E-13
MRGPRX3	0.61	8.69E-03	*MT-ND2*	0.13	1.98E-15
MROH1	1.66	1.00E-02	*MT-ND3*	0.16	1.13E-11
MROH2B	0.54	5.02E-05	*MT-ND4*	0.17	4.01E-14
MROH3P	0.66	2.68E-02	*MT-ND4L*	0.18	1.01E-12
MROH7-TTC4	1.87	2.75E-02	*MT-ND5*	0.17	2.07E-20
MRPL13	0.66	1.28E-02	*MT-ND6*	0.18	1.38E-28
MRPL24	0.56	1.13E-03	*MTNR1B*	0.64	3.77E-02
MRPL43	0.55	6.34E-03	*MT-RNR1*	0.31	4.72E-04
MRPL51	0.30	4.93E-07	*MT-RNR2*	0.20	6.05E-08
MRPS25	2.95	1.77E-09	*MTSS1L*	2.01	1.11E-05
MRPS33	0.64	1.55E-02	*MT-TF*	0.19	5.44E-07
MRVI1-AS1	0.53	1.76E-02	*MT-TL1*	0.21	1.14E-05
MS4A1	0.56	5.90E-03	*MTTP*	0.49	2.63E-05
MS4A12	0.64	2.79E-02	*MT-TY*	0.17	3.78E-10
MS4A13	0.60	2.55E-02	*MTUS1*	1.67	3.47E-04
MS4A14	0.56	9.67E-03	*MUC19*	0.57	6.21E-04
MS4A3	0.65	2.09E-02	*MUSK*	0.51	2.63E-05
MS4A4A	0.55	1.15E-02	*MXD4*	2.29	3.86E-03
MS4A4E	0.53	3.73E-04	*MXRA7*	2.41	2.34E-04
MS4A6A	0.37	9.28E-09	*MYBPC1*	0.64	1.69E-02
MS4A6E	0.62	2.73E-03	*MYCT1*	0.42	3.05E-05
MSC-AS1	0.54	5.51E-05	*MYH13*	0.58	1.03E-03
MSI2	1.86	8.91E-05	*MYH9*	1.61	1.04E-02
MSMO1	0.61	2.00E-02	*MYHAS*	0.61	2.39E-03

Gene ID	Fold Change: AF+HF RA versus NF RA	FDR
MYL12B	0.50	1.25E-03
MYL7	0.34	9.91E-04
MYLK3	1.91	4.61E-04
MYLK-AS1	1.52	4.18E-02
MYO15B	1.89	7.48E-03
MYO18A	1.75	1.63E-03
MYO18B	3.57	6.17E-07
MYO3A	0.65	7.02E-03
MYO3B	0.65	4.63E-03
MYOM1	1.80	1.81E-02
MYOM2	1.70	7.40E-03
MYOM3	2.15	1.74E-04
MYOZ2	1.64	7.02E-03
MYPN	2.01	3.31E-03
MYRIP	0.64	1.01E-03
MZT2A	1.64	1.85E-02
MZT2B	2.07	4.43E-04
N4BP2L2	1.54	4.10E-03
N6AMT1	0.65	8.33E-03
NAA40	1.76	9.05E-03
NAALAD2	0.64	1.45E-02
NABP1	0.50	3.33E-03
NACA	1.52	1.42E-02
NACC2	1.73	8.61E-04
NADK	1.68	3.43E-03
NAPA	1.57	3.09E-02
NAT9	1.57	4.59E-02
NATD1	1.59	2.24E-02
NAV3	0.62	3.62E-02
NAXD	1.95	3.07E-05
NBEAL2	1.80	5.18E-03
NBN	0.65	5.19E-03
NBPF21P	0.62	1.00E-02
NBPF22P	0.54	4.02E-02
NBPF26	1.81	3.55E-02
NCAM2	0.47	2.60E-05
NCAPG	0.61	3.06E-02
NCKAP1L	0.59	3.08E-03
NCKIPSD	1.65	3.81E-02

Gene ID	Fold Change: AF+HF RA versus NF RA	FDR
NCLN	1.66	3.63E-02
NCOA7	0.59	3.32E-04
NCOR2	1.63	2.24E-03
NDC80	0.59	1.26E-02
NDN	0.24	5.72E-10
NDNF	0.62	2.36E-02
NDRG4	2.97	1.04E-04
NDST3	0.60	6.57E-03
NDUFA3	0.60	1.99E-03
NDUFA4	0.58	2.02E-03
NDUFA6	0.62	4.94E-03
NDUFAB1	1.53	1.62E-02
NDUFAF3	0.41	1.15E-03
NDUFAF8	0.53	8.24E-03
NDUFB10	0.61	1.33E-02
NDUFB4	0.62	5.69E-03
NDUFS7	1.75	1.22E-02
NDUFV3	1.59	1.84E-03
NEAT1	3.95	9.17E-11
NECAB3	1.72	1.02E-03
NECTIN2	1.85	1.81E-03
NECTIN3	0.62	3.38E-03
NECTIN3-AS1	0.54	6.99E-03
NEDD4L	1.65	1.28E-02
NEGR1	0.37	1.77E-09
NEK2	0.52	1.43E-03
NELFB	2.00	4.57E-03
NELFCD	1.62	1.13E-02
NELL1	0.65	2.48E-02
NELL2	0.61	1.00E-02
NEU3	1.78	2.19E-02
NEXN	1.80	7.79E-03
NFASC	2.42	7.29E-04
NFATC1	1.86	7.59E-04
NFATC2	1.69	6.56E-03
NFATC2IP	1.63	1.22E-02
NFIC	2.06	2.63E-04
NID1	2.19	5.94E-03
NIPSNAP2	1.51	2.68E-03

Gene ID	Fold Change: AF+HF RA versus NF RA	FDR
NISCH	1.69	4.72E-03
NKAIN3	0.59	2.88E-03
NLGN1	0.57	1.71E-03
NLRP13	0.62	8.42E-03
NLRP14	0.55	5.51E-04
NLRP4	0.52	4.08E-03
NMI	0.56	8.16E-04
NMNAT1	1.50	4.05E-02
NMRAL2P	0.61	1.21E-02
NMU	0.66	1.36E-02
NMUR2	0.64	1.82E-02
NNT-AS1	1.60	6.05E-04
NOCT	1.69	1.32E-02
NOL3	1.80	4.17E-02
NOL4	0.63	3.71E-02
NORAD	0.52	3.87E-03
NOSTRIN	0.53	2.70E-04
NOVA1	0.56	1.35E-02
NOVA1-AS1	0.58	5.20E-03
NOX3	0.60	6.08E-03
NOX4	0.55	1.05E-02
NOX5	0.64	1.99E-02
NPAS2	1.90	8.02E-04
NPAS3	0.60	2.44E-03
NPC2	0.63	8.23E-03
NPFFR2	0.62	4.74E-02
NPHP3-AS1	0.66	7.20E-03
NPHP4	1.75	3.97E-03
NPHS2	0.56	1.63E-03
NPIPB11	2.04	1.96E-02
NPIPB3	2.66	1.95E-04
NPIPB4	2.43	6.79E-05
NPLOC4	1.88	1.61E-05
NPM1	1.55	1.19E-02
NPNT	0.62	7.30E-03
NPPA-AS1	2.64	2.81E-02
NPR1	0.60	3.48E-02
NPRL3	1.79	1.36E-03
NPY1R	0.53	1.41E-02

Gene ID	Fold Change: AF+HF RA versus NF RA	FDR
NR1I2	0.58	1.05E-02
NR2F2-AS1	0.57	3.83E-04
NR5A2	0.63	1.17E-03
NRCAM	0.63	9.68E-03
NRG1	0.62	5.63E-03
NRIR	0.49	6.16E-04
NRP1	0.60	4.33E-03
NRP2	0.61	1.26E-02
NRSN1	0.64	2.33E-02
NRSN2-AS1	2.22	1.74E-06
NRXN1	0.42	7.30E-05
NSUN4	1.60	3.00E-03
NTN1	2.33	7.27E-04
NTNG1	0.62	2.43E-02
NTRK3	0.65	2.70E-03
NUDCD1	0.66	2.63E-02
NUDT4	1.74	8.18E-03
NUDT6	0.65	4.63E-03
NUP37	0.66	4.78E-03
NUP85	1.53	1.16E-02
NXF1	1.60	6.59E-03
NXPH1	0.63	1.83E-02
NXPH2	0.57	1.59E-03
NYAP2	0.61	3.32E-03
OAZ2	2.17	2.66E-06
OBSCN	2.37	1.54E-04
OFCC1	0.64	4.29E-03
OGDH	1.53	4.47E-03
OIP5	1.76	3.14E-03
OIP5-AS1	1.53	2.07E-03
OLAH	0.61	2.05E-03
OLFM3	0.64	4.26E-02
OLFM4	0.59	2.07E-02
OLR1	0.51	1.32E-02
OOSP1	0.61	2.11E-03
OPA3	1.53	9.67E-03
OPCML	0.62	5.23E-03
OPN5	0.58	6.23E-04
OPRM1	0.64	5.79E-03

Gene ID	Fold Change: AF+HF RA versus NF RA	FDR
OPRPN	0.50	3.55E-03
OR10D3	0.50	3.13E-03
OR11G2	0.61	4.09E-02
OR1L8	0.57	3.95E-03
OR2M3	0.61	1.69E-02
OR2V2	0.62	3.82E-02
OR2Z1	0.54	1.13E-02
OR51L1	0.52	1.99E-03
OR5A1	0.63	8.60E-03
OR5AN1	0.62	2.57E-02
OR5AS1	0.52	3.93E-03
OR5G5P	0.54	3.29E-03
OR6A2	0.63	2.07E-02
OR6N1	0.60	1.38E-02
OR7C1	0.64	1.46E-02
OR8A1	0.60	2.40E-02
OR8B8	0.54	9.42E-03
OR8D1	0.42	1.07E-04
OR9Q1	0.62	2.08E-03
OR9Q2	0.48	2.42E-03
OSBPL1A	1.73	2.04E-02
OSBPL2	1.59	4.56E-03
OSER1-AS1	1.55	1.70E-02
OSGIN2	0.65	4.54E-03
OST4	0.47	1.05E-04
OTOR	0.58	1.12E-03
OTUD6B-AS1	0.65	2.24E-02
OTX2-AS1	0.56	5.29E-05
OXR1	0.64	4.19E-03
P2RY2	2.30	1.32E-03
P3H3	1.63	3.21E-02
PABPC4	1.81	1.86E-03
PACS1	1.60	1.19E-02
PACS2	1.68	2.80E-03
PAFAH1B1	1.71	1.33E-04
PAH	0.64	6.25E-03
PAK1	0.61	8.76E-03
PAK4	1.99	9.58E-04
PALLD	1.97	2.34E-04

Gene ID	Fold Change: AF+HF RA versus NF RA	FDR
PALM2-AKAP2	1.88	3.64E-03
PALMD	1.59	2.20E-02
PAMR1	0.65	1.37E-02
PANX3	0.58	2.39E-02
PAPPA2	0.58	7.31E-04
PAPSS1	0.65	9.90E-03
PAPSS2	0.57	2.23E-02
PAQR9	0.64	2.42E-02
PARP14	0.62	4.46E-03
PARPBP	0.63	1.38E-02
PARVA	1.94	7.99E-04
PARVB	1.68	4.08E-03
PAX1	0.52	1.47E-02
PAX3	0.61	1.17E-03
PBK	0.58	1.59E-03
PCAT1	0.58	1.49E-03
PCAT4	0.53	2.79E-02
PCAT5	0.59	9.42E-03
PCBP2	1.88	4.14E-05
PCDH10	0.54	9.55E-03
PCDH15	0.54	1.42E-03
PCDH17	0.53	7.43E-03
PCDH18	0.38	6.60E-05
PCED1B	0.54	1.47E-04
PCGEM1	0.61	2.87E-02
PCGF3	1.98	8.81E-05
PCID2	1.59	5.52E-03
PCLO	0.52	4.35E-05
PCNT	2.45	3.12E-04
PCNX3	1.51	3.07E-02
PCOLCE2	0.53	4.16E-04
PCSK2	0.61	3.15E-03
PCSK5	0.62	2.00E-03
PCSK7	1.86	3.38E-05
PCYOX1	0.51	1.10E-06
PDE10A	0.60	2.14E-03
PDE11A	0.62	2.83E-03
PDE1A	0.44	3.82E-07
PDE3A	1.89	1.22E-03

Gene ID	Fold Change: AF+HF RA versus NF RA	FDR	Gene ID	Fold Change: AF+HF RA versus NF RA	FDR
PDE4B	0.64	3.11E-02	*PIAS4*	1.83	1.35E-03
PDE5A	0.66	6.65E-03	*PID1*	0.52	2.81E-03
PDE6H	0.45	3.39E-04	*PIEZO2*	0.64	3.38E-03
PDE7B	0.49	5.92E-03	*PIGK*	0.64	1.84E-03
PDE8A	1.51	3.88E-02	*PIK3C2G*	0.51	2.23E-06
PDE8B	1.97	1.44E-02	*PIK3CG*	0.57	6.76E-04
PDILT	0.55	2.01E-03	*PIK3R1*	1.77	7.25E-03
PDK4	5.07	4.30E-03	*PIK3R6*	0.64	1.60E-02
PDLIM1	1.61	5.20E-03	*PINK1*	1.65	1.73E-02
PDLIM5	1.53	2.03E-02	*PIP5K1A*	1.79	7.10E-04
PDP2	1.59	1.19E-02	*PIP5K1C*	1.53	1.03E-02
PDPK1	1.76	4.46E-04	*PITPNB*	1.64	4.19E-03
PDYN-AS1	0.61	2.67E-03	*PKHD1*	0.62	6.50E-03
PDZD2	2.31	3.91E-04	*PKHD1L1*	0.44	4.07E-02
PDZPH1P	0.62	3.28E-02	*PKIA*	0.51	1.62E-05
PEBP1	0.65	7.09E-03	*PKIA-AS1*	0.66	2.22E-02
PEBP4	1.72	2.54E-02	*PKIB*	0.65	1.35E-02
PECAM1	0.66	8.84E-03	*PKN1*	1.64	2.28E-03
PEF1	1.56	5.09E-03	*PKN2-AS1*	0.58	3.53E-04
PELI1	0.60	6.83E-03	*PLA2G2A*	0.10	6.66E-07
PER1	2.77	1.94E-07	*PLA2G4A*	0.54	6.46E-04
PER2	2.37	1.80E-03	*PLA2G4C*	1.86	8.30E-04
PEX14	1.56	4.69E-03	*PLA2G7*	0.64	3.61E-03
PEX5L	0.63	1.53E-03	*PLAC8*	0.62	2.78E-03
PEX6	1.83	2.21E-03	*PLAGL2*	1.71	2.45E-03
PFN1	0.44	6.46E-04	*PLCE1*	1.51	4.61E-02
PGAP2	1.64	2.54E-03	*PLCH1*	0.60	5.06E-04
PGAP3	1.58	3.24E-02	*PLCXD2*	0.66	1.60E-02
PGS1	1.61	3.06E-03	*PLCZ1*	0.50	3.00E-06
PHF14	0.64	3.98E-03	*PLD3*	2.04	7.99E-05
PHF2	1.83	3.16E-04	*PLD5*	0.65	3.40E-03
PHF21B	1.61	2.40E-02	*PLEC*	1.97	1.90E-04
PHF24	0.65	4.63E-02	*PLEK*	0.53	7.61E-04
PHF2P2	0.61	1.18E-02	*PLEKHA2*	2.25	4.38E-06
PHPT1	0.50	7.38E-03	*PLEKHF1*	3.54	5.60E-06
PHRF1	1.73	5.80E-04	*PLEKHG7*	0.66	8.80E-03
PHYHD1	2.21	7.52E-03	*PLEKHH2*	0.64	4.33E-02
PHYKPL	1.79	4.46E-04	*PLEKHM1P1*	1.58	6.06E-03
PI16	0.59	1.40E-02	*PLEKHM2*	1.82	2.64E-04

Gene ID	Fold Change: AF+HF RA versus NF RA	FDR	Gene ID	Fold Change: AF+HF RA versus NF RA	FDR
PLEKHO1	1.51	4.94E-02	*POR*	1.68	2.67E-03
PLIN2	0.59	3.68E-02	*POT1*	0.67	6.24E-03
PLK4	0.65	2.20E-02	*POT1-AS1*	0.62	1.09E-02
PLPP4	0.63	4.15E-03	*POTEJ*	0.23	2.70E-02
PLPP7	1.79	3.15E-03	*POTEKP*	0.64	2.58E-02
PLPPR1	0.62	3.92E-03	*POU2AF1*	0.57	3.59E-03
PLPPR4	0.56	1.09E-02	*PPARA*	1.96	8.58E-05
PLPPR5	0.62	6.84E-03	*PPARD*	1.51	8.73E-03
PLRG1	0.64	2.09E-02	*PPARG*	0.64	5.46E-03
PLSCR1	0.45	4.85E-05	*PPFIA2*	0.58	2.22E-03
PLSCR2	0.62	1.06E-02	*PPIEL*	1.90	2.56E-04
PLSCR4	0.60	2.87E-02	*PPIL2*	1.95	3.81E-04
PLSCR5	0.60	3.16E-02	*PPM1F*	1.76	2.65E-02
PLUT	0.66	1.08E-02	*PPM1K*	1.85	1.20E-02
PLXDC2	0.49	2.94E-06	*PPP1R12C*	2.07	1.01E-05
PLXNA1	1.60	4.26E-03	*PPP1R13B*	1.85	4.11E-04
PMFBP1	0.60	1.18E-03	*PPP1R1C*	0.42	5.05E-07
PMM1	2.15	5.74E-04	*PPP1R26*	1.73	8.83E-03
PMM2	1.77	9.06E-03	*PPP1R3A*	0.62	1.28E-02
PNISR	1.61	8.28E-03	*PPP2CB*	1.54	6.16E-03
PNLIPRP3	0.59	5.03E-04	*PPP2R1A*	1.63	1.98E-03
PNPLA7	1.88	1.12E-02	*PPP2R1B*	2.10	3.05E-03
POFUT2	1.75	2.10E-03	*PPP2R2D*	1.74	1.52E-04
POGLUT1	1.66	4.26E-02	*PPP3CA*	0.56	7.71E-04
POGZ	1.68	4.05E-04	*PPP4R4*	0.58	1.91E-03
POLG	1.70	1.99E-02	*PPP5C*	1.71	8.71E-04
POLM	1.73	7.99E-03	*PPP6R2*	1.73	4.56E-03
POLQ	0.64	1.97E-03	*PPRC1*	1.87	3.55E-02
POLR2K	0.49	6.56E-04	*PQLC1*	1.65	2.80E-03
POLR2L	0.40	6.69E-05	*PQLC2L*	0.66	3.52E-02
POLR3D	1.85	1.74E-03	*PRADC1*	1.55	4.00E-02
POLR3E	1.70	2.37E-04	*PRAMENP*	0.61	2.95E-03
POM121	1.68	3.47E-04	*PRDM9*	0.57	2.16E-03
POM121C	1.60	1.59E-03	*PRDX1*	0.44	7.73E-06
POM121L2	0.54	2.43E-04	*PRDX5*	0.40	5.14E-05
POMT1	1.82	3.68E-03	*PRELP*	0.36	8.07E-05
PON3	0.53	2.26E-04	*PREX2*	0.43	2.45E-09
POP4	1.53	4.43E-02	*PRG4*	0.21	3.12E-02
POPDC2	1.77	3.12E-03	*PRKAG2*	1.98	1.08E-02

Gene ID	Fold Change: AF+HF RA versus NF RA	FDR	Gene ID	Fold Change: AF+HF RA versus NF RA	FDR
PRKCB	0.66	6.05E-03	*PTPRE*	1.67	4.63E-03
PRKCQ	0.62	8.36E-04	*PTPRG-AS1*	0.65	8.86E-03
PRKCZ	1.59	3.52E-02	*PTPRO*	0.62	3.55E-02
PRKD1	0.44	8.66E-03	*PTPRQ*	0.43	5.75E-04
PRKD2	1.55	1.09E-02	*PTPRR*	0.57	8.67E-05
PRKRIP1	2.01	1.07E-05	*PTPRT*	0.65	1.51E-02
PRLR	0.58	3.73E-04	*PUDPP2*	0.56	9.23E-04
PRR16	0.63	3.26E-02	*PURG*	0.64	2.11E-02
PRR3	1.61	6.43E-03	*PUS1*	2.05	1.20E-04
PRRC2B	1.50	9.90E-03	*PXMP4*	1.56	1.83E-02
PRRG4	0.57	1.59E-03	*PYGM*	0.45	1.03E-03
PRSS12	0.58	3.89E-03	*QKI*	1.51	6.23E-03
PRSS23	0.51	1.14E-05	*QPCT*	0.58	9.42E-03
PRSS35	0.40	1.28E-03	*QRICH1*	1.51	4.17E-03
PSAP	0.51	3.81E-04	*QRICH2*	1.93	4.75E-03
PSG1	0.60	3.97E-02	*QSOX1*	1.86	3.05E-03
PSG8	0.56	7.27E-05	*QTRT1*	1.54	3.55E-02
PSG9	0.63	3.93E-02	*R3HDM2*	1.84	1.40E-04
PSKH1	1.62	6.87E-03	*RAB11FIP3*	2.13	1.31E-04
PSMC3	0.66	1.32E-02	*RAB19*	0.60	4.81E-02
PSMC5	0.66	2.94E-02	*RAB20*	1.72	8.77E-03
PSME4	1.78	3.03E-04	*RAB38*	0.58	8.98E-04
PTBP1	1.52	3.74E-02	*RAB3C*	0.63	1.19E-02
PTCHD4	0.53	1.53E-03	*RAB40B*	1.59	9.43E-03
PTDSS2	1.57	7.42E-03	*RAB40C*	1.98	1.03E-03
PTGES3	1.58	4.00E-03	*RABGAP1L*	1.53	1.09E-02
PTGIS	0.49	1.32E-03	*RACK1*	0.61	5.40E-03
PTGS1	0.59	9.98E-03	*RAD51-AS1*	1.91	1.19E-03
PTH1R	1.70	3.53E-02	*RAD9A*	1.52	2.82E-02
PTH2R	0.66	3.83E-03	*RAF1*	1.71	3.88E-04
PTN	0.62	2.02E-02	*RAI1*	1.96	6.75E-04
PTP4A1	0.62	4.27E-02	*RALGDS*	1.59	1.22E-02
PTP4A2	2.23	1.68E-03	*RALYL*	0.50	2.53E-03
PTPDC1	1.60	1.65E-02	*RAMP1*	2.90	1.05E-06
PTPN1	2.01	3.56E-06	*RANBP10*	1.78	3.12E-04
PTPN13	0.53	1.03E-03	*RANBP17*	0.61	1.27E-02
PTPN20	0.64	4.37E-02	*RANBP3*	1.83	1.06E-04
PTPRB	0.58	5.39E-03	*RANBP3L*	0.57	1.58E-03
PTPRC	0.46	1.95E-03	*RANGAP1*	1.67	2.06E-03

Gene ID	Fold Change: AF+HF RA versus NF RA	FDR	Gene ID	Fold Change: AF+HF RA versus NF RA	FDR
RAP1GAP2	1.82	2.25E-02	*RGS17*	0.63	2.71E-02
RAPGEF4-AS1	0.65	4.33E-02	*RGS18*	0.51	2.71E-03
RASD1	5.20	3.56E-05	*RGS5*	0.48	2.51E-04
RASGEF1B	0.66	1.04E-02	*RGS7*	0.64	4.21E-03
RASGRF2	0.61	6.92E-04	*RGS9*	0.66	2.44E-02
RASGRF2-AS1	0.65	3.50E-02	*RHBDD3*	1.86	1.55E-02
RASSF4	1.70	3.13E-03	*RHBDF1*	1.66	1.71E-02
RASSF9	0.47	1.26E-03	*RHBDF2*	1.69	3.62E-03
RAVER1	2.13	2.00E-04	*RHOH*	0.63	6.95E-04
RBAK-RBAKDN	2.12	1.19E-02	*RHOJ*	0.53	1.41E-04
RBM11	0.36	5.72E-05	*RHOU*	0.53	1.26E-03
RBM14-RBM4	2.18	1.58E-04	*RILPL1*	2.07	3.12E-05
RBM20	1.92	3.16E-03	*RIMS1*	0.64	2.64E-02
RBM26-AS1	0.54	1.60E-04	*RIMS2*	0.63	2.41E-02
RBM28	1.62	6.76E-04	*RING1*	1.85	7.20E-03
RBM39	1.55	2.36E-03	*RIPK1*	1.66	1.34E-03
RBM4	2.37	1.34E-05	*RIPK2*	0.63	9.67E-03
RBM43	0.54	4.29E-03	*RIPOR1*	1.86	3.12E-04
RBM47	0.65	3.59E-03	*RIT2*	0.62	3.93E-02
RBM4B	2.05	2.05E-03	*RMND5B*	1.71	8.94E-04
RBM5	1.88	3.38E-05	*RMST*	0.50	6.79E-05
RBPMS	1.92	5.47E-04	*RNASE1*	0.22	1.14E-06
RBPMS2	1.68	6.46E-04	*RNASEL*	0.60	5.51E-03
RBX1	1.60	6.87E-03	*RNF115*	1.52	3.35E-02
RCAN2	1.80	1.46E-02	*RNF122*	1.80	3.73E-02
RCC2	1.68	1.70E-03	*RNF123*	1.72	1.92E-03
RCN1	0.65	2.53E-03	*RNF144B*	0.45	4.47E-07
RDH10-AS1	0.66	1.89E-02	*RNF150*	1.91	5.43E-03
REG4	0.48	1.13E-05	*RNF152*	0.62	2.95E-04
RER1	1.63	5.75E-03	*RNF181*	0.51	2.45E-04
RERG	0.62	1.27E-02	*RNF207*	1.87	6.02E-03
RERGL	0.53	1.62E-05	*RNF216*	1.68	4.92E-02
RETNLB	0.62	3.24E-02	*RNF217-AS1*	0.65	4.67E-02
REXO1	1.79	1.21E-03	*RNF219-AS1*	0.66	1.73E-02
RFX1	1.64	4.41E-03	*RNF40*	1.76	1.10E-03
RFX3	0.58	4.87E-03	*RNF5*	0.62	4.06E-02
RFX6	0.60	3.30E-02	*RNH1*	1.51	2.24E-02
RGPD3	0.65	3.76E-02	*RNLS*	0.62	5.96E-04
RGS13	0.63	1.52E-02	*RNPS1*	1.73	2.24E-04

Gene ID	Fold Change: AF+HF RA versus NF RA	FDR
ROBO1	0.51	1.59E-03
ROCK1P1	0.66	2.21E-02
ROMO1	0.47	3.83E-04
ROR1	2.13	1.82E-02
RORB	0.51	2.85E-03
RORB-AS1	0.54	9.49E-04
ROS1	0.65	4.86E-02
RP1	0.65	1.41E-02
RPE65	0.55	9.68E-03
RPGRIP1L	0.60	1.49E-03
RPH3A	0.64	3.97E-03
RPL11	0.48	1.80E-03
RPL18	0.42	4.26E-04
RPL19	0.60	9.26E-03
RPL23	0.34	1.16E-05
RPL27	0.36	9.04E-06
RPL3	0.51	4.38E-03
RPL30	0.49	8.27E-06
RPL31	0.63	7.57E-03
RPL32P3	1.70	5.22E-03
RPL34	0.47	4.36E-04
RPL35	0.61	4.30E-02
RPL36	0.52	6.38E-03
RPL37A	0.63	3.31E-03
RPL38	0.47	5.31E-05
RPL39L	0.62	2.70E-03
RPL4	0.50	5.06E-04
RPL8	0.35	7.53E-05
RPLP0	0.53	4.57E-03
RPLP1	0.65	3.20E-02
RPLP2	0.57	9.80E-03
RPS11	0.33	6.02E-05
RPS13	0.52	3.18E-03
RPS14	0.61	3.05E-03
RPS15	0.43	9.56E-04
RPS16	0.45	8.84E-03
RPS18	0.54	3.29E-03
RPS20	0.56	7.69E-04
RPS21	0.52	7.15E-03

Gene ID	Fold Change: AF+HF RA versus NF RA	FDR
RPS24	0.55	2.48E-03
RPS25	0.55	9.44E-04
RPS27A	0.59	1.26E-02
RPS5	0.57	5.25E-03
RPS6	0.51	7.11E-04
RPS6KA2	1.72	7.95E-03
RPS6KA5	0.65	8.47E-03
RPS8	0.45	4.99E-04
RRAGA	0.43	2.11E-04
RRBP1	1.85	8.11E-03
RRH	0.61	4.62E-03
RRNAD1	1.54	1.43E-02
RRP12	1.84	6.10E-03
RSAD2	0.62	2.55E-02
RSL24D1	0.64	3.22E-02
RSPO3	0.57	1.50E-02
RTKN2	0.65	1.86E-02
RTN1	0.60	9.46E-04
RTN4RL1	1.75	2.17E-02
RUBCN	1.70	1.25E-03
RUBCNL	0.65	1.85E-02
RUNDC1	1.54	2.54E-02
RXFP1	0.66	1.69E-02
RXFP2	0.63	2.09E-02
RXRB	1.78	5.35E-03
RYR2	1.94	1.26E-02
S1PR1	0.61	4.38E-02
S1PR3	0.55	4.33E-03
SAFB	1.62	5.56E-04
SAFB2	1.73	5.06E-04
SAG	0.63	2.76E-02
SALL2	0.57	2.65E-03
SAMD12-AS1	0.62	1.56E-02
SAMD13	0.65	7.74E-03
SAMD15	0.61	1.09E-02
SAMD3	0.65	7.91E-03
SAMD4B	1.84	5.31E-05
SAMD7	0.60	2.35E-03
SAMD9	0.60	4.17E-02

Gene ID	Fold Change: AF+HF RA versus NF RA	FDR
SAMMSON	0.65	2.45E-02
SAMSN1	0.53	1.43E-03
SAP18	0.58	2.78E-04
SAP30BP	1.59	8.17E-04
SART1	1.57	1.15E-02
SATB1-AS1	0.61	2.34E-02
SATB2	0.56	3.81E-04
SAXO1	0.65	4.10E-03
SCAMP1-AS1	0.62	4.49E-03
SCARA3	0.67	1.91E-02
SCARA5	0.59	6.21E-03
SCD	0.18	1.29E-02
SCEL	0.61	8.32E-03
SCG5	0.61	4.68E-02
SCGB3A2	3.51	2.71E-02
SCGN	0.49	1.96E-05
SCHLAP1	0.65	4.42E-02
SCIN	0.63	1.60E-02
SCML4	0.64	2.78E-03
SCN11A	0.48	6.00E-07
SCN2A	0.58	6.40E-03
SCN5A	2.31	1.08E-05
SCN7A	0.44	7.99E-05
SCN8A	0.66	1.15E-02
SDF4	1.69	3.38E-03
SDHAP3	1.52	3.98E-03
SEC13	1.81	6.52E-05
SEC16A	1.74	6.52E-05
SEC24A	1.52	2.19E-02
SEC24B-AS1	0.65	4.51E-03
SEC31A	1.50	2.38E-02
SEC31B	1.55	3.05E-02
SECISBP2	1.53	2.10E-03
SEL1L2	0.62	1.61E-03
SELENOO	2.43	3.04E-07
SELP	0.58	3.29E-02
SEMA3C	0.60	3.50E-02
SEMA3D	0.59	2.10E-02
SEMA4B	2.02	5.53E-04

Gene ID	Fold Change: AF+HF RA versus NF RA	FDR
SEMA6D	0.61	2.49E-03
SEPT2	1.67	4.46E-03
SEPT7P9	0.53	7.78E-03
SERPINA5	0.55	1.01E-02
SERPINA9	0.60	1.02E-02
SERPINB11	0.61	1.62E-03
SERPINB12	0.43	3.33E-04
SERPINB13	0.61	3.77E-02
SERPINB2	0.55	4.08E-02
SERPINB6	2.12	8.50E-04
SERPINB7	0.52	2.91E-04
SERPINB8	0.57	1.34E-03
SERPINE1	3.58	4.69E-02
SERPINE3	0.55	7.06E-03
SERPING1	0.37	5.06E-04
SERPINI1	0.65	1.49E-02
SERPINI2	0.32	3.61E-04
SERTAD4	0.50	9.59E-04
SESTD1	0.64	1.75E-03
SETD6	1.65	1.32E-02
SEZ6L	0.66	5.23E-03
SF1	1.70	1.36E-03
SF3A2	1.56	4.90E-02
SFPQ	1.54	1.91E-03
SFRP4	0.59	2.88E-03
SFSWAP	1.71	9.81E-05
SFXN2	1.65	2.90E-02
SFXN4	1.84	4.72E-04
SFXN5	1.73	1.83E-03
SGCA	1.58	2.24E-02
SGCZ	0.62	1.16E-02
SGIP1	0.66	4.77E-02
SGMS1-AS1	0.65	1.24E-02
SGO2	0.63	6.86E-03
SGSM2	1.72	2.81E-03
SGTA	2.05	3.30E-03
SH2B1	1.82	2.41E-03
SH2B3	1.73	5.88E-04
SH3BP2	1.97	4.03E-02

Gene ID	Fold Change: AF+HF RA versus NF RA	FDR
SH3GL1	1.71	2.62E-03
SH3GL3	0.52	2.71E-03
SH3GLB2	1.68	2.09E-02
SH3PXD2A	1.52	9.56E-03
SH3RF3	1.54	5.90E-03
SHC1	1.67	1.19E-02
SHC2	1.79	1.44E-02
SHISA9	0.65	4.35E-03
SHOX2	0.46	3.07E-03
SIDT1	0.66	1.76E-02
SIK2	1.92	1.49E-02
SIM1	0.60	7.69E-03
SIN3B	1.67	2.26E-03
SIRPB2	0.50	1.22E-02
SIRT2	1.72	2.59E-03
SIRT3	1.52	1.23E-02
SKA1	0.61	9.22E-03
SKAP1	0.66	5.18E-03
SKAP2	0.61	2.00E-03
SKI	1.66	1.53E-02
SKOR2	0.64	3.18E-02
SLAMF1	0.59	7.67E-04
SLC12A1	0.61	2.24E-03
SLC12A4	1.67	1.02E-02
SLC13A4	1.51	3.90E-02
SLC14A2	0.67	3.56E-03
SLC15A1	0.59	2.44E-03
SLC15A2	0.56	1.93E-02
SLC15A5	0.61	1.71E-02
SLC16A14	0.56	4.69E-03
SLC16A1-AS1	1.91	3.73E-04
SLC17A1	0.53	7.29E-04
SLC17A2	0.47	4.14E-05
SLC17A3	0.50	4.46E-02
SLC17A5	1.74	1.35E-03
SLC17A8	0.62	2.66E-03
SLC19A2	2.85	8.36E-04
SLC1A1	0.67	9.60E-03
SLC1A2	0.61	2.09E-02

Gene ID	Fold Change: AF+HF RA versus NF RA	FDR
SLC1A3	2.32	9.65E-04
SLC20A2	1.55	1.65E-02
SLC22A16	0.55	9.27E-05
SLC22A2	0.60	1.90E-03
SLC22A4	1.73	7.76E-04
SLC22A5	1.87	2.37E-04
SLC24A2	0.63	1.03E-03
SLC25A11	0.55	2.18E-02
SLC25A18	2.50	9.95E-06
SLC25A20	1.72	2.13E-03
SLC25A21	0.67	8.36E-03
SLC25A25-AS1	2.89	3.82E-07
SLC25A29	2.40	2.21E-05
SLC25A31	0.60	7.57E-03
SLC25A42	1.78	1.19E-02
SLC26A5	0.58	4.62E-04
SLC26A7	0.63	1.67E-02
SLC26A8	0.65	7.98E-03
SLC27A1	2.71	1.87E-06
SLC27A2	0.60	4.04E-03
SLC27A4	1.56	2.12E-02
SLC27A5	1.80	2.15E-03
SLC28A3	0.66	2.31E-02
SLC2A1	1.69	3.37E-02
SLC2A2	0.60	4.71E-02
SLC30A5	1.50	1.19E-02
SLC30A8	0.65	1.29E-02
SLC31A1	1.66	2.61E-02
SLC35C2	1.82	1.69E-03
SLC35E2B	1.61	1.90E-02
SLC36A3	0.63	8.75E-03
SLC38A2	1.84	2.36E-02
SLC38A7	1.59	7.59E-03
SLC39A12	0.63	2.02E-03
SLC39A13	1.60	2.82E-02
SLC39A14	1.84	3.32E-04
SLC39A8	0.67	4.04E-02
SLC40A1	0.57	1.06E-03
SLC43A1	1.68	3.00E-02

Gene ID	Fold Change: AF+HF RA versus NF RA	FDR
SLC44A5	0.57	5.35E-03
SLC45A4	1.79	2.32E-03
SLC4A10	0.66	1.39E-02
SLC4A4	0.65	5.41E-03
SLC5A12	0.33	3.71E-02
SLC5A6	1.67	7.20E-03
SLC5A8	0.60	1.65E-02
SLC5A9	0.50	2.86E-03
SLC6A6	3.63	3.98E-04
SLC7A1	1.90	1.32E-02
SLC7A11	0.36	3.40E-03
SLC7A11-AS1	0.43	3.32E-04
SLC7A13	0.63	5.30E-03
SLC7A6	1.67	1.02E-03
SLC7A8	2.53	1.54E-06
SLC8A1	1.67	3.30E-02
SLC8A1-AS1	0.63	1.40E-02
SLC9A2	0.57	1.37E-04
SLC9A3R1	1.79	3.49E-02
SLC9A9	0.51	7.75E-06
SLC9C2	0.63	4.77E-03
SLCO1B1	0.65	3.50E-02
SLCO4C1	0.60	2.03E-02
SLCO6A1	0.64	2.27E-02
SLFN11	0.49	1.62E-04
SLIRP	0.48	1.28E-04
SLIT2	0.50	1.37E-04
SLN	0.35	6.94E-03
SLU7	0.63	5.09E-03
SMAD3	1.84	9.42E-03
SMAD9	0.55	4.63E-02
SMARCA4	1.50	9.08E-03
SMARCB1	1.88	8.81E-05
SMC2	0.60	3.69E-03
SMC2-AS1	0.35	1.04E-03
SMCO1	0.41	5.69E-06
SMG1	1.59	2.07E-03
SMG1P3	2.35	2.45E-07
SMG5	1.71	7.03E-04

Gene ID	Fold Change: AF+HF RA versus NF RA	FDR
SMG9	1.57	8.04E-03
SMILR	0.62	1.99E-02
SMIM15-AS1	0.65	1.18E-03
SMIM21	0.56	1.04E-02
SMIM4	1.81	1.48E-03
SMLR1	0.59	6.95E-03
SMPD4	1.64	6.53E-04
SNAI2	0.40	3.05E-04
SNAP23	1.67	3.82E-03
SNAP25-AS1	0.64	3.88E-02
SNAP47	2.31	2.46E-03
SNAP91	0.63	2.44E-02
SNAPC4	1.84	1.55E-02
SNCAIP	0.65	4.98E-02
SNHG14	1.57	1.02E-02
SNHG17	1.51	2.38E-02
SNORD3A	0.44	1.09E-02
SNRNP70	1.75	4.06E-03
SNRPB2	0.57	1.08E-03
SNRPD2	0.60	4.63E-03
SNTA1	1.84	4.50E-04
SNTB1	0.63	9.74E-04
SNTG1	0.63	1.61E-02
SNTN	0.54	1.89E-04
SOD1	0.41	8.74E-10
SOGA1	1.61	1.12E-03
SON	1.72	1.06E-03
SORBS1	2.44	2.75E-07
SORBS2	1.77	4.83E-02
SORCS1	0.55	6.59E-04
SORCS3	0.64	1.15E-02
SORT1	1.55	6.69E-03
SOS2	1.53	3.32E-03
SOSTDC1	0.56	1.61E-03
SOX5	0.43	5.45E-05
SP140	0.62	4.01E-03
SP140L	0.61	8.69E-04
SPAAR	0.57	6.71E-05
SPACA3	0.62	3.26E-02

Gene ID	Fold Change: AF+HF RA versus NF RA	FDR
SPACA6	1.73	7.82E-03
SPAG16	0.59	1.28E-03
SPAG17	0.63	7.93E-03
SPARC	0.43	3.81E-04
SPARCL1	0.50	8.83E-03
SPATA16	0.61	1.67E-02
SPATA17	0.55	2.55E-04
SPATA18	0.64	1.15E-02
SPATA6	0.59	6.70E-05
SPCS2	1.56	6.27E-03
SPDYE5	2.53	7.89E-04
SPEF2	0.58	7.20E-05
SPERT	0.46	2.92E-04
SPG7	1.82	3.67E-04
SPHKAP	0.59	4.31E-03
SPOCK1	1.72	1.73E-02
SPOCK3	0.63	1.46E-02
SPON1	0.66	1.50E-02
SPP1	0.26	1.72E-03
SPP2	0.56	6.73E-04
SPPL2A	1.69	1.55E-03
SPPL2B	2.38	5.33E-05
SPRR2E	0.53	7.39E-03
SPTB	1.73	1.33E-03
SPTLC3	0.62	8.07E-03
SPTSSB	0.50	1.10E-02
SQSTM1	2.21	9.44E-06
SRA1	1.51	3.70E-03
SRL	2.24	3.21E-07
SRP14	0.32	3.32E-06
SRRM2	1.91	1.01E-03
SRRT	1.54	1.82E-02
SRSF1	2.03	2.07E-04
SRSF3	1.51	3.05E-02
SS18L1	1.59	4.56E-03
SSBP3	1.51	1.13E-02
ST18	0.59	5.51E-04
ST3GAL1	2.54	3.21E-07
ST3GAL2	1.58	2.99E-02

Gene ID	Fold Change: AF+HF RA versus NF RA	FDR
ST3GAL4	2.01	2.95E-03
ST3GAL5	0.59	7.28E-03
ST3GAL6	2.03	2.83E-03
ST6GAL2	0.65	2.55E-02
ST6GALNAC3	0.50	5.97E-06
ST6GALNAC5	0.51	5.55E-06
ST8SIA1	0.54	2.95E-04
ST8SIA4	0.50	4.97E-04
ST8SIA6	0.50	6.98E-05
STAB2	0.64	3.61E-03
STAC	0.60	3.70E-03
STAG3L2	2.62	1.12E-03
STAG3L4	1.65	2.87E-03
STAP1	0.66	1.65E-02
STARD3	1.81	4.21E-03
STARD4	0.50	3.78E-02
STARD4-AS1	0.59	7.71E-04
STARD7	1.54	4.04E-02
STARD7-AS1	1.83	2.23E-05
STAT3	1.70	6.12E-04
STEAP2	0.44	4.07E-06
STEAP2-AS1	0.61	1.14E-03
STIM2	0.49	1.05E-03
STK11	1.57	2.17E-02
STK31	0.66	5.77E-03
STK32A	0.65	7.86E-03
STK35	1.61	5.06E-03
STMN2	0.61	3.90E-02
STMND1	0.63	1.21E-02
STOML3	0.60	1.08E-02
STON2	0.59	5.22E-04
STOX2	1.50	2.66E-02
STPG2	0.58	2.87E-04
STRN4	1.63	1.01E-02
STX16-NPEPL1	1.58	3.03E-03
STX2	2.58	5.33E-06
STX5	1.66	2.02E-03
STXBP4	0.65	2.88E-03
STXBP5-AS1	0.57	3.00E-04

Gene ID	Fold Change: AF+HF RA versus NF RA	FDR
STYK1	0.60	4.77E-03
SUGCT	0.61	2.93E-04
SUGP2	1.63	1.99E-03
SULF1	0.66	3.28E-02
SULT1C4	0.50	2.84E-03
SULT6B1	0.65	2.89E-03
SUN1	1.73	1.68E-04
SUN2	1.56	4.71E-02
SUN3	0.62	3.52E-02
SURF4	1.65	4.54E-03
SUZ12P1	1.77	1.96E-04
SV2C	0.66	3.29E-02
SVEP1	0.55	2.04E-02
SVIL	2.90	4.60E-05
SYBU	0.59	4.71E-04
SYCP1	0.58	2.23E-03
SYCP2	0.42	1.58E-06
SYF2	0.65	2.56E-02
SYF2P2	1.80	3.76E-02
SYMPK	2.13	1.08E-05
SYNDIG1	2.75	1.57E-05
SYNE3	1.75	2.68E-03
SYNGR1	1.60	1.49E-02
SYNJ2	2.01	4.21E-04
SYNPO	1.53	6.49E-03
SYT1	0.60	2.09E-02
SYT10	0.51	2.17E-02
SYT2	1.63	3.67E-02
SYT6	0.58	9.53E-03
SZT2	1.58	2.55E-03
TACC2	2.19	1.82E-04
TACC3	1.79	1.22E-02
TACR2	1.91	5.90E-03
TACR3	0.66	4.69E-02
TADA2B	1.57	8.51E-03
TANC1	2.24	2.81E-04
TANC2	0.64	2.28E-03
TANGO2	3.19	8.04E-07
TAOK2	1.76	2.10E-03
TAPT1-AS1	0.63	4.18E-03
TBC1D1	1.84	2.77E-04
TBC1D10B	1.68	5.21E-03
TBC1D32	0.64	3.93E-03
TBC1D8	1.60	4.64E-02
TBCD	1.55	7.48E-03
TBX15	0.58	1.08E-02
TBX18	0.44	1.11E-05
TBX19	0.66	1.77E-02
TBX5	2.26	8.35E-04
TBX5-AS1	2.57	1.07E-04
TC2N	0.39	4.06E-08
TCAP	0.39	5.51E-04
TCF24	1.96	4.79E-03
TCF25	1.73	1.50E-04
TCF4	0.48	2.29E-05
TCF7	1.82	6.26E-04
TCL6	0.55	1.28E-02
TCP11	0.57	6.27E-03
TDRD1	0.66	4.48E-03
TDRD15	0.57	1.64E-02
TEAD3	2.10	1.01E-05
TEK	0.57	2.22E-04
TEKT3	0.63	4.96E-03
TEKT5	0.64	2.73E-02
TERB1	0.64	1.12E-02
TERB2	0.44	1.60E-03
TET1	0.63	2.71E-03
TEX26	0.58	5.24E-04
TEX26-AS1	0.64	1.45E-02
TEX41	0.60	1.16E-02
TEX48	0.59	2.54E-02
TFAP2B	0.59	2.18E-02
TFAP2D	0.59	2.74E-03
TFEB	2.37	5.58E-05
TFEC	0.58	2.95E-03
TGM3	0.56	4.06E-03
TGM6	0.59	5.40E-03
TGM7	0.67	3.73E-02

Gene ID	Fold Change: AF+HF RA versus NF RA	FDR
THAP4	1.83	2.81E-04
THEM7P	0.65	3.44E-03
THEMIS	0.61	1.51E-02
THEMIS3P	0.60	1.65E-02
THOP1	2.40	2.34E-04
THRAP3	1.56	2.97E-03
THRB-AS1	0.62	4.02E-03
THSD4	1.67	3.74E-03
THSD7A	0.60	7.55E-03
THSD7B	0.65	1.83E-02
THYN1	0.66	3.82E-02
TIAM1	0.58	3.19E-04
TIFA	0.62	4.43E-02
TIGIT	0.60	1.72E-03
TIMD4	0.61	1.25E-03
TIMM44	1.79	1.52E-03
TIMM8B	0.64	4.89E-02
TIMP2	0.58	4.83E-03
TINAG	0.61	1.34E-02
TJAP1	1.82	1.63E-04
TLE3	1.72	1.01E-02
TLL1	0.60	9.49E-03
TLR1	0.56	2.10E-04
TLR4	0.42	2.34E-04
TM4SF1	0.28	6.35E-04
TM4SF20	0.54	7.09E-04
TM9SF4	1.61	7.71E-04
TMBIM7P	0.60	7.97E-04
TMC1	0.65	4.69E-03
TMC3-AS1	0.60	3.30E-03
TMC5	0.67	7.89E-03
TMCO3	1.61	4.48E-03
TMCO5B	0.62	4.96E-02
TMEFF2	0.56	9.45E-04
TMEM100	2.21	1.04E-03
TMEM104	1.76	3.83E-04
TMEM108	0.65	3.93E-02
TMEM120B	1.58	3.07E-02
TMEM127	1.68	2.25E-03

Gene ID	Fold Change: AF+HF RA versus NF RA	FDR
TMEM132B	0.64	1.62E-02
TMEM132D	0.64	8.79E-03
TMEM144	0.60	3.66E-03
TMEM154	0.64	6.18E-03
TMEM156	0.64	1.99E-03
TMEM161A	1.72	3.34E-03
TMEM165	1.80	1.56E-04
TMEM169	0.63	1.28E-02
TMEM175	1.65	1.61E-02
TMEM178B	1.86	2.46E-02
TMEM196	0.54	4.63E-03
TMEM2	0.65	1.02E-02
TMEM200A	0.62	3.35E-03
TMEM201	1.72	2.03E-03
TMEM212	0.60	2.77E-03
TMEM214	1.61	3.30E-02
TMEM222	1.56	1.49E-02
TMEM232	0.63	2.31E-02
TMEM244	0.55	2.91E-04
TMEM246	0.59	1.77E-03
TMEM26-AS1	0.57	1.69E-03
TMEM43	2.69	5.06E-05
TMEM45A	0.61	8.75E-03
TMEM5-AS1	0.57	7.68E-03
TMEM63A	1.71	3.95E-03
TMEM63B	1.53	1.59E-02
TMEM64	0.66	6.16E-03
TMEM71	2.38	7.74E-03
TMEM74	0.60	1.15E-02
TMEM99	0.66	1.57E-02
TMOD1	1.65	2.05E-03
TMPRSS15	0.58	1.61E-02
TMPRSS9	1.61	1.15E-02
TMTC2	0.58	1.92E-03
TMTC3	0.66	1.32E-02
TNFAIP1	1.61	2.71E-02
TNFSF10	0.43	1.68E-06
TNFSF11	0.61	1.84E-03
TNFSF15	0.59	3.46E-03

Gene ID	Fold Change: AF+HF RA versus NF RA	FDR
TNFSF8	0.59	2.92E-03
TNIP2	1.58	3.76E-02
TNIP3	0.63	2.07E-02
TNK2	2.15	5.35E-03
TNN	0.61	3.02E-02
TNNC1	0.47	3.62E-06
TNNI3	0.58	2.70E-03
TNNT1	3.65	3.17E-07
TNR	0.63	2.15E-02
TNRC18	1.74	1.95E-03
TNS1	1.80	8.89E-04
TOB2	2.54	1.01E-05
TOM1	1.73	4.41E-04
TOM1L2	1.84	9.08E-05
TOMM22	0.66	4.47E-02
TOMM7	0.56	1.91E-04
TOP2A	0.61	1.78E-02
TOP3B	2.23	5.76E-05
TOX3	0.63	9.05E-03
TP53I11	1.78	3.39E-02
TP53TG1	0.53	3.08E-04
TP63	0.62	5.25E-03
TPD52L2	1.78	5.95E-04
TPGS1	2.59	1.69E-04
TPH2	0.58	3.82E-03
TPI1	0.49	5.51E-04
TPK1	0.63	4.61E-04
TPPP	2.17	1.45E-03
TPRA1	1.54	6.86E-03
TPRG1	0.66	6.84E-03
TPT1	0.50	2.36E-03
TPT1-AS1	2.09	2.29E-05
TPTE2	0.65	1.08E-02
TPTEP1	0.51	4.25E-04
TRABD2A	0.65	2.91E-02
TRAF2	1.87	1.59E-03
TRAK1	1.54	7.69E-03
TRAP1	1.62	7.80E-03
TRAPPC1	0.27	5.67E-04

Gene ID	Fold Change: AF+HF RA versus NF RA	FDR
TRAPPC12	1.72	2.66E-03
TRAT1	0.63	2.76E-02
TRDMT1	0.55	9.47E-05
TREM1	0.53	1.40E-02
TRHDE	0.55	3.32E-03
TRIB1	2.15	9.16E-03
TRIM14	0.66	4.15E-03
TRIM5	0.61	7.12E-03
TRIM54	1.98	7.82E-04
TRIM63	2.71	1.82E-11
TRIM68	0.66	2.60E-02
TRIM9	0.63	1.73E-03
TRIML2	0.64	2.31E-02
TRMT44	1.56	1.82E-03
TRMU	1.80	1.10E-03
TRNP1	1.64	5.27E-03
TRPC3	0.58	2.19E-02
TRPC4	0.38	1.96E-05
TRPC6	0.60	2.72E-03
TRPC7	0.63	1.21E-02
TRPM4	1.81	1.06E-03
TRPM6	0.65	4.56E-03
TRPM8	0.65	2.77E-02
TRPS1	0.66	1.68E-02
TRPV1	1.91	6.23E-04
TRPV5	0.65	3.65E-02
TSC2	1.55	1.44E-02
TSC22D1	2.16	3.51E-02
TSC22D4	2.27	4.00E-03
TSGA13	0.63	8.68E-03
TSHR	0.52	1.04E-03
TSHZ2	0.60	1.92E-03
TSNARE1	1.58	1.09E-02
TSPAN18	1.75	2.08E-03
TSPAN32	2.25	2.58E-02
TSPAN8	0.66	1.79E-02
TSPAN9	1.62	7.12E-03
TSSK3	1.61	2.68E-02
TTC29	0.64	8.11E-03

Gene ID	Fold Change: AF+HF RA versus NF RA	FDR
TTC31	1.56	1.90E-02
TTC38	1.86	9.13E-04
TTC39A	1.62	7.40E-03
TTC6	0.62	1.93E-03
TTK	0.50	5.40E-04
TTN-AS1	1.69	4.64E-02
TTYH3	2.02	9.62E-04
TUBAL3	0.49	3.38E-03
TUBB	0.49	7.90E-04
TUBB4B	0.57	1.74E-02
TUBD1	1.54	6.24E-03
TUBGCP2	1.63	3.93E-03
TUBGCP6	1.70	2.98E-03
TUFM	0.54	3.78E-03
TUG1	1.61	5.38E-03
TULP3P1	0.58	2.88E-03
TUSC3	0.65	4.36E-03
TWIST1	0.62	1.10E-03
TXLNA	1.54	4.39E-03
TXNDC8	0.63	2.11E-02
TXNL4B	1.67	6.78E-03
TYR	0.52	1.59E-03
U2AF2	1.54	4.64E-02
U91319.1	0.65	5.81E-03
U95743.1	0.61	1.25E-03
UBAC1	1.67	8.06E-04
UBALD2	2.47	1.36E-04
UBB	0.24	2.16E-08
UBE2B	1.77	1.08E-04
UBE2G2	1.76	1.02E-03
UBE2H	1.60	1.04E-03
UBE2J2	2.29	4.16E-05
UBE2K	1.57	1.36E-03
UBE2QL1	1.53	2.03E-02
UBE2U	0.63	1.79E-02
UBE2Z	1.53	3.00E-03
UBFD1	1.72	8.67E-04
UBL5	0.20	1.10E-07
UBN1	1.50	1.31E-02

Gene ID	Fold Change: AF+HF RA versus NF RA	FDR
UBR4	1.52	1.47E-02
UBTD1	1.65	4.56E-03
UCKL1	2.05	2.46E-02
UFL1-AS1	0.55	3.63E-04
UGT1A8	0.55	2.61E-03
UGT2A3	0.60	2.94E-02
UGT2B7	0.66	3.75E-02
ULK1	2.05	5.33E-04
UNC13C	0.61	2.51E-03
UNC45A	1.60	1.56E-02
UNC5C	0.43	2.83E-09
UNC5D	0.63	9.15E-03
UNC79	0.57	2.95E-05
UNK	1.76	6.15E-04
UNKL	1.81	2.58E-03
UPF3A	1.77	3.59E-04
UQCR10	0.47	2.24E-04
UQCRB	0.47	2.94E-06
UQCRQ	0.31	8.99E-07
URGCP	1.66	8.51E-03
USF2	1.60	1.07E-02
USH2A	0.52	1.79E-04
USP2	1.68	1.31E-02
USP22	1.51	2.83E-03
USP31	1.61	6.65E-03
USP36	1.61	6.16E-04
USP53	1.79	1.32E-03
UTRN	0.66	2.94E-02
UTS2B	0.64	7.23E-03
VAC14	1.56	2.41E-03
VAMP5	0.54	4.06E-03
VASH1	4.57	4.48E-04
VASP	1.66	1.09E-02
VAT1	0.66	2.93E-02
VAV3	0.61	7.90E-03
VCAM1	0.49	1.08E-02
VCAN	0.38	5.90E-03
VCL	2.54	1.03E-08
VCPKMT	1.67	8.35E-04

Gene ID	Fold Change: AF+HF RA versus NF RA	FDR
VDAC3	0.65	1.09E-02
VEGFB	0.53	1.91E-02
VEGFC	0.46	1.39E-03
VEPH1	0.61	2.17E-03
VEZF1	1.95	2.17E-04
VIM	0.47	2.83E-02
VIM-AS1	0.66	1.45E-02
VIT	0.50	4.61E-03
VN2R1P	0.65	3.89E-02
VNN2	0.60	2.05E-02
VNN3	0.60	1.47E-02
VPS51	1.76	6.21E-04
VRK2	0.66	7.91E-03
VSTM2A	0.65	4.14E-02
VSTM2L	2.07	1.74E-02
VTA1	0.63	3.00E-03
VWA5A	0.62	8.56E-03
VWDE	0.57	1.50E-02
VWF	0.58	1.25E-04
WASL	1.53	1.39E-02
WDR17	0.63	1.18E-02
WDR20	1.70	4.61E-04
WDR37	1.51	1.11E-02
WDR49	0.43	1.18E-03
WDR5	1.52	1.70E-02
WDR63	0.62	1.95E-02
WDR64	0.61	1.23E-03
WDR72	0.62	1.39E-02
WDR73	1.89	2.68E-04
WDR78	0.59	6.35E-04
WDR87	0.52	1.49E-03
WDR95P	0.57	3.07E-03
WDSUB1	1.68	6.37E-03
WEE2-AS1	0.63	2.17E-02
WFDC2	0.62	2.22E-02
WFDC8	0.59	1.65E-03
WIF1	0.58	1.88E-03
WIPI2	1.60	6.12E-03
WNT16	0.47	4.20E-05

Gene ID	Fold Change: AF+HF RA versus NF RA	FDR
WNT2	0.62	1.40E-02
WNT4	1.68	4.47E-02
WNT8B	0.64	2.03E-02
WRAP73	1.77	2.28E-02
WSB1	1.70	1.83E-02
WT1	0.57	1.70E-02
WT1-AS	0.62	9.05E-03
WTAPP1	0.56	7.53E-05
WTIP	1.65	3.17E-02
XDH	0.65	6.10E-03
XIRP2	0.39	9.32E-05
XKR3	0.61	2.35E-02
XKR4	0.59	9.87E-04
XPO1	1.95	3.31E-05
XPR1	1.88	3.21E-03
XRCC4	0.59	6.52E-04
XYLT2	2.00	4.08E-05
YAF2	1.51	4.64E-03
YBEY	1.95	2.80E-03
YBX2	3.12	1.51E-04
YBX3	1.73	2.76E-02
YPEL1	2.04	1.81E-04
YPEL2	1.51	1.39E-02
YTHDF1	1.50	2.21E-03
YWHAB	0.64	4.53E-03
YWHAEP7	0.63	9.04E-03
YY1AP1	1.90	1.40E-04
Z94057.1	0.49	6.73E-03
Z97206.2	0.61	3.28E-02
Z98749.3	1.74	2.08E-02
ZBTB16	4.26	1.77E-11
ZBTB17	1.61	8.25E-03
ZBTB38	1.58	1.34E-02
ZBTB41	0.63	6.60E-03
ZBTB43	1.62	1.21E-03
ZBTB46	1.72	6.58E-03
ZBTB48	1.73	9.91E-03
ZBTB49	1.70	6.59E-04
ZBTB7A	1.70	1.12E-02

Gene ID	Fold Change: AF+HF RA versus NF RA	FDR
ZBTB7B	1.76	4.04E-02
ZC3H7B	1.52	1.36E-02
ZC3HC1	1.59	4.14E-03
ZCCHC17	1.58	9.65E-03
ZDHHC11B	1.82	2.28E-02
ZDHHC14	1.76	1.74E-02
ZDHHC16	1.54	3.71E-03
ZDHHC18	1.91	2.89E-02
ZDHHC23	0.63	1.65E-02
ZDHHC24	1.57	4.34E-02
ZDHHC3	1.56	1.05E-03
ZEB1-AS1	0.61	1.04E-03
ZEB2P1	0.59	4.93E-03
ZER1	2.44	2.58E-07
ZFHX4-AS1	0.62	2.90E-02
ZFP36	2.80	1.72E-02
ZFP36L2	0.46	9.74E-03
ZFP69B	0.64	3.72E-02
ZFPM1	2.05	2.48E-03
ZFPM2-AS1	0.60	1.19E-03
ZFYVE21	1.69	2.04E-03
ZFYVE27	1.56	9.06E-03
ZHX1-C8orf76	1.54	1.08E-02
ZHX2	1.82	1.47E-04
ZMIZ1	2.25	2.13E-06
ZMYM5	1.65	1.39E-03
ZMYND12	0.63	4.27E-03
ZMYND8	1.50	3.42E-02
ZNF142	1.52	1.33E-02
ZNF189	2.76	1.10E-06
ZNF207	1.68	2.24E-04
ZNF214	0.61	5.46E-03
ZNF215	0.64	1.50E-02
ZNF251	1.52	1.40E-02
ZNF274	1.53	3.11E-03
ZNF276	1.97	7.95E-05
ZNF282	1.75	2.47E-04
ZNF362	1.72	6.81E-03
ZNF366	0.56	5.39E-05

Gene ID	Fold Change: AF+HF RA versus NF RA	FDR
ZNF385B	0.61	3.60E-02
ZNF44	1.62	2.36E-03
ZNF479	0.57	8.32E-03
ZNF492	0.63	1.22E-02
ZNF500	1.72	8.59E-03
ZNF516	1.51	1.52E-02
ZNF559-ZNF177	1.55	2.58E-02
ZNF562	1.55	3.14E-03
ZNF579	1.87	2.55E-02
ZNF586	1.95	2.24E-04
ZNF623	1.83	8.85E-03
ZNF663P	0.51	9.16E-04
ZNF672	1.61	1.08E-02
ZNF679	0.63	1.15E-02
ZNF680	1.54	2.68E-03
ZNF692	1.74	1.26E-03
ZNF7	1.68	4.95E-03
ZNF70	1.85	4.97E-04
ZNF727	0.66	4.73E-02
ZNF729	0.61	3.06E-03
ZNF74	1.57	4.36E-02
ZNF740	1.64	8.37E-03
ZNF75A	1.51	1.01E-02
ZNF767P	1.65	2.07E-03
ZNF787	1.50	2.90E-02
ZNF804A	0.58	1.18E-02
ZNF804B	0.56	3.59E-04
ZNF812P	0.62	2.85E-02
ZNF814	1.81	1.55E-04
ZNF826P	0.56	6.46E-04
ZNF847P	0.60	4.42E-02
ZNF852	1.60	1.16E-02
ZNF862	1.51	1.12E-02
ZNF865	1.94	3.83E-03
ZPBP	0.52	1.32E-02
ZPLD1	0.61	5.82E-03
ZRANB2-AS2	0.63	1.58E-03
ZSCAN25	1.65	2.62E-03
ZSCAN30	1.54	9.64E-03

Gene ID	Fold Change: AF+HF RA versus NF RA	FDR
ZSWIM8	1.67	2.51E-03
ZXDC	1.78	1.05E-03

Cardiomyocyte nuclei (CMN) were sorted from the right atria (RA) of non-failing human hearts (NF, n = 3) and failing hearts with a history of atrial fibrillation (AF+HF, n = 5). RNA was isolated and RNA-sequencing was performed from these samples. Genes with a linear fold change ≤ 0.667 and ≥ 1.5 with a false discovery rate (FDR) < 0.05 were considered significant and are included in the table.

Table S5. Differentially expressed RNA-sequencing transcripts from human atrial fibrillation with heart failure LA cardiomyocyte nuclei compared to non-failing LA cardiomyocyte nuclei

Gene ID	Fold Change: AF+HF LA versus NF LA	FDR
A1CF	2.53	3.82E-04
AACS	0.66	3.64E-03
AASS	3.05	4.49E-07
AB015752.1	1.55	1.90E-02
ABCA1	0.53	5.88E-03
ABCA10	0.45	9.53E-09
ABCA2	0.52	6.90E-04
ABCA9	0.48	3.89E-05
ABCA9-AS1	0.29	6.79E-05
ABCB1	0.57	7.29E-03
ABCB8	0.55	2.64E-03
ABCC9	1.95	2.36E-05
ABCG1	0.34	1.98E-09
ABLIM2	0.43	9.64E-07
AC002384.2	0.45	2.51E-02
AC002463.1	3.99	2.10E-05
AC004540.1	0.54	2.29E-03
AC004834.1	0.65	1.06E-02
AC005037.1	1.59	8.78E-03
AC005162.3	0.52	4.17E-02
AC005394.2	0.33	7.70E-05
AC005842.1	0.57	2.00E-02
AC005865.2	0.40	8.90E-05
AC006369.1	2.62	1.86E-02
AC007220.1	0.58	3.45E-02
AC007376.2	1.73	4.76E-02
AC007382.1	1.74	1.41E-02
AC007389.1	1.52	2.11E-02
AC007603.3	3.36	5.78E-03
AC007906.2	0.56	2.81E-02
AC007923.4	0.59	1.79E-02
AC008050.1	2.06	2.76E-02
AC008575.1	1.52	1.35E-02
AC008691.1	0.58	5.59E-04
AC008780.1	0.38	7.72E-05

Gene ID	Fold Change: AF+HF LA versus NF LA	FDR
AC008991.1	0.46	4.99E-03
AC009093.8	0.52	3.39E-03
AC009262.1	0.59	3.67E-02
AC009403.2	0.60	1.31E-02
AC009502.1	1.69	4.27E-02
AC010880.1	1.75	4.77E-02
AC010997.3	0.53	1.54E-02
AC011247.1	0.44	3.73E-05
AC011290.1	0.62	2.92E-02
AC011346.1	2.09	2.66E-03
AC011447.3	0.56	9.20E-05
AC011450.1	0.60	9.39E-03
AC011477.1	0.51	2.89E-02
AC012038.1	3.20	1.73E-05
AC012414.5	0.51	6.16E-04
AC012494.1	0.39	1.75E-02
AC013565.1	0.63	4.09E-02
AC015689.1	0.37	4.59E-04
AC015813.2	1.55	3.84E-04
AC016831.5	2.25	1.56E-04
AC017101.1	1.72	1.81E-02
AC018467.1	0.25	8.22E-06
AC018647.1	1.77	1.87E-02
AC019209.3	2.58	6.34E-03
AC019211.1	2.08	2.30E-02
AC020912.1	0.51	8.29E-03
AC021231.1	1.53	7.74E-03
AC021351.1	3.95	2.89E-03
AC021683.2	0.24	7.82E-09
AC022113.1	0.32	5.72E-04
AC022182.1	0.63	1.56E-02
AC022784.1	0.56	9.79E-03
AC024257.1	0.67	3.81E-02
AC024603.1	0.54	5.92E-04
AC024901.1	0.59	1.09E-04

Gene ID	Fold Change: AF+HF LA versus NF LA	FDR
AC025263.1	0.43	2.66E-03
AC025272.1	0.64	3.59E-02
AC025470.2	0.18	4.04E-15
AC025524.2	0.40	4.23E-03
AC025741.1	1.93	8.50E-04
AC025809.1	0.59	5.22E-03
AC026116.1	1.73	5.20E-03
AC027117.2	1.75	4.44E-04
AC044839.1	0.47	3.60E-03
AC058791.1	1.95	4.65E-03
AC063952.1	2.97	6.73E-03
AC068481.1	0.59	2.50E-02
AC068587.6	0.58	1.48E-03
AC073023.1	1.87	2.03E-02
AC073585.1	0.48	4.82E-02
AC079062.1	0.55	4.41E-02
AC079296.1	0.49	2.60E-03
AC079340.1	0.53	2.97E-02
AC079760.1	1.62	4.35E-03
AC079943.2	0.58	9.89E-03
AC083800.1	3.85	3.50E-05
AC083902.2	0.29	4.44E-03
AC084064.1	0.44	2.14E-02
AC084879.1	0.60	5.56E-04
AC087379.2	0.42	2.45E-02
AC087457.1	0.51	3.84E-03
AC087482.1	0.57	1.52E-03
AC087516.2	4.70	5.73E-05
AC087565.3	0.61	1.67E-03
AC087672.3	0.56	6.45E-03
AC090155.1	0.35	1.00E-02
AC090193.1	0.47	1.70E-04
AC090409.1	1.72	4.01E-02
AC090518.1	1.61	4.86E-02
AC090771.2	2.02	1.62E-02
AC090809.1	0.63	1.44E-02
AC091182.1	0.47	3.35E-03
AC091588.2	2.59	1.72E-04
AC091588.3	1.74	2.66E-02
AC092042.3	0.61	1.52E-02
AC092053.3	1.79	1.33E-02
AC092164.1	0.66	3.57E-02
AC092167.1	0.46	6.63E-04
AC092573.2	2.10	3.47E-02
AC092598.1	0.51	5.88E-03
AC092769.1	0.34	1.72E-07
AC093151.3	2.43	8.45E-03
AC093607.1	2.60	1.83E-02
AC095032.1	1.53	1.27E-02
AC096666.1	0.56	3.23E-02
AC096669.1	0.31	3.40E-04
AC098650.1	1.55	2.73E-02
AC098850.4	5.22	2.33E-03
AC098864.1	12.01	8.93E-06
AC099329.3	1.87	5.79E-05
AC099518.3	0.48	1.02E-04
AC104170.1	1.60	4.29E-03
AC104461.1	1.61	5.10E-03
AC104574.2	0.66	3.38E-03
AC104806.2	1.90	1.72E-07
AC104836.1	1.83	6.14E-05
AC105105.5	0.55	3.70E-02
AC105760.1	0.50	3.45E-03
AC106053.1	0.61	1.02E-02
AC106729.1	1.77	3.64E-02
AC107027.3	1.77	1.60E-03
AC107068.2	0.17	9.63E-05
AC107208.1	7.24	1.83E-08
AC107973.1	2.80	6.40E-03
AC107983.2	0.36	3.41E-02
AC108067.1	2.29	3.11E-02
AC108156.1	1.94	4.00E-03
AC108516.2	1.64	2.62E-02
AC110023.1	0.61	9.15E-03
AC112487.1	0.44	6.80E-03
AC113137.1	1.84	9.56E-03
AC113378.1	1.80	7.92E-03
AC113383.1	0.44	1.77E-05

Gene ID	Fold Change: AF+HF LA versus NF LA	FDR
AC116618.1	1.81	1.87E-03
AC117473.1	1.83	3.84E-02
AC118757.1	0.60	4.27E-02
AC119396.1	0.61	4.21E-02
AC119674.1	0.42	4.60E-04
AC123912.4	0.57	1.35E-02
AC126696.1	0.55	1.75E-02
AC128689.1	0.63	2.74E-02
AC131532.1	0.54	3.13E-02
AC131956.2	0.53	8.90E-03
AC137579.1	1.71	3.30E-02
AC137579.2	0.46	3.37E-03
AC138512.1	0.53	2.08E-03
AC138904.3	0.17	3.72E-12
AC244517.2	0.56	6.75E-04
AC244669.1	2.34	3.40E-04
AC246817.1	0.42	4.47E-04
AC246817.2	0.52	3.81E-03
ACCS	0.63	6.30E-03
ACOX3	0.65	6.06E-03
ACSF2	0.58	3.51E-03
ACSS2	0.61	1.49E-04
ACSS3	2.12	1.81E-05
ACTG2	0.51	2.70E-02
ACTN1	0.45	5.54E-06
ACTN4	0.41	2.32E-09
ACVR2A	1.53	5.98E-03
ACVRL1	0.66	1.78E-02
ADA	0.45	6.99E-05
ADA2	0.58	1.11E-03
ADAM11	0.32	3.34E-07
ADAM19	0.33	9.76E-07
ADAMTS10	0.45	9.48E-05
ADAMTS12	0.62	6.79E-03
ADAMTS14	0.45	4.96E-04
ADAMTS15	0.45	1.31E-04
ADAMTS17	0.49	1.11E-03
ADAMTS2	0.65	1.00E-02
ADAMTS7	0.49	4.17E-03

Gene ID	Fold Change: AF+HF LA versus NF LA	FDR
ADAMTS7P1	0.38	2.64E-03
ADAMTSL2	0.48	8.86E-03
ADAMTSL4-AS1	1.94	1.49E-03
ADAP2	0.57	2.21E-04
ADCY1	0.47	8.57E-05
ADCY3	0.43	3.43E-09
ADCY4	0.56	5.82E-03
ADCY5	0.41	2.15E-07
ADD2	0.58	8.65E-03
ADGRB3	0.65	8.88E-03
ADGRF5	0.55	3.19E-06
ADH1B	0.33	9.00E-06
ADM	3.26	1.01E-03
ADPRHL2	0.61	4.07E-02
ADSSL1	0.64	7.86E-03
AF064858.1	0.52	4.17E-03
AF121897.1	0.44	7.17E-03
AFAP1L2	0.50	3.88E-05
AFF3	0.51	1.63E-04
AHI1	1.71	7.74E-05
AICDA	0.42	2.71E-02
AIF1L	0.45	7.98E-05
AIFM2	0.65	3.19E-03
AJ009632.2	0.62	2.35E-02
AKR1C2	0.57	4.66E-03
AKR1C6P	0.65	2.51E-02
AKR1C7P	0.50	3.41E-02
AKR1C8P	0.46	7.49E-03
AKR1E2	0.65	3.75E-03
AL022068.1	1.61	2.73E-02
AL049775.2	0.54	1.26E-02
AL049874.3	0.63	4.35E-02
AL078590.2	3.54	7.11E-11
AL078590.3	2.79	1.56E-09
AL080248.1	1.85	2.85E-03
AL110292.1	0.42	2.45E-02
AL121772.1	0.55	9.17E-03
AL121936.1	0.43	3.00E-03

Gene ID	Fold Change: AF+HF LA versus NF LA	FDR
AL133417.1	5.38	1.83E-05
AL135936.1	1.81	6.80E-03
AL136164.2	1.73	3.40E-04
AL137058.2	1.80	8.76E-04
AL137145.2	0.55	3.06E-04
AL137224.1	1.94	4.33E-02
AL138737.1	0.61	1.07E-02
AL157832.3	0.52	3.18E-03
AL158152.2	1.53	1.34E-03
AL162632.1	0.45	1.50E-02
AL353803.2	2.60	9.27E-03
AL355596.1	2.36	1.13E-04
AL356258.1	0.51	4.12E-03
AL357314.1	0.55	3.61E-02
AL357833.1	0.20	5.82E-06
AL358394.3	1.86	3.94E-02
AL359745.1	0.41	4.44E-02
AL365255.1	0.45	4.57E-06
AL390036.1	0.61	4.74E-02
AL390334.1	0.57	3.89E-02
AL390860.1	1.69	2.94E-02
AL445307.1	1.96	7.80E-03
AL445584.2	0.44	3.50E-06
AL451069.1	0.32	1.32E-04
AL512625.3	3.69	1.04E-03
AL513164.1	1.97	5.14E-04
AL513318.2	0.55	2.45E-02
AL589843.1	0.62	2.40E-02
AL589935.1	1.52	1.24E-02
AL590560.1	0.49	4.60E-03
AL590648.3	1.93	2.22E-02
AL590666.2	0.56	1.59E-02
AL591178.1	1.91	8.50E-04
AL591242.1	1.56	3.86E-02
AL591543.1	0.28	3.37E-11
AL592287.1	0.65	1.48E-02
AL596220.1	1.73	1.30E-03
ALDH1B1	0.58	8.60E-03
ALDH1L1	1.64	2.10E-03

Gene ID	Fold Change: AF+HF LA versus NF LA	FDR
ALDH1L1-AS2	1.61	2.44E-02
ALDOA	0.65	1.51E-02
ALDOB	2.59	3.00E-04
ALOX12P2	0.53	2.19E-02
ALOX15	7.82	2.71E-05
ALOX5	0.63	9.50E-03
ANKRA2	1.58	2.69E-03
ANKRD27	0.65	1.70E-03
ANKRD29	0.35	4.87E-08
ANKRD45	0.12	2.99E-05
ANO1	0.64	2.22E-02
ANO5	1.68	1.31E-04
ANP32AP1	1.61	2.38E-03
ANPEP	0.51	1.56E-02
ANTXRL	0.44	4.42E-04
ANXA13	0.34	2.63E-05
ANXA3	0.61	8.45E-03
ANXA8L1	0.36	3.83E-05
AP001596.1	0.40	2.19E-02
AP002373.1	2.27	2.73E-03
AP002518.2	2.41	4.82E-09
AP002989.1	2.36	2.62E-04
AP003031.2	0.54	4.13E-02
AP003059.2	0.55	1.15E-03
AP003063.1	0.64	3.23E-03
AP005121.1	0.40	1.71E-02
AP005242.1	0.47	4.32E-02
AP1AR	1.56	1.66E-03
AP2M1	0.61	9.64E-04
AP2S1	0.60	7.23E-03
AP3S1	1.63	8.18E-04
APBB1IP	0.46	5.68E-08
APC	1.51	2.02E-03
APCDD1	0.49	2.74E-05
APOD	0.24	5.79E-05
APOL2	0.60	2.50E-03
APOL3	0.45	7.22E-08
APOL4	0.29	4.89E-09
APPL2	1.57	1.31E-03

Gene ID	Fold Change: AF+HF LA versus NF LA	FDR
AQP7	0.54	3.72E-02
AQP7P1	0.33	4.29E-03
AREG	6.07	2.90E-03
ARHGAP22	0.39	1.29E-13
ARHGAP23	0.52	1.70E-05
ARHGAP24	1.79	6.14E-05
ARHGAP28	0.55	2.05E-04
ARHGAP30	0.61	1.29E-03
ARHGDIB	0.62	9.36E-03
ARHGEF1	0.54	2.12E-04
ARHGEF2	0.54	4.44E-04
ARHGEF3	0.65	1.50E-02
ARHGEF4	0.38	2.26E-05
ARL13B	1.50	2.10E-03
ARMC8	1.58	1.72E-04
ARNTL2-AS1	3.00	1.25E-03
ARPP21	0.59	2.46E-03
ARRB1	0.40	9.77E-09
ARRB2	0.59	6.23E-03
ARRDC4	1.86	3.24E-02
ART1	0.34	8.50E-04
ASAH2B	1.91	8.43E-05
ASAP3	0.62	1.36E-03
ASB10	0.34	3.01E-05
ASB2	0.31	2.62E-04
ASIC1	0.56	2.30E-02
ATAD2	2.49	5.65E-05
ATG2A	0.63	1.24E-03
ATP10A	1.68	8.12E-04
ATP1A3	0.43	9.95E-06
ATP2A3	0.61	3.26E-02
ATP2B4	0.51	2.19E-03
ATP8B4	0.61	2.52E-03
ATRNL1	2.02	1.51E-04
BAALC	0.60	4.52E-02
BAAT	1.95	7.96E-04
BAMBI	2.53	2.44E-03
BBS10	1.75	1.67E-02
BBS12	2.21	4.42E-04

Gene ID	Fold Change: AF+HF LA versus NF LA	FDR
BBX	1.56	8.59E-04
BCAM	0.54	2.62E-02
BCAR3	0.61	1.71E-03
BCKDK	0.64	9.16E-03
BEAN1	2.54	2.96E-04
BEST3	0.29	7.62E-11
BIN1	0.31	2.34E-09
BLNK	0.42	7.60E-06
BLVRB	0.65	7.14E-03
BLZF1	1.55	5.06E-04
BMP1	0.63	1.33E-02
BMP10	50.67	3.66E-03
BMP7	0.40	2.51E-05
BMT2	1.54	1.24E-02
BOC	0.52	4.38E-06
BST1	0.61	9.54E-03
BTBD2	0.55	1.34E-03
BTG2	2.98	2.00E-02
BTG3	1.60	1.43E-03
BTN2A1	0.61	4.77E-04
BTN2A3P	0.49	3.83E-02
BTN3A1	0.51	3.22E-03
BTNL3	0.31	2.27E-04
BTNL8	0.65	2.29E-02
BTNL9	0.40	9.60E-11
BX640514.1	0.29	5.89E-04
BYSL	1.51	7.98E-03
BZW1	1.51	3.04E-02
C10orf107	0.60	3.87E-02
C10orf126	1.87	2.49E-02
C10orf128	0.61	1.20E-02
C12orf40	1.90	3.49E-03
C12orf65	0.64	4.60E-03
C12orf77	2.81	4.69E-02
C14orf132	2.67	2.11E-02
C14orf144	0.27	1.23E-05
C14orf166	1.51	9.09E-03
C15orf52	0.50	6.90E-04
C17orf53	0.55	9.15E-03

Gene ID	Fold Change: AF+HF LA versus NF LA	FDR
C19orf53	0.64	1.42E-03
C1orf21	1.52	6.43E-03
C1QB	0.46	2.18E-03
C1QC	0.49	2.13E-02
C1QL3	1.52	4.52E-02
C1QTNF7	1.82	1.84E-02
C1RL	0.59	3.15E-03
C22orf34	0.65	1.75E-02
C22orf46	0.63	1.92E-02
C2orf71	0.44	5.09E-04
C3orf52	3.29	2.15E-05
C4BPB	0.48	1.54E-02
C4orf47	0.54	6.99E-05
C5orf46	4.08	2.07E-03
C5orf47	2.23	1.20E-02
C5orf51	1.61	8.32E-04
C8orf49	0.63	4.19E-03
C9orf3	0.59	1.76E-03
CA8	1.88	2.06E-03
CABLES1	0.53	2.14E-04
CABP1	0.35	4.32E-09
CACNA1D	0.46	2.27E-04
CACNA1E	0.54	4.29E-02
CACNA1G	0.25	1.61E-09
CACNA2D2	0.49	1.25E-03
CACNA2D4	0.63	2.73E-02
CALB1	2.48	1.55E-06
CALCB	0.65	4.55E-03
CALD1	0.32	6.24E-06
CALR	0.55	1.95E-03
CALR4P	2.35	1.10E-02
CAMK2A	0.44	4.67E-05
CAMK2G	0.62	1.40E-03
CAMKK1	0.57	3.64E-02
CAMTA2	0.64	1.27E-03
CAND2	0.53	1.56E-04
CAPN1	0.64	2.86E-03
CAPN12	0.59	3.16E-02
CAPN5	0.52	9.05E-03

Gene ID	Fold Change: AF+HF LA versus NF LA	FDR
CAPN7	1.51	1.02E-03
CAPNS1	0.63	2.45E-03
CARMN	0.33	6.04E-17
CASQ1	0.39	4.85E-04
CASQ2	0.60	1.07E-03
CATSPERB	0.59	1.65E-04
CAV1	0.44	1.57E-04
CBFA2T3	0.45	1.49E-03
CBFB	1.89	4.57E-06
CBLB	2.30	3.29E-10
CCDC110	0.49	1.21E-03
CCDC127	0.55	8.65E-03
CCDC181	1.58	1.19E-02
CCDC190	0.41	1.45E-04
CCDC6	1.54	5.49E-04
CCDC70	0.54	2.73E-02
CCDC93	1.83	5.23E-05
CCM2L	0.50	1.26E-04
CCNH	1.53	2.98E-03
CCT2	0.27	3.67E-11
CD109	1.56	2.20E-03
CD200	0.64	3.64E-02
CD200R1	0.57	3.81E-02
CD209	0.42	7.23E-04
CD274	2.34	3.68E-04
CD33	0.50	3.38E-03
CD34	0.50	4.68E-04
CD38	0.55	8.71E-04
CD48	0.46	3.33E-02
CD58	0.61	3.02E-05
CD82	0.56	3.18E-03
CDC25B	0.62	1.83E-02
CDC37	0.56	3.12E-06
CDC37L1	1.51	2.64E-03
CDC6	0.41	3.36E-04
CDCA2	0.48	9.15E-06
CDH20	1.72	1.23E-02
CDH22	0.43	1.08E-04
CDH26	0.60	7.72E-05

Gene ID	Fold Change: AF+HF LA versus NF LA	FDR
CDH5	0.59	2.61E-03
CDH6	0.53	2.26E-03
CDHR3	0.54	1.43E-06
CDK14	1.54	2.95E-03
CDK18	0.55	3.51E-05
CDKL2	0.63	6.19E-03
CDKN1A	1.93	1.18E-02
CDO1	0.55	1.15E-02
CDON	0.66	2.27E-02
CEBPD	1.95	2.06E-03
CERS3-AS1	0.30	1.20E-06
CERS4	0.49	5.66E-04
CES1	0.35	1.25E-03
CES4A	1.59	3.07E-02
CETN4P	1.81	6.56E-03
CETP	0.46	2.54E-02
CFAP221	1.62	1.81E-02
CFAP46	0.45	1.84E-04
CFAP61	2.82	4.48E-04
CFD	0.43	2.03E-02
CGNL1	0.59	1.07E-02
CHCHD4	2.00	9.96E-05
CHD3	0.65	7.60E-03
CHDH	0.50	6.41E-04
CHGB	4.45	7.03E-03
CHID1	0.64	4.99E-03
CHMP2A	0.62	8.97E-03
CHPT1	1.63	1.11E-03
CHST11	0.64	2.89E-02
CHST3	1.79	1.26E-05
CIB2	0.59	2.36E-02
CILP	0.31	4.38E-06
CKAP2	1.58	2.20E-02
CKB	0.53	1.71E-02
CKM	0.42	8.45E-06
CLCN1	0.61	3.22E-02
CLEC7A	0.62	4.88E-02
CLECL1	0.54	3.64E-02
CLGN	1.89	1.56E-04

Gene ID	Fold Change: AF+HF LA versus NF LA	FDR
CLMP	0.55	3.02E-04
CLSTN2	0.51	3.82E-04
CLSTN3	0.61	3.18E-03
CLUH	0.66	4.33E-03
CLVS1	0.60	1.86E-02
CMKLR1	0.46	2.20E-06
CNGB1	1.65	2.35E-02
CNKSR3	0.52	4.51E-04
CNMD	1.62	1.08E-02
CNN1	0.31	2.83E-03
CNN2	0.56	7.15E-03
CNNM4	0.52	2.44E-04
CNPPD1	0.64	4.99E-03
CNTFR	0.48	1.02E-04
CNTNAP3	0.51	1.95E-03
CNTNAP3B	0.44	9.91E-06
CNTNAP3P2	0.52	1.32E-03
CNTROB	0.48	1.78E-05
COL15A1	0.37	1.96E-09
COL18A1	0.64	2.41E-02
COL27A1	0.25	2.96E-11
COL4A2	0.57	1.17E-03
COL5A1	0.53	1.60E-03
COL5A3	0.42	1.27E-05
COL6A1	0.50	8.80E-04
COL6A2	0.62	2.04E-02
COL6A3	0.62	1.05E-02
COL8A1	0.39	2.62E-02
COL9A1	2.58	9.74E-03
COLGALT1	0.63	6.84E-04
COQ8B	0.65	2.56E-02
CORO2A	0.58	3.00E-02
COTL1	0.60	5.20E-03
COX4I1	0.65	5.73E-04
COX5A	0.63	2.03E-04
COX6B1	0.64	8.18E-04
COX7A1	0.52	3.90E-03
CPA4	2.74	3.45E-03
CPAMD8	0.52	2.04E-06

Gene ID	Fold Change: AF+HF LA versus NF LA	FDR
CPB2-AS1	0.26	9.96E-03
CPEB4	2.12	1.51E-05
CPN1	0.65	3.96E-02
CPNE2	0.63	3.22E-03
CPVL	0.61	1.81E-02
CR1	0.41	2.64E-05
CRACR2A	0.64	3.29E-02
CRAT	0.66	4.80E-03
CRB1	1.63	4.52E-03
CREB3L1	0.56	2.27E-04
CREB5	1.92	2.23E-02
CRHR2	0.49	1.22E-03
CRIP2	0.63	1.95E-02
CROCCP3	0.66	2.51E-02
CRY1	1.59	4.87E-03
CRYBA1	1.50	2.54E-02
CRYGS	1.51	2.32E-02
CRYZ	1.60	1.88E-02
CSF1R	0.58	2.85E-04
CSF2RB	0.34	1.57E-06
CSF3R	0.57	1.32E-02
CSRNP1	2.57	3.36E-04
CSRP1	0.56	4.75E-02
CSRP3	0.45	7.57E-08
CSTB	0.57	4.27E-03
CTHRC1	2.50	2.26E-07
CTIF	0.59	3.01E-03
CTNND1	0.65	1.53E-03
CTNS	0.57	1.01E-03
CTSA	0.61	4.44E-03
CTSB	0.63	1.75E-02
CTSL3P	0.42	1.30E-04
CTXND1	2.64	1.39E-03
CUZD1	0.48	4.46E-02
CX3CR1	0.34	1.85E-03
CXCL12	0.40	2.79E-05
CXXC4	1.56	5.89E-03
CYB5D2	0.66	9.88E-03
CYB5R3	0.61	1.68E-04

Gene ID	Fold Change: AF+HF LA versus NF LA	FDR
CYP4F12	0.30	1.56E-06
CYTH4	0.35	4.38E-06
DAB2IP	0.52	2.21E-05
DACH1	0.52	3.34E-03
DACT1	0.40	5.35E-05
DACT2	0.40	2.00E-03
DAGLA	0.58	1.90E-02
DAND5	0.51	5.51E-03
DAPK3	0.65	7.30E-03
DAXX	0.66	1.97E-02
DCLK1	0.50	3.33E-07
DCTN1	0.63	1.33E-03
DDA1	0.65	1.64E-02
DDX39A	0.59	1.54E-04
DEAF1	0.66	4.04E-02
DEDD2	0.66	2.23E-02
DENND4B	0.54	2.66E-05
DEPDC4	0.66	4.01E-02
DERA	1.53	7.19E-04
DES	0.52	2.97E-04
DGAT1	0.62	9.09E-03
DGAT2	0.36	1.79E-03
DGKB	0.55	2.93E-03
DHRS4-AS1	0.56	1.20E-04
DHX15	1.56	1.49E-03
DIAPH3	2.48	1.31E-02
DIRAS1	0.57	2.29E-02
DISC1FP1	0.43	8.39E-04
DLG4	0.65	9.46E-03
DMAP1	0.65	1.05E-02
DMPK	0.60	2.30E-02
DMTN	0.64	6.54E-03
DNAJB2	0.58	3.29E-03
DNALI1	0.66	1.57E-02
DNER	2.24	1.93E-02
DNM1	0.61	8.59E-04
DNTT	2.18	9.54E-03
DOCK2	0.64	2.09E-03
DOCK5	1.69	4.86E-03

Gene ID	Fold Change: AF+HF LA versus NF LA	FDR
DOCK6	0.61	2.42E-03
DOCK8	0.62	1.35E-03
DOK6	0.59	2.38E-04
DOPEY2	0.64	2.17E-03
DPYSL4	0.29	2.96E-04
DRICH1	0.62	4.69E-02
DSC3	0.34	4.67E-05
DSCAML1	0.61	6.73E-04
DTL	0.65	3.69E-02
DTX3	0.64	1.77E-02
DTX4	0.51	5.23E-05
DUS1L	0.61	4.76E-02
DUSP10	0.42	1.26E-07
DUSP8	0.61	1.93E-02
EBF1	0.48	8.19E-06
EBF2	0.31	1.64E-19
EBF3	0.49	1.72E-07
ECH1	0.58	1.00E-03
ECHDC3	1.57	1.81E-03
ECSIT	0.65	4.19E-03
EDIL3	0.46	7.84E-06
EDNRA	1.56	2.11E-02
EEF1A2	0.64	2.90E-02
EFCAB12	2.76	3.74E-04
EFCC1	0.49	4.60E-04
EGFLAM	0.49	2.26E-06
EGFR	1.65	3.27E-02
EGR1	5.40	7.04E-04
EGR2	2.06	1.37E-03
EHD1	0.52	2.87E-03
EHD2	0.64	4.15E-02
EHF	0.55	2.81E-02
EIF4EBP1	1.63	4.91E-02
ELFN1	0.51	4.61E-03
ELL2	2.46	3.36E-04
ELMOD1	1.97	7.16E-04
ELMOD2	1.83	7.77E-06
ELN	0.41	5.26E-04
EMCN	0.61	9.35E-03
EML1	0.56	7.84E-06
ENC1	0.48	3.01E-04
ENGASE	0.62	3.39E-02
ENPP7P10	0.62	5.09E-03
EPB41L1	0.57	1.54E-04
EPHA7	2.21	4.78E-07
EPHB2	0.46	4.00E-06
EPHX1	0.65	1.35E-02
EPM2AIP1	1.78	3.60E-05
ERBB3	0.39	9.05E-05
ERICH6B	1.66	1.43E-02
ERRFI1	1.86	2.32E-04
ERVK13-1	1.60	2.43E-03
ESRRB	0.58	1.29E-03
ETNPPL	0.46	4.22E-02
ETV5	2.00	1.84E-03
ETV6	0.59	1.28E-02
EXPH5	0.53	2.40E-04
EXTL1	0.64	3.12E-02
F11	0.45	4.03E-03
F13A1	0.35	1.02E-12
FAAH	0.61	3.01E-02
FADS2	0.45	2.87E-06
FADS3	0.55	1.22E-04
FAH	0.65	6.28E-03
FAIM2	0.40	1.78E-03
FAM105A	0.48	1.52E-04
FAM111A	0.66	1.84E-02
FAM117A	0.50	2.47E-05
FAM129A	0.49	5.61E-03
FAM153A	0.24	4.83E-03
FAM161A	2.01	5.58E-04
FAM167A-AS1	0.43	3.19E-06
FAM189A1	0.36	8.05E-05
FAM20A	0.52	1.03E-03
FAM212B	3.23	1.99E-11
FAM214A	2.06	4.02E-07
FAM21FP	0.56	3.36E-02
FAM221A	1.58	7.34E-03

Gene ID	Fold Change: AF+HF LA versus NF LA	FDR	Gene ID	Fold Change: AF+HF LA versus NF LA	FDR
FAM24B	0.54	2.12E-02	*FLI1*	0.55	2.37E-06
FAM35A	0.59	7.56E-03	*FLII*	0.57	2.58E-05
FAM3C	1.64	4.02E-03	*FLJ21408*	0.65	2.62E-02
FAM46A	1.67	3.70E-02	*FLNB*	0.48	1.05E-04
FAM49B	0.66	3.22E-03	*FLNC*	0.39	7.67E-05
FAM57A	0.62	4.64E-02	*FLNC-AS1*	0.28	8.98E-06
FAM66A	0.59	2.70E-02	*FLOT1*	0.56	3.50E-05
FAM78A	0.48	5.19E-05	*FNDC5*	0.52	5.93E-03
FAM81A	0.62	7.75E-04	*FNDC7*	0.55	2.97E-02
FAM89A	0.62	4.82E-02	*FNIP2*	1.53	2.55E-02
FAR1	1.64	1.70E-05	*FOSL2*	1.84	2.13E-02
FAXC	0.50	4.32E-02	*FOXM1*	0.53	4.60E-03
FBLIM1	0.63	7.89E-03	*FOXO1*	1.61	1.29E-03
FBLN1	0.42	4.82E-05	*FOXO3*	2.08	4.48E-07
FBLN2	0.51	1.39E-04	*FREM1*	0.38	1.95E-11
FBLN5	0.63	1.65E-03	*FRG1BP*	1.79	1.56E-04
FBN1	0.56	1.87E-03	*FRG1CP*	1.67	1.22E-03
FBN2	0.38	1.22E-02	*FRG1DP*	1.98	7.67E-05
FBXO17	0.56	1.51E-02	*FRG1JP*	1.70	1.44E-02
FBXO39	2.29	1.51E-02	*FRMD6*	0.66	7.92E-03
FCGBP	0.46	2.72E-04	*FRS2*	0.60	2.64E-04
FCGRT	0.54	6.76E-04	*FSD2*	0.48	2.45E-04
FEM1C	1.93	9.12E-04	*FTL*	0.60	2.02E-02
FERMT1	0.50	2.99E-05	*FYB1*	0.53	9.69E-05
FEZ1	0.62	2.86E-03	*FYCO1*	0.64	2.39E-03
FGD2	0.50	5.25E-04	*FZR1*	0.66	1.49E-02
FGD3	0.58	2.52E-04	*GALNT15*	0.48	3.83E-05
FGD4	2.02	3.52E-04	*GALNT17*	3.75	4.87E-03
FGD5	0.55	9.95E-06	*GALNT5*	2.14	1.75E-02
FGF1	0.65	4.92E-02	*GAPDH*	0.55	4.46E-03
FGF10	0.55	3.24E-03	*GAS1RR*	0.57	1.01E-03
FGF10-AS1	0.37	7.19E-03	*GATA3*	0.48	1.59E-03
FGF12	0.50	2.05E-03	*GBP2*	0.65	3.96E-02
FGF18	0.48	1.73E-03	*GBX1*	0.32	2.25E-04
FGFR1	0.50	4.82E-06	*GDA*	0.48	4.66E-03
FGFR2	0.27	1.02E-04	*GDF6*	0.21	1.22E-07
FHOD3	0.53	1.29E-03	*GDNF-AS1*	2.94	2.88E-05
FITM1	0.59	4.87E-02	*GDPD5*	0.55	5.30E-03
FKBP5	5.04	4.00E-17	*GEM*	0.35	5.56E-04

Gene ID	Fold Change: AF+HF LA versus NF LA	FDR
GEMIN4	0.51	3.50E-02
GFI1B	1.99	3.30E-02
GFRA1	0.66	2.27E-03
GFRA3	0.51	3.74E-03
GGA3	0.63	1.17E-03
GGT7	0.62	3.98E-03
GHRHR	1.81	3.36E-02
GIMAP2	0.47	1.63E-03
GIMAP4	0.50	1.72E-05
GIMAP8	0.63	1.83E-02
GJB7	1.78	2.08E-03
GLDN	1.75	4.14E-02
GLI3	0.64	6.68E-03
GLOD4	0.61	6.96E-03
GLP1R	0.25	1.37E-03
GLRB	1.86	3.83E-05
GLS2	2.50	3.27E-04
GMNC	0.58	1.25E-02
GNAI2	0.64	2.06E-03
GNAO1	0.58	3.25E-03
GNB4	1.59	1.80E-02
GNB5	0.59	8.48E-03
GNG12	1.75	2.95E-03
GNG2	0.58	2.79E-04
GNPDA2	1.54	9.61E-03
GOLT1B	1.91	9.30E-05
GPA33	0.58	1.90E-02
GPC5	1.61	3.01E-02
GPM6A	1.64	3.27E-02
GPNMB	0.48	9.36E-04
GPR137C	1.66	1.54E-03
GPRC5B	0.60	1.43E-03
GPRC5D-AS1	1.51	3.02E-02
GPSM1	0.54	1.03E-02
GPT2	0.48	1.90E-03
GPX3	0.57	1.02E-02
GRAMD1B	0.58	2.59E-04
GRIA1	0.45	1.56E-02
GRIK3	0.46	2.01E-05
GRIN2A	0.43	7.23E-04
GRIP2	0.54	2.79E-04
GRXCR2	0.08	1.02E-04
GSG1L	0.39	1.18E-03
GSTA8P	3.52	2.89E-03
GSTM5	0.30	5.06E-04
GTF2IP7	0.44	3.08E-03
GUCY1A2	0.64	2.29E-02
GUCY1A3	2.18	1.10E-03
GULOP	0.65	1.90E-02
GUSBP11	0.64	7.56E-03
H1F0	0.59	8.56E-03
HAAO	0.56	8.38E-03
HCG27	0.56	4.04E-02
HDC	2.23	1.09E-02
HECTD3	0.66	5.82E-03
HELLPAR	0.64	2.79E-02
HENMT1	0.45	8.97E-03
HEPACAM	0.38	5.55E-03
HES1	2.39	2.66E-03
HEXDC	0.56	1.58E-04
HFE	1.62	4.04E-02
HFE2	0.58	5.25E-03
HID1	0.49	4.78E-03
HIP1	0.56	1.35E-04
HIRA	0.45	1.43E-05
HIST2H2BE	1.78	1.25E-02
HIVEP2	1.67	5.45E-03
HIVEP3	0.57	5.54E-03
HK2	0.64	4.06E-02
HLA-DMA	0.12	2.79E-04
HLA-DRB5	1.87	3.94E-02
HLX-AS1	0.48	1.52E-04
HMCES	0.60	1.25E-03
HMCN1	0.63	1.55E-02
HMCN2	0.52	6.81E-05
HOPX	3.35	8.31E-03
HRASLS5	0.59	5.76E-04
HRC	0.65	3.61E-02

Gene ID	Fold Change: AF+HF LA versus NF LA	FDR
HRH1	0.55	1.98E-03
HRH2	0.51	2.10E-03
HS1BP3	0.50	1.91E-02
HS3ST2	1.90	1.39E-03
HS3ST3A1	3.51	3.46E-02
HS6ST1	1.58	7.38E-04
HSD17B14	0.59	4.04E-02
HTR1E	1.67	2.04E-02
HTR4	0.29	8.58E-06
HTRA3	0.48	5.84E-04
HYAL4	4.24	5.22E-07
HYALP1	5.57	9.78E-06
HYOU1	0.54	1.48E-04
ICMT	0.56	4.76E-05
IDH2	0.38	1.02E-13
IFNG-AS1	3.18	1.34E-02
IFT122	1.69	5.85E-04
IGF1	0.32	1.72E-07
IGFBP4	0.57	8.56E-03
IGFBP5	0.33	9.33E-07
IGFN1	2.13	4.30E-02
IGSF11	2.16	1.63E-04
IGSF3	0.51	1.52E-06
IGSF9B	0.64	2.95E-02
IL10RA	0.41	8.28E-09
IL15RA	0.49	4.32E-06
IL1R2	1.92	6.80E-03
IL6ST	1.54	9.55E-04
ILVBL	0.61	9.85E-04
IMPA2	0.63	9.55E-04
INPP5J	0.44	3.74E-04
INPPL1	0.60	6.37E-04
INSC	0.61	1.36E-02
INTS11	0.63	7.17E-03
INTS6	1.53	5.77E-03
IP6K2	1.55	5.02E-03
IPO13	0.61	1.61E-04
IQSEC1	0.55	5.09E-05
ISOC1	0.61	2.26E-03

Gene ID	Fold Change: AF+HF LA versus NF LA	FDR
ITFG2-AS1	0.61	1.02E-03
ITGA3	0.55	9.14E-04
ITGAM	0.51	1.82E-05
ITGAV	1.70	4.70E-05
ITGB2	0.44	1.56E-04
ITGBL1	0.64	4.40E-03
ITIH5	0.49	1.41E-07
ITLN1	28.67	1.77E-04
ITPK1	1.74	2.55E-03
ITPKB	0.41	2.08E-13
ITPR3	0.54	1.27E-04
IVNS1ABP	1.89	4.08E-04
JAG1	0.66	1.71E-03
JAK1	1.59	3.33E-03
JAKMIP3	0.30	9.50E-06
JAML	0.66	3.58E-02
KANK2	0.63	1.98E-03
KANK4	0.66	3.93E-02
KAT2A	0.64	1.69E-02
KAZN	0.44	8.13E-07
KCNA4	5.37	5.07E-17
KCNAB2	1.74	5.33E-03
KCNIP2	0.57	3.46E-02
KCNJ8	3.53	7.21E-06
KCNK3	0.35	2.09E-07
KCNK5	0.33	1.92E-05
KCNN1	0.30	1.41E-07
KCNN2	0.47	1.71E-03
KCNT2	2.36	1.53E-03
KCNU1	0.54	2.37E-02
KCTD1	1.80	5.22E-04
KEAP1	0.61	3.59E-04
KHDRBS3	1.53	2.66E-03
KIAA0930	0.66	3.29E-02
KIAA1257	0.52	9.87E-03
KIAA1324L	1.65	2.22E-02
KIAA1522	0.59	2.55E-03
KIAA1586	1.55	4.19E-03
KIAA1671	0.59	1.57E-03

Gene ID	Fold Change: AF+HF LA versus NF LA	FDR
KIAA1755	0.51	1.13E-03
KIF1A	0.58	7.07E-04
KIF5C	1.62	2.11E-02
KIFAP3	2.24	1.71E-09
KIFC3	0.59	4.49E-03
KIRREL1	0.54	2.59E-05
KITLG	0.57	4.29E-03
KL	0.59	4.46E-03
KLB	0.63	8.32E-03
KLF15	2.37	4.79E-05
KLF9	2.32	2.54E-09
KLHDC8B	0.64	1.33E-02
KLHL11	1.52	4.29E-02
KLHL21	0.52	1.30E-04
KLHL3	1.95	2.74E-05
KLHL32	0.28	8.97E-06
KLHL36	1.98	1.57E-04
KLRA1P	1.69	2.72E-04
KSR1	0.55	2.45E-04
KYAT1	0.55	1.33E-04
L3MBTL2	0.62	2.30E-03
LAMA2	1.63	1.75E-02
LAMA3	1.79	1.62E-04
LAMA4	0.43	1.82E-04
LAMA5	0.47	1.66E-05
LAMB2	0.66	1.02E-02
LAMB3	0.61	3.24E-02
LAMC2	0.40	8.22E-08
LARP6	0.66	2.27E-03
LCAT	0.60	3.54E-02
LCP1	0.54	2.12E-03
LDB2	0.64	6.37E-03
LDHA	0.62	4.06E-02
LDLRAD4	1.85	2.27E-04
LEPR	2.04	6.55E-06
LGI2	0.47	4.95E-05
LHFPL6	0.61	7.69E-04
LHX4	0.54	1.34E-03
LHX6	0.53	4.04E-02

Gene ID	Fold Change: AF+HF LA versus NF LA	FDR
LIFR	1.58	1.00E-03
LILRB4	0.52	9.09E-03
LIMK1	0.63	1.83E-02
LIMS2	0.50	9.68E-06
LIN7A	2.44	4.49E-07
LINC00211	3.04	2.28E-05
LINC00299	0.51	2.18E-04
LINC00327	0.50	1.16E-03
LINC00443	3.78	2.02E-03
LINC00513	2.09	1.17E-04
LINC00519	2.09	3.29E-03
LINC00583	1.83	2.08E-02
LINC00598	0.60	2.08E-02
LINC00616	0.39	1.02E-04
LINC00639	0.47	1.54E-04
LINC00641	1.53	2.77E-04
LINC00649	0.62	2.35E-02
LINC00670	0.60	3.10E-02
LINC00691	3.63	4.25E-05
LINC00840	0.49	1.16E-03
LINC00847	0.63	1.21E-02
LINC00854	0.61	2.06E-02
LINC00871	0.55	2.89E-02
LINC00879	0.52	1.51E-02
LINC00886	1.65	7.51E-03
LINC00924	0.45	6.73E-04
LINC00939	0.59	2.69E-02
LINC00954	0.53	1.11E-02
LINC00964	0.65	4.37E-02
LINC00970	0.51	3.29E-05
LINC01032	1.72	3.56E-03
LINC01060	0.46	3.00E-03
LINC01091	1.87	1.70E-02
LINC01105	9.27	2.21E-06
LINC01107	0.57	1.85E-02
LINC01133	0.62	2.41E-02
LINC01151	0.52	3.30E-02
LINC01182	0.65	1.56E-02
LINC01197	0.58	2.58E-04

Gene ID	Fold Change: AF+HF LA versus NF LA	FDR
LINC01252	0.52	3.38E-03
LINC01355	1.51	2.14E-02
LINC01359	1.73	3.89E-03
LINC01375	0.64	3.71E-02
LINC01428	2.03	2.49E-04
LINC01446	2.46	2.36E-03
LINC01470	0.55	5.68E-03
LINC01476	0.58	1.37E-03
LINC01481	1.63	2.05E-02
LINC01482	0.48	2.75E-05
LINC01484	0.45	2.72E-03
LINC01505	1.67	1.06E-02
LINC01531	0.44	5.06E-04
LINC01550	0.53	6.79E-05
LINC01572	0.66	7.59E-03
LINC01600	0.60	2.09E-02
LINC01663	3.36	4.96E-02
LINC01681	0.62	2.48E-03
LINC01708	1.54	3.59E-02
LINC01732	0.41	9.66E-03
LINC01789	0.63	3.03E-02
LINC01807	0.43	5.20E-05
LINC01859	0.54	9.07E-03
LINC01876	0.50	1.50E-03
LINC01881	1.76	2.63E-05
LINC01885	0.46	1.79E-02
LINC01915	0.44	8.21E-03
LINC01942	0.23	7.96E-06
LINC02035	1.53	3.55E-02
LINC02082	1.71	3.96E-02
LINC02101	7.09	5.14E-04
LINC02102	0.58	4.27E-02
LINC02137	2.07	3.44E-02
LINC02206	0.53	3.00E-03
LINC02211	0.31	1.13E-02
LINC02284	0.48	7.44E-03
LINC02319	0.41	4.89E-03
LINC02355	1.61	2.74E-02
LINC02398	0.54	2.89E-05

Gene ID	Fold Change: AF+HF LA versus NF LA	FDR
LINC02426	1.79	2.44E-02
LINC02432	2.94	5.90E-04
LINC02436	1.78	4.20E-02
LINC02541	0.28	1.60E-04
LINC-PINT	2.26	2.30E-04
LIPC	0.59	1.66E-03
LITAF	0.57	8.48E-03
LMAN1L	0.27	5.52E-03
LMO2	0.61	4.34E-02
LMOD1	0.29	3.60E-06
LONRF1	1.55	4.17E-02
LOXL3	0.55	2.84E-03
LPA	0.66	2.39E-02
LPCAT2	0.41	1.51E-08
LPCAT4	0.40	7.09E-06
LRP1	0.53	1.02E-04
LRP1B	0.27	2.80E-05
LRP8	0.57	4.31E-04
LRRC1	1.75	1.28E-02
LRRC10	0.17	1.06E-20
LRRC20	0.59	3.07E-03
LRRC49	0.52	1.83E-02
LRRC6	2.72	1.72E-03
LRRC66	1.51	3.04E-02
LRRC74A	0.48	8.48E-05
LRRTM4	0.58	3.15E-02
LRSAM1	0.66	8.34E-03
LTBR	0.65	3.24E-02
LYN	0.63	1.21E-03
LZTS1	0.40	7.84E-06
MAATS1	0.60	3.91E-03
MACROD1	0.59	5.93E-03
MAF	0.47	7.84E-06
MAGOHB	1.54	6.73E-03
MAIP1	1.56	7.59E-03
MAL	0.42	1.31E-06
MAMDC2-AS1	0.49	2.40E-02
MAN1A1	1.95	6.98E-04
MAN1C1	0.56	5.72E-03

Gene ID	Fold Change: AF+HF LA versus NF LA	FDR
MAN2A2	0.65	1.38E-02
MAP1A	0.58	3.32E-02
MAP2K6	0.55	3.28E-04
MAP3K14	0.54	9.71E-05
MAPK3	0.51	8.32E-06
MAPK4	2.14	2.57E-02
MAPRE3	0.58	1.07E-03
MAPT	0.42	1.31E-05
MARC1	0.40	1.27E-05
MARCKS	0.55	4.58E-03
MARS	0.65	5.54E-03
MAST3	0.63	2.02E-02
MAST4	1.68	5.15E-04
MATN2	0.51	1.53E-04
MBD4	2.21	5.76E-04
MBNL2	1.52	1.91E-03
MBP	0.55	2.26E-04
MCF2L	0.60	5.80E-04
MCL1	2.00	1.49E-03
MCM3AP-AS1	0.61	1.96E-02
MCOLN2	0.48	5.09E-04
MDH2	0.66	3.55E-03
MDM1	2.44	1.10E-04
MED25	0.59	2.59E-04
MED30	1.80	1.37E-03
MEG3	0.66	1.58E-02
MEGF6	0.49	2.06E-03
MEGF8	0.64	5.20E-03
MEGF9	0.64	2.11E-02
MEOX1	0.48	2.52E-05
MEOX2	0.46	5.65E-05
MGLL	0.38	2.16E-16
MGST2	1.52	3.18E-03
MICAL1	0.66	3.97E-02
MICALCL	1.80	1.63E-04
MIIP	0.63	2.08E-02
MILR1	0.40	2.39E-05
MINDY1	0.65	6.26E-03
MINDY3	1.51	4.96E-03
MIR1-1HG	0.56	1.64E-02
MIR193BHG	1.66	3.68E-04
MIR31HG	2.42	6.57E-03
MIR34AHG	0.53	9.82E-05
MIR4432HG	0.64	1.88E-02
MKI67	0.49	4.45E-03
MKNK2	2.43	1.91E-03
MLXIPL	0.38	8.53E-05
MMP12	2.66	4.09E-02
MMP15	0.38	4.24E-06
MOB3B	1.75	3.19E-05
MPI	0.60	3.00E-03
MRAS	0.62	1.86E-02
MROH1	0.63	8.51E-03
MRPL18	0.59	8.57E-05
MRPL43	0.66	2.88E-02
MRPS25	1.52	8.60E-03
MRVI1	0.42	3.94E-03
MRVI1-AS1	0.53	3.59E-02
MS4A6A	0.63	5.20E-03
MSI1	0.65	1.35E-02
MSMO1	1.55	4.41E-02
MT1E	2.92	3.13E-04
MT-ATP6	0.39	4.50E-05
MT-ATP8	0.52	1.30E-02
MT-CO1	0.50	4.00E-03
MT-CO2	0.40	2.58E-04
MT-CO3	0.53	4.45E-03
MT-CYB	0.40	1.64E-05
MTHFD2L	1.62	1.66E-03
MTMR14	1.51	2.73E-03
MT-ND1	0.40	2.72E-04
MT-ND2	0.38	8.33E-05
MT-ND4	0.41	8.43E-05
MT-ND4L	0.41	1.25E-04
MT-ND5	0.46	2.39E-05
MT-ND6	0.53	1.43E-05
MT-TF	0.49	3.16E-02
MTURN	1.82	2.63E-04

Gene ID	Fold Change: AF+HF LA versus NF LA	FDR	Gene ID	Fold Change: AF+HF LA versus NF LA	FDR
MTUS1	1.56	3.01E-04	*NDFIP2*	1.59	9.50E-04
MUC16	4.97	1.52E-04	*NDNF*	0.50	2.16E-03
MUC3A	0.25	1.23E-12	*NDUFA3*	0.62	4.53E-04
MVP	0.60	2.60E-03	*NDUFAF8*	0.61	1.79E-02
MYBL1	0.59	1.78E-02	*NDUFB10*	0.55	5.14E-05
MYBPC3	0.61	4.70E-02	*NDUFB3*	0.59	1.04E-03
MYBPHL	0.39	2.15E-02	*NDUFB7*	0.62	9.21E-03
MYCT1	0.50	5.89E-04	*NEFM*	0.37	1.28E-02
MYH11	0.33	1.85E-05	*NEK11*	1.89	8.33E-05
MYH3	0.60	6.18E-03	*NES*	0.51	1.53E-03
MYH7B	0.47	1.04E-04	*NEU3*	1.66	2.90E-02
MYH9	0.41	2.75E-09	*NEURL1B*	0.66	1.81E-02
MYL2	0.25	1.15E-02	*NFASC*	1.69	2.58E-02
MYL4	0.50	5.96E-04	*NFKBIA*	1.93	1.36E-03
MYL7	0.43	5.39E-03	*NFKBIZ*	1.52	8.18E-03
MYLK4	0.49	9.57E-05	*NFXL1*	0.44	1.95E-02
MYLK-AS1	1.62	7.98E-03	*NHLRC2*	1.59	5.76E-04
MYO10	0.50	5.59E-05	*NID1*	0.51	7.41E-03
MYO1C	0.58	3.39E-05	*NKAIN3*	0.63	4.94E-03
MYO1E	0.56	1.45E-04	*NKD1*	0.55	1.15E-03
MYO1F	0.59	1.46E-03	*NLRC5*	0.59	8.70E-04
MYO5C	0.57	3.78E-04	*NLRP1*	0.65	2.29E-03
MYO7A	0.64	1.97E-02	*NLRP3*	3.85	1.07E-05
MYO7B	0.60	1.06E-02	*NLRP4*	1.92	3.09E-02
MYOC	0.49	2.80E-02	*NLRX1*	0.59	6.02E-04
MYOCD	0.63	1.18E-02	*NMI*	0.54	3.79E-03
MYOF	0.60	3.08E-02	*NMNAT1*	0.58	1.43E-03
NAALADL2	1.64	9.84E-04	*NMNAT2*	0.53	1.01E-02
NACC1	0.66	1.72E-02	*NMRK2*	0.51	3.55E-02
NAMPT	1.74	9.29E-03	*NMUR1*	0.13	2.52E-10
NAT1	0.53	1.82E-04	*NOC3L*	1.78	1.46E-03
NAV1	0.50	2.52E-05	*NOL4L*	0.53	8.58E-05
NAV3	0.41	1.04E-05	*NOTCH3*	0.56	2.16E-03
NBEAL2	0.63	1.13E-02	*NOX5*	0.46	4.62E-03
NBN	1.58	9.59E-04	*NPHP4*	1.61	7.00E-03
NBPF26	2.41	1.60E-04	*NPPB*	4.91	4.19E-03
NCKAP1L	0.48	1.17E-05	*NPSR1-AS1*	0.58	2.02E-03
NCKIPSD	1.75	8.69E-03	*NPTXR*	0.48	4.67E-05
NCR3LG1	1.70	3.22E-03	*NR2F1-AS1*	1.52	2.88E-02

Gene ID	Fold Change: AF+HF LA versus NF LA	FDR	Gene ID	Fold Change: AF+HF LA versus NF LA	FDR
NR5A2	0.62	7.69E-04	*PAK1*	0.63	9.07E-03
NRBP2	0.64	1.41E-02	*PAK5*	2.37	2.27E-04
NREP-AS1	2.27	4.86E-02	*PAMR1*	0.51	5.73E-05
NRL	0.65	4.05E-02	*PAQR3*	1.99	1.03E-10
NRP1	0.47	1.66E-06	*PARP9*	0.66	1.54E-02
NRP2	0.57	2.86E-03	*PARVA*	1.58	9.64E-03
NRXN1	0.55	3.97E-03	*PBXIP1*	0.66	2.50E-02
NRXN3	2.97	7.56E-04	*PCDH1*	0.66	1.79E-02
NTF3	0.63	3.35E-03	*PCDH17*	0.56	1.17E-02
NTRK1	0.48	6.77E-03	*PCDH18*	0.48	1.95E-03
NTRK2	2.01	2.94E-02	*PCDH9*	1.94	2.72E-05
NTRK3	0.61	1.90E-04	*PCED1B*	0.61	1.39E-03
NUCB1	0.60	2.47E-05	*PCNA*	0.54	3.16E-04
NUMBL	0.59	2.77E-03	*PCNX2*	0.64	9.27E-03
NWD2	0.56	7.42E-03	*PCNX3*	0.64	4.12E-03
NXN	0.58	3.64E-03	*PDCD4*	1.62	4.78E-02
NXPE1	2.09	2.42E-03	*PDE10A*	0.64	5.03E-03
NXPE4	1.82	3.65E-03	*PDE1A*	0.63	3.93E-03
NYNRIN	0.67	3.45E-02	*PDE3B*	1.50	1.88E-02
OAF	0.30	7.67E-09	*PDE4A*	0.48	9.88E-07
OBSCN	0.60	1.01E-02	*PDE7A*	1.92	1.02E-04
OBSL1	0.60	1.03E-02	*PDE8B*	1.95	7.03E-03
OLFML2B	0.54	9.64E-04	*PDE9A*	0.49	4.25E-06
OPLAH	0.64	4.32E-02	*PDGFD*	2.48	7.15E-03
OPRPN	2.36	1.97E-02	*PDGFRB*	0.33	5.91E-20
OR2B11	2.93	1.49E-02	*PDIA4*	0.58	1.21E-03
OR3A2	0.54	9.69E-05	*PDIA6*	0.65	1.18E-02
OR56A4	2.66	4.83E-02	*PDK4*	6.08	1.13E-04
OR5AH1P	0.63	8.31E-03	*PDLIM7*	0.29	1.96E-09
ORC6	1.94	7.82E-09	*PECR*	0.58	1.01E-04
OSBP2	0.58	6.03E-05	*PFKFB2*	0.66	2.76E-02
OSER1	1.54	1.33E-02	*PFKP*	0.55	6.49E-06
OSGIN2	1.60	5.82E-04	*PGAP1*	1.71	3.66E-05
OSMR-AS1	1.66	2.48E-02	*PGBD2*	1.60	4.80E-02
P2RX3	0.43	3.42E-03	*PGM2L1*	0.52	1.70E-03
P2RX4	0.63	2.38E-03	*PGM3*	1.62	1.07E-02
P2RX6	0.51	5.82E-03	*PGM5*	0.60	9.67E-03
P2RY6	0.45	5.53E-03	*PGPEP1*	0.64	5.12E-03
PACSIN3	0.52	6.91E-06	*PHACTR1*	0.45	2.44E-04

Gene ID	Fold Change: AF+HF LA versus NF LA	FDR
PHACTR3	0.20	1.65E-07
PHF19	0.58	7.28E-04
PHLDB1	0.66	1.02E-02
PHPT1	0.57	1.71E-02
PI16	0.53	9.87E-04
PIAS1	1.70	6.24E-05
PIDD1	0.59	2.12E-02
PIK3AP1	0.54	6.49E-06
PIK3CD	0.61	3.35E-03
PIK3R1	2.08	4.83E-05
PITPNB	2.03	8.08E-07
PITPNM2	0.48	4.70E-07
PITX2	0.37	2.42E-02
PKD1L2	0.50	2.17E-03
PKD2	1.51	4.73E-03
PKDCC	0.65	2.00E-02
PKHD1L1	3.51	1.21E-03
PKN1	0.60	1.35E-04
PLA2G12A	1.75	1.14E-03
PLA2G4E	0.50	3.81E-03
PLA2R1	0.56	2.81E-05
PLAC8L1	1.83	1.62E-04
PLBD2	0.64	1.26E-03
PLCB2	0.38	2.04E-06
PLCD3	0.53	4.74E-03
PLCG2	0.47	2.37E-06
PLCL1	1.66	2.83E-02
PLEK	0.33	1.61E-09
PLEKHF1	1.92	4.43E-03
PLEKHG5	0.46	4.58E-04
PLK2	0.59	4.03E-02
PLPP7	0.63	7.69E-03
PLTP	0.36	1.68E-04
PLXNA4	0.41	4.95E-05
PLXND1	0.47	1.35E-06
PM20D1	2.77	1.68E-04
PMFBP1	0.60	4.25E-03
PMVK	0.63	1.76E-03
PNLDC1	0.58	1.51E-02

Gene ID	Fold Change: AF+HF LA versus NF LA	FDR
PNPLA3	0.43	1.26E-02
PNPLA6	0.56	4.44E-04
POGLUT1	2.74	1.16E-06
POLD1	0.56	1.41E-02
POLDIP2	0.64	1.01E-02
POLR2L	0.65	4.63E-02
POSTN	5.82	1.29E-06
POU6F1	0.63	4.70E-03
PPA1	0.66	8.37E-03
PPARA	1.55	3.09E-03
PPARGC1B	0.38	1.18E-09
PPFIA4	0.61	1.08E-02
PPIP5K2	1.53	3.88E-03
PPL	0.61	3.92E-03
PPP1R12B	0.63	3.38E-03
PPP1R13L	0.49	5.07E-04
PPP1R14C	0.67	1.65E-02
PPP1R1A	0.56	2.13E-02
PPP1R1C	0.58	8.48E-04
PPP1R3A	1.67	5.27E-03
PPP1R3B	0.60	2.37E-02
PPP1R42	0.63	3.81E-02
PPP6R1	0.60	3.09E-04
PQLC2L	0.45	2.10E-05
PRAG1	0.56	4.71E-04
PRDM16	0.60	1.33E-02
PRDM5	1.52	6.14E-03
PRDX1	0.62	4.17E-03
PRDX5	0.62	9.01E-03
PRDX6	1.75	8.90E-05
PRELID2	0.49	2.62E-02
PREX1	0.48	1.30E-08
PREX2	0.60	1.34E-04
PRG4	7.80	5.15E-03
PRICKLE1	2.32	1.50E-03
PRKAG2	0.56	1.79E-02
PRKCA	0.66	1.84E-02
PRKCD	0.58	6.60E-04
PROM1	0.38	2.26E-07

Gene ID	Fold Change: AF+HF LA versus NF LA	FDR
PROS1	2.14	5.53E-04
PROX1	0.61	7.39E-03
PRR12	0.60	1.51E-03
PRR5L	0.63	2.16E-03
PRSS23	0.61	3.46E-04
PRUNE2	0.37	5.17E-06
PSG8	0.66	2.54E-02
PSMC4	0.66	2.28E-03
PSMD6-AS2	1.50	2.03E-02
PTGFR	0.58	9.59E-03
PTGFRN	0.59	9.13E-04
PTGIS	0.51	8.74E-04
PTGS1	1.70	1.41E-02
PTH1R	1.61	4.70E-02
PTP4A2	1.65	3.06E-02
PTP4A3	0.49	1.29E-03
PTPRD	1.67	3.46E-02
PTPRE	0.58	5.75E-04
PTPRO	1.68	2.01E-02
PTPRS	0.61	2.28E-04
PTPRU	0.59	1.73E-02
PYGB	0.51	6.91E-06
PYGM	0.22	7.08E-14
PZP	0.66	3.71E-03
QPRT	0.58	1.06E-02
QSOX1	1.55	2.20E-02
RAB19	0.51	4.24E-02
RAB6C-AS1	0.48	3.48E-02
RAD21	1.56	5.25E-03
RAD23A	0.60	2.75E-04
RAET1E-AS1	0.65	2.97E-02
RAI1	0.65	1.51E-02
RAP1GAP	0.48	7.41E-05
RAPGEFL1	0.58	2.00E-02
RASA1	1.67	6.93E-05
RASA3	0.56	8.61E-05
RASAL2	1.70	2.52E-04
RASD1	3.48	3.34E-05
RASGEF1A	0.50	5.19E-03

Gene ID	Fold Change: AF+HF LA versus NF LA	FDR
RASGRF2	0.54	1.67E-06
RASGRF2-AS1	0.46	4.86E-03
RASL12	0.48	3.84E-03
RASSF3	0.54	7.77E-06
RASSF9	0.57	2.80E-02
RBM11	0.56	4.43E-02
RBM38	0.57	7.69E-04
RBM43	0.40	9.69E-05
RBMS3	1.72	1.61E-03
RBP7	0.52	1.14E-03
RCAN3	0.56	2.01E-04
RCSD1	0.65	3.45E-02
RETSAT	0.55	1.63E-02
RFPL3S	1.83	2.35E-02
RFX2	0.54	4.16E-02
RGL3	0.59	2.08E-02
RGMA	0.45	5.22E-07
RGS2	2.53	7.18E-05
RGS3	0.31	9.01E-09
RGS5	0.62	1.03E-02
RGS6	0.44	1.57E-06
RHBDF1	0.61	7.14E-03
RHOBTB1	1.54	4.17E-03
RHOBTB3	1.64	6.02E-05
RIDA	1.84	5.72E-04
RIMKLA	0.61	9.91E-03
RIOK1	1.70	3.36E-04
RIPK2	1.52	1.27E-02
RNASE1	0.51	1.77E-02
RNF144B	0.60	5.49E-04
RNF152	0.66	7.23E-04
RNF165	0.33	9.60E-11
RNF175	0.47	6.57E-04
RNF187	0.61	2.60E-03
RNF212	0.55	3.45E-02
RNF220	0.58	1.63E-04
RNF5	0.64	2.44E-02
ROBO2	0.41	3.72E-02
ROGDI	0.58	1.02E-02

Gene ID	Fold Change: AF+HF LA versus NF LA	FDR
ROPN1	0.52	3.11E-02
ROR2	1.64	3.59E-02
RPL10P19	0.53	6.32E-03
RPL3L	0.42	3.18E-05
RPS6KA2	1.70	3.27E-03
RPSAP52	1.94	1.49E-02
RRAS	0.62	4.26E-03
RRBP1	1.59	2.99E-02
RSPO1	3.34	4.88E-04
RSPO4	0.47	2.92E-03
RSU1	0.65	1.28E-02
RTN3	0.64	3.57E-03
RTN4RL1	0.56	9.22E-03
RUNDC3B	1.59	2.12E-02
RYR1	0.64	1.64E-02
S1PR3	0.34	8.06E-10
SAA1	0.13	1.83E-02
SAMD4A	0.54	5.20E-05
SAMHD1	1.77	2.82E-03
SBK1	0.22	4.99E-09
SCAF1	0.66	3.63E-03
SCAMP5	0.57	1.20E-03
SCARA3	0.55	4.94E-04
SCARA5	0.34	1.00E-10
SCGB3A2	8.86	5.82E-06
SCNN1G	0.38	6.65E-03
SCTR	0.65	2.65E-02
SEC14L3	2.26	4.04E-02
SEC14L5	0.30	3.29E-05
SELENBP1	0.47	5.85E-03
SELENOM	0.61	1.38E-02
SEMA3C	0.59	2.45E-02
SEMA5A	0.56	4.81E-04
SEMA5B	0.49	3.94E-03
SEPT10	1.53	1.01E-03
SEPT5	0.59	2.74E-03
SEPT9	0.60	1.62E-04
SERPINB2	2.54	8.56E-03
SERPINE1	6.85	4.33E-04

Gene ID	Fold Change: AF+HF LA versus NF LA	FDR
SERPINE3	2.08	9.31E-04
SERPINI2	0.30	4.76E-05
SERTAD4	0.22	9.65E-11
SESN1	1.55	3.66E-03
SETBP1	1.51	5.68E-03
SGF29	0.65	6.12E-03
SGK2	0.32	2.37E-05
SGSH	0.55	1.09E-02
SGSM1	0.50	8.93E-06
SH2D3C	0.58	4.29E-04
SH3BGR	0.53	1.09E-03
SH3D21	0.56	1.06E-02
SH3GL2	0.51	4.70E-03
SH3GL3	0.61	2.60E-02
SH3RF2	0.29	1.09E-03
SHANK3	0.65	1.49E-02
SHC2	0.64	4.69E-02
SHC4	2.40	5.80E-04
SHE	0.65	1.84E-03
SHMT1	0.61	5.57E-03
SHOX2	0.08	2.37E-31
SHQ1	1.76	2.61E-05
SIGLEC1	0.22	1.23E-13
SIPA1L3	0.67	7.88E-03
SIRPB2	0.48	6.63E-03
SIRT2	0.44	1.46E-09
SIX1	0.43	5.83E-03
SKAP2	0.38	1.13E-12
SLC12A7	0.55	4.60E-03
SLC12A9	0.63	1.71E-02
SLC16A9	2.17	1.17E-06
SLC19A2	2.76	9.01E-05
SLC1A2	0.38	5.31E-07
SLC1A7	0.44	1.79E-05
SLC20A2	0.65	1.09E-02
SLC22A1	0.50	6.39E-04
SLC22A3	0.33	1.17E-05
SLC22A5	1.70	2.18E-04
SLC24A5	2.00	3.18E-02

Gene ID	Fold Change: AF+HF LA versus NF LA	FDR
SLC25A18	1.83	6.22E-04
SLC25A23	0.66	1.86E-02
SLC25A32	1.79	4.71E-04
SLC25A33	0.64	2.03E-03
SLC25A40	1.63	2.69E-03
SLC25A48	1.92	1.24E-02
SLC27A1	0.67	2.62E-02
SLC27A6	0.51	9.72E-06
SLC29A1	0.65	4.02E-03
SLC29A4	0.45	1.84E-04
SLC2A4	0.64	4.30E-02
SLC34A2	2.72	8.24E-03
SLC35B1	0.62	6.17E-03
SLC35B3	1.53	3.32E-02
SLC35F1	0.35	6.30E-05
SLC35G1	1.75	7.66E-04
SLC36A2	0.52	1.45E-02
SLC37A1	0.66	1.07E-02
SLC38A2	1.82	1.41E-02
SLC39A8	1.68	5.38E-03
SLC44A5	0.64	2.30E-02
SLC4A3	0.51	1.64E-03
SLC4A7	1.88	1.05E-04
SLC5A4-AS1	0.55	7.42E-03
SLC5A9	0.45	1.83E-04
SLC6A4	1.90	2.09E-03
SLC6A5	0.50	8.51E-04
SLC6A6	2.10	1.98E-02
SLC7A11-AS1	0.57	2.44E-02
SLC7A2	1.78	3.37E-05
SLC7A5	0.52	1.29E-02
SLC7A8	1.89	9.96E-05
SLC8B1	0.58	3.53E-04
SLC9A9	0.62	6.37E-04
SLC9C2	1.55	3.45E-02
SLCO1C1	2.83	2.70E-03
SLCO2B1	0.58	2.42E-03
SLFN11	0.63	1.73E-02
SLFN12	0.61	1.86E-02

Gene ID	Fold Change: AF+HF LA versus NF LA	FDR
SLMAP	0.61	2.77E-02
SLPI	7.84	1.57E-03
SLX4	0.60	6.65E-03
SMAD4	1.74	1.29E-06
SMAD6	2.13	3.94E-02
SMAD7	1.98	3.29E-03
SMAD9	2.72	2.75E-04
SMARCA2	1.55	9.59E-04
SMC6	1.66	3.82E-04
SMCO1	0.46	3.02E-05
SMCO4	1.73	7.59E-03
SMOC2	0.64	2.81E-04
SMTN	0.54	2.44E-04
SMYD1	0.47	8.19E-06
SMYD2	3.71	2.14E-02
SNTB1	0.39	1.88E-15
SNX22	0.29	6.69E-12
SOCS4	1.66	1.01E-04
SOD3	0.39	5.88E-05
SORCS1	0.60	1.87E-03
SORCS2	0.43	1.09E-03
SORL1	0.64	1.75E-02
SOS2	1.51	1.18E-03
SOX9-AS1	1.78	2.62E-02
SPAAR	0.66	2.27E-03
SPAM1	3.49	2.36E-05
SPATA20	0.65	1.42E-02
SPATA22	0.65	1.36E-02
SPATA6L	1.53	8.00E-03
SPEG	0.59	1.07E-02
SPHKAP	0.60	3.08E-03
SPI1	0.53	5.76E-04
SPINK14	2.26	1.86E-02
SPINK5	1.78	1.15E-02
SPINK6	2.08	3.60E-03
SPINK8	0.53	4.12E-02
SPNS2	0.58	4.99E-03
SPON2	0.47	1.43E-03
SPRED1	1.81	1.12E-03

Gene ID	Fold Change: AF+HF LA versus NF LA	FDR
SPRTN	1.52	3.42E-03
SPRY4	2.07	4.16E-05
SPSB4	0.46	3.19E-06
SPTAN1	0.57	2.56E-04
SPTBN4	0.60	1.36E-03
SREBF1	0.36	3.75E-08
SRF	0.65	1.28E-02
SRL	0.64	1.21E-03
SRSF12	1.62	2.05E-02
SSX2IP	1.54	8.11E-03
ST14	0.54	1.28E-02
ST3GAL1	1.58	4.61E-03
ST3GAL2	0.62	1.26E-02
ST3GAL4	1.61	2.52E-02
ST3GAL5	0.49	9.96E-05
ST6GAL2	0.57	8.73E-03
ST6GALNAC5	0.31	1.96E-20
ST8SIA1	0.55	1.12E-04
ST8SIA2	0.50	1.70E-03
STAB1	0.36	3.22E-06
STAT5A	0.60	1.66E-03
STC1	2.62	7.37E-05
STC2	0.51	2.24E-03
STEAP1B	0.66	1.85E-02
STEAP3	0.47	8.98E-06
STK11IP	0.65	2.30E-02
STK38L	0.55	2.16E-03
STK40	0.65	7.54E-04
STOML3	2.60	1.87E-03
STRIP2	0.61	2.53E-02
STUM	0.54	1.60E-03
STX2	1.69	3.46E-03
SULT4A1	0.36	4.24E-03
SYCP2	0.59	1.98E-03
SYDE2	0.53	7.33E-03
SYK	0.49	7.78E-07
SYN2	0.61	1.28E-02
SYNM	0.28	2.42E-08
SYNPO	0.61	2.58E-04

Gene ID	Fold Change: AF+HF LA versus NF LA	FDR
SYPL2	0.51	1.29E-04
SYT10	0.55	4.92E-02
SYT13	0.50	4.81E-04
SYT7	0.44	7.29E-04
SYT9	0.57	1.66E-03
TACO1	0.63	1.26E-02
TAF12	0.63	6.77E-04
TAGLN	0.46	8.21E-03
TAPBPL	0.62	4.75E-03
TAS1R1	0.45	1.49E-02
TASP1	1.77	1.14E-03
TBC1D2B	0.65	3.27E-03
TBC1D8	1.77	6.80E-03
TBCB	0.66	3.59E-02
TBCEL	1.51	1.15E-03
TBPL1	1.95	4.19E-05
TCF24	0.51	4.63E-04
TCN2	0.55	3.71E-04
TDH	0.50	3.46E-02
TDRP	0.63	1.90E-03
TEAD2	0.64	1.65E-02
TEAD4	0.60	1.60E-02
TECR	0.56	7.54E-04
TEK	0.56	3.99E-05
TEKT3	0.63	1.51E-02
TEMN3-AS1	3.99	2.38E-02
TENM3	1.96	1.71E-02
TESC	0.40	2.15E-07
TESMIN	0.63	1.89E-02
TET1	0.61	5.35E-04
TFDP2	1.59	4.64E-03
TGFA	0.47	1.66E-03
TGFB2	0.41	5.29E-10
TGIF1	1.59	4.92E-03
TINAGL1	0.57	2.98E-03
TIPARP	1.78	3.74E-02
TLE2	0.51	1.31E-04
TLL2	0.41	8.90E-05
TLN1	0.63	1.49E-04

Gene ID	Fold Change: AF+HF LA versus NF LA	FDR
TLN2	0.45	7.57E-08
TLR3	0.45	1.92E-03
TLR5	0.57	1.35E-03
TMC3-AS1	0.62	1.17E-02
TMCC2	0.51	3.01E-04
TMED11P	0.49	1.46E-02
TMEM100	4.09	5.52E-14
TMEM117	1.52	4.45E-03
TMEM132B	0.53	1.80E-04
TMEM132C	1.79	1.66E-03
TMEM150C	0.64	4.93E-03
TMEM167A	1.54	5.68E-03
TMEM173	0.63	1.69E-02
TMEM178A	0.38	2.15E-04
TMEM220-AS1	0.48	2.79E-04
TMEM229B	0.55	3.94E-03
TMEM236	2.34	5.45E-03
TMEM25	0.60	1.70E-03
TMEM30A	1.85	1.51E-04
TMEM30CP	0.60	1.62E-02
TMEM38A	0.45	1.84E-04
TMEM38B	1.53	1.01E-03
TMEM39A	1.93	2.51E-04
TMEM39B	0.64	2.23E-02
TMEM43	1.55	4.26E-02
TMEM51	0.55	7.09E-04
TMEM55A	1.56	3.96E-03
TMEM71	2.51	9.47E-04
TMPRSS5	0.51	1.89E-05
TMTC1	2.16	3.08E-06
TMX4	1.57	1.77E-03
TNFAIP3	3.00	2.24E-04
TNFRSF10B	1.52	7.00E-03
TNFRSF10D	1.66	2.21E-02
TNFRSF19	0.35	4.91E-09
TNFRSF1B	0.63	1.97E-02
TNFSF10	0.51	8.90E-05
TNFSF13B	1.53	1.35E-02
TNNT2	0.58	2.46E-03

Gene ID	Fold Change: AF+HF LA versus NF LA	FDR
TNRC18	0.66	1.01E-02
TNS3	0.63	7.92E-03
TNS4	2.14	2.42E-02
TOGARAM2	0.47	1.34E-03
TOX	0.42	2.72E-04
TPCN1	0.59	3.97E-04
TPM1	0.56	2.11E-03
TPM2	0.39	5.91E-08
TPM4	0.56	4.17E-03
TPTE2P1	0.64	4.70E-02
TPTE2P3	1.90	3.89E-02
TRAC	0.08	1.28E-12
TRAF3IP3	0.58	1.39E-04
TRERF1	0.53	1.34E-06
TRG-AS1	0.35	6.77E-08
TRIB1	2.47	2.51E-04
TRIM14	0.63	1.97E-03
TRIM21	0.63	3.33E-02
TRIM38	0.64	2.67E-02
TRIM54	0.64	1.54E-02
TRIM55	0.51	3.38E-03
TRIM59	2.33	1.07E-05
TRIP10	0.59	2.89E-02
TRPC3	1.91	7.74E-03
TRPV1	0.67	1.09E-02
TRPV4	0.48	1.40E-03
TSC2	0.66	8.13E-03
TSC22D4	1.72	3.15E-02
TSHR	0.60	7.38E-03
TSHZ2	0.50	1.78E-06
TSHZ3	0.65	5.26E-03
TSKU	0.33	6.41E-04
TSPAN15	0.38	1.67E-10
TSPAN2	0.57	8.29E-03
TSPAN9	0.54	3.91E-05
TSPEAR	0.28	2.24E-07
TTC28-AS1	1.54	3.00E-03
TTC31	0.65	7.34E-03
TTC32	1.52	1.85E-03

Gene ID	Fold Change: AF+HF LA versus NF LA	FDR
TTLL1	0.57	1.67E-02
TTYH2	0.50	1.41E-04
TTYH3	0.56	3.82E-04
TUBB	0.62	4.96E-03
TUBE1	1.64	5.22E-04
TUFM	0.66	2.97E-02
TUFT1	0.46	8.20E-03
TUG1	1.56	2.75E-03
TWIST2	0.35	1.05E-07
TXNDC16	1.76	8.72E-07
TXNL4B	1.73	9.34E-04
TYK2	0.64	9.73E-03
UBA7	0.59	1.31E-02
UBASH3A	0.51	2.53E-02
UBE2O	0.65	3.00E-03
UBXN6	0.63	1.56E-02
UCP2	0.49	1.60E-04
UGT3A1	1.96	1.86E-02
ULK3	0.63	1.65E-02
UNC13D	0.39	2.08E-03
UNC45B	0.59	1.49E-04
UNC5B	0.44	3.50E-05
UNC5C	0.61	2.48E-04
UNC80	2.96	2.20E-06
UNQ6494	2.02	3.52E-02
UPK1B	3.22	2.65E-05
USO1	1.64	7.98E-05
USP28	0.52	5.60E-05
USP31	1.69	5.39E-04
USP53	2.29	1.90E-08
UTP11	0.56	2.83E-04
UVSSA	0.66	4.46E-02
VAMP8	0.54	2.66E-04
VASH2	0.44	3.20E-03
VAT1L	0.58	3.88E-02
VAV1	0.52	7.41E-05
VCAM1	2.72	7.18E-04
VCL	1.67	3.09E-04
VEGFB	0.58	2.73E-02

Gene ID	Fold Change: AF+HF LA versus NF LA	FDR
VEZF1	1.60	2.56E-03
VIPR2	0.51	8.20E-03
VIT	0.52	4.73E-03
VLDLR-AS1	0.63	5.14E-03
VN2R3P	0.35	4.12E-02
VNN2	0.47	8.68E-03
VSNL1	1.61	2.99E-02
VSTM5	1.88	1.81E-02
VWF	0.66	1.33E-03
WASL	2.03	1.15E-06
WDFY3-AS2	0.66	2.45E-02
WDR41	1.69	6.03E-05
WDR49	0.45	1.01E-03
WDR62	0.54	6.66E-04
WDR66	0.53	2.36E-03
WEE2-AS1	0.53	3.82E-03
WFDC1	0.39	5.97E-05
WFS1	0.61	5.67E-03
WISP1	0.58	2.11E-02
WISP3	1.67	2.30E-02
WNK2	0.58	1.34E-03
WSCD2	0.48	5.14E-03
WT1	1.71	3.10E-02
XIRP2	0.45	2.72E-04
XKR4	0.55	5.35E-05
XPO1	1.54	1.66E-03
XPR1	1.64	1.01E-02
YARS	0.63	2.18E-04
YBX3	1.90	3.38E-03
YIPF7	1.77	2.85E-03
Z84485.1	0.66	9.97E-03
Z95331.1	0.60	5.89E-03
ZBTB16	2.13	3.29E-05
ZBTB7C	0.41	9.98E-09
ZDHHC21	1.81	3.88E-06
ZFAND5	1.92	1.66E-02
ZFP36	2.67	9.27E-03
ZHX1-C8orf76	1.55	1.37E-03
ZMYND12	0.65	2.95E-02

Gene ID	Fold Change: AF+HF LA versus NF LA	FDR
ZNF180	0.64	8.50E-04
ZNF189	3.35	1.63E-13
ZNF259P1	1.57	4.12E-02
ZNF280B	0.63	3.82E-03
ZNF300P1	1.97	2.73E-06
ZNF330	1.78	3.69E-03
ZNF365	0.53	5.88E-03
ZNF366	0.53	2.60E-06
ZNF385B	2.95	3.03E-07
ZNF423	0.47	9.33E-07
ZNF451	1.75	6.55E-06
ZNF622	2.37	1.09E-06

Gene ID	Fold Change: AF+HF LA versus NF LA	FDR
ZNF624	0.45	7.84E-06
ZNF710	0.62	1.37E-03
ZNF732	1.58	6.37E-03
ZNF774	0.59	2.58E-03
ZNF800	1.50	5.29E-03
ZNF815P	0.45	7.28E-05
ZNF90	0.55	5.99E-03
ZNF93	0.62	1.57E-03
ZNHIT3	0.65	2.41E-02
ZPBP	0.54	1.92E-02
ZRANB2	1.69	4.02E-03
ZRANB2-AS1	1.69	2.92E-03

Cardiomyocyte nuclei (CMN) were sorted from the left atria (LA) of non-failing human hearts (NF, $n = 5$) and failing hearts with a history of atrial fibrillation (AF+HF, $n = 5$). RNA was isolated and RNA-sequencing was performed from these samples. Genes with a linear fold change ≤ 0.667 and ≥ 1.5 with a false discovery rate (FDR) < 0.05 were considered significant and are included in the table.

Table S6. Differentially expressed RNA-sequencing transcripts from humans with heart failure RA cardiomyocyte nuclei compared to non-failing RA cardiomyocyte nuclei

Gene ID	Fold Change: HF RA versus NF RA	FDR
ABCA9	0.58	3.41E-02
ABCB4	0.58	1.21E-02
ABHD17C	0.26	3.60E-02
AC005162.3	0.17	2.73E-04
AC005332.1	2.21	1.24E-03
AC005351.1	0.17	1.18E-05
AC005865.2	0.31	4.34E-02
AC006023.2	3.50	3.72E-03
AC008464.1	0.39	2.02E-02
AC008691.1	0.32	2.03E-03
AC008700.1	0.41	3.56E-03
AC008705.1	0.25	2.18E-02
AC008991.1	0.21	3.86E-03
AC009951.1	0.55	2.18E-02
AC011700.1	0.60	4.75E-02
AC016831.5	1.91	1.02E-02
AC018529.3	0.32	1.21E-02
AC018630.2	2.01	1.31E-02
AC021087.4	0.39	4.62E-02
AC022706.1	0.09	2.26E-02
AC024940.1	0.06	4.66E-03
AC025271.4	0.10	7.06E-03
AC039056.2	0.18	2.61E-02
AC058791.1	1.68	4.34E-02
AC067750.1	2.40	1.24E-03
AC068587.1	0.19	4.77E-02
AC068700.1	0.51	3.75E-02
AC073389.2	0.14	2.68E-03
AC083902.2	3.22	1.54E-05
AC087203.1	0.26	2.67E-02
AC090213.1	5.60	1.05E-02
AC090616.6	3.07	4.63E-02
AC091230.1	0.29	1.54E-05
AC092349.1	0.49	4.64E-02
AC093607.1	4.12	2.00E-02
AC093752.2	2.29	2.33E-02
AC093908.1	1.77	5.55E-03
AC097658.1	2.30	3.35E-02
AC097662.1	2.20	3.55E-02
AC098679.1	0.41	4.66E-03
AC098679.3	0.33	9.11E-03
AC100800.1	1.75	3.37E-02
AC103591.3	0.13	1.74E-02
AC107398.3	0.26	1.25E-02
AC107983.2	4.79	4.19E-03
AC112203.1	0.08	3.14E-02
AC112777.1	2.27	4.86E-02
AC114488.2	0.16	4.01E-02
AC117529.1	0.36	3.37E-02
AC131902.3	0.06	3.71E-02
AC239802.2	0.57	4.58E-02
ACSL6	0.32	1.28E-02
ACTA1	3.40	7.64E-04
ADAMTS3	0.46	3.94E-03
ADAMTS7	0.45	1.15E-03
ADAP2	0.40	1.17E-02
ADGRF4	3.37	3.31E-02
ADORA3	0.30	4.94E-02
AL009031.1	0.33	1.21E-02
AL021068.2	2.04	4.65E-02
AL031666.2	2.43	8.59E-03
AL033519.1	5.04	1.04E-08
AL078590.3	2.10	2.59E-03
AL136164.3	1.68	4.86E-02
AL136317.2	0.06	4.00E-03
AL139022.1	1.85	4.64E-02
AL139807.1	2.75	4.62E-02
AL162727.2	0.37	4.20E-02
AL355864.1	0.30	2.99E-02
AL391825.1	2.08	4.87E-02
AL591178.1	2.96	4.63E-02
AL596202.1	0.46	1.31E-02
ALDOB	3.09	7.27E-04
ALG9	1.85	3.35E-02

Gene ID	Fold Change: HF RA versus NF RA	FDR
ANKRA2	1.71	2.18E-02
ANLN	0.39	4.88E-02
AP000897.1	1.91	5.40E-03
AP000911.1	0.42	4.82E-02
AP001781.2	3.58	4.99E-03
AP006222.1	0.33	9.71E-03
AP1G2	0.38	3.08E-02
APOLD1	2.53	1.54E-05
AQP7	0.49	3.94E-03
AREG	3.82	5.69E-05
ARHGEF2	0.62	2.27E-02
ARHGEF26	2.24	8.59E-03
ARID4A	0.63	4.75E-02
ARRB2	0.50	1.64E-02
ASPN	2.91	1.76E-05
ATAT1	1.61	3.28E-02
ATP8B3	0.20	4.65E-02
BBS12	1.87	2.67E-02
BEST3	0.25	6.59E-03
BHLHE41	0.55	2.40E-02
BLOC1S1	0.62	4.50E-02
BMPR1APS1	1.84	2.20E-03
BOK	2.83	2.37E-02
BTG2	2.63	4.25E-03
BUB1B	0.17	7.27E-03
C10orf10	1.99	5.79E-03
C10orf128	0.47	4.73E-02
C10orf71	0.62	2.17E-02
C3orf58	3.63	2.44E-05
C5orf51	2.01	8.25E-04
CA11	0.30	4.86E-02
CAMKK1	0.39	2.90E-02
CASC19	0.27	1.30E-02
CASKIN1	0.35	4.34E-02
CBWD6	0.28	3.19E-02
CCBE1	3.51	6.71E-03
CD74	2.23	4.66E-03
CEBPD	1.91	3.90E-03
CES3	0.31	3.92E-02
CFAP61	4.43	1.18E-03

Gene ID	Fold Change: HF RA versus NF RA	FDR
CFD	2.01	9.81E-03
CHD3	0.58	1.20E-02
CHRNA7	2.28	4.63E-02
CHST3	1.77	1.76E-02
CLEC2D	0.51	4.65E-02
CLTCL1	1.63	2.67E-02
CNN2P3	0.02	8.23E-05
COLEC12	1.93	2.98E-02
CORO6	0.58	7.46E-03
CPEB4	1.77	3.27E-03
CR392039.4	2.63	1.21E-02
CRIM1	1.61	4.89E-02
CSPG4	1.68	2.92E-02
CXXC5	0.54	4.63E-02
CYP2E1	0.31	6.34E-04
CYTH4	0.30	3.28E-02
DAAM1	0.65	4.01E-02
DDX18P3	0.36	2.34E-02
DIMT1P1	3.56	2.73E-04
DOCK5	2.14	1.18E-05
DTX4	0.35	2.72E-02
E2F2	0.18	3.15E-02
EBLN2	1.62	4.48E-02
EDNRA	1.66	2.11E-02
EGFR	2.22	1.06E-06
EGLN3	1.74	4.14E-02
ELFN1	0.41	3.99E-02
ELL2	2.19	2.81E-04
EMCN	0.49	2.59E-03
EMP1	1.87	4.34E-02
ENO3	0.55	2.13E-02
EPHA5	4.00	3.85E-03
ERRFI1	1.95	1.74E-02
ETV6	0.60	2.91E-02
EYA1	0.59	1.39E-02
EYA4	1.75	3.74E-02
FAM124A	0.47	2.11E-02
FAM126B	2.28	2.73E-04
FAM180A	0.40	4.34E-02
FAM185BP	2.47	4.18E-02

Gene ID	Fold Change: HF RA versus NF RA	FDR	Gene ID	Fold Change: HF RA versus NF RA	FDR
FAM212B	2.00	2.83E-03	*HLA-DQA1*	2.77	7.62E-04
FAM46C	0.58	4.77E-02	*HLA-DRA*	2.30	6.83E-04
FARP2	1.77	3.99E-02	*HLA-DRB1*	2.17	1.74E-02
FBXL22	0.55	4.34E-02	*HOPX*	2.47	5.14E-03
FBXO40	0.60	3.35E-02	*HPR*	6.73	9.81E-03
FCN1	0.20	4.94E-02	*HRH2*	0.29	2.57E-08
FGF7	0.54	1.17E-02	*HSPB1*	1.56	3.29E-02
FIBIN	2.76	3.90E-03	*HTR5BP*	2.35	1.74E-02
FKBP5	3.24	9.25E-04	*IFFO1*	0.49	3.27E-03
FMNL1	0.46	3.49E-02	*IL10RA*	0.47	9.77E-03
FNDC3B	1.62	1.45E-02	*IL32*	2.53	3.38E-02
FOSL2	1.78	2.52E-03	*IL6R*	0.55	1.96E-02
FOXO3	1.87	1.25E-03	*INHBA*	0.32	1.02E-02
FP671120.4	1.59	3.08E-02	*IQCD*	0.22	3.38E-02
FSD2	0.49	5.55E-03	*IQGAP3*	0.28	2.31E-02
G0S2	0.36	4.45E-02	*IRF4*	0.26	4.66E-03
GABRB1	0.34	4.10E-03	*IRS2*	1.77	1.02E-02
GADD45G	2.34	5.13E-03	*ITGA2B*	0.35	4.34E-02
GALNT16	1.80	2.29E-02	*ITGAM*	0.55	3.35E-02
GALNT5	0.29	4.53E-02	*JAK2*	2.00	8.26E-04
GALNT8	0.47	3.92E-02	*KAZN*	0.45	1.39E-02
GDA	0.13	4.73E-02	*KCNH7*	0.21	3.31E-02
GEN1	1.72	3.13E-02	*KCNIP2*	0.52	3.31E-03
GINS3	12.47	1.17E-04	*KLF15*	2.60	8.80E-07
GOLGA8S	0.32	6.32E-03	*KLHL36*	2.67	3.25E-06
GPR153	2.04	3.90E-03	*KLHL41*	0.55	1.74E-02
GRXCR2	0.14	8.72E-08	*KSR1*	0.58	4.60E-02
GTSE1	0.30	4.94E-02	*LBR*	1.72	3.38E-02
GXYLT1P6	0.06	2.73E-04	*LCNL1*	0.43	3.24E-02
HBB	35.66	2.18E-05	*LILRA2*	0.21	4.36E-02
HCN1	0.49	6.32E-03	*LIMS1*	0.59	5.51E-03
HEG1	1.74	4.50E-03	*LINC00513*	1.82	1.63E-02
HES6	0.21	2.28E-02	*LINC00881*	1.96	1.34E-02
HFE	2.03	3.07E-02	*LINC01237*	0.26	4.86E-02
HIST1H1D	0.15	5.04E-04	*LINC-PINT*	1.62	4.40E-02
HIST1H1E	0.40	2.18E-04	*LMAN1L*	0.34	1.17E-04
HIST1H2AL	0.18	1.22E-03	*LMOD1*	0.46	3.33E-02
HIST1H2BH	0.27	5.69E-05	*LPCAT4*	0.58	3.92E-02
HIST4H4	0.46	1.26E-02	*LPL*	2.53	2.01E-04
HIVEP2	1.51	3.92E-02	*LRRC10*	0.30	4.57E-03

Gene ID	Fold Change: HF RA versus NF RA	FDR	Gene ID	Fold Change: HF RA versus NF RA	FDR
LRRC39	0.52	1.46E-02	*NPR3*	0.59	4.11E-02
LRRC75A	2.00	4.71E-02	*NSUN7*	0.58	2.99E-02
LRRC8A	1.59	3.63E-02	*NTRK2*	2.68	8.58E-03
LST1	0.12	2.84E-04	*NUDT4*	1.64	3.20E-02
LTBP4	1.87	2.97E-02	*OBSL1*	0.60	2.11E-02
MAF	0.54	2.91E-02	*OGFRL1*	1.62	3.50E-02
MAP1A	0.59	1.21E-02	*OGN*	3.43	2.83E-04
MAST4	1.60	1.17E-02	*OPRL1*	0.19	3.10E-02
MBD4	1.61	1.90E-02	*OR7E2P*	0.19	2.14E-03
MCHR1	0.14	1.11E-02	*P2RX6*	0.44	4.23E-02
MECOM	0.50	8.25E-04	*PAK1*	0.61	2.51E-02
MESTP1	1.63	7.82E-03	*PCLO*	0.54	1.92E-03
MFAP4	2.04	4.34E-02	*PCSK6*	2.07	1.70E-04
MIR4451	1.86	3.14E-02	*PDE3B*	1.54	3.74E-02
MKI67	0.10	1.04E-08	*PDK4*	5.39	1.04E-08
MKNK2	2.65	1.25E-06	*PDLIM1*	1.53	3.99E-02
MORF4L1P3	1.81	4.99E-02	*PDSS1P1*	2.38	2.67E-02
MPP7	0.59	3.08E-02	*PER1*	1.63	1.58E-02
MSR1	0.43	1.37E-02	*PGR-AS1*	0.51	2.91E-02
MT2A	2.43	2.77E-03	*PIK3R1*	1.68	3.14E-02
MTCO1P11	2.11	7.26E-03	*PIM1*	1.73	2.61E-03
MTCO2P11	2.52	2.74E-02	*PITPNM2*	0.57	1.87E-02
MTF2	1.57	2.37E-02	*PKIA*	0.45	6.11E-03
MTND1P11	2.00	1.24E-02	*PLA2G4F*	0.21	3.00E-02
MTND4P14	2.10	4.11E-02	*PLEKHF1*	2.64	2.72E-04
MTND5P14	2.08	4.29E-02	*PLEKHH3*	0.46	2.61E-02
MUC20P1	0.13	4.47E-02	*PLOD2*	0.50	2.79E-03
MYC	2.08	2.24E-02	*PM20D1*	0.29	4.14E-05
MYH7B	0.50	1.43E-02	*POGLUT1*	2.19	2.79E-04
MYO1F	0.46	3.69E-03	*POLQ*	0.32	2.70E-04
MYO5C	0.51	3.02E-03	*POTEJ*	6.45	3.14E-02
MYOC	4.67	1.70E-03	*POU5F1B*	0.22	2.86E-02
NCKAP1L	0.54	3.14E-02	*PPARGC1B*	0.53	4.66E-03
NEB	0.40	7.47E-03	*PPIAP16*	1.88	2.10E-02
NFATC4	0.39	1.01E-02	*PRELID2*	0.35	1.54E-05
NFKBIA	1.83	1.69E-02	*PRKG2*	0.37	3.75E-02
NIN	0.43	2.18E-04	*PRNCR1*	0.28	2.97E-02
NKAIN2	2.29	1.66E-02	*PROX1*	0.53	3.58E-03
NPM1P11	0.20	9.56E-03	*PSD3*	1.63	3.20E-02
NPM1P30	0.27	1.54E-02	*PTN*	2.33	2.67E-03

Gene ID	Fold Change: HF RA versus NF RA	FDR
PTPN14	1.68	1.49E-02
PTPRO	0.22	7.22E-03
PTX3	4.29	4.28E-03
PXDC1	1.78	1.29E-02
PYGM	0.56	2.51E-02
RAB8B	1.85	2.91E-02
RASD1	3.73	4.21E-03
RGCC	3.13	1.80E-03
RGS3	0.46	4.28E-03
RIOX1	1.85	1.15E-02
RN7SKP25	3.29	4.34E-02
RN7SL336P	3.08	3.16E-02
RNASE2	0.09	4.56E-03
RNF165	0.36	1.21E-02
RNU4ATAC16P	0.05	1.80E-03
RNU6-653P	2.22	4.75E-02
ROR1	1.86	2.10E-02
RPSAP48	0.20	1.61E-02
RRAD	0.35	4.25E-04
RUNX1T1	0.56	2.18E-02
S100A8	0.18	5.36E-04
S100A9	0.20	1.17E-02
S1PR1	1.64	8.91E-03
SAMD4A	0.61	4.18E-02
SDCBP	1.72	3.48E-02
SEMA6C	0.55	1.65E-02
SEMA6D	0.50	1.51E-03
SEPHS1P1	3.00	4.24E-02
SERPINE1	3.44	9.64E-04
SERTAD4	0.19	1.17E-04
SGSM1	0.49	2.24E-03
SH3RF1	1.60	4.73E-02
SIPA1L2	0.61	2.67E-02
SIRPB2	0.32	2.74E-02
SLC19A1	0.43	4.57E-03
SLC19A2	2.29	4.83E-04
SLC1A3	1.82	4.27E-02
SLC25A18	2.48	2.52E-03
SLC26A2	1.96	9.89E-03
SLC38A2	1.92	2.24E-03

Gene ID	Fold Change: HF RA versus NF RA	FDR
SLC44A5	0.11	4.89E-03
SLC4A7	1.95	1.22E-02
SLC7A8	2.64	2.73E-04
SLFN5	0.56	1.26E-02
SMOC2	2.38	4.50E-03
SMYD1	0.52	6.31E-03
SMYD2	2.11	3.55E-02
SNORA5C	2.29	4.69E-02
SPRY1	1.85	2.57E-03
SPTLC3	0.50	4.66E-03
SREBF1	0.46	2.16E-02
SRSF12	2.39	4.82E-02
ST3GAL5	0.39	1.43E-04
ST8SIA4	0.19	1.22E-02
STAB1	0.52	3.99E-02
STARD4-AS1	0.53	9.56E-03
STC2	0.46	2.66E-02
STK17B	2.58	2.24E-03
STX3	0.61	1.90E-02
SULT1A1	0.56	3.99E-02
SYK	0.39	1.63E-02
SYNE3	1.62	3.72E-02
SYT13	0.18	7.35E-04
TAS2R43	4.20	1.05E-05
TBPL1	2.79	2.18E-05
TBX18	0.42	5.13E-03
TBX5	0.58	1.75E-02
TET1	0.51	7.48E-03
TLL1	0.36	2.92E-02
TMC8	0.41	3.99E-02
TMEM100	2.20	1.78E-02
TMEM131L	0.60	3.22E-02
TMEM144	0.42	9.25E-04
TMTC1	1.60	4.73E-02
TOB2	1.84	7.74E-03
TOP2A	0.18	4.82E-03
TRA2A	1.59	4.01E-02
TRIM2	0.63	4.29E-02
TRIM45	0.65	3.25E-02
TSACC	3.16	1.21E-02

Gene ID	Fold Change: HF RA versus NF RA	FDR
TSPYL1	1.59	3.34E-02
TUBA3E	4.25	2.84E-04
TWF1	1.96	1.44E-02
TXNL4B	2.79	7.67E-04
UBE2QL1	0.47	2.20E-03
VCL	1.60	2.65E-02
VMP1	1.82	4.62E-02
VTCN1	0.40	1.94E-04
WDR17	0.18	8.59E-03
WFDC3	0.15	3.35E-02
WISP2	0.52	1.96E-02
XIRP1	1.95	4.66E-03
XRRA1	0.42	9.25E-04
ZBTB16	2.30	2.84E-04
ZNF189	2.44	1.92E-04
ZNF330	1.78	1.36E-02
ZNF589	0.50	4.83E-04
ZNF677	1.61	2.31E-02

Cardiomyocyte nuclei (CMN) were sorted from the right atria (RA) of non-failing human hearts (NF, $n = 6$) and failing hearts (HF, $n = 3$). RNA was isolated and RNA-sequencing was performed from these samples. Genes with a linear fold change ≤ 0.667 and ≥ 1.5 with a false discovery rate (FDR) < 0.05 were considered significant and are included in the table.

Table S7. Differentially expressed RNA-sequencing transcripts from humans with heart failure LA cardiomyocyte nuclei compared to non-failing LA cardiomyocyte nuclei

Gene ID	Fold Change: HF LA versus NF LA	FDR
ABCA1	0.25	2.69E-05
ABCB4	0.56	2.03E-02
ABCD3	1.79	5.02E-04
ABCG1	0.37	1.75E-03
ABCG2	2.88	3.78E-02
AC000374.1	0.05	3.26E-03
AC002480.2	0.18	2.48E-02
AC004466.3	2.58	7.51E-03
AC004594.1	0.28	6.21E-03
AC005180.1	0.17	1.20E-02
AC005180.2	0.18	2.54E-03
AC005351.1	0.19	5.22E-03
AC005358.1	0.31	1.61E-02
AC005358.2	0.36	1.12E-03
AC005550.3	0.16	1.04E-02
AC005865.2	0.45	2.01E-02
AC007216.3	2.19	2.34E-02
AC007342.7	3.23	5.69E-05
AC007996.1	3.06	3.91E-05
AC009084.2	2.37	4.31E-02
AC009093.1	0.12	5.69E-05
AC009411.2	0.03	3.65E-03
AC009955.2	0.27	4.57E-02
AC010997.6	0.37	3.59E-03
AC011284.1	0.24	2.45E-02
AC011752.1	0.05	3.75E-03
AC015813.7	3.39	1.87E-04
AC018742.1	0.07	9.37E-05
AC018797.3	2.84	1.14E-02
AC024940.1	0.05	2.94E-03
AC025741.1	3.02	4.07E-02
AC027117.1	2.17	9.14E-03
AC027449.1	2.23	1.38E-02
AC036108.2	0.18	1.20E-03
AC036108.3	0.33	1.17E-02
AC063952.1	2.27	2.08E-03
AC064799.1	0.38	1.44E-02
AC069360.1	0.25	2.14E-02
AC078846.1	1.76	2.00E-02
AC078950.1	0.37	4.95E-02
AC083843.3	1.90	4.06E-02
AC087457.1	0.15	1.79E-02
AC090193.1	0.10	2.86E-02
AC090229.1	3.06	4.01E-02
AC090236.2	2.04	3.61E-02
AC091182.1	0.21	4.59E-03
AC091230.1	0.32	7.15E-03
AC091393.1	2.31	3.90E-02
AC091825.1	2.22	3.97E-02
AC092053.3	2.04	3.49E-02
AC092171.4	3.15	3.61E-03
AC092687.1	0.55	2.00E-02
AC093908.1	1.62	4.70E-02
AC100788.1	0.31	4.07E-02
AC104371.1	2.65	4.77E-02
AC104564.6	0.42	4.60E-02
AC104695.2	3.36	9.28E-04
AC104958.1	0.18	4.82E-02
AC106801.1	0.23	3.54E-04
AC107068.1	0.21	3.60E-04
AC107208.1	4.83	8.57E-03
AC107980.1	0.07	2.54E-03
AC107983.2	5.82	2.82E-03
AC108673.3	2.92	6.19E-03
AC109779.1	0.26	1.63E-02
AC109826.1	0.06	2.16E-02
AC138904.1	0.22	1.04E-02
AC138904.3	0.32	4.88E-02
AC243562.2	4.09	2.06E-04
ACSL6	0.25	1.46E-02
ACSM3	0.42	4.16E-02
ACSM5	0.05	9.38E-04
ACSS2	0.63	4.99E-02
ACTA2	0.43	4.22E-02

Gene ID	Fold Change: HF LA versus NF LA	FDR
ACTA2-AS1	0.24	2.23E-02
ACTG1P18	0.23	7.92E-03
ACTG2	0.31	2.37E-03
ADAM22	1.91	2.00E-02
ADAMTS1	0.41	9.12E-03
ADAMTS12	0.36	4.67E-02
ADAMTS17	0.45	2.47E-02
ADAMTSL4	0.48	3.99E-03
ADAP2	0.30	1.83E-02
ADGRG6	2.07	4.87E-02
ADIPOQ	0.16	4.88E-02
ADM	3.27	1.87E-02
ADRA1A	0.14	1.03E-04
ADRA2A	0.20	1.66E-02
AGAP2	0.24	1.33E-02
AIF1	0.42	3.63E-02
AIF1L	0.42	1.48E-03
AK5	3.05	6.30E-03
AKTIP	2.02	3.00E-03
AL008628.1	3.79	3.97E-02
AL021068.2	2.52	4.16E-02
AL022323.4	0.41	2.43E-02
AL033519.1	4.32	1.73E-03
AL078590.3	2.96	4.47E-02
AL121772.3	0.45	3.85E-02
AL132642.1	0.16	8.59E-05
AL136164.3	1.94	5.64E-03
AL136164.4	2.00	3.73E-02
AL138688.2	3.03	3.96E-02
AL138828.1	2.25	3.26E-03
AL139805.1	0.20	1.67E-02
AL157714.2	0.29	9.76E-03
AL159166.1	0.27	1.62E-03
AL160281.1	2.11	6.58E-04
AL162632.1	0.21	4.66E-03
AL162632.3	0.18	3.26E-03
AL353729.2	0.24	1.46E-02
AL354760.1	2.58	1.04E-02
AL450384.2	2.09	4.84E-02
AL451069.1	0.35	6.50E-04

Gene ID	Fold Change: HF LA versus NF LA	FDR
AL512649.2	3.33	3.00E-02
AL589666.2	3.09	5.22E-03
AL645568.1	1.86	1.71E-02
ALDOB	4.11	8.55E-08
AMOTL1	1.81	1.60E-03
ANGPT1	5.24	2.29E-03
AP000560.1	0.26	4.66E-03
AP001172.1	0.32	4.67E-02
AP002884.3	0.40	6.21E-03
AP003170.1	0.43	3.85E-03
AP003469.4	1.70	1.90E-02
APBB1IP	0.29	5.22E-03
APOD	0.16	3.44E-07
AQP7P2	0.09	2.06E-04
AREG	3.29	4.23E-06
ARHGAP17	1.58	4.82E-02
ARHGAP9	0.36	8.43E-03
ARHGEF4	0.24	2.08E-03
ASB10	0.34	3.06E-04
ASB2	0.38	1.05E-03
ATAD2	2.02	6.81E-04
ATP1A1	1.61	2.89E-02
B4GALT1	1.97	3.40E-03
BBS12	2.71	2.37E-04
BEST3	0.31	2.14E-02
BHLHE41	0.54	4.33E-02
BIN1	0.38	3.78E-02
BLNK	0.19	4.23E-02
BMP10	163.24	1.51E-05
BMP4	2.47	2.67E-02
BNC2	0.47	1.41E-02
BTG2	2.22	1.24E-07
BTNL9	0.36	5.22E-03
C15orf52	0.60	2.09E-02
C15orf61	4.25	1.12E-03
C1GALT1P1	1.89	4.95E-02
C1orf186	0.08	4.99E-02
C1QB	0.46	4.82E-02
C3orf52	4.16	5.42E-04
C3orf58	1.95	1.92E-02

Gene ID	Fold Change: HF LA versus NF LA	FDR
CACNA1E	0.06	5.42E-04
CACNA1H	0.32	1.83E-02
CADPS2	0.25	4.62E-04
CALB1	2.25	3.12E-02
CARMIL1	2.34	2.97E-02
CAV1	0.58	3.12E-02
CBX4	0.45	6.21E-03
CCNH	1.55	3.41E-02
CCT2	0.56	4.86E-02
CD14	0.35	3.36E-02
CD209	0.25	1.11E-03
CD274	1.82	2.74E-02
CD9	2.51	2.95E-06
CDCA2	0.24	1.97E-02
CDH22	0.25	4.98E-03
CDKN1A	2.14	5.69E-05
CDKN1C	0.41	1.65E-03
CEACAM19	0.36	4.63E-03
CEBPD	1.80	1.73E-02
CENPF	0.26	3.68E-02
CERK	1.61	2.08E-02
CETN4P	2.22	3.22E-03
CHST3	2.25	4.59E-03
CIDEA	0.28	4.66E-03
CISH	2.25	3.41E-02
CLEC10A	0.31	4.19E-02
CLUHP3	1.58	4.02E-02
CMSS1	0.52	3.30E-02
CNN2	0.56	1.79E-02
CNOT6L	1.59	4.69E-02
CNTNAP3	0.41	4.12E-02
COL27A1	0.27	6.56E-05
COL9A1	8.43	7.47E-05
COLQ	0.38	8.09E-04
COMTD1	0.45	2.00E-02
CPAMD8	0.33	5.32E-03
CPNE5	1.90	1.85E-03
CPSF4	1.75	2.68E-02
CR1	0.21	7.90E-03
CSPG4P12	8.09	4.23E-04

Gene ID	Fold Change: HF LA versus NF LA	FDR
CSRNP1	1.80	3.57E-02
CSRP3	0.51	4.95E-02
CXCR4	2.74	3.73E-02
CXCR6	0.45	4.59E-03
CYTH4	0.27	1.36E-02
DAB2	0.35	3.66E-03
DCUN1D2-AS	2.39	1.26E-02
DERL3	3.72	8.81E-05
DGAT2	0.16	1.38E-02
DHRS9	0.32	2.48E-03
DHX15	1.73	7.14E-03
DNAH14	0.46	3.19E-02
DNER	4.10	7.15E-04
DNM1	0.45	3.77E-02
DNM1P51	3.88	1.32E-02
DNMT1	0.59	1.56E-02
DOK3	0.21	3.26E-03
DPYSL4	0.30	2.20E-03
DUSP1	2.01	3.65E-03
DUSP10	0.38	2.48E-03
EBF2	0.43	1.87E-04
EBLN2	1.71	2.48E-02
EDNRA	1.89	1.46E-02
EED	1.62	4.07E-02
EGR1	3.27	1.73E-02
EGR2	4.07	4.25E-04
EHBP1	0.51	9.15E-03
EHD2	0.54	2.37E-02
ELL2	2.04	4.66E-03
ELN	0.44	7.49E-03
EMP1	2.03	6.55E-03
EPB41	0.58	1.93E-02
EPHA7	1.74	3.65E-02
EPM2AIP1	1.74	1.36E-02
EPS8	0.59	3.90E-02
ERC2	0.48	1.73E-02
ERRFI1	1.95	3.20E-02
ETV1	0.62	4.01E-02
ETV6	0.49	1.37E-02
EXOC8	1.85	5.85E-03

Gene ID	Fold Change: HF LA versus NF LA	FDR	Gene ID	Fold Change: HF LA versus NF LA	FDR
EZR	1.53	4.88E-02	*GRIA1*	0.20	3.02E-02
F13A1	0.22	1.87E-04	*GRIP2*	0.57	4.88E-02
F2R	2.09	7.14E-03	*GRXCR2*	0.09	5.69E-05
FABP4	0.17	1.74E-03	*GSTO2*	2.81	2.54E-04
FABP5	0.39	4.06E-02	*GUCY1A2*	0.36	5.35E-03
FAM106A	3.33	8.20E-03	*GUCY2C*	0.53	4.90E-02
FAM111B	0.10	2.01E-02	*HAMP*	16.63	2.26E-03
FAM126B	1.83	1.03E-02	*HBB*	18.74	5.69E-05
FAM131A	2.22	2.67E-03	*HFE2*	0.60	3.22E-02
FAM184A	0.32	2.45E-03	*HIST1H1C*	0.45	9.99E-04
FAM212B	2.62	1.16E-03	*HIST1H1E*	0.43	1.30E-02
FAM89A	0.30	3.24E-02	*HIST1H2BD*	0.49	3.91E-02
FARP2	1.72	2.73E-02	*HIST1H2BH*	0.22	2.10E-02
FASN	0.11	3.89E-04	*HK2*	0.37	4.43E-02
FCGBP	0.25	1.74E-02	*HLA-DRB1*	2.19	7.66E-03
FCN1	0.15	1.75E-03	*HLX*	0.40	4.69E-02
FGF12-AS3	0.33	5.43E-04	*HNRNPLP2*	2.43	2.55E-02
FGF7	0.46	7.51E-03	*HOMER1*	0.57	2.43E-02
FGFR3	2.77	4.73E-02	*HOXA2*	0.01	1.03E-04
FIBIN	3.15	3.07E-03	*HOXA3*	0.11	7.36E-04
FIGN	0.66	4.95E-02	*HOXA-AS2*	0.17	3.60E-02
FNBP4	0.63	2.48E-02	*HOXB3*	0.16	2.17E-03
FOSB	9.34	9.37E-05	*HSPA12B*	0.39	1.56E-02
FOSL2	1.97	5.34E-04	*HTR4*	0.23	1.50E-05
FOXC1	2.01	4.91E-02	*HTR5BP*	2.58	2.14E-02
FOXO3	1.79	7.14E-03	*IFFO1*	0.59	4.88E-02
FP325317.1	0.17	4.70E-02	*IGF1*	0.26	6.96E-05
FSD2	0.51	2.84E-02	*IGFBP3*	2.39	1.24E-02
FSTL1	2.00	5.69E-04	*IL10RA*	0.50	4.16E-02
FYB2	0.42	2.37E-02	*IL2RA*	0.21	5.22E-03
G0S2	0.21	9.27E-03	*INMT*	2.09	2.58E-03
GATA5	0.23	1.60E-03	*ITIH5*	0.46	8.20E-03
GCSAM	3.47	2.78E-02	*ITPKB*	0.49	2.37E-02
GIMAP4	0.54	1.90E-02	*ITPRIP*	1.91	1.12E-03
GJB7	2.58	5.84E-04	*JAKMIP3*	0.36	2.03E-02
GNPDA2	2.06	1.19E-02	*JUNB*	2.27	2.82E-03
GPD1	0.22	9.43E-03	*KCNA4*	4.08	4.59E-02
GPER1	0.40	3.22E-02	*KCNA7*	0.13	2.54E-03
GPRC5A	2.95	1.69E-02	*KCNJ8*	2.37	1.03E-04
GPT2	0.34	1.88E-03	*KCNK3*	0.36	3.26E-03

Gene ID	Fold Change: HF LA versus NF LA	FDR
KCNN2	0.52	3.40E-02
KCTD1	2.02	7.48E-05
KIAA1671	0.55	1.32E-02
KIF23	0.30	4.62E-02
KLHL21	0.53	4.98E-02
KLHL36	3.19	1.86E-08
KLHL41	0.35	1.72E-02
KRBA1	2.08	5.27E-03
LAIR1	0.32	3.65E-03
LAMA4	0.46	2.50E-02
LCNL1	0.35	4.23E-03
LCP1	0.45	3.19E-02
LEPR	2.64	1.40E-02
LGALS12	0.06	1.32E-02
LHX6	0.18	4.32E-03
LIN7A	2.32	3.89E-04
LINC00222	1.97	3.36E-02
LINC00473	0.19	1.43E-02
LINC00598	0.31	1.16E-02
LINC00641	1.70	3.12E-02
LINC00702	0.43	2.04E-02
LINC00881	2.15	2.37E-02
LINC01105	6.59	6.76E-03
LINC01134	0.05	7.00E-03
LINC01291	0.03	1.69E-03
LINC01293	0.06	2.75E-02
LINC01355	1.92	1.72E-02
LINC01504	2.85	4.84E-02
LINC01695	0.32	7.40E-03
LINC01697	0.20	1.87E-03
LINC02137	3.56	2.80E-04
LINC02188	0.10	3.19E-02
LINC02432	6.28	4.85E-08
LINC02541	0.13	1.77E-02
LIPE	0.17	5.08E-04
LMBR1L	1.66	3.53E-03
LMCD1	0.52	1.32E-02
LMOD1	0.34	5.63E-04
LPAR3	1.92	4.69E-02
LRRC10	0.23	1.51E-05

Gene ID	Fold Change: HF LA versus NF LA	FDR
LRRC8A	1.67	1.14E-02
LTBP1	2.33	1.31E-02
LVRN	0.09	1.92E-05
LZTS1	0.29	1.18E-02
MAF	0.38	1.09E-02
MAP3K21	0.39	4.57E-02
MAP3K3	1.52	3.64E-02
MAPK4	2.20	7.14E-03
MARCKS	0.55	6.74E-03
MARS2	2.31	1.38E-02
MBD4	1.68	2.24E-02
MCL1	1.66	2.57E-02
MDK	4.35	5.44E-04
MFSD2B	2.74	4.57E-03
MIR1-1HG	0.52	2.00E-02
MKI67	0.12	1.70E-03
MKNK2	2.97	9.95E-07
MLIP-AS1	1.66	4.76E-02
MLXIPL	0.10	3.26E-03
MMP19	3.12	1.50E-03
MNDA	0.24	3.83E-02
MOB3C	1.87	4.98E-03
MORF4L1P3	2.18	1.28E-02
MPEG1	0.38	6.33E-03
MPP3	1.63	3.12E-02
MRPL50	1.60	4.67E-02
MRVI1	0.37	5.85E-03
MS4A6A	0.49	4.73E-02
MT1E	2.35	6.69E-03
MT1X	2.82	2.48E-02
MT-CO2	0.40	2.06E-04
MT-CYB	0.48	1.35E-02
MTMR14	1.77	2.83E-02
MT-ND1	0.53	2.86E-02
MT-ND2	0.48	3.55E-03
MTND2P28	0.43	1.12E-02
MT-ND3	0.44	3.38E-02
MT-ND4L	0.52	2.66E-02
MT-RNR2	0.46	4.28E-04
MTUS2-AS1	0.37	4.19E-03

Gene ID	Fold Change: HF LA versus NF LA	FDR
MUC3A	0.41	3.39E-02
MYC	2.21	7.15E-04
MYH7B	0.44	9.52E-04
MYLK	0.50	2.03E-02
MYO10	0.55	4.84E-02
MYO5C	0.52	1.79E-02
MYO7A	0.37	1.08E-02
MYOCD	0.53	1.28E-02
MYOF	0.59	3.73E-02
MYRFL	0.15	6.71E-03
MZT2A	2.39	2.86E-02
NAV1	0.58	1.97E-02
NAXD	1.63	1.82E-02
NCF4	0.21	5.36E-04
NCKIPSD	1.89	1.31E-02
NEK7	0.48	2.37E-04
NFKBIA	2.26	1.50E-05
NID2	1.71	1.32E-02
NIN	0.53	1.06E-03
NLRP3	4.08	3.12E-04
NMUR1	0.08	4.57E-03
NOTCH3	0.49	1.92E-03
NOX5	0.11	1.21E-02
NPR3	3.01	7.14E-03
NRAP	0.52	4.61E-02
NRN1	0.30	6.21E-03
NRROS	0.20	7.49E-03
NRXN3	3.07	5.69E-05
NSUN5P2	3.67	2.89E-03
NTM	3.55	3.77E-02
NTRK2	2.87	1.80E-04
NXN	0.54	4.91E-02
NXT1	2.29	1.74E-02
OIT3	4.38	1.93E-04
OPCML	5.00	3.53E-03
OR10P1	2.77	2.34E-02
PA2G4P4	2.03	2.89E-03
PARP10	0.30	4.69E-02
PCA3	0.14	1.72E-02
PCDH18	0.34	9.43E-03

Gene ID	Fold Change: HF LA versus NF LA	FDR
PCOLCE2	1.93	6.33E-03
PCTP	2.06	3.46E-02
PDGFRB	0.35	1.03E-04
PDK4	5.15	2.70E-07
PDLIM7	0.40	1.65E-04
PER2	1.79	2.79E-02
PHACTR3	0.25	8.17E-04
PHLPP2	1.66	4.55E-02
PI4KAP1	2.46	2.93E-02
PKD1L1	0.51	3.96E-02
PKMP3	2.30	2.36E-02
PLA1A	1.89	4.95E-02
PLA2G12A	1.67	6.21E-03
PLA2G4F	0.18	2.12E-02
PLEK	0.25	5.42E-04
PLEKHF1	3.00	1.09E-04
PLEKHJ1	1.87	3.08E-02
PLIN1	0.16	2.43E-02
PLK2	0.53	2.47E-02
PLOD2	0.37	3.03E-04
PLTP	0.35	2.15E-02
PLXDC2	1.79	4.90E-03
POGLUT1	2.29	1.11E-03
POSTN	4.59	1.49E-09
POU2F2	0.34	1.64E-02
PPARGC1B	0.39	1.54E-04
PPP1R12B	0.56	1.73E-02
PPP1R15B	1.58	2.62E-02
PPP1R1C	0.49	4.67E-02
PPP1R3B	0.49	9.54E-03
PRAG1	0.37	2.89E-02
PRDX6	1.85	5.22E-03
PRELID2	0.35	3.26E-03
PROM1	0.47	4.70E-02
PROX1	0.49	5.36E-04
PSMD5-AS1	1.59	3.12E-02
PTGS1	2.70	3.90E-02
PTGS2	3.50	1.24E-02
PTP4A3	0.56	4.07E-02
PTPN6	0.36	1.40E-02

Gene ID	Fold Change: HF LA versus NF LA	FDR
PTPRO	0.25	3.12E-02
PWAR1	1.94	2.12E-03
R3HDM1	0.56	2.20E-02
RAB8B	1.90	1.43E-02
RASD1	8.17	1.60E-17
RASD2	0.18	8.81E-05
RASGRF2	0.41	4.66E-03
RBFOX1	0.58	3.22E-02
RBPMS2	0.59	2.02E-02
RGS1	3.18	3.00E-03
RGS2	2.16	5.22E-03
RGS3	0.49	8.16E-03
RGS5	0.37	9.99E-04
RNASE1	0.48	4.86E-02
RNASE2	0.09	1.14E-02
RNF133	0.23	7.49E-03
RNF165	0.35	1.01E-03
RNU6-709P	0.02	2.61E-03
RNY1P16	3.56	1.35E-03
ROR2	2.03	1.93E-04
RPL3L	0.37	3.36E-08
RPS20P22	0.53	4.70E-02
RPS27AP11	0.05	3.17E-02
RSRP1	1.68	1.38E-02
RUNX1T1	0.58	2.43E-02
S100A8	0.19	4.04E-03
S100A9	0.20	1.05E-02
SAA1	0.04	2.37E-04
SAA2	0.07	3.47E-02
SALL4P1	0.05	5.42E-04
SAMD4A	0.60	1.80E-02
SCD	0.21	1.22E-02
SEMA4A	3.16	8.84E-03
SEMA6B	0.46	3.19E-02
SERTAD4	0.26	2.34E-02
SETBP1	1.63	1.46E-02
SHISA4	0.40	1.79E-03
SHOX2	0.05	3.36E-08
SIGLEC1	0.26	8.24E-04
SIRPB2	0.31	4.07E-02

Gene ID	Fold Change: HF LA versus NF LA	FDR
SKAP2	0.32	3.13E-03
SLC12A2	0.58	1.97E-02
SLC16A9	2.84	3.17E-02
SLC19A2	2.62	3.36E-08
SLC22A3	0.19	3.95E-03
SLC22A5	2.16	1.51E-04
SLC25A18	2.34	2.54E-03
SLC25A29	1.78	8.20E-03
SLC26A2	2.16	1.14E-02
SLC29A4	0.27	3.95E-03
SLC2A13	2.78	6.45E-03
SLC35F1	0.23	3.26E-03
SLC39A8	1.90	2.79E-02
SLC45A3	0.10	5.69E-05
SLC4A3	0.64	3.12E-02
SLC5A12	4.15	4.73E-03
SLC6A4	2.93	3.09E-03
SLC7A8	2.42	7.24E-04
SLC9A7P1	7.91	2.38E-06
SLCO1C1	2.44	4.38E-03
SLIT3	1.89	1.46E-02
SMAD4	1.53	4.72E-02
SMAD7	3.02	1.75E-07
SMG1	1.73	1.03E-02
SMYD1	0.46	2.81E-03
SMYD2	2.82	2.72E-02
SNCG	0.41	4.76E-02
SOX18	0.29	2.26E-03
SPN	0.28	7.24E-04
SPRTN	1.78	1.28E-02
SPRY2	1.68	7.22E-03
SPRYD4	1.60	4.23E-02
SREBF1	0.29	7.62E-05
SREBF2	1.64	5.66E-03
SSH2	0.63	4.43E-02
ST3GAL5	0.51	4.57E-02
ST6GALNAC4P1	3.07	7.55E-03
STAB1	0.38	1.37E-02
STAMBPL1	0.41	2.76E-02
STC1	4.31	2.20E-08

Gene ID	Fold Change: HF LA versus NF LA	FDR
STC2	0.33	1.96E-03
STEAP4	0.37	9.55E-03
STK35	2.05	6.15E-03
STK38	0.61	1.18E-02
STK39	0.56	7.53E-03
STK40	0.62	2.00E-02
STX3	0.55	1.45E-02
SULF1	2.21	3.62E-02
SUMO2P8	1.78	3.60E-02
SUPT16HP1	0.16	7.07E-04
SUSD6	1.60	6.70E-03
SYNM	0.42	1.05E-04
SYT2	0.38	2.88E-04
SYT7	0.49	2.16E-02
TACR2	1.96	2.73E-03
TAF12	0.55	3.57E-02
TANC1	1.59	4.42E-02
TBX2	0.21	5.08E-04
TBX3	0.28	5.71E-06
TDRD9	3.95	1.33E-02
TF	0.31	2.75E-02
TGFB2	0.29	1.39E-03
THBD	1.84	3.17E-02
THRSP	0.08	4.84E-03
TIAM1	2.20	3.18E-02
TIPARP	1.95	2.62E-05
TM4SF1	2.39	2.03E-02
TMEM100	3.29	3.34E-04
TMEM144	0.57	3.60E-02
TMEM2	1.76	3.24E-02
TMEM38A	0.37	1.24E-02
TMEM40	0.31	1.96E-02
TNFAIP3	2.38	8.07E-05
TNFRSF10D	3.07	1.20E-02
TNFRSF11B	0.31	4.73E-02
TNFSF10	0.31	1.12E-02
TOB2	2.25	9.87E-05

Gene ID	Fold Change: HF LA versus NF LA	FDR
TOP2A	0.21	1.66E-02
TPM2	0.52	3.53E-03
TPM3	0.38	9.96E-03
TPPP3	2.60	3.36E-03
TRIM2	0.63	2.65E-02
TRIM52	1.61	4.07E-02
TRIM55	0.58	4.86E-02
TRPC6	2.78	1.19E-02
TRPM2	0.28	3.48E-03
TTC23	0.43	1.20E-02
TUBA3D	4.26	5.69E-04
TUBA3E	3.64	2.73E-02
TUFT1	0.39	1.86E-02
TXNL4B	2.00	3.68E-02
UBL5P3	0.20	3.63E-02
UCP2	0.47	4.70E-02
UGT2A3P7	0.18	3.99E-02
UNC13D	0.26	3.36E-08
USP31	1.71	6.15E-03
VIT	0.26	1.69E-05
VPS26BP1	0.45	1.30E-02
VTCN1	0.48	4.14E-02
WDR62	0.34	6.21E-03
WNT2B	2.09	2.37E-04
XRRA1	0.45	7.24E-03
Z99289.1	0.16	3.24E-05
Z99289.2	0.16	1.64E-02
ZBTB5	1.69	2.19E-02
ZC3H12A	1.86	4.01E-02
ZFP36	1.98	4.66E-03
ZNF189	2.68	1.80E-04
ZNF330	1.71	1.99E-02
ZNF385B	2.54	2.73E-02
ZNF460	1.56	4.95E-02
ZNF721	0.60	1.31E-02
ZNF778	1.59	3.80E-02
ZNRF1	0.59	1.72E-02

Cardiomyocyte nuclei (CMN) were sorted from the left atrium (LA) of non-failing human hearts (NF, n = 7) and failing hearts (HF, n = 3), RNA was isolated, and RNA-sequencing

was performed. Genes with a linear fold change ≤ 0.67 and ≥ 1.5 with a false discovery rate (FDR) < 0.05 were considered significant and are included in the table.

Table S8. Comparison of significantly dysregulated transcripts in human atrial samples with atrial fibrillation, heart failure, or non-failing cardiomyocyte nuclei and their associated fold change

Gene ID	Fold Change: AF+HF LA versus NF LA	Fold Change: HF LA versus NF LA	Fold Change: NF LA versus NF RA	Fold Change: AF+HF RA versus NF RA	Fold Change: HF RA versus NF RA
AB015752.1	1.55		0.46	0.64	
ABCA1	0.45	0.25	1.83		
ABCA12			0.42	0.65	
ABCC9	1.95		0.62		
ABCG1	0.34	0.37	2.78		
ABLIM2	0.43		1.58		
ABTB2			2.32	2.42	
AC004053.1			0.41	0.59	
AC004830.1			0.33	0.67	
AC005165.1			0.41	0.52	
AC005550.3		0.16	2.49		
AC008464.1			0.37		0.39
AC008700.1			0.50		0.41
AC008705.1			0.36		0.25
AC009084.2		2.37	0.46		
AC009411.2		0.03	7.04		
AC011752.1		0.05	5.51		
AC018742.1		0.07	9.85		
AC021683.2	0.24		0.49		
AC024940.1		0.05	0.12		0.06
AC025034.1			0.52	0.56	
AC025271.4			0.21		0.10
AC025741.1	1.93	3.02	0.34		
AC083902.2	0.29		3.66		3.22
AC090229.1		3.06	0.33		
AC098864.1	12.01		0.34		
AC104806.2	1.90		0.35		
AC104958.1		0.18	3.17		
AC106801.1		0.23	2.07		
AC107980.1		0.07	3.78		
AC109779.1		0.26	4.43		
AC112203.1			0.19		0.08
AC114316.2			0.34	0.56	
AC117529.1			0.35		0.36
AC131902.3			0.14		0.06
AC243562.2		4.09	0.39		
AC244021.1			0.19	0.40	

Gene ID	Fold Change: AF+HF LA versus NF LA	Fold Change: HF LA versus NF LA	Fold Change: NF LA versus NF RA	Fold Change: AF+HF RA versus NF RA	Fold Change: HF RA versus NF RA
ACTA1			5.28		3.40
ACTG2	0.51	0.31	4.78		
ACVRL1	0.66		1.59		
ADAMTS3			0.40	0.52	0.46
ADAMTSL2	0.48		0.34	0.56	
ADD2	0.58		2.29		
ADGRF5	0.55		1.64	0.51	
ADGRG6		2.07	0.45	0.51	
ADIPOQ		0.16	12.19		
ADM	3.26	3.27	0.32		
ADRA1A		0.14	2.80		
AGAP2		0.24	2.56		
AIF1L	0.45	0.42	2.77		
AL008628.1		3.79	0.40		
AL009031.1			0.55		0.33
AL022068.1	1.61		0.21	0.55	
AL031686.1			0.35	0.43	
AL157714.2		0.29	3.05		
AL159166.1		0.27	2.44		
AL359075.2			0.52	0.57	
AL450384.2		2.09	0.52		
ALCAM			0.39	0.27	
ALDH1B1	0.58		1.80	0.54	
ANGPT1		5.24	0.33	0.31	
ANKRD29	0.35		2.12		
ANKRD45	0.12		2.99		
ANO1	0.64		1.90		
ANXA3	0.61		2.53	0.54	
ANXA8L1	0.36		2.19		
AOC3			2.22	0.46	
APOD	0.24	0.16	6.59		
APOL3	0.45		1.71		
APOLD1			1.92		2.53
ARHGAP20			2.91	0.62	
ARHGDIB	0.62		1.80	0.45	
ARHGEF4	0.38	0.24	2.64		
ART3			0.54	0.56	
ASPG			0.53	1.68	
ASPN			2.48		2.91
AZIN1			1.56	1.71	

Gene ID	Fold Change: AF+HF LA versus NF LA	Fold Change: HF LA versus NF LA	Fold Change: NF LA versus NF RA	Fold Change: AF+HF RA versus NF RA	Fold Change: HF RA versus NF RA
BCL2			2.42	1.87	
BMP10	50.67	163.24	0.00		
BRINP3			0.31	0.29	
BST1	0.61		0.55		
BTNL9	0.40	0.36	2.07		
C1GALT1P1		1.89	0.48		
C1QC	0.49		1.76	0.19	
C3AR1			2.02	0.47	
C3orf52	3.29	4.16	0.24		
CACNA1D	0.46		0.19		
CACNA2D2	0.49		0.25		
CACNB2			0.60	2.03	
CADPS2		0.25	2.22		
CARMIL1		2.34	0.55		
CASC19			0.30	0.61	0.27
CASKIN1			0.24		0.35
CAV1	0.44	0.58	1.50		
CBFA2T3	0.45		2.09		
CCDC158			0.45	0.61	
CD209	0.42	0.25	2.29	0.59	
CD63			1.57	0.52	
CD82	0.56		1.96		
CDCA2	0.48	0.24	2.20		
CDH5	0.59		1.60	0.61	
CDKN1C		0.41	2.83		
CEACAM19		0.36	1.82		
CES3			0.46		0.31
CFAP221	1.62		0.46		
CFAP46	0.45		0.39		
CGNL1	0.59		0.62	0.50	
CHDH	0.50		0.49		
CHST11	0.64		1.52	1.55	
CILP	0.31		5.41		
CLEC2D			0.60		0.51
CLMAT3			4.43	0.56	
CLSTN2	0.51		1.57		
CLU			2.01	0.50	
CMKLR1	0.46		2.05		
CNTN3			0.56	0.54	
COL6A4P2			0.26	0.52	

Gene ID	Fold Change: AF+HF LA versus NF LA	Fold Change: HF LA versus NF LA	Fold Change: NF LA versus NF RA	Fold Change: AF+HF RA versus NF RA	Fold Change: HF RA versus NF RA
COL9A1	2.58	8.43	0.17	0.48	
COLEC12			2.91		1.93
COLGALT1	0.63		1.56		
COX6A2			1.51	0.34	
CPAMD8	0.52	0.33	2.72		
CPNE5		1.90	0.50	2.64	
CREB3L1	0.56		1.90		
CRIM1			1.86		1.61
CRYAB			1.52	0.61	
CSF1R	0.58		1.56	0.65	
CSRNP3			1.61	0.62	
CSRP3	0.45	0.51	1.58		
CST3			1.75	0.50	
CSTB	0.57		1.52	0.45	
CTIF	0.59		1.53	1.61	
CTSC			1.90	0.57	
CTSL3P	0.42		0.37	0.54	
CTSZ			1.52	1.73	
CXCL12	0.40		2.14	0.47	
CXXC4	1.56		0.59	0.58	
DAB1			0.25	0.63	
DAB2		0.35	2.24	1.62	
DACH1	0.52		2.01	0.52	
DDX18P3			0.26		0.36
DERL3		3.72	0.48		
DGAT2	0.36	0.16	5.26		
DGKG			1.75	2.50	
DHRS9		0.32	4.30		
DNASE1L3			0.10	0.58	
DNER	2.24	4.10	0.26		
DNM1	0.61	3.88	1.85		
DNM1P51		3.88	0.35		
DOK3		0.21	2.04		
DPP3P1			0.41	0.40	
DPY19L2P3			0.58	0.54	
DSC3	0.34		3.56		
DTX4	0.51		1.92	0.61	0.35
DUSP10	0.42	0.38	1.83		
DUSP6			1.80	2.32	
E2F2			0.21		0.18

Gene ID	Fold Change: AF+HF LA versus NF LA	Fold Change: HF LA versus NF LA	Fold Change: NF LA versus NF RA	Fold Change: AF+HF RA versus NF RA	Fold Change: HF RA versus NF RA
EBF2	0.31	0.43	2.01	0.54	
EGFLAM	0.49		3.60		
EGFR	1.65		1.52	1.83	2.22
EHD1	0.52		1.93		
EHD2	0.64	0.54	2.15	0.60	
EIF4EBP2			1.79	1.64	
ELN	0.41	0.44	1.94		
EMB			2.73	0.56	
EMC10			0.60	6.02	
EPHA5			9.73		4.00
EPHA7	2.21	1.74	0.64		
EPS8		0.59	1.92		
ERC2		0.48	1.58		
ETV1		0.62	1.82		
F13A1	0.35	0.22	2.20	0.42	
FABP4		0.17	6.07		
FABP5		0.39	1.88		
FADS2	0.45		1.68		
FADS3	0.55		1.54	1.52	
FAM189A1	0.36		5.14		
FAM78B			2.17	1.90	
FAM89A	0.62	0.30	3.49		
FASN		0.11	4.85	0.11	
FBN1	0.56		1.57	0.50	
FBN2	0.38		3.72		
FGFR3		2.77	0.45		
FLJ46284			0.55	0.53	
FLNC	0.28		1.84		
FMO3			0.41	0.66	
FMO6P			0.38	0.62	
FRMD6	0.66		1.75	0.59	
FRRS1			0.48	0.61	
GADD45G			1.96		2.34
GALNT16			1.95		1.80
GATA5		0.23	6.43		
GBP2	0.65		1.53	0.46	
GCSAM		3.47	0.34		
GEM	0.35		2.81		
GFOD1			1.53	1.79	
GIMAP4	0.50	0.54	1.50	0.44	

Gene ID	Fold Change: AF+HF LA versus NF LA	Fold Change: HF LA versus NF LA	Fold Change: NF LA versus NF RA	Fold Change: AF+HF RA versus NF RA	Fold Change: HF RA versus NF RA
GIMAP6			1.66	0.60	
GLDN	1.75		0.28		
GOLGA8S			0.30		0.32
GPA33	0.58		2.50		
GPAM			2.28	0.44	
GPATCH8			0.60	1.69	
GPC5	1.61		0.33		
GPD1		0.22	3.11		
GPR153			1.69		2.04
GPT2	0.48	0.34	2.42		
GPX1			1.59	0.19	
GRIN2A	0.43		1.77		
GRIP2	0.54	0.57	1.86	1.76	
GRXCR2	0.08	0.09	0.37		0.14
GUCY1A2	0.64	0.36	3.32	0.59	
H19			1.79	0.25	
HAMP		16.63	0.01		
HCN1			0.44		0.49
HELLPAR	0.64		0.49	0.63	
HEPACAM	0.38		4.15		
HES6			0.32		0.21
HIST1H1D			0.44		0.15
HK2	0.64	0.37	2.24		
HMCES	0.60		1.60		
HMCN2	0.52		3.37		
HOXB3		0.16	2.30		
HRC	0.65		1.70	0.53	
HSD17B7P2			0.51	0.64	
HSPA12B		0.39	2.99		
HSPA8			1.76	0.52	
HSPB1			1.65	0.55	1.56
HTR4	0.29	0.23	2.37		
IDH2	0.38		1.69		
IFITM2			1.56	0.29	
IGF1	0.32	0.26	2.23	2.15	
IGFBP4	0.57		1.68	0.45	
IGFN1	2.13		0.10		
IGSF11	2.16		0.66		
IL15			0.61	0.57	
IL2RA		0.21	3.72		

Gene ID	Fold Change: AF+HF LA versus NF LA	Fold Change: HF LA versus NF LA	Fold Change: NF LA versus NF RA	Fold Change: AF+HF RA versus NF RA	Fold Change: HF RA versus NF RA
IL5RA			0.36	0.51	
INPP5J	0.44		0.60		
IRF4			0.37		0.26
IRS2			1.83		1.77
ITGA2B			0.19		0.35
ITGBL1	0.64		1.89	0.49	
ITIH5	0.49	0.46	2.96		
KCNH7			0.09	0.43	0.21
KCNN2	0.47	0.52	1.78		
KCTD16			0.28	0.57	
KDR			1.70	0.49	
KIAA1324L	1.65		0.45	0.54	
KIAA1549L			0.29	0.62	
KIF6			0.29	0.61	
KLHL41		0.35	1.87		0.55
LAMA4	0.43	0.46	2.09		
LDLRAD4	1.85		0.65	2.01	
LGALS1			1.62	0.27	
LHX6	0.53	0.18	3.25		
LIN7A	2.44	2.32	0.64		
LINC00282			0.62	0.64	
LINC00299	0.51		0.33	0.53	
LINC00484			0.64	1.65	
LINC00535			0.48	0.66	
LINC00571			0.29	0.50	
LINC00649	0.62		0.64		
LINC00702		0.43	2.58		
LINC00964	0.65		0.46		
LINC01237			0.20		0.26
LINC01252	0.52		0.51	0.58	
LINC01359	1.73		0.61	1.86	
LINC01881	1.76		0.55		
LINC02137	2.07	3.56	0.44	3.17	
LINC02334			0.23	0.51	
LINC02541	0.28	0.13	17.86		
LIPE		0.17	4.05		
LPL			3.08	3.18	2.53
LRP1B	0.27		3.11		
LRRC20	0.59		2.09		
LRRCC1			0.53	0.63	

Gene ID	Fold Change: AF+HF LA versus NF LA	Fold Change: HF LA versus NF LA	Fold Change: NF LA versus NF RA	Fold Change: AF+HF RA versus NF RA	Fold Change: HF RA versus NF RA
LTF			7.04	0.66	
LVRN		0.09	4.18		
LY9			0.37	0.64	
LZTS1	0.40	0.29	2.02		
MAPT	0.42		1.63		
MCHR1			0.31		0.14
MED30	1.80		2.99		
MEGF10			0.33	0.56	
MEGF9	0.64		1.75		
MEOX1	0.48		2.57		
MEOX2	0.46		2.38	0.47	
MFGE8			1.85	1.83	
MGLL	0.38		1.80		
MIR100HG			0.45	0.51	
MIR4432HG	0.64		0.25		
MOCS1			1.63	1.52	
MRVI1	0.42	0.37	1.64		
MS4A4A			1.90	0.55	
MT-ATP6	0.39		1.65	0.13	
MT-ATP8	0.52		1.70	0.13	
MT-CO1	0.50		1.75	0.18	
MT-CO2	0.40	0.40	1.86	0.13	
MT-CO3	0.53		1.70	0.19	
MT-CYB	0.40	0.48	1.70	0.12	
MT-ND1	0.40	0.53	1.86	0.15	
MT-ND2	0.38	0.48	1.72	0.13	
MTND2P28		0.43	1.88		
MT-ND3		0.44	2.06	0.16	
MT-ND4	0.41		1.89	0.17	
MT-ND4L	0.41	0.52	1.89	0.18	
MT-ND6	0.53		1.77	0.18	
MYCT1	0.50		2.09	0.42	
MYH9	0.41		1.54	1.61	
MYL2	0.25		8.24		
MYOC	0.63		5.12		4.67
MYRIP			0.61	0.64	
NCKIPSD	1.75	1.89	0.64	1.65	
NDNF	0.50		3.35	0.62	
NDRG4			1.60	2.97	
NECTIN3-AS1			0.37	0.54	

Gene ID	Fold Change: AF+HF LA versus NF LA	Fold Change: HF LA versus NF LA	Fold Change: NF LA versus NF RA	Fold Change: AF+HF RA versus NF RA	Fold Change: HF RA versus NF RA
NES	0.51		1.83		
NEURL1B	0.66		2.33		
NFASC	1.69		0.56	2.42	
NMUR1	0.13	0.08	3.60		
NOTCH3	0.56	0.49	3.21		
NPPB	4.91		5.38		
NPR3		3.01	0.28		0.59
NRN1		0.30	2.17		
NTM		3.55	0.12		
NWD2	0.56		5.00		
OBSL1	0.60		0.61		0.60
OR10P1		2.77	0.46		
OR7E2P			0.41		0.19
ORC6	1.94		0.59		
OSBP2	0.58		1.57		
OSGIN2	1.60		0.61	0.65	
OSMR-AS1	1.66		0.52		
P2RX3	0.43		9.34		
P2RY2			2.74	2.30	
PAQR3	1.99		0.59		
PCDH1	0.48		2.69		
PCDH15			0.44	0.54	
PCDH18	0.48	0.34	1.78	0.38	
PDCD4	1.62		0.59		
PDE1A	0.63		0.54	0.44	
PDE9A	0.49		2.05		
PDGFRB	0.33	0.35	1.74		
PDLIM7	0.29	0.40	1.76		
PGM2L1	0.52		2.13		
PIK3R1	2.08		1.57	1.77	1.68
PIM1			1.54		1.73
PITX2	0.37		17.67		
PKN1	0.60		1.54	1.64	
PLCB2	0.38		0.53		
PLCH1			0.24	0.60	
PLEC			1.53	1.97	
PLIN1		0.16	5.83		
PLK2	0.59	0.53	1.65		
PLTP	0.36	0.35	2.42		
PLXNA1			1.55	1.60	

Gene ID	Fold Change: AF+HF LA versus NF LA	Fold Change: HF LA versus NF LA	Fold Change: NF LA versus NF RA	Fold Change: AF+HF RA versus NF RA	Fold Change: HF RA versus NF RA
POLQ			0.58	0.64	0.32
POLR2L	0.65		1.55	0.40	
POPDC2			1.54	1.77	
POSTN	5.82	4.59	0.26		
POTEJ			0.10	0.23	6.45
POU5F1B			0.18		0.22
PPA1	0.66		1.92		
PPARG			1.83	0.64	
PRAG1	0.56	0.37	2.49		
PRELID2	0.49	0.35	0.52		0.35
PRICKLE1	2.32		0.45		
PRSS35			0.19	0.40	
PTGS2		3.50	0.36		
PTN			2.21	0.62	2.33
PTPRE	0.58		1.66	1.67	
PTPRQ			0.50	0.43	
RANBP17			0.57	0.61	
RAP1GAP	0.48		0.49		
RAPGEF4-AS1			0.50	0.65	
RASD2		0.18	2.66		
RASGRF2	0.54	0.41	1.90	0.61	
RCAN2			1.64	1.80	
RETSAT	0.55		2.19		
RGCC			2.20		3.13
RGS5	0.62	0.37	2.77	0.48	
RGS6	0.44		0.66		
RGS9			0.49	0.66	
RNASE1	0.51	0.48	2.13	0.22	
RNF207			0.64	1.87	
RNU6-709P		0.02	3.90		
RNY1P16		3.56	0.47		
ROBO2	0.41		3.78		
ROGDI	0.58		0.64		
ROR2	1.64	2.03	0.52		
RPL3L	0.42	0.37	2.44		
RPS6KA2	1.70		1.53	1.72	
RRAS	0.62		1.63		
RSAD2			2.07	0.62	
RTN3	0.64		1.57		

Gene ID	Fold Change: AF+HF LA versus NF LA	Fold Change: HF LA versus NF LA	Fold Change: NF LA versus NF RA	Fold Change: AF+HF RA versus NF RA	Fold Change: HF RA versus NF RA
S1PR1			1.65	0.61	1.64
SAA1	0.13	0.04	17.12		
SALL4P1		0.05	4.60		
SCD		0.21	3.64	0.18	
SCGN			0.11	0.49	
SCML4			0.45	0.64	
SELP			0.56	0.58	
SEMA6B		0.46	2.20		
SEPT7P9			0.50	0.53	
SEPT9	0.60		1.71		
SERPINE1	6.85		4.26	3.58	3.44
SERPINI2	0.30		0.25	0.32	
SH2D3C	0.58		2.16		
SH3RF3			1.63	1.54	
SHOX2	0.08	0.05	5.18	0.46	
SIX1	0.43		5.41		
SLC12A2		0.58	1.52		
SLC15A2			0.34	0.56	
SLC1A3			1.54	2.32	1.82
SLC22A3	0.33	0.19	4.38		
SLC22A4			0.64	1.73	
SLC22A5	1.70	2.16	0.61	1.87	
SLC26A5			0.41	0.58	
SLC27A6	0.51		2.16		
SLC29A4	0.45	0.27	2.59		
SLC35F1	0.35	0.23	2.22		
SLC39A8	1.68	1.90	0.66	0.67	
SLC5A12		4.15	0.11	0.33	
SLC7A11			0.27	0.43	
SLC7A11-AS1	0.57		0.34	0.43	
SLC9A3R1			1.91	1.79	
SLC9A7P1		7.91	0.37		
SLCO2B1	0.58		1.98		
SLIT3		1.89	0.59		
SLN			0.39	0.35	
SMAD6	2.13		0.38		
SMAD7	1.98	3.02	0.36		
SMAD9	2.72		0.33	0.55	
SMOC2	0.64		3.69		2.38
SMYD2	3.71	2.82	0.35		2.11

Gene ID	Fold Change: AF+HF LA versus NF LA	Fold Change: HF LA versus NF LA	Fold Change: NF LA versus NF RA	Fold Change: AF+HF RA versus NF RA	Fold Change: HF RA versus NF RA
SNCG		0.41	2.18		
SNTB1	0.39		2.93	0.63	
SOD3	0.39		1.97		
SORL1	0.64		1.62		
SOX18		0.29	1.89		
SOX9-AS1	1.78		0.49		
SPARC			2.01	0.43	
SPARCL1			1.58	0.50	
SPN		0.28	2.58		
SPRY1			1.88		1.85
SPTLC3			0.58	0.62	0.50
STARD4			0.40	0.59	
STC2	0.51	0.33	2.13		0.46
STEAP4		0.37	2.08		
STIM2			0.60	0.49	
STK39		0.56	1.58		
STX2	1.69		1.61	2.58	
SULT1A1			0.65		0.56
SVEP1			1.61	0.55	
SYNPO	0.61		1.54	1.53	
SYT13	0.50		0.47		0.18
TAGLN	0.46		1.58		
TCAP			1.90	0.39	
TCF24	0.51		1.79	1.96	
TDH	0.50		3.61		
TGFB2	0.41	0.29	2.41		
THBD		1.84	1.72		
THRSP		0.08	6.73		
TIAM1		2.20	0.49	0.58	
TMEM108			0.44	0.65	
TMEM132B	0.53		0.24	0.64	
TMEM2		1.76	0.61	1.72	
TMEM38A	0.45	0.37	1.89		
TNFRSF11B		0.31	2.36		
TNFRSF19	0.35		1.50		
TNK2			0.59	2.15	
TNNT1			1.71	3.65	
TNS3	0.63		1.65		
TOX	0.42		3.23		
TRAC	0.08		2.93		

Gene ID	Fold Change: AF+HF LA versus NF LA	Fold Change: HF LA versus NF LA	Fold Change: NF LA versus NF RA	Fold Change: AF+HF RA versus NF RA	Fold Change: HF RA versus NF RA
TRIM45			0.64		0.65
TRPC4			0.30	0.38	
XIRP1		2.78	0.44	0.60	
TRPM2		0.28	2.57		
TSC22D1			1.61	2.16	
TSHZ2	0.50		1.58	0.60	
TSKU	0.33		2.40		
TSSK3			0.62	1.61	
TUBB	0.62		1.62	0.49	
TUBB4B			1.62	0.57	
UNC13D	0.39	0.26	2.94		
VN2R3P	0.35		0.20		
WDR49	0.45		0.29	0.43	
WISP1	0.58		0.39		
WISP2			0.46		0.52
WSCD2	0.48		0.19		
XIRP1			1.74		1.95
ZBTB7C	0.41		2.02		
ZNF385B	2.95	2.54	0.27	0.61	
ZSCAN30			0.66	1.54	

Cardiomyocyte nuclei (CMN) were sorted from non-failing (NF) human right atria (RA, *n* = 6) and left atria (LA, *n* = 7), from failing hearts with a history of atrial fibrillation (AF+HF) from the RA (*n* = 5) and LA (*n* = 5), from failing hearts (HF) from the RA (*n* = 3) and LA (*n* = 3). RNA was isolated and RNA-sequencing was performed. Genes with a linear fold change ≤ 0.67 and ≥ 1.5 with a false discovery rate (FDR) < 0.05 were considered statistically significant.

Table S9. Known atrial fibrillation GWAS loci and their associated fold change in human atrial fibrillation with heart failure, heart failure alone, and non-failing atrial cardiomyocyte nuclei

Gene ID	Fold Change: AF+HF LA versus NF LA	Fold Change: HF LA versus NF LA	Fold Change: NF LA versus NF RA	Fold Change: AF+HF RA versus NF RA	Fold Change: HF RA versus NF RA
ARHGAP26				1.52	
BEST3	0.29	0.31			0.25
C9orf3	0.59				
CAND2	0.53			1.64	
CASC17				0.60	
CASC20				0.66	
CASQ2	0.60				
CAV1	0.44	0.58	1.50		
CCDC92				1.62	
CDKN1A	1.93	2.14		2.18	
CREB5	1.92				
DGKB	0.55			0.56	
EPHA3				0.56	
FAM161A	2.01				
GNB4	1.59				
HCN4			0.23		
IGF1R				2.15	
KCNH2				1.58	
KCNN2	0.47	0.52	1.78		
KRR1				0.59	
LINC00477				0.55	
LINC00964	0.65		0.46		
LINC01142				0.54	
METTL11B				0.64	
NACA				1.52	
NAV2			1.52		
PHLDA1			3.08		
PHLDB2			2.64		
PITX2	0.37		17.67		
PMVK	0.63				
PPFIA4	0.61				
REC114			0.40		
RPL3L	0.42	0.37	2.44		
SMAD7	1.98	3.02	0.36		
STC2	0.51	0.33	2.13		0.46
SYNPO2L			1.70		
TBX3		0.28			

Gene ID	Fold Change: AF+HF LA versus NF LA	Fold Change: HF LA versus NF LA	Fold Change: NF LA versus NF RA	Fold Change: AF+HF RA versus NF RA	Fold Change: HF RA versus NF RA
TBX5				2.26	0.58
TBX5-AS1				2.57	
XPO1	1.54			1.95	

Cardiomyocyte nuclei (CMN) were sorted from non-failing (NF) human right atria (RA, *n* = 6) and left atria (LA, *n* = 7), from failing hearts with a history of atrial fibrillation (AF+HF) from the RA (*n* = 5) and LA (*n* = 5), from failing hearts (HF) from the RA (*n* = 3) and LA (*n* = 3). RNA was isolated and RNA-sequencing was performed. Genes with a linear fold change ≤ 0.67 and ≥ 1.5 with a false discovery rate (FDR) < 0.05 were considered statistically significant.

Table S10. Significantly dysregulated transcription factors and their associated fold change in human atrial fibrillation with heart failure, heart failure alone, or non-failing atrial cardiomyocyte nuclei

Gene ID	Fold Change: AF+HF LA versus NF LA	Fold Change: HF LA versus NF LA	Fold Change: NF LA versus NF RA	Fold Change: AF+HF RA versus NF RA	Fold Change: HF RA versus NF RA
AFF3	0.51				
AFF4				1.67	
AHR			1.57		
ARID4A					0.63
ARID5A				2.52	
ATOH8			0.54		
BARX2				0.65	
BATF				0.57	
BAZ2A				1.63	
BBX	1.56				
BCL6B			1.61		
BHLHE41		0.54			0.55
BNC2		0.47			
CAMTA2	0.64				
CARHSP1				2.07	
CBFB	1.89				
CEBPA			7.41		
CEBPD	1.95	1.80			1.91
CREB3				2.02	
CREB3L1	0.56		1.90		
CREB5	1.92				
CSDC2				2.55	
CUX1				1.81	
DBX2				0.59	
DEAF1	0.66			1.72	
DMRT1				0.62	
DUXA				0.62	
E2F2			0.21		0.18
E2F4				1.51	
E2F7				0.66	
EAF2				0.60	
EBF1	0.48			0.45	
EBF2	0.31	0.43	2.01	0.54	
EBF3	0.49			0.62	
EGR1	5.40	3.27		4.12	
EGR2	2.06	4.07			
EHF	0.55				

Gene ID	Fold Change: AF+HF LA versus NF LA	Fold Change: HF LA versus NF LA	Fold Change: NF LA versus NF RA	Fold Change: AF+HF RA versus NF RA	Fold Change: HF RA versus NF RA
ELF5				0.67	
ERG				0.62	
ESR1				0.58	
ESRRA				2.38	
ESRRB	0.58				
ETS1				0.58	
ETV1		0.62	1.82		
ETV5	2.00				
ETV6	0.59	0.49			0.60
FLI1	0.55			0.55	
FOSB		9.34			
FOSL2	1.84	1.97		2.60	1.78
FOXB1				0.64	
FOXC1		2.01			
FOXK1				1.79	
FOXK2				2.06	
FOXM1	0.53				
FOXO1	1.61				
FOXO3	2.08	1.79		1.88	1.87
FOXP4				1.86	
GATA3	0.48				
GATA4				1.59	
GATA5		0.23	6.43		
GATA6				2.11	
GATAD2B				1.70	
GBX1	0.32				
GCM1				0.57	
GFI1B	1.99				
GLI3	0.64				
GLIS3				0.56	
GRHL2				0.65	
GTF2I				1.74	
GTF2IRD1				4.36	
GTF2IRD2				2.56	
GTF2IRD2B				2.00	
HES1	2.39				
HES6			0.32		0.21
HEY1			2.14		
HIVEP2	1.67				1.51
HIVEP3	0.57				

Gene ID	Fold Change: AF+HF LA versus NF LA	Fold Change: HF LA versus NF LA	Fold Change: NF LA versus NF RA	Fold Change: AF+HF RA versus NF RA	Fold Change: HF RA versus NF RA
HKR1				1.59	
HLX		0.40			
HNF4G				0.63	
HOPX	3.35				2.47
HOXA2		0.01			
HOXA3		0.11			
HOXB3		0.16	2.30		
HOXB4			2.71		
HSF1				1.61	
ID1				0.20	
ID2			0.55		
ID4			0.47		
IKZF1				0.64	
IKZF2				0.61	
IRF4			0.37		0.26
IRF6			0.60		
IRX3			0.25		
ISX				0.58	
JARID2				1.58	
JDP2				1.54	
JUNB		2.27			
KLF15	2.37			4.62	2.60
KLF17				0.62	
KLF6			1.69		
KLF9	2.32			1.91	
L3MBTL4			0.62		
LEUTX				0.65	
LHX4	0.54				
LHX6	0.53	0.18	3.25		
LHX9				0.56	
LIN28B				0.63	
LITAF	0.57				
MAF	0.47	0.38		0.35	0.54
MAFK				1.84	
MBD4	2.21	1.68		2.37	1.61
MECOM				0.44	0.50
MEF2D				1.90	
MEOX1	0.48		2.57		
MEOX2	0.46		2.38	0.47	
MLXIP				1.71	

Gene ID	Fold Change: AF+HF LA versus NF LA	Fold Change: HF LA versus NF LA	Fold Change: NF LA versus NF RA	Fold Change: AF+HF RA versus NF RA	Fold Change: HF RA versus NF RA
MLXIPL	0.38	0.10			
MXD4				2.29	
MYBL1	0.59				
MYC		2.21			2.08
MYT1			0.31		
NCOR2				1.63	
NFATC1				1.86	
NFATC2				1.69	
NFATC4					0.39
NFIC				2.06	
NFXL1	0.44				
NKX3-1			0.57		
NPAS2				1.90	
NPAS3				0.60	
NR1I2				0.58	
NR2F1			0.64		
NR4A2			0.36		
NR5A2	0.62			0.63	
NRL	0.65				
OSR1			5.11		
PAX1				0.52	
PAX3				0.61	
PIAS1	1.70				
PIAS4				1.83	
PITX2	0.37		17.67		
PKNOX2			0.61		
PLAGL2				1.71	
POU2F2		0.34			
POU5F1B			0.18		0.22
POU6F1	0.63				
POU6F2			0.43		
PPARA	1.55			1.96	
PPARD				1.51	
PPARG			1.83	0.64	
PRDM16	0.60				
PRDM5	1.52				
PRDM9				0.57	
PROX1	0.61	0.49			0.53
RFX1				1.64	
RFX2	0.54				

Gene ID	Fold Change: AF+HF LA versus NF LA	Fold Change: HF LA versus NF LA	Fold Change: NF LA versus NF RA	Fold Change: AF+HF RA versus NF RA	Fold Change: HF RA versus NF RA
RFX3				0.58	
RFX6				0.60	
RORB				0.51	
RXRB				1.78	
SALL1			0.06		
SALL2				0.57	
SATB2				0.56	
SHOX2	0.08	0.05	5.18	0.46	
SIM1				0.60	
SIX1	0.43		5.41		
SIX4			1.70		
SMAD3				1.84	
SMAD4	1.74	1.53			
SMAD6	2.13		0.38		
SMAD7	1.98	3.02	0.36		
SMAD9	2.72		0.33	0.55	
SNAI2				0.40	
SNAPC4				1.84	
SOX18		0.29	1.89		
SOX5				0.43	
SP140				0.62	
SP140L				0.61	
SPI1	0.53				
SREBF1	0.36	0.29			0.46
SREBF2		1.64			
SRF	0.65				
ST18				0.59	
STAT3				1.70	
STAT4			1.73		
STAT5A	0.60				
TADA2B				1.57	
TBX15				0.58	
TBX18				0.44	0.42
TBX19				0.66	
TBX2		0.21			
TBX20			0.62		
TBX3		0.28			
TBX5				2.26	0.58
TCF4				0.48	
TCF7				1.82	

Gene ID	Fold Change: AF+HF LA versus NF LA	Fold Change: HF LA versus NF LA	Fold Change: NF LA versus NF RA	Fold Change: AF+HF RA versus NF RA	Fold Change: HF RA versus NF RA
TEAD2	0.64				
TEAD3				2.10	
TEAD4	0.60				
TFAP2B				0.59	
TFAP2D				0.59	
TFDP2	1.59				
TFEB				2.37	
TFEC				0.58	
TGIF1	1.59				
THAP4				1.83	
TOX	0.42		3.23		
TOX3				0.63	
TP63				0.62	
TRERF1	0.53				
TRPS1				0.66	
TSC22D1			1.61	2.16	
TSC22D4	1.72			2.27	
TSHZ3	0.65				
TULP3			0.59		
TWIST1				0.62	
TWIST2	0.35				
USF2				1.60	
VEZF1	1.60			1.95	
VTN			3.77		
WT1	1.71			0.57	
YBX2				3.12	
ZBTB16	2.13			4.26	2.30
ZBTB17				1.61	
ZBTB38				1.58	
ZBTB41				0.63	
ZBTB43				1.62	
ZBTB46				1.72	
ZBTB48				1.73	
ZBTB49				1.70	
ZBTB7A				1.70	
ZBTB7B				1.76	
ZBTB7C	0.41		2.02		
ZFPM1				2.05	
ZHX2				1.82	
ZMIZ1				2.25	

Gene ID	Fold Change: AF+HF LA versus NF LA	Fold Change: HF LA versus NF LA	Fold Change: NF LA versus NF RA	Fold Change: AF+HF RA versus NF RA	Fold Change: HF RA versus NF RA
ZNF142				1.52	
ZNF180	0.64				
ZNF189	3.35	2.68		2.76	2.44
ZNF214				0.61	
ZNF215				0.64	
ZNF225			0.62		
ZNF251				1.52	
ZNF273			0.59		
ZNF274				1.53	
ZNF276				1.97	
ZNF282				1.75	
ZNF362				1.72	
ZNF366	0.53			0.56	
ZNF423	0.47				
ZNF44				1.62	
ZNF451	1.75				
ZNF460		1.56			
ZNF469			0.61		
ZNF479				0.57	
ZNF492				0.63	
ZNF500				1.72	
ZNF516				1.51	
ZNF536			0.29		
ZNF562				1.55	
ZNF579				1.87	
ZNF586				1.95	
ZNF589					0.50
ZNF623				1.83	
ZNF624	0.45				
ZNF672				1.61	
ZNF677					1.61
ZNF679				0.63	
ZNF680				1.54	
ZNF692				1.74	
ZNF7				1.68	
ZNF70				1.85	
ZNF710	0.62				
ZNF732	1.58				
ZNF737			0.64		
ZNF74				1.57	

Gene ID	Fold Change: AF+HF LA versus NF LA	Fold Change: HF LA versus NF LA	Fold Change: NF LA versus NF RA	Fold Change: AF+HF RA versus NF RA	Fold Change: HF RA versus NF RA
ZNF740				1.64	
ZNF75A				1.51	
ZNF763			0.47		
ZNF774	0.59				
ZNF778		1.59			
ZNF787				1.50	
ZNF800	1.50				
ZNF814				1.81	
ZNF852				1.60	
ZNF865				1.94	
ZNF90	0.55				
ZNF93	0.62				
ZSCAN20			1.63		
ZSCAN30			0.66	1.54	
ZXDC				1.78	

Table showing significantly dysregulated transcription factors from cardiomyocyte nuclei (CMN) that were sorted from non-failing (NF) human right atria (RA, *n* = 6) and left atria (LA, *n* = 7), from failing hearts with a history of atrial fibrillation (AF+HF) from the RA (*n* = 5) and LA (*n* = 5), from failing hearts (HF) from the RA (*n* = 3) and LA (*n* = 3). RNA was isolated and RNA-sequencing was performed. Genes with a linear fold change ≤ 0.67 and ≥ 1.5 with a false discovery rate (FDR) < 0.05 were considered statistically significant. Table is related to **Figures 1D, 1E.**

Table S11. Murine iNICD LA versus control LA differentially expressed gene list

Gene ID	Fold Change: iNICD LA Versus Control LA	Adjusted *P* Value
Notch1	25.35	0.00E+00
Lrrc8e	40.45	4.06E-211
AW011738	12.68	3.40E-167
Myom3	10.34	1.83E-99
Nup62cl	13.56	6.36E-66
Pmm1	2.78	4.57E-64
Mpp2	3.91	1.23E-63
Igfbpl1	6.26	9.86E-61
Pla2g3	10.31	1.92E-60
Nrarp	10.09	2.65E-60
Nacad	0.15	1.05E-55
2900052N01 Rik	10.45	9.76E-51
Nat8l	6.72	2.82E-48
Cck	10.12	2.30E-46
Card11	5.57	1.06E-44
Nt5dc2	0.43	2.85E-42
Ctdspl	2.46	1.76E-41
Hes1	3.70	3.66E-41
Aldh1b1	5.34	1.80E-39
Clasp1	2.45	1.71E-38
Galntl4	4.46	3.35E-38
Gucy1b3	3.16	8.69E-38
E230008N13 Rik	7.28	2.20E-34
Hey1	3.08	1.74E-31
Heyl	2.96	9.79E-31
Esam	2.60	9.72E-29
Grhl3	6.04	2.75E-27
Cdh13	1.72	1.92E-26
Ces2g	2.94	6.92E-26
Odf3b	4.62	7.97E-26
Pde1b	3.84	1.14E-23
Tnks	1.88	1.24E-23
Serinc3	1.75	2.05E-23
Cspg4	3.13	9.02E-23
Tmx4	2.09	4.79E-22
Siae	3.52	1.25E-21
Pkia	1.67	1.30E-21
Fbxo40	2.39	1.54E-21
Tab3	1.75	2.29E-19
2010300C02 Rik	3.19	2.85E-19
Rabgap1	1.65	5.85E-18
Golga1	1.58	8.66E-17
Plxnb1	3.08	1.07E-16
Rgs9	3.53	2.37E-16
Hint1	0.59	2.61E-16
Kctd1	2.26	2.72E-16
Nppa	0.29	2.95E-16
Myl4	0.61	5.71E-16
4833422C13 Rik	3.95	1.10E-15
Rc3h2	1.44	4.27E-15
Slc25a45	2.32	5.35E-15
Arl4a	0.47	1.45E-14
Vsnl1	3.52	1.73E-14
Btbd11	3.46	2.10E-14
Gm13845	2.75	2.10E-14
Cpt1b	1.91	2.90E-14
Epdr1	1.47	5.53E-14
Ppp6c	1.33	6.92E-14
Stom	2.53	9.08E-14
Dmxl2	0.71	1.09E-13
Prrx2	2.75	1.11E-13
Maged2	2.39	1.15E-13
Slit3	1.91	1.86E-13
Bai2	0.50	2.24E-13
Itm2c	1.73	3.02E-13
Mybpc3	1.79	5.55E-13
Tmem117	0.53	7.01E-13
Prkg1	1.98	7.34E-13
Ddhd2	0.67	8.61E-13
Fam49a	1.84	1.39E-12

Gene ID	Fold Change: iNICD LA Versus Control LA	Adjusted *P* Value
Ramp1	2.72	1.71E-12
Ppargc1b	1.82	1.82E-12
Slc8a2	2.49	1.82E-12
Ccrn4l	0.31	1.91E-12
Col27a1	2.51	2.75E-12
Shank2	3.10	5.04E-12
Perp	2.11	5.99E-12
Adamtsl1	1.97	8.56E-12
Kcnq1	2.08	1.36E-11
Lsm11	2.18	2.35E-11
Pdcl	1.47	2.60E-11
Igsf3	2.04	3.97E-11
Abr	2.01	4.11E-11
Edar	2.59	4.40E-11
Arpc5l	1.52	4.86E-11
Smyd2	2.23	5.06E-11
Dzip1l	1.70	6.11E-11
Ehd3	1.53	8.04E-11
Fat3	2.88	9.24E-11
6330416G13 Rik	0.50	1.99E-10
Atp1b2	2.11	2.03E-10
Lrp1b	2.75	2.53E-10
Gcgr	3.06	2.81E-10
Mtus1	1.69	4.46E-10
Gm12409	3.09	6.27E-10
Acot12	3.09	6.94E-10
Rasd1	2.46	9.05E-10
Aif1l	0.42	1.12E-09
Ak2	1.36	1.15E-09
Zbtb6	1.43	1.87E-09
Fndc5	0.66	2.40E-09
Ndufa8	1.39	2.94E-09
Gcap14	1.25	3.54E-09
Mlana	0.40	3.54E-09
Adora1	2.64	4.20E-09
Pkdcc	0.58	5.64E-09
Mcpt4	2.93	6.37E-09
Lass6	1.89	6.37E-09

Gene ID	Fold Change: iNICD LA Versus Control LA	Adjusted *P* Value
Whrn	0.42	8.69E-09
Serpinb6b	1.56	8.70E-09
Cpox	0.70	1.31E-08
Hcn1	2.87	1.37E-08
Gja6	2.76	1.72E-08
Fam110c	2.55	2.44E-08
Psmb7	1.42	2.81E-08
Srd5a1	1.98	3.29E-08
Tfam	1.29	3.56E-08
Spats2l	1.71	3.56E-08
Doc2g	0.60	4.08E-08
Oaz2-ps	1.39	4.39E-08
Acsf2	1.83	5.07E-08
Itga7	1.54	5.08E-08
Atp8b2	1.37	5.77E-08
Plxnb2	1.48	6.31E-08
Rap1gap2	1.37	7.40E-08
Prdx6	1.24	8.08E-08
Atcay	0.44	8.08E-08
Ctnnbl1	1.49	9.64E-08
Lpgat1	0.69	9.64E-08
Aqp4	2.70	1.39E-07
Chn1	1.40	1.46E-07
Sipa1l1	1.93	1.57E-07
Dhcr24	2.06	1.79E-07
Dgkg	0.43	2.00E-07
Frem3	2.61	2.33E-07
Ip6k3	2.30	2.63E-07
Arvcf	1.41	2.80E-07
Gria1	2.52	3.32E-07
Ncan	2.63	3.41E-07
4933424G05 Rik	2.61	3.48E-07
Kcnip2	0.40	3.71E-07
Gpr173	1.94	4.31E-07
Tlk1	1.25	4.58E-07
Bcam	1.48	5.39E-07
Tmod1	1.26	5.39E-07
Cntfr	0.44	5.97E-07

Gene ID	Fold Change: iNICD LA Versus Control LA	Adjusted *P* Value
Adap1	0.50	6.01E-07
Notch2	1.36	6.89E-07
Ccdc157	1.28	6.97E-07
Palm2	2.19	7.55E-07
Nt5c3	1.66	8.11E-07
Prima1	2.32	8.21E-07
Cyp39a1	1.80	8.21E-07
Ube2d1	1.46	8.58E-07
Scx	0.39	8.66E-07
Ift57	1.38	8.86E-07
Adamts19	0.57	9.13E-07
4930596M17 Rik	2.52	9.19E-07
Haus8	1.60	9.42E-07
Tanc1	1.40	1.00E-06
Dennd1a	1.53	1.43E-06
Pck2	1.55	1.50E-06
Pard3	1.24	1.55E-06
Palmd	1.34	1.63E-06
4632428C04 Rik	2.09	1.71E-06
Tnnt2	0.73	1.79E-06
Mybph	2.50	2.03E-06
AI414108	1.44	2.09E-06
Pitpnm3	2.18	2.19E-06
Cyb5r3	1.34	2.27E-06
Nr4a2	0.44	2.29E-06
Kpna2	0.41	2.59E-06
Vsig2	1.72	3.00E-06
Masp1	1.75	3.22E-06
Idua	0.75	3.66E-06
Ryk	1.29	3.73E-06
Lama2	1.43	3.74E-06
Fkbp11	0.57	3.96E-06
Kbtbd12	0.64	4.25E-06
Slc23a1	2.18	4.25E-06
Agt	1.81	4.25E-06
Sh3bgr	1.79	4.39E-06
4833442J19 Rik	2.18	4.51E-06
Radil	1.62	4.72E-06
Fzd2	0.59	4.72E-06
Agbl2	2.17	5.07E-06
Rhd	0.49	5.10E-06
Vamp5	1.35	5.23E-06
Scai	1.46	5.56E-06
1700055N04 Rik	0.46	5.86E-06
Ssbp2	1.23	6.13E-06
Podn	0.67	6.40E-06
Fntb	0.70	6.75E-06
Rtf1	1.34	6.75E-06
2410017P09 Rik	1.32	6.78E-06
Tbx5	0.64	6.78E-06
Pkig	1.52	7.32E-06
Igf2r	1.39	7.59E-06
Oxct1	0.81	7.59E-06
Fgf18	2.12	7.61E-06
Lgmn	1.35	7.73E-06
Rapsn	1.78	7.92E-06
Pnrc2	1.36	7.93E-06
Pik3r3	1.32	9.02E-06
Gpbar1	2.21	9.34E-06
Gpr180	0.79	1.13E-05
Slc19a2	1.67	1.13E-05
Plod1	0.68	1.21E-05
Nrip2	1.72	1.22E-05
Pitpnc1	1.80	1.33E-05
Sln	0.66	1.33E-05
Obsl1	0.69	1.38E-05
Pak6	0.60	1.41E-05
Bmp2	1.89	1.56E-05
Pla2g16	1.29	1.58E-05
1110012L19 Rik	1.92	1.78E-05
Tmem50b	1.30	1.79E-05
Abra	0.54	2.06E-05

Gene ID	Fold Change: iNICD LA Versus Control LA	Adjusted *P* Value
Lrrc4b	2.05	2.09E-05
Gpsm3	1.54	2.09E-05
Slc25a34	2.13	2.17E-05
Slc25a37	0.75	2.35E-05
Arl5c	1.99	2.45E-05
Cacna2d1	1.32	2.45E-05
Ldhd	0.65	2.69E-05
Gatsl3	1.57	2.87E-05
Efcab2	1.62	2.88E-05
Ppfia4	1.70	3.04E-05
Pip5k1b	0.75	3.13E-05
1110002N22 Rik	1.30	3.43E-05
Myh8	2.13	3.74E-05
Atp1a2	1.84	4.35E-05
B930025P03 Rik	2.20	4.39E-05
Dixdc1	1.85	4.45E-05
Egln1	0.76	4.82E-05
Cxxc4	1.97	5.31E-05
Nrgn	2.21	5.46E-05
Satb1	1.43	5.46E-05
Fxyd6	1.78	5.59E-05
Pcdhga8	1.96	5.64E-05
Ntan1	1.44	5.64E-05
Sik1	0.50	6.12E-05
Dag1	0.84	6.15E-05
Ceacam16	2.22	6.18E-05
Cpa5	2.07	6.18E-05
Cd82	1.59	6.18E-05
Mthfd2	2.19	6.53E-05
Ptpla	0.53	6.73E-05
Carm1	0.74	6.93E-05
Thsd4	1.66	7.26E-05
Smtnl2	1.93	7.48E-05
Tmc7	1.73	7.70E-05
Sh3bgrl2	1.66	7.70E-05
Trib3	1.88	7.77E-05
Ctgf	0.45	8.12E-05

Gene ID	Fold Change: iNICD LA Versus Control LA	Adjusted *P* Value
Baz2a	1.17	8.17E-05
Npy1r	0.53	8.33E-05
Asb11	0.60	9.26E-05
Nt5c1a	1.84	9.28E-05
Mmachc	0.82	9.28E-05
Maged1	0.76	9.38E-05
Ggcx	0.76	1.04E-04
Lrrfip2	1.33	1.06E-04
Hdlbp	0.81	1.06E-04
Plod2	1.21	1.09E-04
Nr4a3	0.47	1.21E-04
Aox1	1.75	1.25E-04
Lrp6	1.27	1.36E-04
Mgmt	0.56	1.39E-04
Laptm4b	1.33	1.47E-04
Ube2h	1.27	1.52E-04
Gatsl2	1.59	1.62E-04
Fam125a	0.78	1.64E-04
Inpp5j	1.44	1.68E-04
Prkab1	0.62	1.68E-04
Gmppa	0.77	1.68E-04
Lgi3	0.49	1.68E-04
4930534B04 Rik	1.62	1.75E-04
Sec22b	0.73	1.81E-04
Abat	1.60	1.87E-04
1110067D22 Rik	1.50	1.87E-04
Ezr	1.26	1.98E-04
Fam81a	2.12	2.03E-04
Amotl2	0.70	2.03E-04
Mest	2.11	2.05E-04
Sh3tc1	0.66	2.07E-04
Il18bp	1.68	2.10E-04
Fbxw8	0.79	2.12E-04
Frmd5	0.52	2.15E-04
Sipa1l2	1.44	2.17E-04
Cyr61	0.48	2.24E-04
Senp7	1.51	2.26E-04

Gene ID	Fold Change: iNICD LA Versus Control LA	Adjusted *P* Value
Hars2	1.27	2.53E-04
Akt2	0.78	2.65E-04
Tead1	1.34	2.69E-04
Aspdh	2.09	2.70E-04
Chd3	1.33	2.70E-04
Mapk6	0.75	2.70E-04
Egln3	0.57	2.70E-04
Col4a5	1.29	2.76E-04
Mr1	1.26	2.80E-04
Rerg	1.81	2.81E-04
Ank3	1.26	2.94E-04
Actg1	0.48	2.99E-04
Csdc2	1.55	3.17E-04
Dok4	1.57	3.23E-04
2610028H24 Rik	0.48	3.23E-04
Pter	1.44	3.24E-04
Rnf187	0.84	3.25E-04
Acad12	0.60	3.29E-04
Nomo1	0.86	3.29E-04
Aldob	0.49	3.29E-04
Zfp385b	1.69	3.29E-04
Gpc1	0.64	3.29E-04
Ino80c	1.23	3.66E-04
Syde2	0.81	3.66E-04
Tbc1d16	0.72	3.66E-04
Tuba1c	0.50	3.66E-04
Ndufa4	0.79	3.70E-04
Tslp	1.81	3.71E-04
Ntsr2	0.53	4.07E-04
Ces3b	2.00	4.23E-04
Inha	0.71	4.24E-04
Trim72	0.72	4.31E-04
Ghr	1.43	4.32E-04
Gss	1.32	4.32E-04
Chst11	0.65	4.40E-04
1810046J19 Rik	0.78	4.56E-04
1500004A13 Rik	0.54	4.56E-04
Acy1	0.73	4.62E-04
Lbh	1.75	4.67E-04
Setd7	1.18	4.75E-04
Dhrs13	1.53	5.31E-04
Nudt16	1.53	5.32E-04
Padi2	0.58	5.32E-04
Gpx3	0.71	5.36E-04
Rcsd1	1.56	5.39E-04
Htra1	1.23	5.39E-04
Zzef1	1.26	5.44E-04
Adcy6	1.33	5.49E-04
BC021891	0.51	5.73E-04
Tecta	2.04	5.74E-04
Spon1	1.57	5.84E-04
D19Bwg135 7e	1.75	5.88E-04
Gipr	2.03	5.89E-04
Gramd4	1.34	5.89E-04
Trim37	1.30	6.12E-04
Tmem100	0.52	6.15E-04
Jup	1.28	6.44E-04
Epb4.1l1	0.71	6.48E-04
Zbtb26	1.30	6.48E-04
Pygl	1.75	6.60E-04
Lpar3	2.02	6.77E-04
Cited4	0.59	7.47E-04
BC023829	1.22	7.63E-04
Itga2b	0.64	7.78E-04
Aldh5a1	1.33	7.90E-04
Mt3	0.62	8.03E-04
Egr2	0.50	8.06E-04
Dusp5	0.53	8.09E-04
Ube2e3	1.42	8.31E-04
Tap2	1.36	8.49E-04
Fbxo9	1.46	8.58E-04
Cuedc1	1.29	8.63E-04
Scube2	1.88	8.88E-04

Gene ID	Fold Change: iNICD LA Versus Control LA	Adjusted *P* Value
Plin4	0.59	8.88E-04
Dtnbp1	1.27	9.45E-04
Spata24	1.75	9.61E-04
Cnga3	2.00	9.64E-04
Mrps6	0.74	9.87E-04
Eno3	0.71	9.87E-04
Crlf3	0.81	1.01E-03
Ldha	0.79	1.01E-03
Slc22a7	0.51	1.01E-03
Ston2	1.58	1.05E-03
Pzp	0.56	1.05E-03
Grm1	0.59	1.07E-03
Gm9899	1.54	1.09E-03
Adrb1	1.53	1.09E-03
Mrvi1	1.87	1.09E-03
Gm1976	1.57	1.09E-03
Gnai1	1.44	1.09E-03
Adamts1	0.54	1.12E-03
Creb3l2	0.71	1.14E-03
Pxmp2	0.66	1.17E-03
Id3	0.75	1.18E-03
Abi2	1.21	1.20E-03
Gas6	1.24	1.21E-03
Mpi	1.28	1.21E-03
Tpmt	1.48	1.22E-03
Fyco1	1.44	1.26E-03
Morf4l1	1.76	1.27E-03
Myog	1.90	1.27E-03
Iah1	1.60	1.27E-03
L3mbtl3	1.41	1.30E-03
Tspo	1.23	1.31E-03
Mtus2	0.71	1.31E-03
Ppp1r3g	1.96	1.39E-03
Sytl2	1.48	1.40E-03
Guk1	0.80	1.40E-03
Ctsh	1.30	1.44E-03
Cdc42se2	1.16	1.53E-03
Arfgap1	0.73	1.60E-03

Gene ID	Fold Change: iNICD LA Versus Control LA	Adjusted *P* Value
Slc17a9	0.59	1.60E-03
Gchfr	0.56	1.62E-03
Trak2	1.50	1.67E-03
Klhl30	1.49	1.67E-03
Fut10	1.41	1.67E-03
D730040F13 Rik	1.32	1.67E-03
Npepl1	0.84	1.67E-03
Pbld2	0.63	1.67E-03
Flot2	1.26	1.72E-03
Scn10a	0.52	1.72E-03
Myl7	0.70	1.79E-03
Crip3	0.53	1.79E-03
Acsl6	1.60	1.84E-03
Gfod1	0.77	1.88E-03
Atp6ap1l	1.79	1.96E-03
Otud1	0.53	2.02E-03
Shisa2	0.60	2.03E-03
Whamm	1.27	2.06E-03
Stbd1	1.83	2.07E-03
Lhfpl4	1.64	2.09E-03
Nfatc2	1.52	2.09E-03
Tmprss13	1.90	2.10E-03
Gpr22	1.92	2.14E-03
Sord	0.68	2.14E-03
Pik3ca	1.15	2.16E-03
Rxfp1	0.53	2.20E-03
2510009E07 Rik	1.32	2.23E-03
Hspb3	1.40	2.24E-03
Lypd6b	1.91	2.25E-03
Apip	0.82	2.29E-03
Tnni2	1.92	2.31E-03
Tnxb	1.35	2.34E-03
Epb4.1l3	1.52	2.36E-03
Kcnd3	1.25	2.36E-03
Wtip	0.75	2.36E-03
Ctsd	1.22	2.36E-03
Glrx	1.26	2.41E-03

Gene ID	Fold Change: iNICD LA Versus Control LA	Adjusted *P* Value
Plbd2	0.86	2.41E-03
Tead3	0.72	2.41E-03
Ropn1	1.84	2.44E-03
Bcl2	1.53	2.44E-03
Yif1b	0.75	2.44E-03
Tmco4	0.79	2.45E-03
Rhobtb1	0.64	2.52E-03
Slc15a4	0.77	2.53E-03
Id1	0.59	2.53E-03
2310015B20 Rik	1.82	2.53E-03
Ncald	1.72	2.53E-03
Plekha2	1.25	2.56E-03
Tmem82	1.81	2.61E-03
Ppp1r13b	0.80	2.64E-03
Gpr17	0.57	2.64E-03
Gramd2	1.90	2.65E-03
Art3	1.35	2.66E-03
Iqgap3	1.73	2.72E-03
Tex9	1.57	2.72E-03
Zfhx2	0.75	2.74E-03
Fos	0.55	2.81E-03
Thap4	0.84	2.86E-03
Limch1	1.28	2.87E-03
Pkm2	1.25	2.87E-03
Gng2	0.70	2.87E-03
Cnksr1	0.53	2.87E-03
Tmem51	1.38	2.90E-03
1110034G24 Rik	0.67	2.91E-03
Enho	0.64	3.06E-03
Tacc2	1.34	3.18E-03
Slc25a25	0.65	3.21E-03
Pqlc1	1.29	3.22E-03
Apbb1	0.74	3.31E-03
Ccrl2	1.63	3.32E-03
Zfp105	1.49	3.35E-03
1700021K19 Rik	1.31	3.35E-03

Gene ID	Fold Change: iNICD LA Versus Control LA	Adjusted *P* Value
Pgm5	1.29	3.35E-03
Sepw1	1.19	3.43E-03
Fhl2	1.77	3.47E-03
Lgi2	0.55	3.47E-03
Gm5779	1.82	3.64E-03
St8sia5	1.82	3.65E-03
Hn1	0.83	3.71E-03
Egr1	0.54	3.71E-03
Chac1	1.67	3.77E-03
2810011L19 Rik	1.79	3.78E-03
Slc7a7	1.67	3.78E-03
Reln	1.42	3.85E-03
Rhoq	1.25	3.87E-03
Sbk1	1.72	3.88E-03
Ddr1	1.24	3.94E-03
Herc3	1.32	3.99E-03
Nle1	0.69	4.01E-03
D630045J12 Rik	0.62	4.08E-03
Polg	0.81	4.15E-03
Npm3	0.66	4.19E-03
Grsf1	1.15	4.27E-03
Ankrd6	1.59	4.36E-03
Zfp949	1.37	4.39E-03
Olfml2a	1.65	4.40E-03
5730403B10 Rik	1.21	4.42E-03
Tymp	1.77	4.45E-03
Arhgap31	0.81	4.47E-03
Smad7	0.62	4.47E-03
Mterfd3	1.58	4.63E-03
Slc12a6	1.34	4.71E-03
Slc8a3	1.84	4.82E-03
Nkain1	0.55	4.82E-03
Cenpf	1.83	4.85E-03
Sertad1	0.66	4.86E-03
Optn	0.77	4.91E-03
Mdk	0.64	4.91E-03

Gene ID	Fold Change: iNICD LA Versus Control LA	Adjusted *P* Value
Acot7	0.75	4.91E-03
Kank1	0.88	5.01E-03
Fam132b	1.76	5.07E-03
6430598A04 Rik	0.72	5.17E-03
4833424O15 Rik	1.84	5.21E-03
Cyp2j6	1.39	5.24E-03
Dhrs3	1.48	5.29E-03
Peli2	1.42	5.30E-03
Arhgap42	1.25	5.34E-03
Acss1	0.75	5.35E-03
Zbtb42	0.71	5.35E-03
Mfn2	0.78	5.39E-03
Chmp4c	1.36	5.40E-03
Dynlt3	1.18	5.40E-03
Neil3	1.79	5.47E-03
Ttyh3	1.28	5.49E-03
Rbm14	0.76	5.50E-03
Tmc6	0.80	5.51E-03
Lpcat1	1.36	5.59E-03
Kbtbd13	0.54	5.70E-03
1700025G04 Rik	1.43	5.82E-03
Ptchd2	1.77	5.85E-03
Pvrl2	1.36	5.96E-03
Ccdc104	1.26	5.98E-03
Tmem56	1.73	6.05E-03
Fastkd2	0.81	6.05E-03
Gm14057	1.45	6.13E-03
1520402A15 Rik	1.43	6.19E-03
Fry	1.37	6.19E-03
Gpr157	0.61	6.19E-03
Ppp1r3d	1.47	6.34E-03
Drd1a	0.55	6.34E-03
Kif6	0.57	6.39E-03
Kazald1	1.65	6.45E-03
Neto2	1.67	6.52E-03
Srf	0.63	6.55E-03

Gene ID	Fold Change: iNICD LA Versus Control LA	Adjusted *P* Value
Chkb	1.58	6.63E-03
Enox1	1.73	6.65E-03
Amn1	1.53	6.69E-03
Lingo3	0.55	6.83E-03
Ranbp17	1.67	6.99E-03
Dsg2	1.37	7.11E-03
Acvr1	0.85	7.17E-03
Pgam2	0.79	7.21E-03
2310050B05 Rik	0.69	7.25E-03
Mybphl	0.77	7.40E-03
Mapk12	1.34	7.40E-03
Mgat5	1.54	7.41E-03
Vldlr	1.55	7.54E-03
Csnk1g1	1.25	7.57E-03
Krt18	0.62	7.63E-03
Usp32	1.21	7.74E-03
Ptafr	1.59	7.82E-03
H47	0.81	7.82E-03
Sema3c	0.76	7.82E-03
Obfc2a	0.68	7.84E-03
Mybpc2	0.59	7.89E-03
D7Ertd715e	1.69	7.95E-03
Rgs7	1.45	7.95E-03
Tspan7	1.20	7.95E-03
Chtf8	0.85	7.95E-03
Cd164	0.91	8.02E-03
Bdh1	0.56	8.02E-03
6330403A02 Rik	1.79	8.05E-03
Ddt	1.28	8.12E-03
Zfpm2	1.29	8.17E-03
Acsl3	1.39	8.20E-03
D5Ertd579e	1.19	8.21E-03
Irs2	0.57	8.21E-03
Pomp	1.13	8.24E-03
Maz	0.87	8.29E-03
Wfs1	1.47	8.40E-03
Gm13807	1.68	8.40E-03

Gene ID	Fold Change: iNICD LA Versus Control LA	Adjusted *P* Value
Aldh3a2	1.31	8.41E-03
Hamp	0.58	8.42E-03
Rgma	1.38	8.46E-03
Arhgap12	1.22	8.46E-03
BC004004	0.83	8.56E-03
Aig1	1.36	8.74E-03
Clec4g	1.79	8.75E-03
Paqr9	0.75	8.96E-03
Icam4	0.56	9.11E-03
Pdk1	0.82	9.11E-03
Fam131c	0.60	9.18E-03
Ntf3	1.77	9.24E-03
BC031353	1.62	9.35E-03
BC022687	0.67	9.35E-03
Pkp4	1.34	9.40E-03
Slc25a4	0.78	9.40E-03
Kif7	0.74	9.40E-03
Tspan3	1.29	9.41E-03
Gsto1	0.72	9.43E-03
Ric3	1.70	9.81E-03
Cited1	0.56	9.84E-03
Egr3	0.57	9.89E-03
Rnd1	1.72	9.99E-03
Lca5	1.44	1.00E-02
Slc38a10	0.82	1.00E-02
Smc1a	1.20	1.00E-02
Sec14l2	1.61	1.01E-02
Slc2a10	0.81	1.01E-02
Nppb	0.60	1.01E-02
Gpr4	0.72	1.02E-02
Adar	1.30	1.02E-02
Gm12295	1.77	1.02E-02
Osbpl10	0.64	1.03E-02
Jag2	0.73	1.03E-02
A330023F24 Rik	1.45	1.03E-02
AI507597	1.73	1.04E-02
Mapre2	0.81	1.04E-02
Syt7	1.44	1.05E-02

Gene ID	Fold Change: iNICD LA Versus Control LA	Adjusted *P* Value
Thsd7a	1.42	1.05E-02
Mast4	1.39	1.05E-02
Usp10	0.81	1.05E-02
Slc38a2	0.59	1.06E-02
Prps2	1.42	1.06E-02
Sra1	0.77	1.06E-02
Tmem108	0.70	1.08E-02
Plin5	0.59	1.08E-02
Ier2	0.59	1.08E-02
Herc1	1.24	1.09E-02
Vegfa	0.71	1.10E-02
Kcp	1.30	1.10E-02
P2rx4	1.19	1.10E-02
Pdk3	1.25	1.13E-02
Cpsf1	0.89	1.20E-02
S100a3	1.74	1.21E-02
Gsta1	1.71	1.21E-02
Asap3	1.29	1.22E-02
L3mbtl1	1.74	1.24E-02
Jag1	1.32	1.25E-02
Gm5424	0.66	1.27E-02
Chml	1.39	1.27E-02
Pde6d	1.27	1.30E-02
Micall1	0.72	1.32E-02
Dgat1	0.84	1.32E-02
Asns	1.73	1.32E-02
Porcn	1.35	1.33E-02
Dtx3	1.12	1.36E-02
Pdxdc1	0.86	1.37E-02
Vdac3	0.85	1.37E-02
Tbc1d10b	0.81	1.37E-02
Adamts15	1.41	1.38E-02
Setdb1	0.87	1.38E-02
Nid2	1.24	1.39E-02
Zrsr1	1.31	1.40E-02
Etv5	0.69	1.42E-02
Ptpro	1.56	1.43E-02
Rin2	1.37	1.44E-02

Gene ID	Fold Change: iNICD LA Versus Control LA	Adjusted *P* Value
Prkcz	0.67	1.44E-02
Hps4	0.84	1.44E-02
Gm3646	1.60	1.45E-02
Gramd1b	1.65	1.45E-02
Lats2	0.85	1.46E-02
Hydin	1.74	1.46E-02
Ppp1r3b	1.27	1.46E-02
Ppp1r14c	1.44	1.47E-02
Ano4	1.38	1.47E-02
Tuba1b	0.74	1.48E-02
Dusp18	0.68	1.48E-02
Tceb3	0.88	1.49E-02
Zfp334	1.30	1.50E-02
Cdk5r1	0.64	1.50E-02
Aldh3b2	0.59	1.51E-02
Dagla	1.39	1.51E-02
Mbd5	1.22	1.51E-02
Pacsin2	1.21	1.51E-02
Golt1b	0.76	1.51E-02
AW555464	0.70	1.51E-02
Gm1574	1.64	1.52E-02
Sept4	1.46	1.53E-02
Rccd1	0.77	1.53E-02
Med9	1.21	1.56E-02
2810474O19 Rik	1.61	1.57E-02
Gm14446	1.33	1.61E-02
Apobec2	1.37	1.62E-02
Rplp0	0.86	1.63E-02
Dgkz	1.30	1.63E-02
Dctn6	1.23	1.63E-02
Itgb1bp3	0.64	1.64E-02
2310002L09 Rik	0.62	1.64E-02
Gosr2	0.83	1.64E-02
Gabarap	0.90	1.65E-02
Gm11627	0.59	1.68E-02
Clcn3	1.15	1.68E-02
Nr1h3	1.19	1.68E-02
Slc1a2	1.67	1.68E-02
Nphp1	1.21	1.69E-02
Fosl2	0.64	1.69E-02
Fam59b	0.59	1.69E-02
Ncdn	0.86	1.70E-02
Wnk1	1.14	1.71E-02
Stk17b	1.29	1.74E-02
Map6d1	1.71	1.74E-02
Dexi	0.80	1.75E-02
Gadd45g	0.59	1.75E-02
Cdca3	1.68	1.76E-02
Arhgef9	1.37	1.76E-02
Col20a1	1.30	1.76E-02
Prkci	0.86	1.76E-02
St8sia2	0.59	1.76E-02
Xcr1	1.67	1.76E-02
A730036I17 Rik	1.72	1.78E-02
Cxcl5	0.58	1.78E-02
Gypc	1.35	1.78E-02
Tuba4a	0.74	1.78E-02
Tcof1	0.88	1.78E-02
Tbc1d1	0.82	1.80E-02
Slc45a4	1.28	1.81E-02
Pcdhga3	1.37	1.83E-02
Btg2	0.58	1.83E-02
Rev3l	1.15	1.87E-02
Ckb	0.77	1.87E-02
4732415M23 Rik	0.72	1.88E-02
Ift74	1.38	1.90E-02
Kcnn2	1.55	1.91E-02
Acss2	1.17	1.94E-02
Mmd	0.79	1.94E-02
Irf4	1.46	1.95E-02
Fosb	0.61	1.99E-02
2210020M01 Rik	1.45	2.00E-02
Vrk1	1.22	2.00E-02

Gene ID	Fold Change: iNICD LA Versus Control LA	Adjusted *P* Value
Ank	1.15	2.02E-02
Cib2	1.43	2.02E-02
Rgs16	0.63	2.03E-02
Kntc1	1.66	2.07E-02
Ggt7	1.36	2.07E-02
Prkg2	1.68	2.07E-02
Pou2af1	0.59	2.07E-02
Camk2g	1.19	2.07E-02
Fam108c	0.86	2.11E-02
Osgepl1	1.34	2.12E-02
Zfp1	1.24	2.13E-02
Sdk2	0.61	2.13E-02
Nkd1	0.68	2.14E-02
Slc39a8	0.69	2.14E-02
Epha4	0.71	2.16E-02
Ankrd13a	0.69	2.16E-02
Tmcc1	1.16	2.17E-02
Dlgap5	1.66	2.21E-02
Tjap1	0.78	2.21E-02
Cbx5	1.15	2.22E-02
Pitrm1	0.89	2.22E-02
Tsc1	0.86	2.22E-02
Agrn	1.30	2.24E-02
Mlxip	1.27	2.24E-02
Tspyl4	1.21	2.25E-02
Bcap31	0.90	2.25E-02
Scn4b	1.70	2.26E-02
Lzic	0.86	2.26E-02
Rab2a	0.88	2.28E-02
Gm9054	0.60	2.31E-02
Gpr172b	0.81	2.31E-02
Homer2	1.33	2.33E-02
Nr4a1	0.59	2.33E-02
Kif20a	1.67	2.34E-02
Tcf4	1.21	2.35E-02
Ralgps2	1.37	2.35E-02
Adrbk2	1.38	2.36E-02
Aph1b	1.34	2.36E-02

Gene ID	Fold Change: iNICD LA Versus Control LA	Adjusted *P* Value
Arhgap36	1.69	2.37E-02
Mmp27	1.67	2.37E-02
Lrtm1	0.59	2.42E-02
Nudt14	0.74	2.45E-02
Mif4gd	0.83	2.45E-02
Lynx1	1.16	2.46E-02
Gm10635	1.69	2.47E-02
Trp53i13	0.79	2.48E-02
Ints4	0.83	2.52E-02
Ckap2l	1.66	2.62E-02
Ankle1	1.64	2.62E-02
Ppp1r13l	0.75	2.62E-02
Beta-s	0.60	2.62E-02
Galk1	0.82	2.62E-02
Smpd1	0.81	2.63E-02
E030010A14 Rik	1.46	2.66E-02
Eny2	1.19	2.66E-02
Dap3	0.83	2.66E-02
Atg16l2	0.81	2.66E-02
Tpbg	0.66	2.66E-02
Trim28	0.88	2.67E-02
Pdgfb	0.69	2.67E-02
Gnb2l1	0.89	2.70E-02
Zfp238	1.17	2.71E-02
2700029M09 Rik	1.21	2.73E-02
Lin37	1.26	2.73E-02
Aldh16a1	0.69	2.74E-02
Ptprf	0.75	2.75E-02
Gm10818	1.67	2.75E-02
Lims2	0.61	2.76E-02
BC060267	1.67	2.77E-02
Mpp1	1.20	2.79E-02
Setd8	0.71	2.79E-02
Ppyr1	1.68	2.79E-02
Lrrk2	1.27	2.80E-02
C230081A13 Rik	1.32	2.80E-02

Gene ID	Fold Change: iNICD LA Versus Control LA	Adjusted *P* Value
Med19	0.86	2.81E-02
Atp6v0a2	0.84	2.81E-02
Gstm5	1.16	2.83E-02
1700120C14 Rik	1.44	2.86E-02
Rasgef1b	0.64	2.87E-02
Mgll	0.74	2.90E-02
Gch1	0.65	2.90E-02
Nek2	1.61	2.91E-02
Tnk2	1.33	2.91E-02
Rorc	0.77	2.91E-02
Csrnp1	0.62	2.92E-02
Klhl23	1.24	2.93E-02
Hmmr	1.57	2.93E-02
Got2	1.21	2.93E-02
Pgpep1l	1.67	2.93E-02
Copb2	0.87	2.93E-02
Srsf3	0.81	2.93E-02
F3	0.70	2.93E-02
Sgk1	0.64	2.93E-02
Esrp2	0.60	2.93E-02
Plscr1	1.32	2.94E-02
Nav2	1.22	2.96E-02
Atp6v1g2	1.29	3.01E-02
Nudt17	0.75	3.01E-02
Gt(ROSA)26 Sor	0.81	3.02E-02
Atp8a2	1.34	3.02E-02
Tcp11	0.60	3.03E-02
Asb12	1.40	3.05E-02
Slc35f5	0.81	3.05E-02
Bub1	1.62	3.06E-02
Rbm19	0.80	3.06E-02
Arl4c	1.35	3.07E-02
A730020M07 Rik	0.64	3.08E-02
Ankrd29	0.76	3.08E-02
Tmcc2	1.17	3.10E-02
Txndc5	0.82	3.11E-02

Gene ID	Fold Change: iNICD LA Versus Control LA	Adjusted *P* Value
Tro	1.33	3.12E-02
Mbp	0.82	3.12E-02
Col4a4	1.43	3.12E-02
4933426M11 Rik	1.11	3.12E-02
Samd1	0.79	3.12E-02
Lrg1	0.61	3.12E-02
Wbscr27	1.49	3.13E-02
Ppa1	0.72	3.13E-02
Gabra3	1.58	3.14E-02
Melk	1.62	3.15E-02
Trim7	0.73	3.17E-02
Mecp2	1.25	3.18E-02
Cplx2	1.43	3.18E-02
Ppa2	1.24	3.19E-02
Phyhd1	0.72	3.22E-02
Dkk3	0.68	3.24E-02
Hddc2	1.49	3.26E-02
Lass4	1.46	3.26E-02
Fam123b	1.43	3.26E-02
A930004D18 Rik	1.42	3.26E-02
Ifih1	1.39	3.26E-02
Kcnh2	1.38	3.26E-02
Tbx20	1.26	3.26E-02
Yipf7	0.80	3.26E-02
Anln	1.66	3.28E-02
B230208H17 Rik	0.86	3.28E-02
Adipor1	0.90	3.31E-02
Sall2	1.32	3.37E-02
Nudt4	0.71	3.38E-02
Sec31a	0.79	3.38E-02
Nr0b2	1.40	3.38E-02
5430416O09 Rik	1.41	3.40E-02
Gm13154	1.60	3.41E-02
Vdr	0.61	3.41E-02
Atl2	1.23	3.41E-02
Ghitm	1.16	3.43E-02

Gene ID	Fold Change: iNICD LA Versus Control LA	Adjusted *P* Value
P4ha1	0.74	3.43E-02
Tbkbp1	0.79	3.47E-02
Cep55	1.62	3.48E-02
Dock3	1.65	3.51E-02
Stk32b	1.59	3.51E-02
0610037P05 Rik	1.14	3.51E-02
Cdv3	0.75	3.51E-02
Gcat	0.72	3.51E-02
Asb14	0.62	3.51E-02
Fkbp9	1.11	3.52E-02
A930005H10 Rik	1.47	3.56E-02
Fam149a	1.31	3.58E-02
Dtnb	0.76	3.59E-02
Nos1	0.61	3.59E-02
Rps19	0.76	3.60E-02
Dap	1.28	3.63E-02
Napg	1.16	3.63E-02
Zfp36	0.61	3.63E-02
Tgfa	0.76	3.63E-02
Sardh	0.76	3.64E-02
Wdr13	1.26	3.65E-02
Dync1li2	1.10	3.65E-02
Sar1a	0.83	3.65E-02
Polr2e	0.90	3.66E-02
Tekt1	1.65	3.67E-02
Hdc	0.65	3.68E-02
Akap12	0.81	3.69E-02
Lrat	1.65	3.73E-02
Adamts14	1.49	3.75E-02
Psip1	1.26	3.77E-02
Il15ra	1.20	3.77E-02
Csnk1e	1.22	3.77E-02
Orai3	0.80	3.82E-02
Zfp295	0.76	3.83E-02
Fam13b	1.18	3.83E-02
Aebp1	0.85	3.83E-02
Rheb	0.79	3.90E-02

Gene ID	Fold Change: iNICD LA Versus Control LA	Adjusted *P* Value
Scn2b	0.64	3.90E-02
Slc22a4	1.29	3.92E-02
Ankrd50	1.31	3.95E-02
Pnpla3	1.63	3.95E-02
Ptgs1	1.30	3.95E-02
Ddn	0.67	3.97E-02
Cxcl1	0.61	3.97E-02
Cd33	1.42	3.97E-02
Atp1a1	0.74	3.98E-02
Slc12a5	0.66	4.03E-02
Cobra1	1.12	4.05E-02
Rassf3	1.18	4.05E-02
Trim9	1.62	4.05E-02
Ankrd13b	0.84	4.05E-02
Csrp2	0.74	4.05E-02
Pif1	1.56	4.09E-02
9030418K01 Rik	1.32	4.10E-02
Mrpl36	1.20	4.11E-02
Zfp641	1.29	4.13E-02
Coro7	1.18	4.14E-02
Adi1	0.73	4.16E-02
Rasip1	0.81	4.17E-02
Tmtc1	1.27	4.18E-02
Dcbld2	1.14	4.18E-02
Ccng1	0.83	4.19E-02
Hapln4	0.63	4.19E-02
4833419F23 Rik	1.51	4.25E-02
Klhl33	1.61	4.25E-02
Arhgap11a	1.52	4.27E-02
Pitx2	1.29	4.32E-02
Aldh1a3	0.63	4.32E-02
Blvra	1.16	4.34E-02
Serf2	0.89	4.34E-02
Ermp1	0.81	4.39E-02
Camkk2	0.78	4.41E-02
5031414D18 Rik	1.63	4.43E-02

Gene ID	Fold Change: iNICD LA Versus Control LA	Adjusted *P* Value
Tlcd1	1.28	4.43E-02
Fam134b	1.32	4.45E-02
Mogs	0.77	4.47E-02
Zfpl1	0.83	4.48E-02
Arv1	0.77	4.52E-02
Igsf1	1.47	4.53E-02
Apod	1.40	4.56E-02
Josd1	0.83	4.57E-02
Abca8b	1.34	4.59E-02
Elp3	1.16	4.59E-02
Ubr3	1.12	4.60E-02
Prpf3	0.82	4.61E-02
Ifitm2	0.79	4.61E-02
Pdcd11	0.85	4.67E-02
Lrfn4	0.75	4.67E-02
Epha2	0.69	4.67E-02
Etnk2	0.65	4.69E-02
Spop	1.17	4.70E-02
Alas2	0.62	4.76E-02

Gene ID	Fold Change: iNICD LA Versus Control LA	Adjusted *P* Value
Hsf1	0.83	4.77E-02
Cadm1	1.43	4.79E-02
Dpy19l4	1.34	4.83E-02
A930003A15 Rik	1.56	4.86E-02
Nr6a1	1.38	4.86E-02
Myocd	1.26	4.86E-02
Acbd5	1.20	4.86E-02
Tnfaip8	1.29	4.88E-02
Ak3	1.15	4.88E-02
Gm11128	1.50	4.91E-02
Pdxk	0.84	4.91E-02
Acy3	0.68	4.91E-02
Tubb5	0.71	4.93E-02
Lman1	0.88	4.93E-02
Hn1l	0.75	4.94E-02
Cdk14	1.28	4.96E-02
Tmem198b	1.26	4.96E-02
Ier5	0.65	4.96E-02

All differentially expressed genes from the RNA-sequencing data set are shown as fold changes of iNICD LA (n = 6) relative to iNICD controls (n = 6) along with adjusted *P* value. False discovery rate (FDR) adjusted *P* values < 0.05 are considered statistically significant.

Table S12. Top 25 Ingenuity Pathway Analysis generated diseases or functions annotations for murine iNICD LA compared to controls

Categories	Diseases or Functions Annotation	*P* Value	# Molecules	Molecules
Liver Hyperplasia/ Hyperproliferation	Liver Tumor	1.74E-08	341	*ABCA8, ACOT7, ACSL6, ACSS2, ACTG1, ACVR1, ACY1, ADAMTS1, ADAMTS19, ADAMTSL1, ADAR, ADIPOR1, AEBP1, AGRN, AGT, AIG1, AK2, AKT2, ALDH1A3, ALDOB, AMN1, ANK3, ANLN, ANO4, AOX1, APIP, ARFGAP1, ARHGAP12, ARHGEF9, ARV1, ARVCF, ASAP3, ASB11, ASB14, ATG16L2, ATL2, ATP1A1, ATP1A2, ATP8A2, BAZ2A, BCL2, BDH1, BLVRA, BTBD11, BUB1, C20orf196, C3orf70, CACNA2D1, CAMK2G, CARD11, CARM1, CCNG1, CCRL2, CD82, CDCA3, CDH13, CDK14, CDK5R1, CEACAM16, CENPF, CHD3, CHMP4C, CLASP1, CLCN3, CLEC4G, CNTFR, COL20A1, COL27A1, COL4A4, COL4A5, CPT1B, CREB3L2, CRIP3, CSNK1E, CTSD, CXCL2, CYP39A1, CYR61, DAG1, DAGLA, DAP3, DCBLD2, DENND1A, DGKG, DHCR24, DMXL2, DPY19L4, DTNB, DTNBP1, EDAR, EGLN1, EHD3, ENOX1, EPHA2, EPHA4, ERMP1, ETV5, F3, FAM131C, FAM13B, FAT3, FBXO9, FBXW8, FOS, FRY, FUT10, GAS6, GCH1, GHITM, GHR, GIPR, GNAI1, GOLT1B, GPR17, GPR4, GRAMD1B, GRHL3, GRIA1, GSTA5, GSTO1, GUCY1B3, HAUS8, HCN1, HDLBP, HERC1, HMMR, HSF1, HYDIN, ICAM4, ID1, ID3, IER5, IFIH1, IFT57, IFT74, IGF2R, IGSF1, IGSF3, IL15RA, INHA, INTS4, IQGAP3, IRS2, ITGA2B, ITGA7, JAG1, JAG2, JUP, KANK1, KBTBD12, KCNH2, KCNIP2, KIAA1211L, KIAA1549, KLHL23/PHOSPHO2-KLHL23, KLHL33, KNTC1, L3MBTL3, LAMA2, LAPTM4B, LATS2, LCA5, LDHA, LGMN, LIMCH1, LMAN1, LPCAT1, LRG1, LRP1B, LRP6, LRRC4B, LRRC8E, LRRK2, LSM11, LYPD6B, MAPK6, MAST4, MBD5, MBP, MDK, MED19, MFN2, MGAT5, MGLL, MGMT, MLXIP, MMACHC, MMP27, MPI, MPP2, MRVI1, MTUS2, MYBPC3, MYBPH, MYH8, MYL4, MYOCD, MYOM3, NAV2, NCALD, NCAN, NEIL3, NFATC2, NID2, NKD1, NOS1, NOTCH1, NPY1R, NR4A1, NR4A3, NTF3, ORAI3, OSGEPL1, OXCT1, PADI2, PAQR9, PARD3, PCDHGA3, PCK2, PDCD11, PDE1B, PDK3, PDXK, PGM5, PHYHD1, PIF1, PIK3CA, PIK3R3, PITPNM3, PITRM1, PKP4, PLOD1, PLXNB1, PLXNB2, PNPLA3, POLG, PPA2, PPARGC1B, PRKG2, PRPF3, PSIP1, PTER, PTGS1, PTPRF, PYGL, RABGAP1, RADIL, RALGPS2, RANBP17, RAP1GAP2, RAPSN, RASGEF1B, RASIP1, RBM14, RC3H2, RCSD1, RELN, REV3L, RGS7, RGS9, RHOBTB1, RIC3, RIN2, RORC, SALL2, SARDH, SATB1, SCN10A, SCUBE2, SDK2, SEC31A, SEMA3C, SERF2, SETDB1, SGK1, SHISA2, SIPA1L2, SLC12A5, SLC12A6, SLC17A9, SLC25A25, SLC35F5, SLC38A2, SLC39A8, SLC45A4, SLC8A2, SLC8A3, SLIT3, SMYD2, SPATS2L, SPON1, SRA1, STK17B, STK32B, STON2, SYDE2, SYTL2, TACC2, TANC1, TBC1D1, TBC1D16, TBKBP1, TBX20, TBX5, TCOF1, TECTA, TGFA, THAP4, TJAP1, TLCD1, TLK1, TMCC2, TMEM108, TMEM50B, TMEM82, TMPRSS13, TMTC1, TNK2, TNNT2, TNXB, TPBG, TRIM28, TRIM9, TRO, TSC1, TSPAN7, TSPYL4, TTYH3, TUBA1C, TUBA4A, TYMP, UBR3, VDR, VEGFA, VLDLR, WNK1, ZBTB26, ZFHX2, ZFPL1, ZFPM2, ZNF385B*
Liver Hyperplasia/ Hyperproliferation	Liver Carcinoma	2.31E-08	323	*ABCA8, ACOT7, ACSL6, ACSS2, ACTG1, ACVR1, ACY1, ADAMTS1, ADAMTS19, ADAMTSL1, ADAR, ADIPOR1, AEBP1, AGRN, AGT, AIG1, AK2, ALDH1A3, ALDOB, AMN1, ANK3, ANLN, ANO4, AOX1, APIP, ARFGAP1, ARHGAP12, ARHGEF9, ARV1, ARVCF, ASAP3, ASB11, ASB14, ATG16L2,*

Categories	Diseases or Functions Annotation	*P* Value	# Molecules	Molecules
				ATL2, ATP1A1, ATP1A2, ATP8A2, BAZ2A, BCL2, BDH1, BLVRA, BTBD11, BUB1, C20orf196, C3orf70, CACNA2D1, CAMK2G, CARD11, CARM1, CCNG1, CCRL2, CD82, CDCA3, CDH13, CDK14, CDK5R1, CEACAM16, CENPF, CHD3, CHMP4C, CLASP1, CLCN3, CLEC4G, CNTFR, COL20A1, COL27A1, COL4A4, COL4A5, CPT1B, CREB3L2, CRIP3, CSNK1E, CTSD, CXCL2, CYP39A1, CYR61, DAG1, DAGLA, DAP3, DCBLD2, DENND1A, DHCR24, DMXL2, DPY19L4, DTNB, DTNBP1, EDAR, EGLN1, EHD3, ENOX1, EPHA2, EPHA4, ERMP1, ETV5, F3, FAM131C, FAM13B, FAT3, FBXO9, FBXW8, FOS, FRY, FUT10, GAS6, GCH1, GHITM, GHR, GIPR, GNAI1, GOLT1B, GPR17, GRAMD1B, GRHL3, GRIA1, GSTA5, GSTO1, GUCY1B3, HAUS8, HCN1, HDLBP, HERC1, HMMR, HSF1, HYDIN, ICAM4, ID1, ID3, IER5, IFIH1, IFT57, IFT74, IGF2R, IGSF1, IGSF3, INTS4, IQGAP3, IRS2, ITGA2B, ITGA7, JAG1, JAG2, JUP, KANK1, KBTBD12, KCNH2, KCNIP2, KIAA1211L, KIAA1549, KLHL23/PHOSPHO2-KLHL23, KLHL33, KNTC1, L3MBTL3, LAMA2, LAPTM4B, LATS2, LCA5, LDHA, LGMN, LIMCH1, LMAN1, LPCAT1, LRP1B, LRP6, LRRC4B, LRRC8E, LRRK2, LSM11, LYPD6B, MAPK6, MAST4, MBD5, MBP, MDK, MED19, MFN2, MGAT5, MGLL, MGMT, MLXIP, MMACHC, MMP27, MPI, MPP2, MRVI1, MTUS2, MYBPC3, MYBPH, MYH8, MYL4, MYOCD, MYOM3, NAV2, NCALD, NCAN, NEIL3, NFATC2, NID2, NOS1, NOTCH1, NPY1R, NR4A1, NR4A3, NTF3, ORAI3, OSGEPL1, OXCT1, PADI2, PAQR9, PARD3, PCDHGA3, PCK2, PDCD11, PDE1B, PDK3, PGM5, PHYHD1, PIF1, PIK3CA, PITPNM3, PITRM1, PKP4, PLOD1, PLXNB1, PNPLA3, PPA2, PPARGC1B, PRKG2, PRPF3, PTER, PTGS1, PTPRF, PYGL, RABGAP1, RADIL, RALGPS2, RANBP17, RAP1GAP2, RAPSN, RASGEF1B, RASIP1, RBM14, RC3H2, RCSD1, RELN, REV3L, RGS7, RGS9, RHOBTB1, RIC3, RIN2, RORC, SALL2, SARDH, SATB1, SCN10A, SCUBE2, SDK2, SEC31A, SEMA3C, SERF2, SETDB1, SGK1, SIPA1L2, SLC12A5, SLC12A6, SLC17A9, SLC25A25, SLC35F5, SLC38A2, SLC39A8, SLC45A4, SLC8A2, SLC8A3, SLIT3, SMYD2, SPON1, SRA1, STK17B, STK32B, STON2, SYDE2, SYTL2, TACC2, TANC1, TBC1D1, TBC1D16, TBKBP1, TBX20, TBX5, TCOF1, TGFA, THAP4, TJAP1, TLCD1, TLK1, TMCC2, TMEM108, TMEM50B, TMEM82, TMPRSS13, TMTC1, TNK2, TNNT2, TNXB, TPBG, TRIM9, TRO, TSC1, TSPAN7, TSPYL4, TTYH3, TYMP, UBR3, VDR, VEGFA, VLDLR, WNK1, ZBTB26, ZFHX2, ZFPL1, ZFPM2, ZNF385B
Liver Hyperplasia/ Hyperproliferation	Liver Cancer	3.62E-08	332	*ABCA8, ACOT7, ACSL6, ACSS2, ACTG1, ACVR1, ACY1, ADAMTS1, ADAMTS19, ADAMTSL1, ADAR, ADIPOR1, AEBP1, AGRN, AGT, AIG1, AK2, AKT2, ALDH1A3, ALDOB, AMN1, ANK3, ANLN, ANO4, AOX1, APIP, ARFGAP1, ARHGAP12, ARHGEF9, ARV1, ARVCF, ASAP3, ASB11, ASB14, ATG16L2, ATL2, ATP1A1, ATP1A2, ATP8A2, BAZ2A, BCL2, BDH1, BLVRA, BTBD11, BUB1, C20orf196, C3orf70, CACNA2D1, CAMK2G, CARD11, CARM1, CCNG1, CCRL2, CD82, CDCA3, CDH13, CDK14, CDK5R1, CEACAM16, CENPF, CHD3, CHMP4C, CLASP1, CLCN3, CLEC4G, CNTFR, COL20A1, COL27A1, COL4A4, COL4A5, CPT1B, CREB3L2, CRIP3, CSNK1E, CTSD, CXCL2, CYP39A1, CYR61, DAG1, DAGLA, DAP3, DCBLD2, DENND1A, DHCR24, DMXL2, DPY19L4, DTNB, DTNBP1, EDAR, EGLN1, EHD3, ENOX1, EPHA2, EPHA4, ERMP1, ETV5, F3, FAM131C, FAM13B, FAT3, FBXO9, FBXW8, FOS,*

Categories	Diseases or Functions Annotation	*P* Value	# Molecules	**Molecules**
				FRY, FUT10, GAS6, GCH1, GHITM, GHR, GIPR, GNAI1, GOLT1B, GPR17, GRAMD1B, GRHL3, GRIA1, GSTA5, GSTO1, GUCY1B3, HAUS8, HCN1, HDLBP, HERC1, HMMR, HSF1, HYDIN, ICAM4, ID1, ID3, IER5, IFIH1, IFT57, IFT74, IGF2R, IGSF1, IGSF3, IL15RA, INTS4, IQGAP3, IRS2, ITGA2B, ITGA7, JAG1, JAG2, JUP, KANK1, KBTBD12, KCNH2, KCNIP2, KIAA1211L, KIAA1549, KLHL23/PHOSPHO2-KLHL23, KLHL33, KNTC1, L3MBTL3, LAMA2, LAPTM4B, LATS2, LCA5, LDHA, LGMN, LIMCH1, LMAN1, LPCAT1, LRG1, LRP1B, LRP6, LRRC4B, LRRC8E, LRRK2, LSM11, LYPD6B, MAPK6, MAST4, MBD5, MBP, MDK, MED19, MFN2, MGAT5, MGLL, MGMT, MLXIP, MMACHC, MMP27, MPI, MPP2, MRVI1, MTUS2, MYBPC3, MYBPH, MYH8, MYL4, MYOCD, MYOM3, NAV2, NCALD, NCAN, NEIL3, NFATC2, NID2, NKD1, NOS1, NOTCH1, NPY1R, NR4A1, NR4A3, NTF3, ORAI3, OSGEPL1, OXCT1, PADI2, PAQR9, PARD3, PCDHGA3, PCK2, PDCD11, PDE1B, PDK3, PGM5, PHYHD1, PIF1, PIK3CA, PITPNM3, PITRM1, PKP4, PLOD1, PLXNB1, PNPLA3, POLG, PPA2, PPARGC1B, PRKG2, PRPF3, PSIP1, PTER, PTGS1, PTPRF, PYGL, RABGAP1, RADIL, RALGPS2, RANBP17, RAP1GAP2, RAPSN, RASGEF1B, RASIP1, RBM14, RC3H2, RCSD1, RELN, REV3L, RGS7, RGS9, RHOBTB1, RIC3, RIN2, RORC, SALL2, SARDH, SATB1, SCN10A, SCUBE2, SDK2, SEC31A, SEMA3C, SERF2, SETDB1, SGK1, SIPA1L2, SLC12A5, SLC12A6, SLC17A9, SLC25A25, SLC35F5, SLC38A2, SLC39A8, SLC45A4, SLC8A2, SLC8A3, SLIT3, SMYD2, SPON1, SRA1, STK17B, STK32B, STON2, SYDE2, SYTL2, TACC2, TANC1, TBC1D1, TBC1D16, TBKBP1, TBX20, TBX5, TCOF1, TGFA, THAP4, TJAP1, TLCD1, TLK1, TMCC2, TMEM108, TMEM50B, TMEM82, TMPRSS13, TMTC1, TNK2, TNNT2, TNXB, TPBG, TRIM28, TRIM9, TRO, TSC1, TSPAN7, TSPYL4, TTYH3, TUBA1C, TUBA4A, TYMP, UBR3, VDR, VEGFA, VLDLR, WNK1, ZBTB26, ZFHX2, ZFPL1, ZFPM2, ZNF385B
Cardiac Arrhythmia	Familial Arrhythmia	1.75E-06	13	*ADRB1, CACNA2D1, DSG2, JUP, KCND3, KCNH2, KCNQ1, MYBPC3, MYL4, NPPA, SCN10A, SCN2B, SCN4B*
Cardiac Arrhythmia	Arrhythmia	6.54E-06	28	*ADORA1, ADRB1, AGT, ATP1A1, ATP1A2, CACNA2D1, DSG2, FHL2, HCN1, JUP, KCND3, KCNH2, KCNIP2, KCNQ1, MYBPC2, MYBPC3, MYL4, NPPA, PITX2, PTGS1, SCN10A, SCN2B, SCN4B, TBX5, TNNT2, TUBA1C, TUBA4A, VEGFA*
Cardiac Arrhythmia, Tachycardia	Tachycardia	1.14E-05	15	*ADORA1, ADRB1, AGT, ATP1A1, ATP1A2, DSG2, JUP, KCNH2, KCNQ1, MYBPC3, NPPA, PITX2, SCN4B, TBX5, TNNT2*
Cardiac Enlargement	Hypertrophy of Heart	3.01E-05	37	*ADCY6, ADORA1, ADRB1, AGT, BCL2, CACNA2D1, CTGF, CTSD, CYP2J2, DTNBP1, DUSP5, EGLN1, FHL2, GPX3, IGF2R, INHA, KCNQ1, MORF4L1, MYBPC3, MYOCD, NFATC2, NOS1, NPPA, NR4A3, NTF3, P2RX4, PIK3CA, PLIN5, PPARGC1B, PRKG1, RAB2A, RHEB, SLC25A4, SMAD7, TBX5, TNNT2, VDR*
Cardiac Enlargement	Hypertrophy of Heart Cells	3.68E-05	20	*ADCY6, ADORA1, ADRB1, AGT, CTGF, CTSD, DTNBP1, DUSP5, FHL2, GPX3, IGF2R, INHA, MYBPC3, MYOCD, NFATC2, NPPA, PRKG1, RAB2A, SMAD7, TBX5*
Cardiac Arrhythmia	Supraventricular Arrhythmia	9.61E-05	18	*ADORA1, ADRB1, AGT, ATP1A1, ATP1A2, HCN1, KCNH2, KCNQ1, MYBPC2, MYL4, NPPA, PITX2, PTGS1, SCN2B, SCN4B, TUBA1C, TUBA4A, VEGFA*
Cardiac Arrhythmia	Familial Atrial Fibrillation	1.50E-04	5	*KCNQ1, MYL4, NPPA, SCN2B, SCN4B*

Categories	Diseases or Functions Annotation	*P* Value	# Molecules	**Molecules**
Cardiac Arrhythmia, Tachycardia	Ventricular Tachycardia	2.09E-04	11	*ADRB1, ATP1A1, DSG2, JUP, KCNH2, KCNQ1, MYBPC3, NPPA, SCN4B, TBX5, TNNT2*
Cardiac Arrhythmia	Cardiac Fibrillation	2.20E-04	18	*ADRB1, AGT, ATP1A1, ATP1A2, DSG2, JUP, KCNH2, KCNQ1, MYBPC2, MYL4, NPPA, PITX2, PTGS1, SCN2B, SCN4B, TUBA1C, TUBA4A, VEGFA*
Cardiac Arrhythmia	Inherited Primary Arrhythmia Syndrome	2.23E-04	8	*CACNA2D1, DSG2, KCND3, KCNH2, KCNQ1, MYBPC3, SCN10A, SCN4B*
Cardiac Arrhythmia, Tachycardia	Supraventricular Tachycardia	2.24E-04	7	*ADORA1, ADRB1, ATP1A1, ATP1A2, KCNH2, NPPA, PITX2*
Cardiac Arrhythmia	Brugada Syndrome	2.91E-04	6	*CACNA2D1, KCND3, KCNH2, KCNQ1, MYBPC3, SCN10A*
Cardiac Arrhythmia	Atrial Fibrillation	3.69E-04	16	*ADRB1, AGT, ATP1A1, ATP1A2, KCNH2, KCNQ1, MYBPC2, MYL4, NPPA, PITX2, PTGS1, SCN2B, SCN4B, TUBA1C, TUBA4A, VEGFA*
Heart Failure	Failure of Heart	4.59E-04	28	*ADRB1, ATP1A1, ATP1A2, CACNA2D1, CTGF, GABRA3, GHR, GSS, GUCY1B3, IDUA, IGF2R, KCND3, KCNH2, LIMS2, MYBPC3, NOS1, NPPA, PDGFB, PPA2, PPARGC1B, PTGS1, RXFP1, SLC12A5, TFAM, TNNT2, VDR, VEGFA, ZFPM2*
Cardiac Congestive Cardiac Failure, Heart Failure	Congestive Heart Failure	4.95E-04	16	*ADRB1, ATP1A1, ATP1A2, CACNA2D1, GSS, GUCY1B3, IDUA, IGF2R, KCNH2, NOS1, NPPA, PDGFB, PPARGC1B, RXFP1, TNNT2, ZFPM2*
Cardiac Arteriopathy	Coronary Artery Disease	7.44E-04	29	*ADORA1, ADRB1, AGT, ALDH5A1, ANKRD29, CACNA2D1, CDH13, CTNNBL1, FOS, GABRA3, GFOD1, GRIA1, GSS, GSTO1, GUCY1B3, ITGA2B, JUP, LRP6, NOS1, PRKG1, PTGS1, RCSD1, SCN10A, TNNT2, TSPO, TUBA1C, TUBA4A, VDR, VEGFA*
Cardiac Arrhythmia	Arrhythmia of Heart Ventricle	8.40E-04	12	*ADRB1, ATP1A1, DSG2, JUP, KCNH2, KCNQ1, MYBPC3, NPPA, SCN10A, SCN4B, TBX5, TNNT2*
Hepatocellular Carcinoma, Liver Hyperplasia/ Hyperproliferation	Hepatocellular Carcinoma	8.65E-04	64	*ABCA8, ACSS2, ACTG1, ACY1, ADAMTS1, ADAR, AGT, ANLN, ATP1A1, ATP8A2, BCL2, BUB1, C3orf70, CACNA2D1, CCNG1, CD82, CDK14, CENPF, CLEC4G, CTSD, CXCL2, CYP39A1, EHD3, EPHA2, F3, FOS, FRY, GCH1, GHITM, GHR, GSTO1, ID1, IFT57, IGF2R, JAG1, JAG2, JUP, KIAA1549, LAPTM4B, MDK, MGLL, MGMT, NEIL3, NFATC2, NOTCH1, NPY1R, PIK3CA, PNPLA3, PTGS1, RAP1GAP2, RASGEF1B, RELN, RORC, SCUBE2, SERF2, SLC25A25, SPON1, STK17B, TGFA, TSPAN7, TYMP, VDR, VEGFA, ZFPM2*
Kidney Failure	Failure of Kidney	8.72E-04	24	*ADORA1, ADRB1, AGT, ATP1A1, BCL2, CACNA2D1, CYR61, GABRA3, GHR, GRIA1, GSS, GUCY1B3, IRF4, KCP, LGMN, Mcpt4, MDK, MR1, NPPA, PTGS1, SYT7, TNNT2, VDR, VEGFA*
Cardiac Arrhythmia	Brugada Syndrome Type 1	9.49E-04	5	*CACNA2D1, KCND3, KCNH2, MYBPC3, SCN10A*
Cardiac Arrhythmia, Tachycardia	Polymorphic Ventricular Tachycardia	9.49E-04	5	*DSG2, KCNH2, KCNQ1, MYBPC3, SCN4B*
Cardiac Enlargement	Hypertrophy of Cardiomyocytes	1.16E-03	15	*ADCY6, ADORA1, ADRB1, AGT, CTGF, DTNBP1, DUSP5, FHL2, IGF2R, MYBPC3, MYOCD, NPPA, PRKG1, SMAD7, TBX5*

Ingenuity Pathway Analysis was performed on the differentially expressed genes from the RNA-sequencing data set for iNICD LA (n = 6) compared to control LA (n = 6) and the top 25 statistically significant results for diseases or function network categories were compiled into the table above. Of the top 25 statistically significant categories, 14 are

related to arrhythmias, 8 of which are not specific to the ventricles. Of those 8, 4 of are specific to atrial arrhythmias.

Table S13. Sharp microelectrode values for murine electrophysiology experiments

Condition	N	RMP (mV)	dV_m/dt_{max} (V/sec)	APA (mV)	APD_{20} (ms)	APD_{50} (ms)	APD_{70} (ms)	APD_{90} (ms)	ERP (ms)
Control LA (sinus rhythm)	9	-78.0 (±0.7)	209.0 (±6.8)	90.9 (±1.3)	2.8 (±0.2)	7.1 (±0.4)	11.9 (±0.7)	22.3 (±1.1)	-
iNICD LA (sinus rhythm)	10	-78.4 (±0.5)	219.7 (±9.0)	94.9 (±1.9)	4.2 (±0.3)	10.4 (±0.4)	16.4 (±0.5)	28.2 (±0.9)	-
Control LA (10Hz pacing)	8	-78.6 (±1.1)	211.7 (±8.0)	87.9 (±2.3)	2.9 (±0.2)	7.5 (±0.4)	12.2 (±0.8)	22.3 (±1.5)	15.4[a] (±1.3)
iNICD LA (10 Hz pacing)	8	-78.0 (±0.3)	220.2 (±11.8)	93.7 (±2.2)	4.5 (±0.4)	11.3 (±0.7)	17.5 (±0.9)	29.6 (±1.6)	15.4[b] (±1.7)
Control LA baseline	7	-75.7 (±1.3)	184.8 (±11.8)	89.0 (±0.9)	3.7 (±0.3)	9.1 (±0.8)	16.5 (±1.7)	28.8 (±2.7)	-
Control LA +dofetilide	7	-75.3 (±0.4)	197.2 (±8.5)	86.1 (±1.8)	3.3 (±0.2)	8.6 (±0.6)	16.4 (±1.6)	30.0 (±3.1)	-
iNICD LA baseline	7	-76.3 (±0.5)	171.7 (±13.2)	89.2 (±2.5)	5.3 (±0.3)	11.0 (±0.5)	17.6 (±0.8)	29.3 (±1.2)	-
iNICD LA +dofetilide	7	-76.7 (±0.8)	152.7 (±10.8)	85.6 (±3.1)	5.4 (±0.4)	11.5 (±0.6)	18.3 (±0.8)	30.1 (±1.1)	-

All data are shown as the average ± SEM. [a]*n* = 7. [b]*n* = 6

Table S14. RT-qPCR Mouse Primer Sequences

Gene	Forward Sequence	Reverse Sequence
Actc1	5' GAC CTC ACT GAC TAC CTC ATG 3'	5' TCT CGT TCT CAA AAT CCA GGG 3'
Dll1	5' CCC TCT GTG TCT TAT CTC CTT TC 3'	5' TTC CTC TTC GTC TGG CTT TC 3'
Dll4	5' CTC CTG TGG GCA TCT GTA TTT 3'	5' CCT CCT CTC TGC TTT CTC ATT T 3'
Etv5	5' AAG TGA TAG CGG AGA CTT TGG 3'	5' GAG ATT TCC CTG GGA CCA TAA A 3'
Etv6	5' GGA GAG GAA AGG AAA GTG GAA A 3'	5' TCG TTC CTG CTT AAT GCT AGA C 3'
Gata3	5' GAC TGA GAG AGC GAG ACA TAG A 3'	5' TTC CGA TCA CCT GAG TAG CA 3'
Gata4	5' CGA GGG TGA GCC TGT ATG TAA 3'	5' GCT AGT GGC ATT GCT GGA GT 3'
Gata6	5' CAG CAC AGA CCT GTT GGA G 3'	5' CTG CTG AGG CCA TTC ATC TT 3'
Gatad2b	5' GTG GGA CAA CAA GGC TTA TCT A 3'	5' TGA CAC TGT GAC CCT GTA ATG 3'
Hes1	5' AAA GCC TAT CAT GGA GAA GAG GCG 3'	5' GGA ATG CCG GGA GCT ATC TTT CTT 3'
Hey1	5' GAA GCG CCG ACG AGA CCG AAT CAA 3'	5' CAG GGC GTG CGC GTC AAA ATA ACC 3'
Hey2	5’ CGA CGT GGG GAG CGA GAA CAA T 3’	5’ GGC AAG AGC ATG GGC ATC AAA GTA 3’
Hopx	5' AGG AGC AGA CGC AGA AAT G 3'	5' GAA GAA GAG ATG GAG TTC CAA GAG 3'
Jag1	5' CTA CTG TGA TTG CCT TCC TGG 3'	5' GTG GAC AGA TAC AGC GAT AAC C 3'
Jag2	5' TTG TTA TGG GTG GCT CTT CC 3'	5' GTG TCC ACC ATA CGC AGA TAA 3'
Jarid2	5' GAG ACC GCC AAG GAA ATG AA 3'	5' TGC TTG TGC AAT CTG GTA GAG 3'
Kcnd3	5' CCT AGC TCC AGC GGA CAA GA 3'	5' CCA CTT ACG TTG AGG ACG ATC A 3'
Kcnh2	5' GCT TAC TGC CCT CTA CTT CAT C 3'	5' CTT TCC AGG ACG GGC ATA TAG 3'
Kcnip2	5' GGC TGT ATC ACG AAG GAG GAA 3'	5' CCG TCC TTG TTT CTG TCC ATC 3'
Kcnq1	5’ CTG GGC TCT GTA GTC TTC ATT C 3’	5’ CTC GTT CAC CGC ATC TTT CT 3’
Nrarp	5' TCC TGG TTG GTC TCT TCT ATC T 3'	5' CAC GAT GGG CTA CAA GTC TAT ATC 3'
Notch1	5' TGA AGA ATG CCT CAG ATG GTG 3'	5' GAG AAC TAC TGG CTC CTC AAA C 3'
Notch2	5' AAC TGT CAG ACC CTG GTG AAC 3'	5' CGA CAA GTG TAG CCT CCA ATC 3'
Notch3	5' TTG GGT CGG TGT GTG AAT AC 3'	5' CGG AGA GAC ACT CAT TGA CAT C 3'
Notch4	5' AAG CTC CCG TAG TCC TAC TT 3'	5' AGC TCT TCC AGA TGG GTT TG 3'
Pitx2	5' CCT GAA GTC GCA GAG AAA GAT AA 3'	5' GGC TAG TGA AAT GAG TCC TCT G 3'
Pparg	5' CCC TGG CAA AGC ATT TGT ATG 3'	5' CAA ATG GTG ATT TGT CCG TTG T 3'

Gene	Forward Sequence	Reverse Sequence
Scn5a	5' GAA GAA GCT GGG CTC CAA GA 3'	5' CAT CGA AGG CCT GCT TGG TC 3'
Shox2	5' CCG AGT ACA GGT TTG GTT TCA 3'	5' TGG CTA GCG GCT CCT ATA A 3'
Tbp	5' GGG ATT CAG GAA GAC CAC ATA G 3'	5' CCT CAC CAA CTG TAC CAT CAG 3'
Tbx5	5' ACC TGG ACC CGT TTG GAC ACA TTA 3'	5' ACG CAG TGT TCT TTG AAC CGA ACC 3'
Tbx18	5' TTC ACA ACT GTC ACT GCC TAT C 3'	5' CAG AGC TTC CAA ACC CAT TCT 3'
